# Supplementary figures and images for: Herbivorous insects independently evolved salivary effectors to regulate plant immunity by destabilizing the malectin-LRR RLP NtRLP4 (part 1 of 2)
Source: eLife. 2026 May 5;14:RP108737. doi: 10.7554/eLife.108737 (PMC13143284; doi:10.7554/eLife.108737)

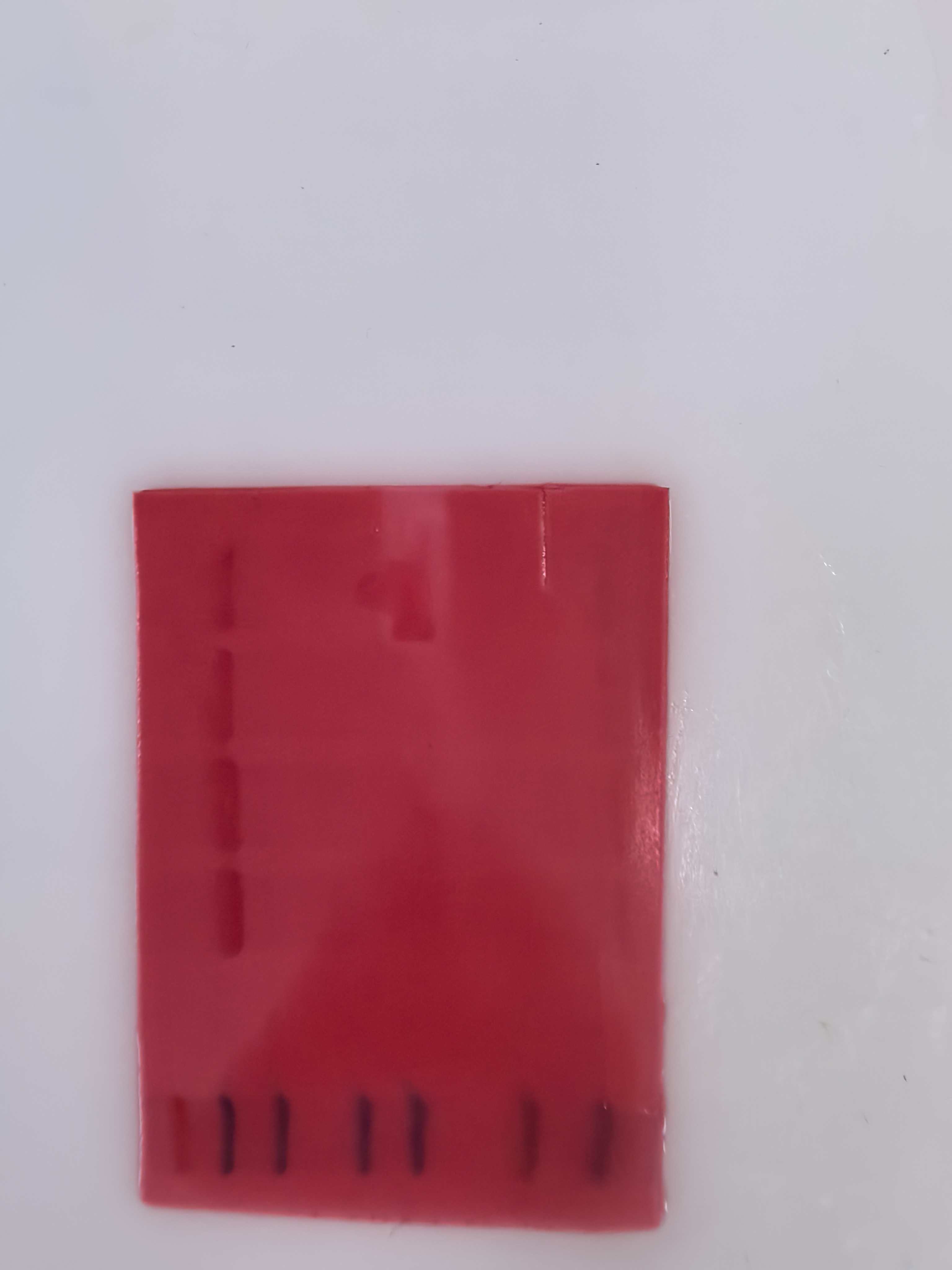

Supplement: Figure 1—source data 2. [file elife-108737-fig1-data2.zip › Figure 1—source data 2/RbCL.jpg]

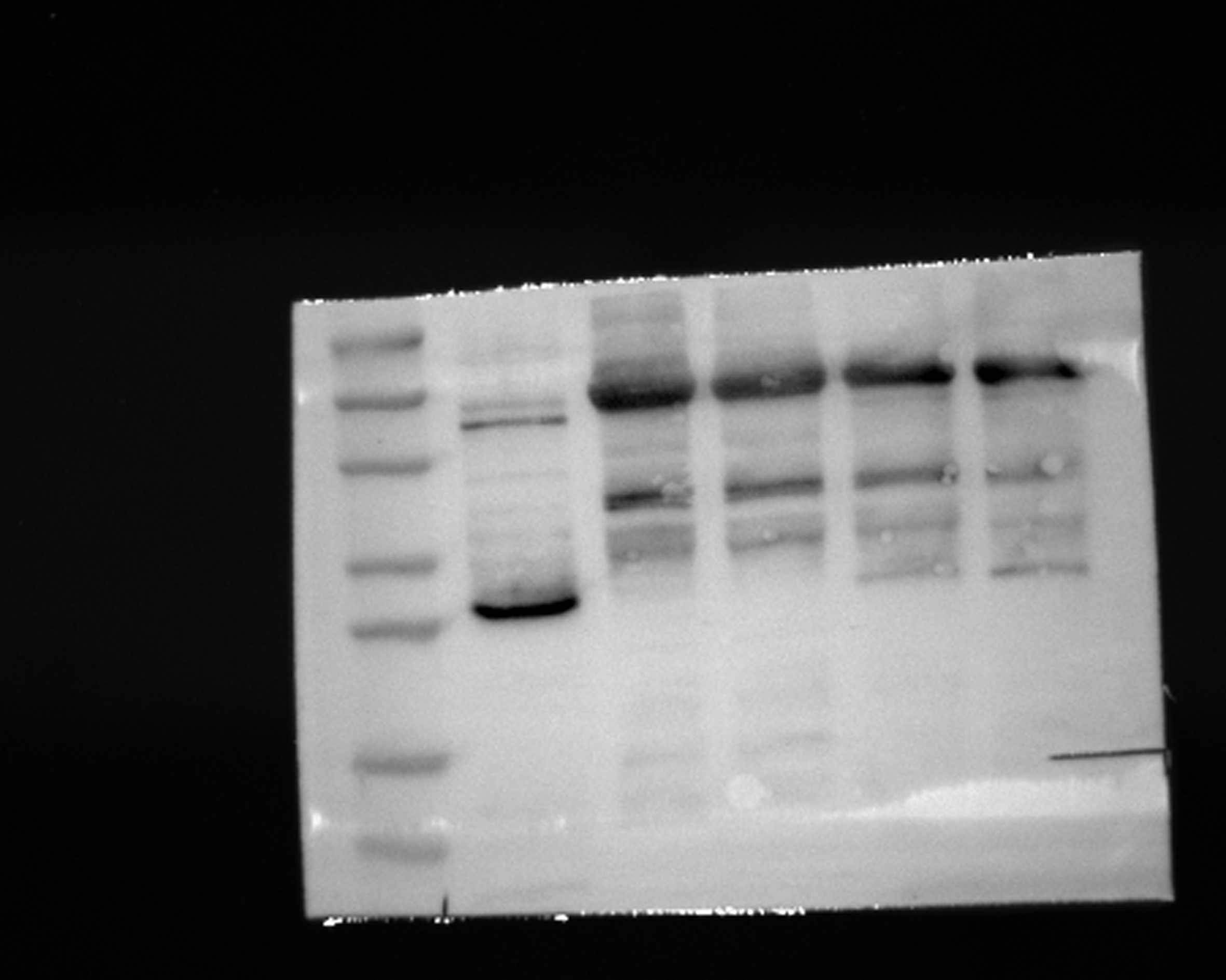

Supplement: Figure 1—source data 2. [file elife-108737-fig1-data2.zip › Figure 1—source data 2/α-BtRDP.tif]

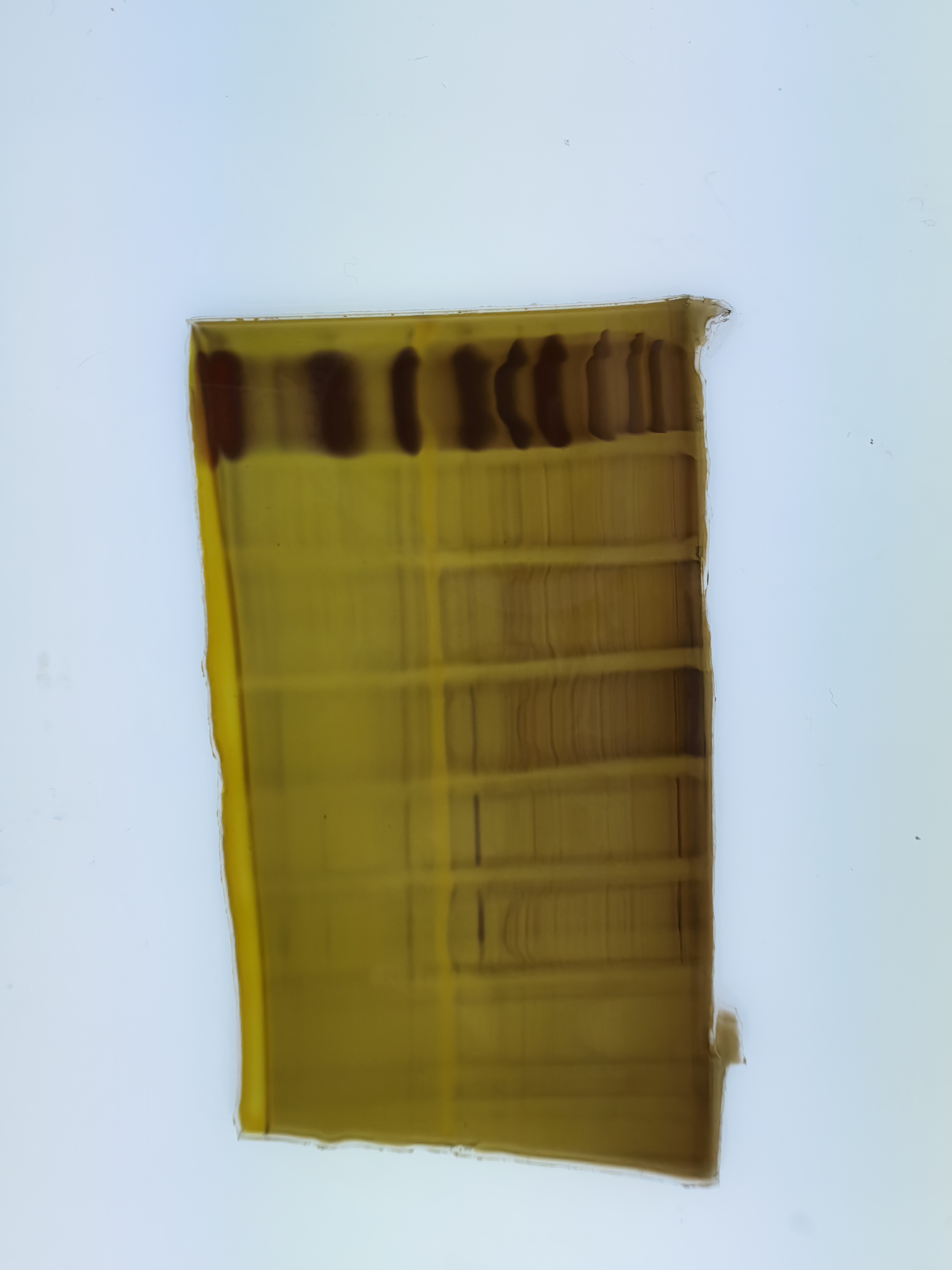

Supplement: Figure 1—source data 4. [file elife-108737-fig1-data4.zip › Figure 1—source data 4/silver stain.jpg]

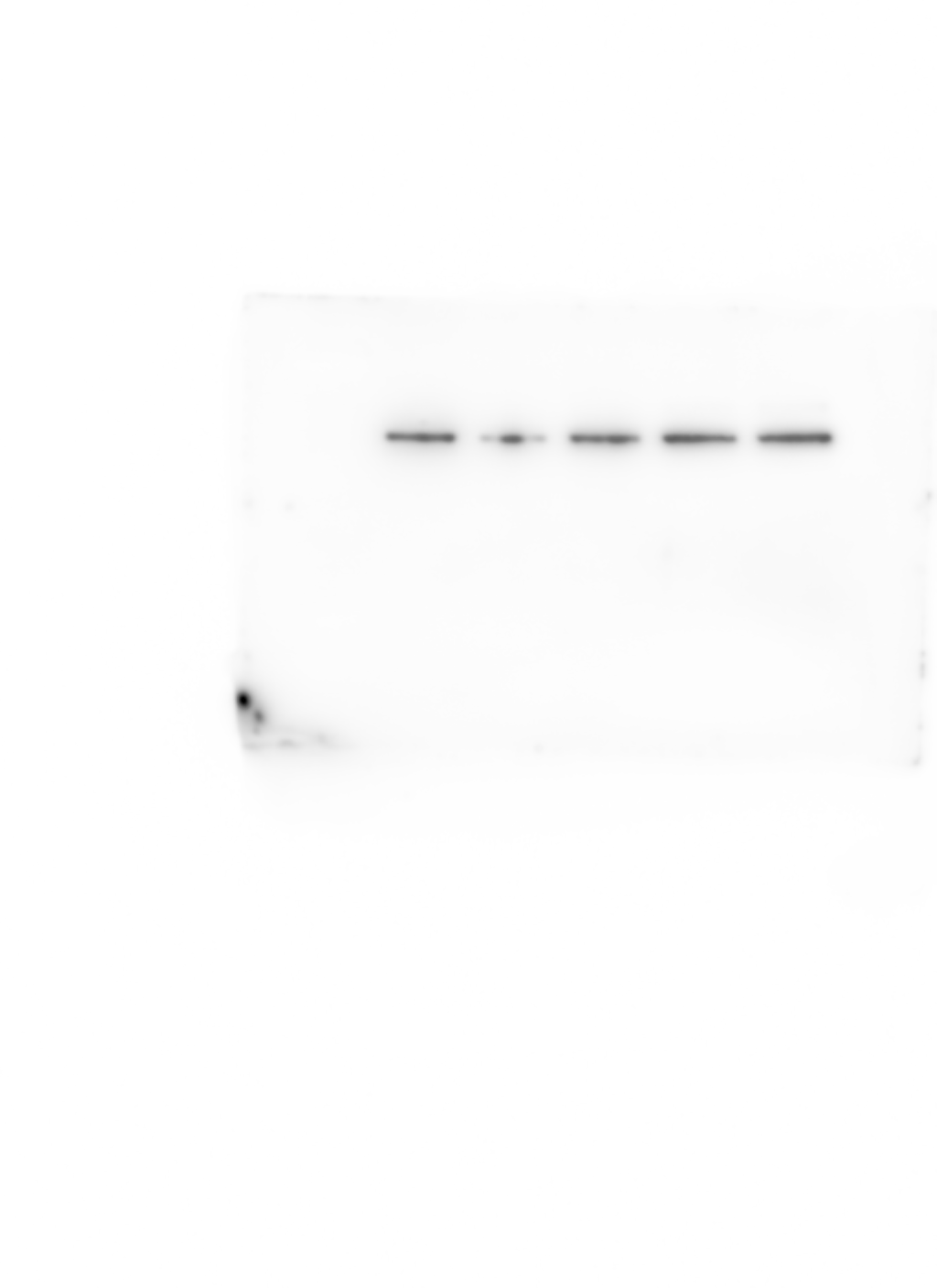

Supplement: Figure 1—source data 4. [file elife-108737-fig1-data4.zip › Figure 1—source data 4/α-actin-blot.tif]

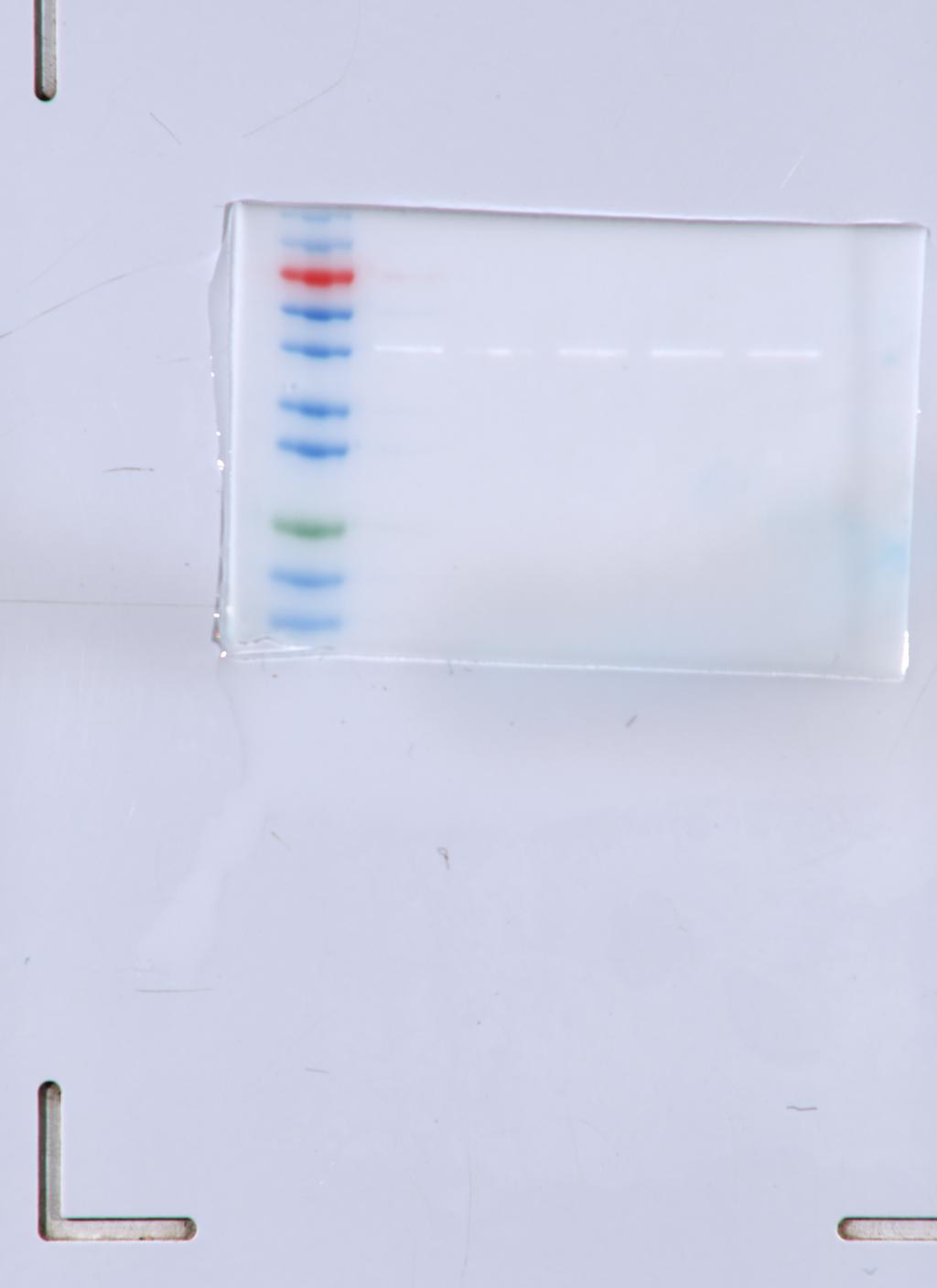

Supplement: Figure 1—source data 4. [file elife-108737-fig1-data4.zip › Figure 1—source data 4/α-actin-marker.jpg]

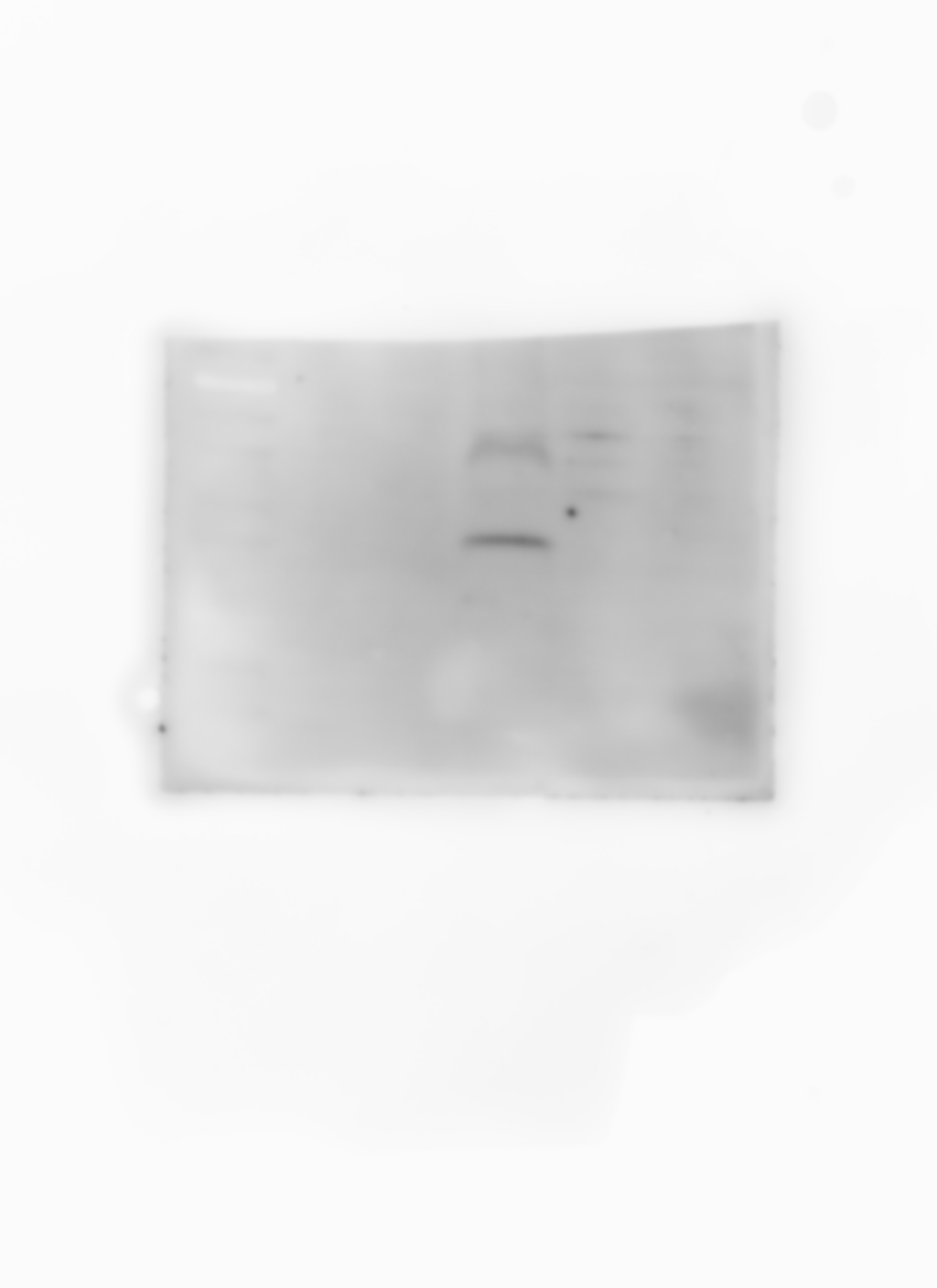

Supplement: Figure 1—source data 4. [file elife-108737-fig1-data4.zip › Figure 1—source data 4/α-BtRDP-blot.tif]

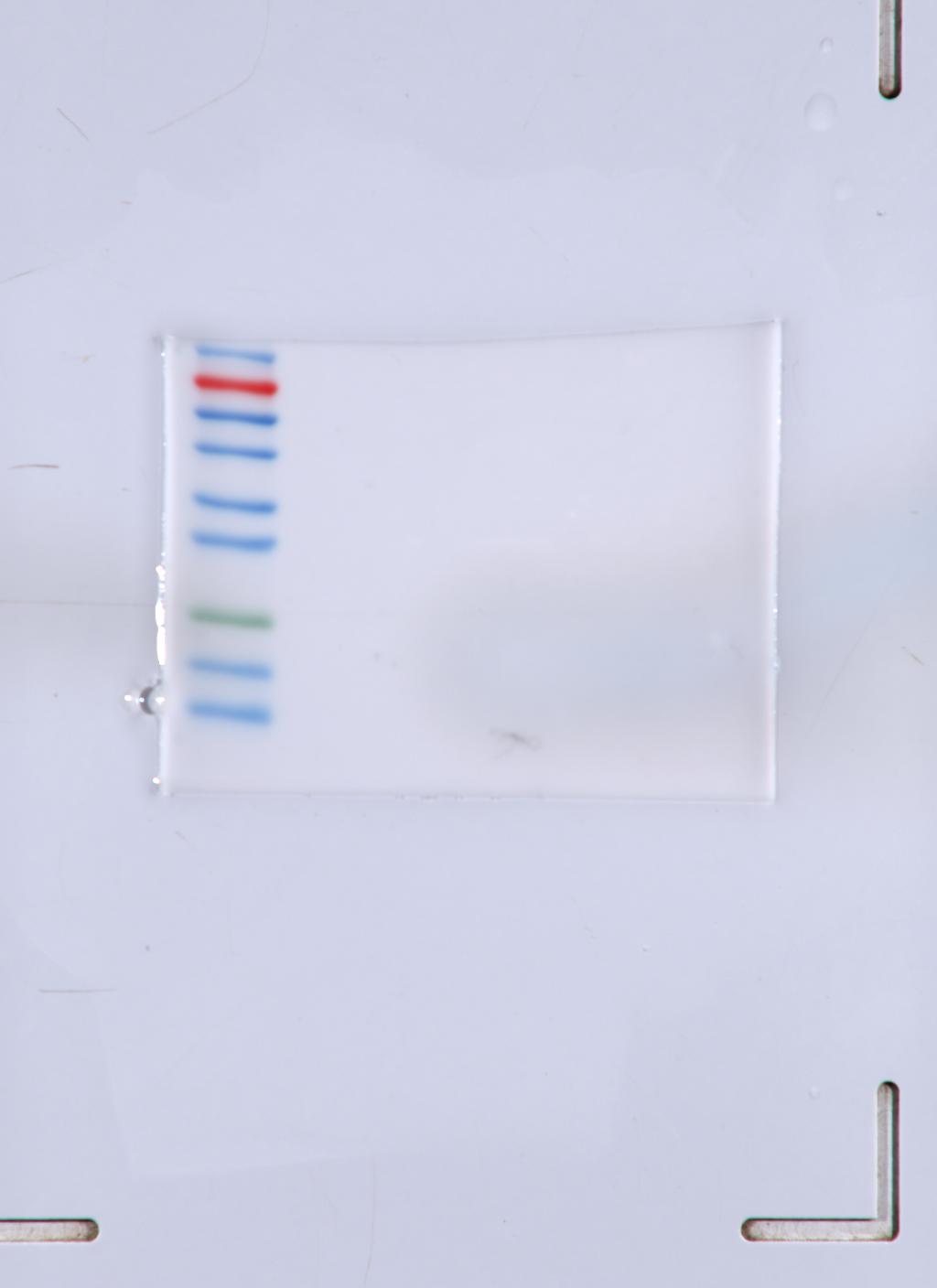

Supplement: Figure 1—source data 4. [file elife-108737-fig1-data4.zip › Figure 1—source data 4/α-BtRDP-Marker.jpg]

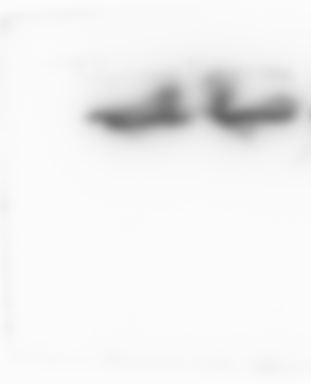

Supplement: Figure 2—source data 2. [file elife-108737-fig2-data2.zip › Figure 2—source data 2/α-actin-blot.tif]

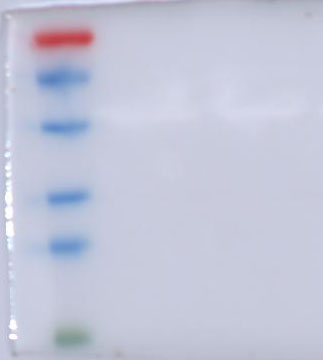

Supplement: Figure 2—source data 2. [file elife-108737-fig2-data2.zip › Figure 2—source data 2/α-actin-marker.jpg]

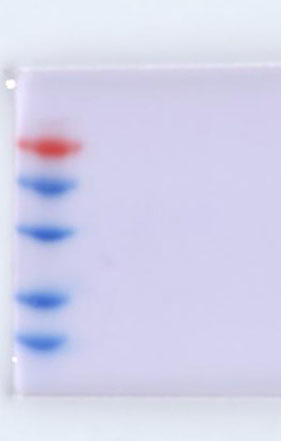

Supplement: Figure 2—source data 2. [file elife-108737-fig2-data2.zip › Figure 2—source data 2/α-BtRDP-blot-marker.jpg]

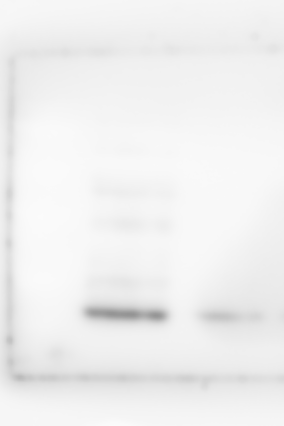

Supplement: Figure 2—source data 2. [file elife-108737-fig2-data2.zip › Figure 2—source data 2/α-BtRDP-blot.tif]

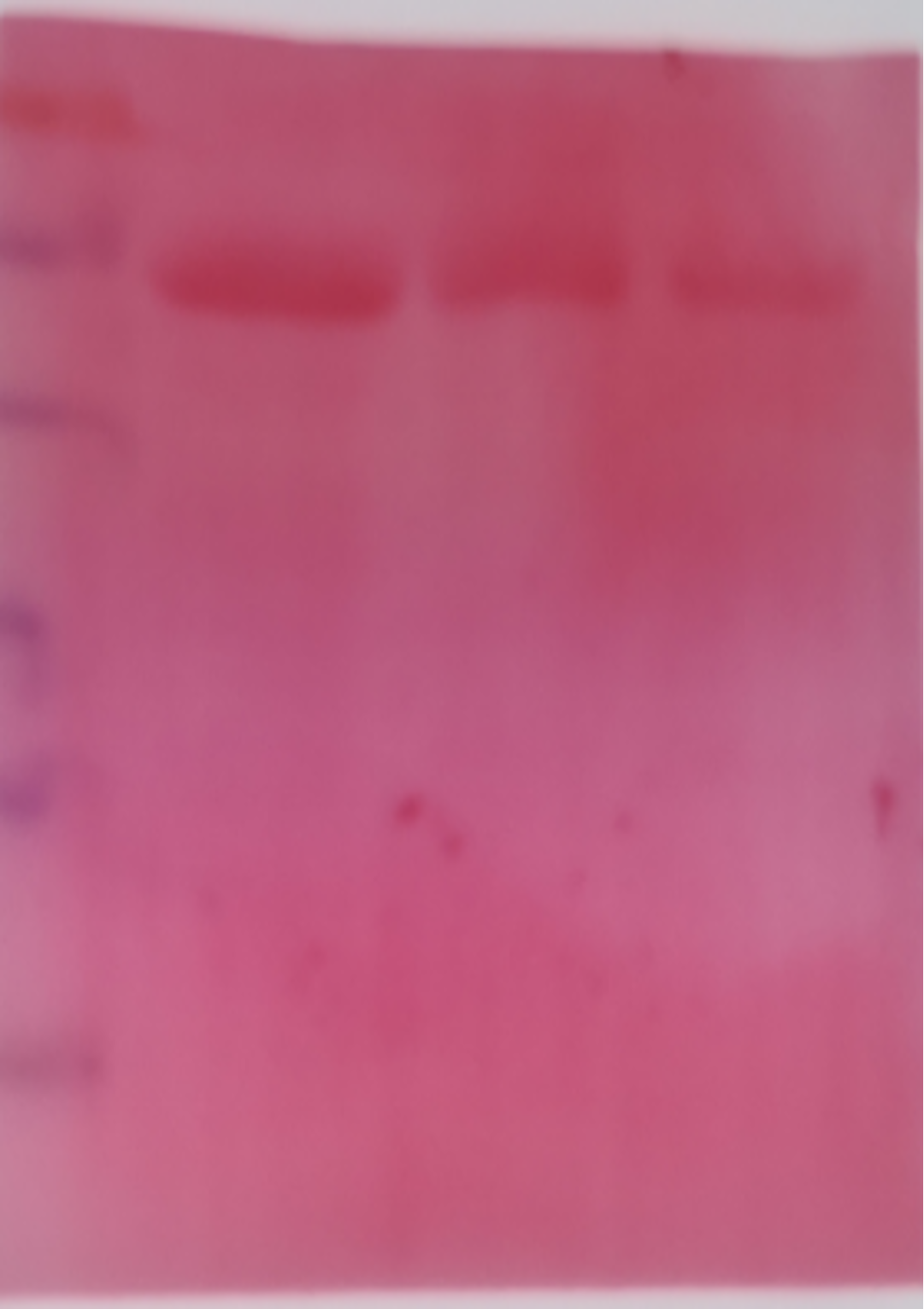

Supplement: Figure 2—source data 4. [file elife-108737-fig2-data4.zip › Figure 2—source data 4/RbCL.JPG]

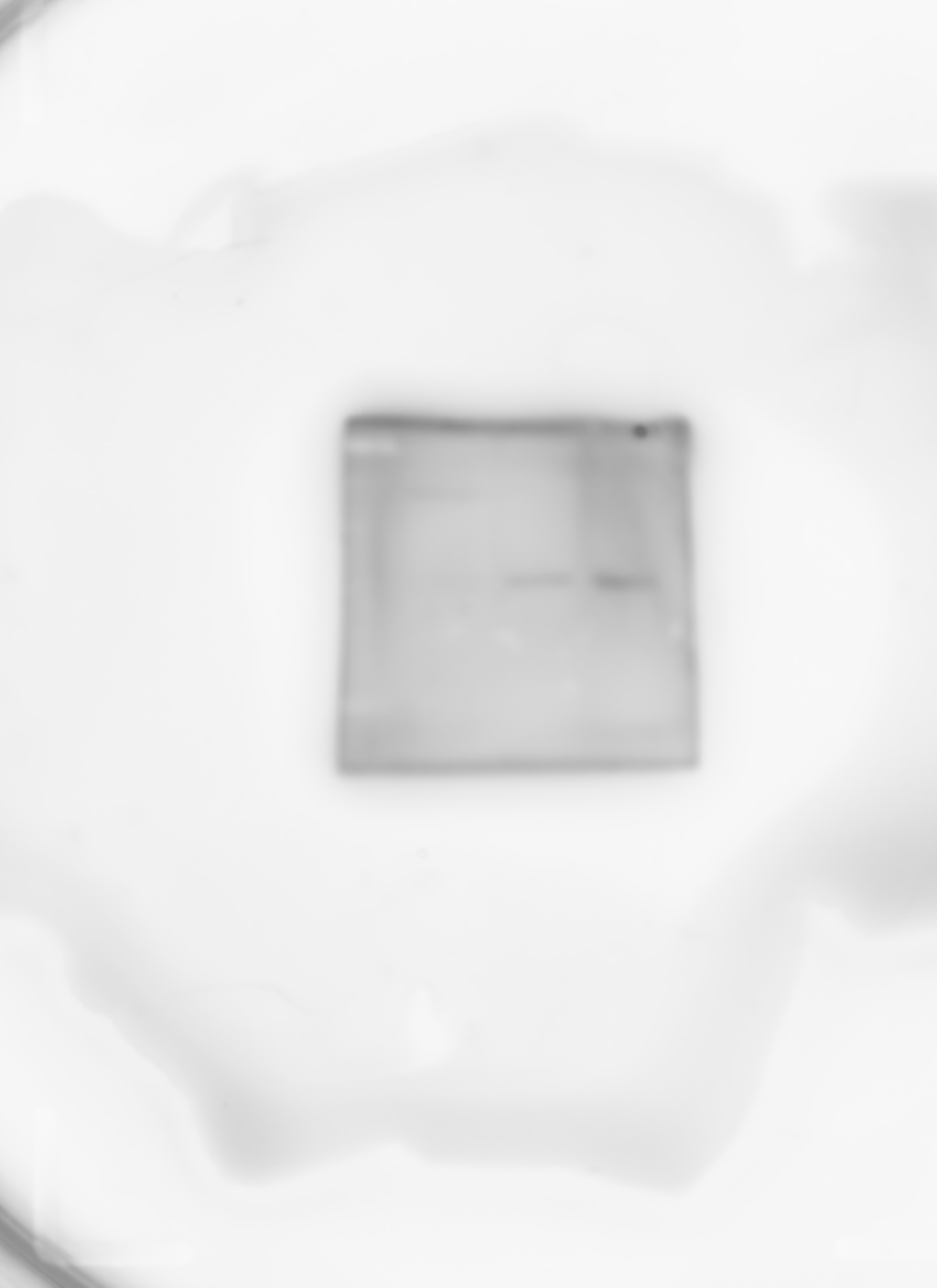

Supplement: Figure 2—source data 4. [file elife-108737-fig2-data4.zip › Figure 2—source data 4/α-BtRDP-blot.tif]

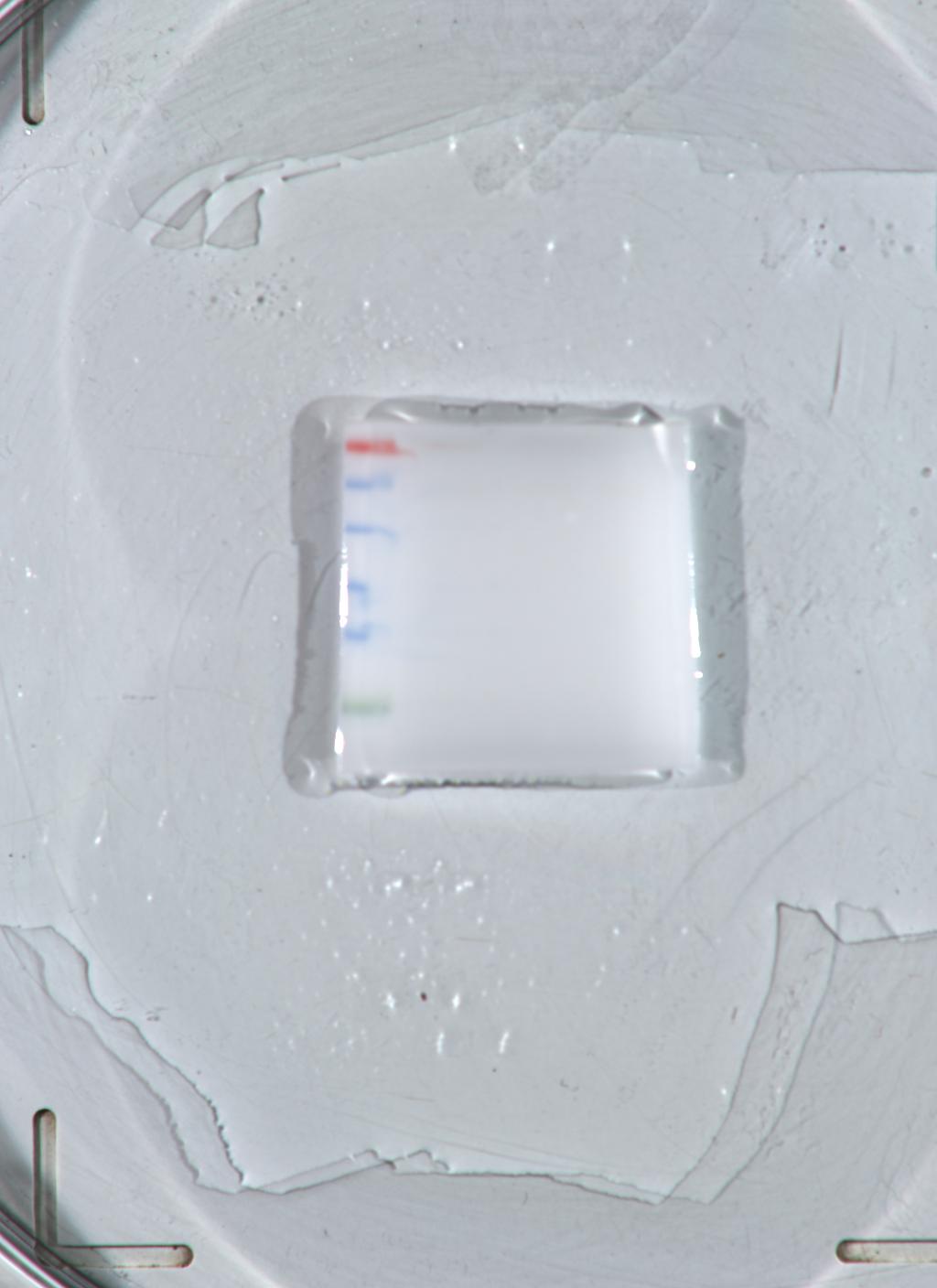

Supplement: Figure 2—source data 4. [file elife-108737-fig2-data4.zip › Figure 2—source data 4/α-BtRDP-marker.jpg]

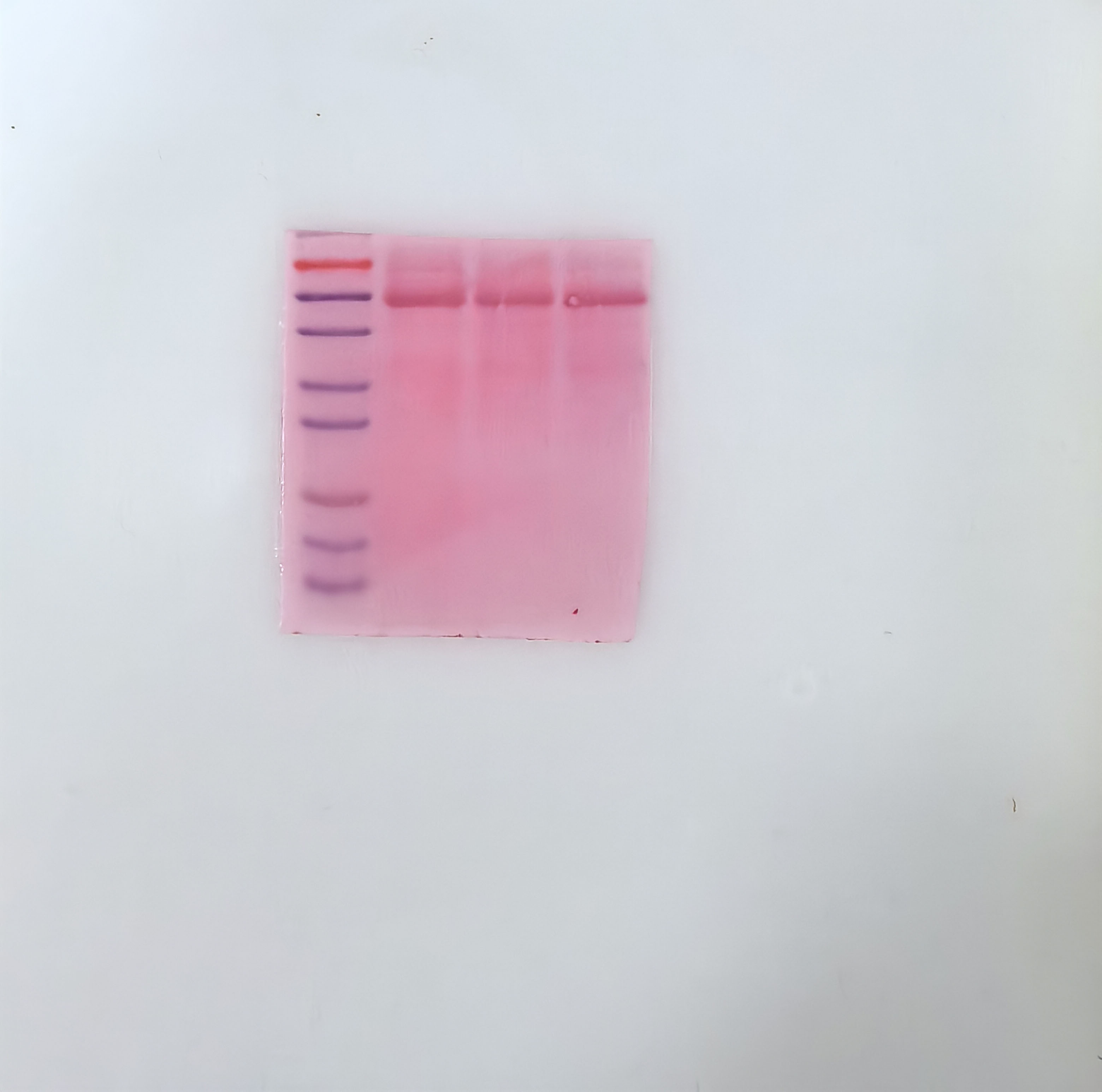

Supplement: Figure 2—source data 6. [file elife-108737-fig2-data6.zip › Figure 2—source data 6/RbCL.jpg]

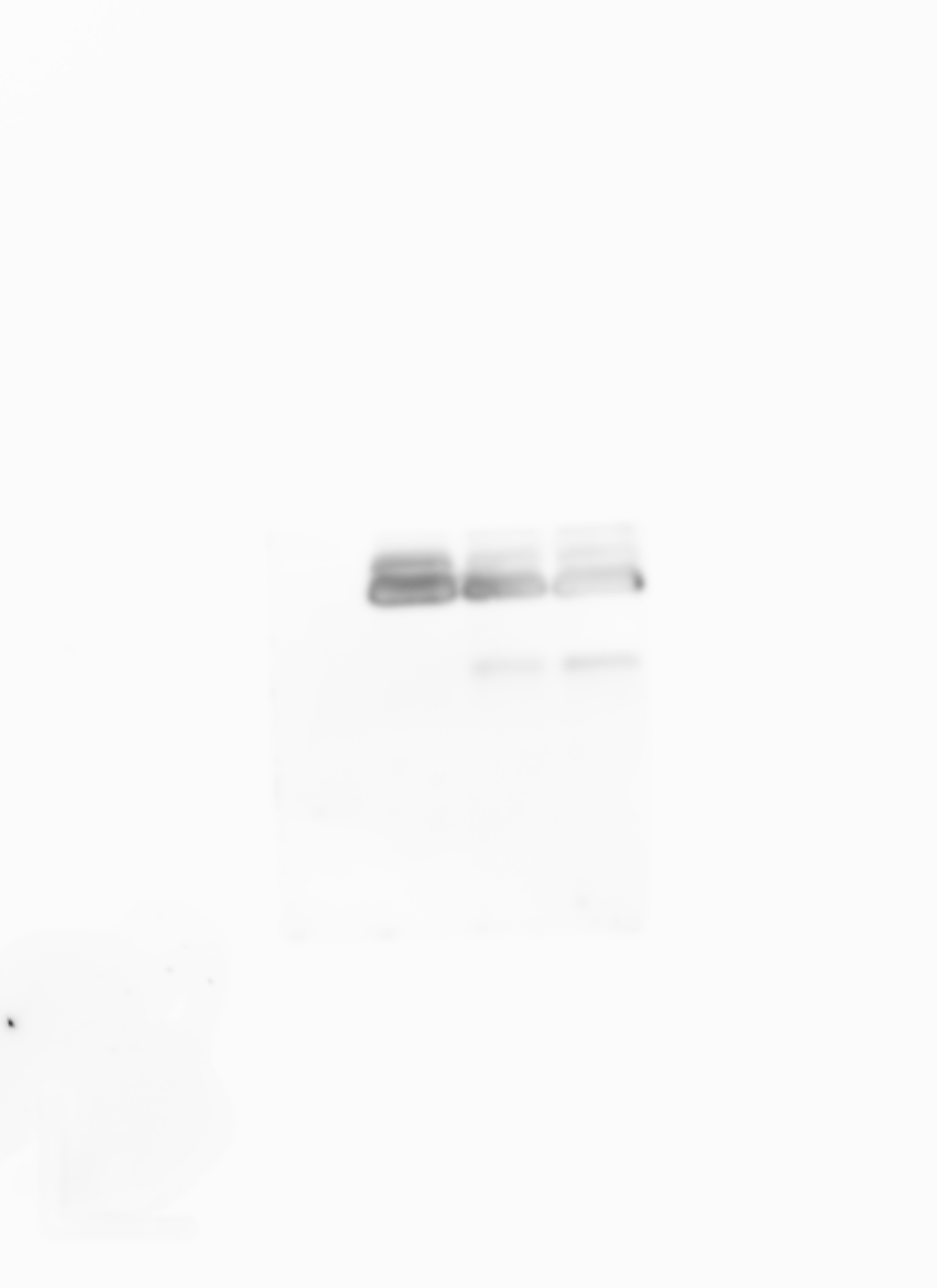

Supplement: Figure 2—source data 6. [file elife-108737-fig2-data6.zip › Figure 2—source data 6/α-BtRDP-blot.tif]

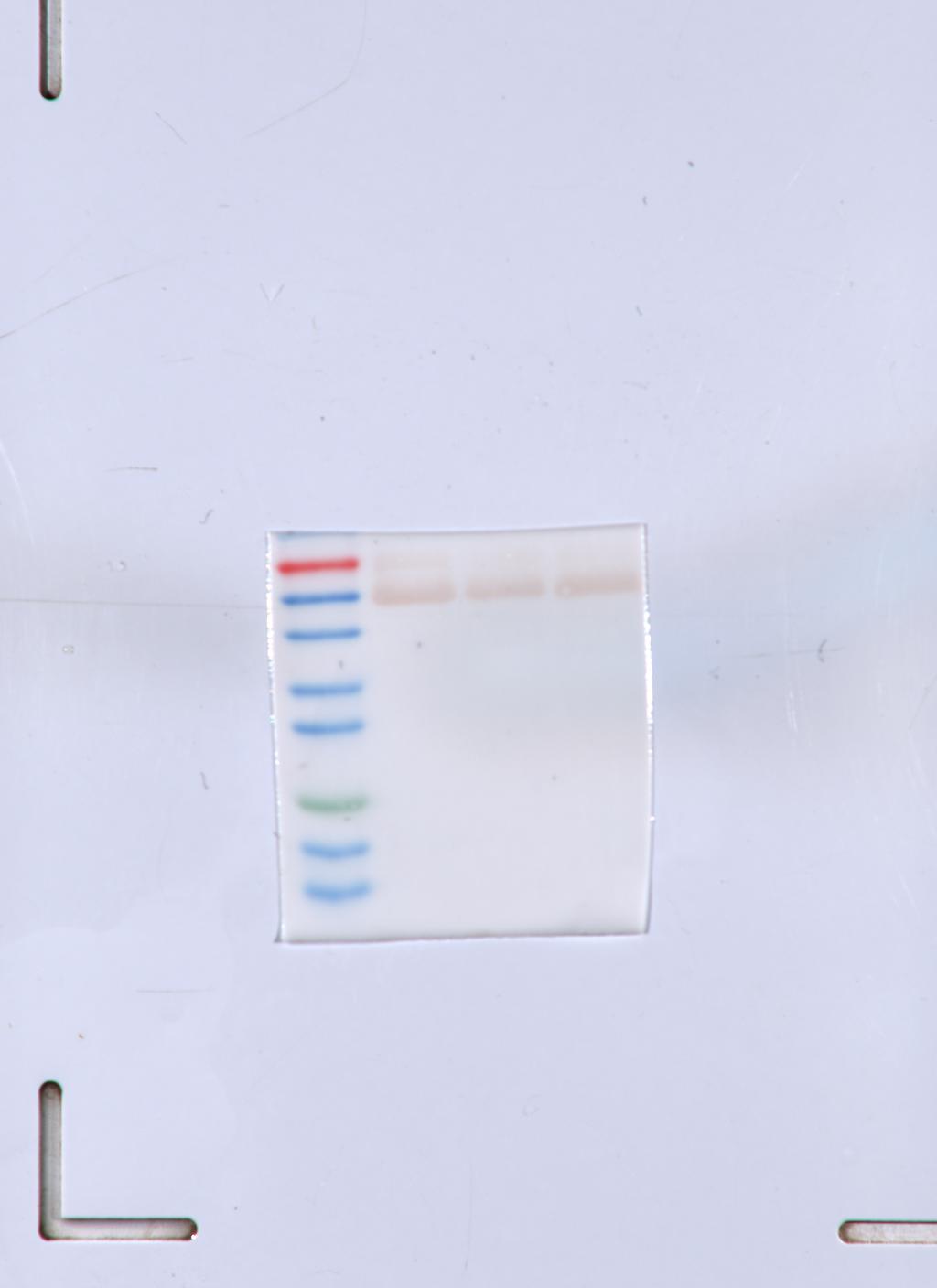

Supplement: Figure 2—source data 6. [file elife-108737-fig2-data6.zip › Figure 2—source data 6/α-BtRDP-marker.jpg]

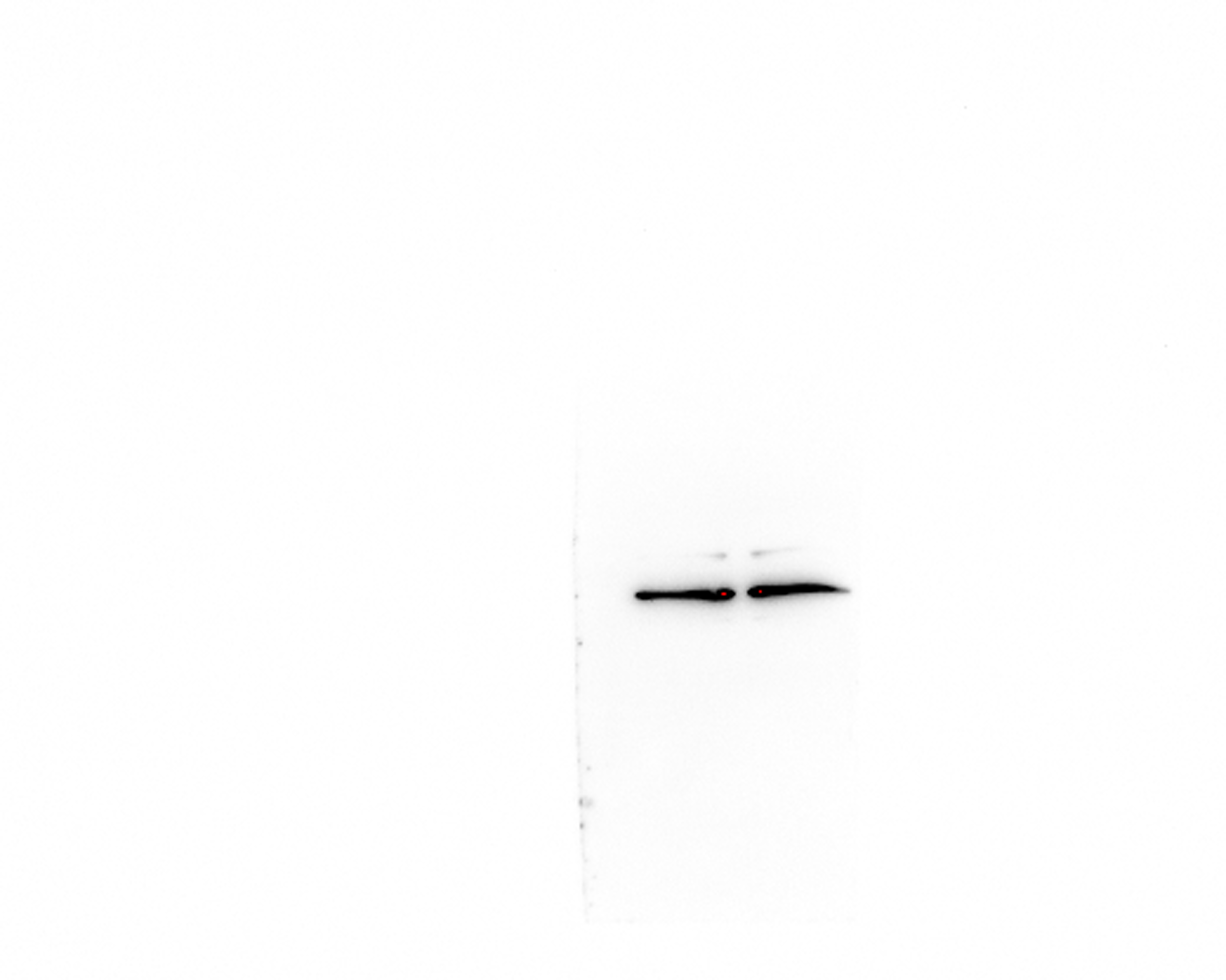

Supplement: Figure 2—figure supplement 1—source data 2. [file elife-108737-fig2-figsupp1-data2.zip › Figure 2—figure supplement 1—source data 2/α-actin-R1-blot.tif]

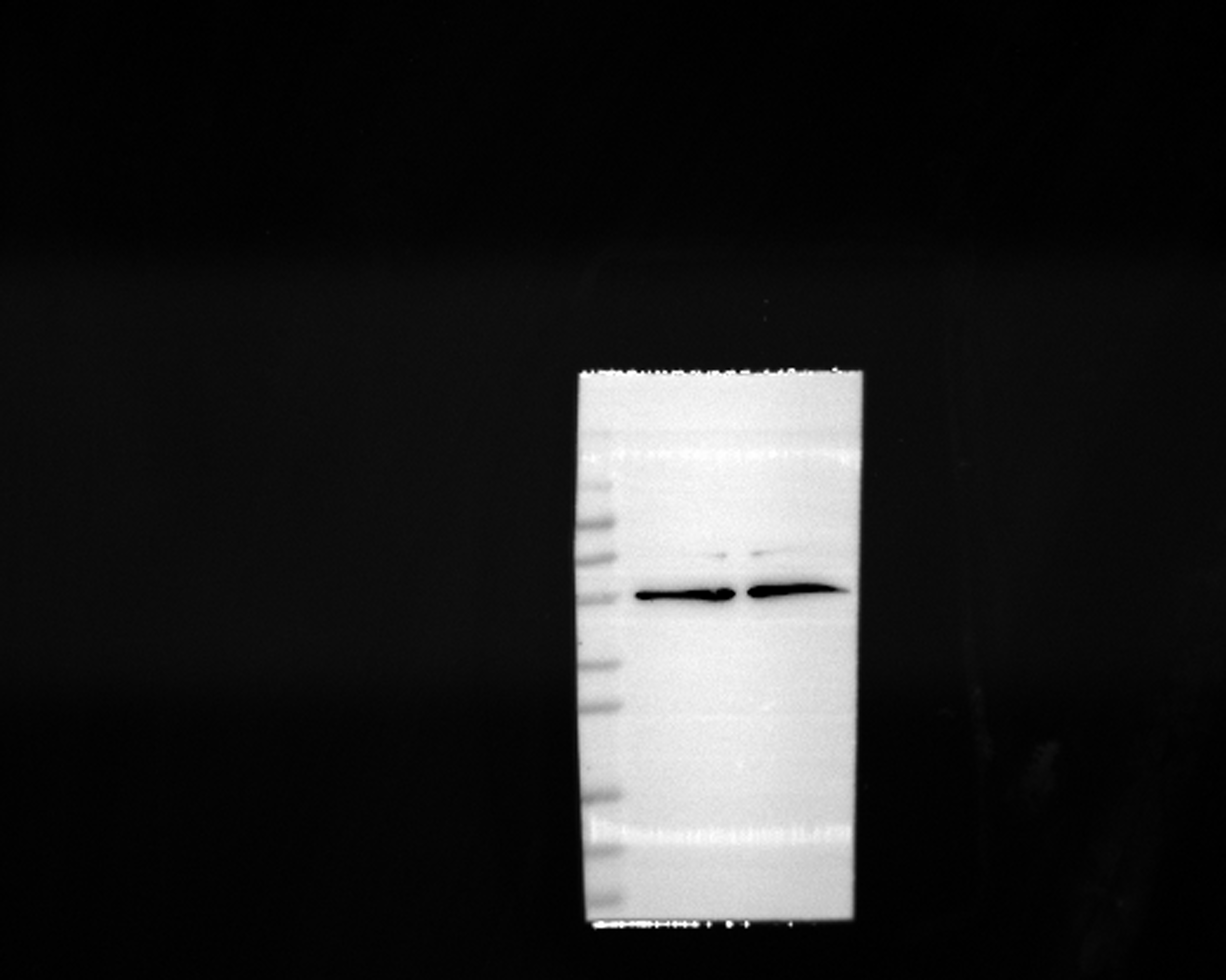

Supplement: Figure 2—figure supplement 1—source data 2. [file elife-108737-fig2-figsupp1-data2.zip › Figure 2—figure supplement 1—source data 2/α-actin-R1-marker.tif]

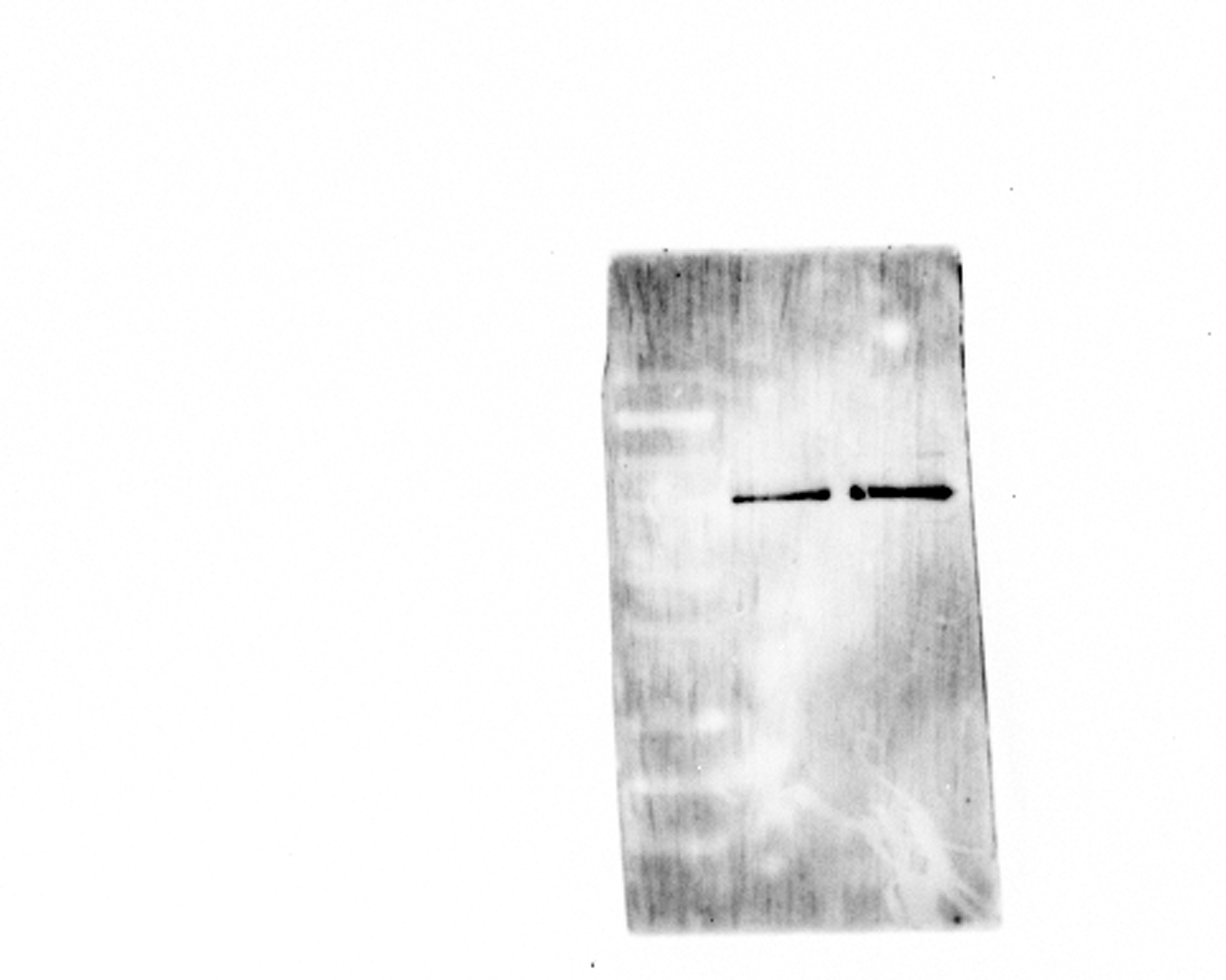

Supplement: Figure 2—figure supplement 1—source data 2. [file elife-108737-fig2-figsupp1-data2.zip › Figure 2—figure supplement 1—source data 2/α-actin-R2-blot.tif]

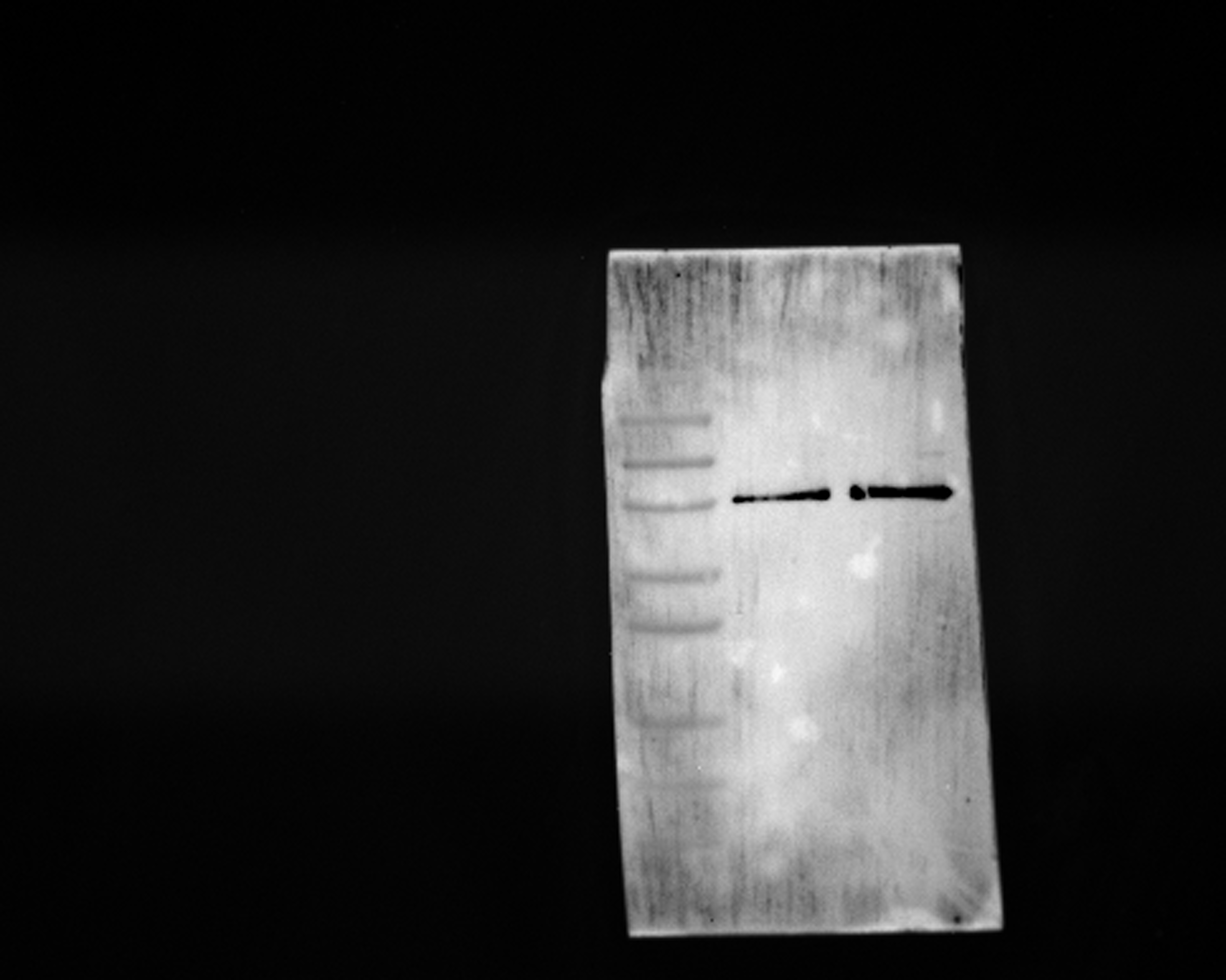

Supplement: Figure 2—figure supplement 1—source data 2. [file elife-108737-fig2-figsupp1-data2.zip › Figure 2—figure supplement 1—source data 2/α-actin-R2-marker.tif]

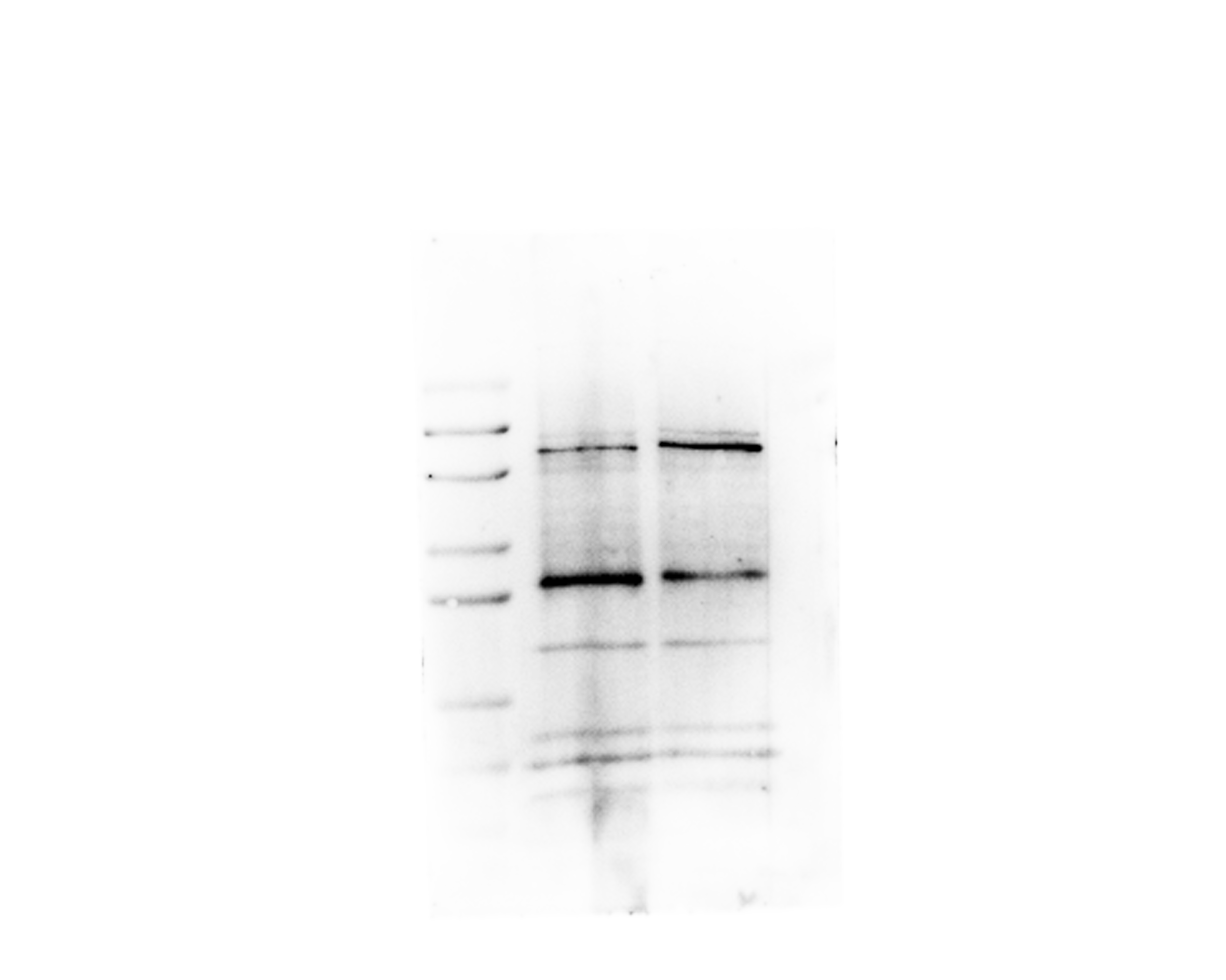

Supplement: Figure 2—figure supplement 1—source data 2. [file elife-108737-fig2-figsupp1-data2.zip › Figure 2—figure supplement 1—source data 2/α-BtRDP-R1-blot.tif]

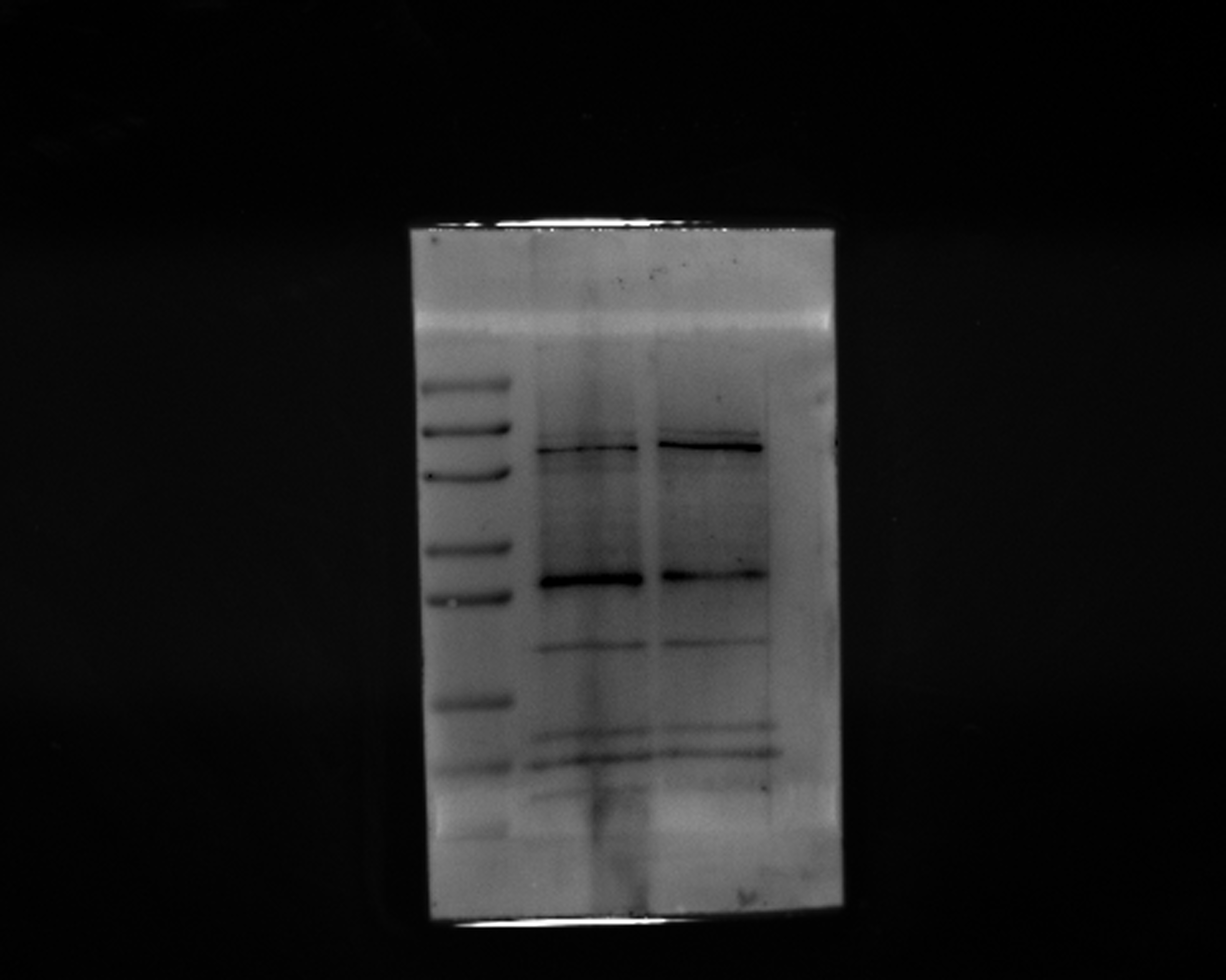

Supplement: Figure 2—figure supplement 1—source data 2. [file elife-108737-fig2-figsupp1-data2.zip › Figure 2—figure supplement 1—source data 2/α-BtRDP-R1-marker.tif]

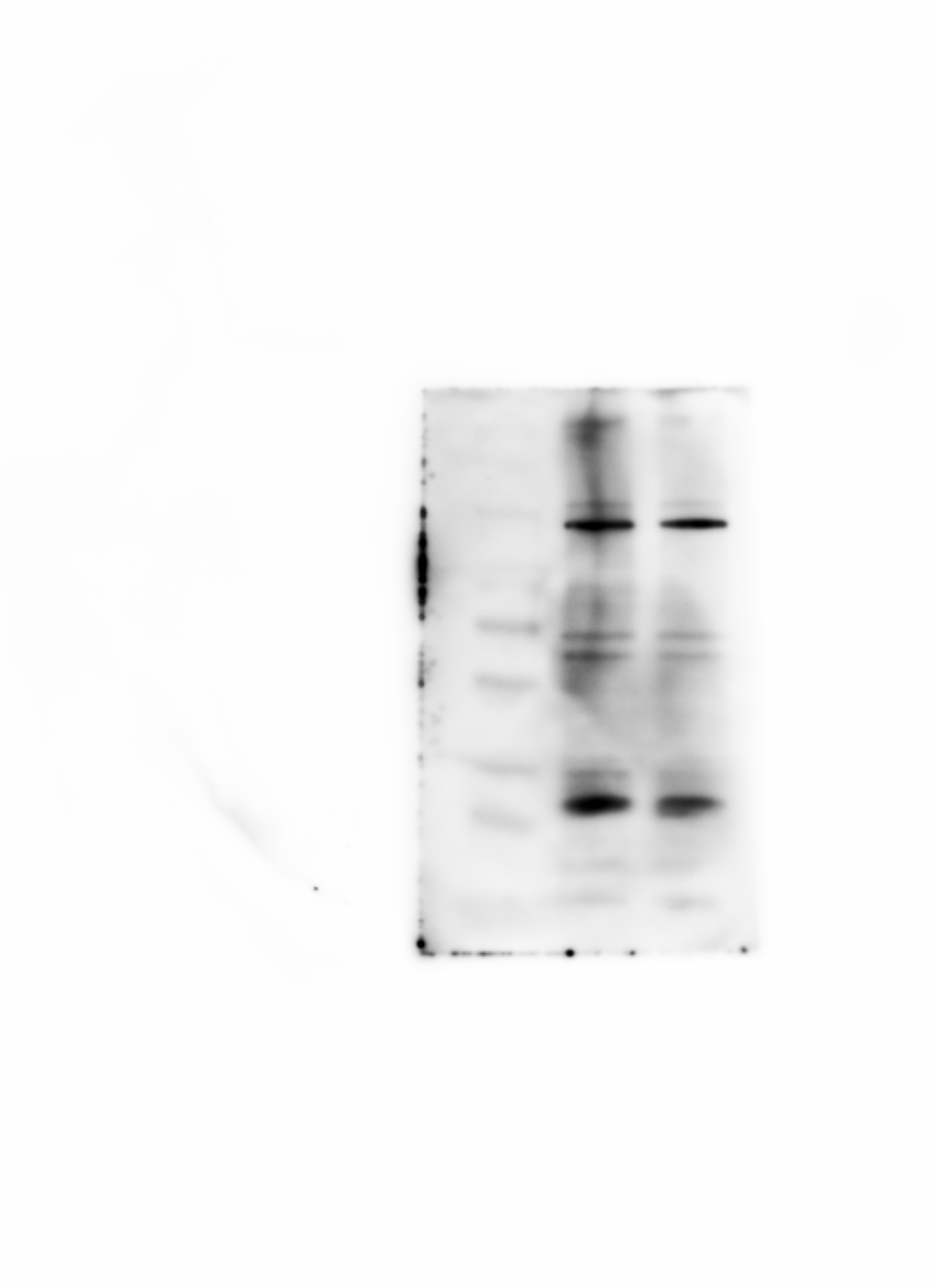

Supplement: Figure 2—figure supplement 1—source data 2. [file elife-108737-fig2-figsupp1-data2.zip › Figure 2—figure supplement 1—source data 2/α-BtRDP-R2-blot.tif]

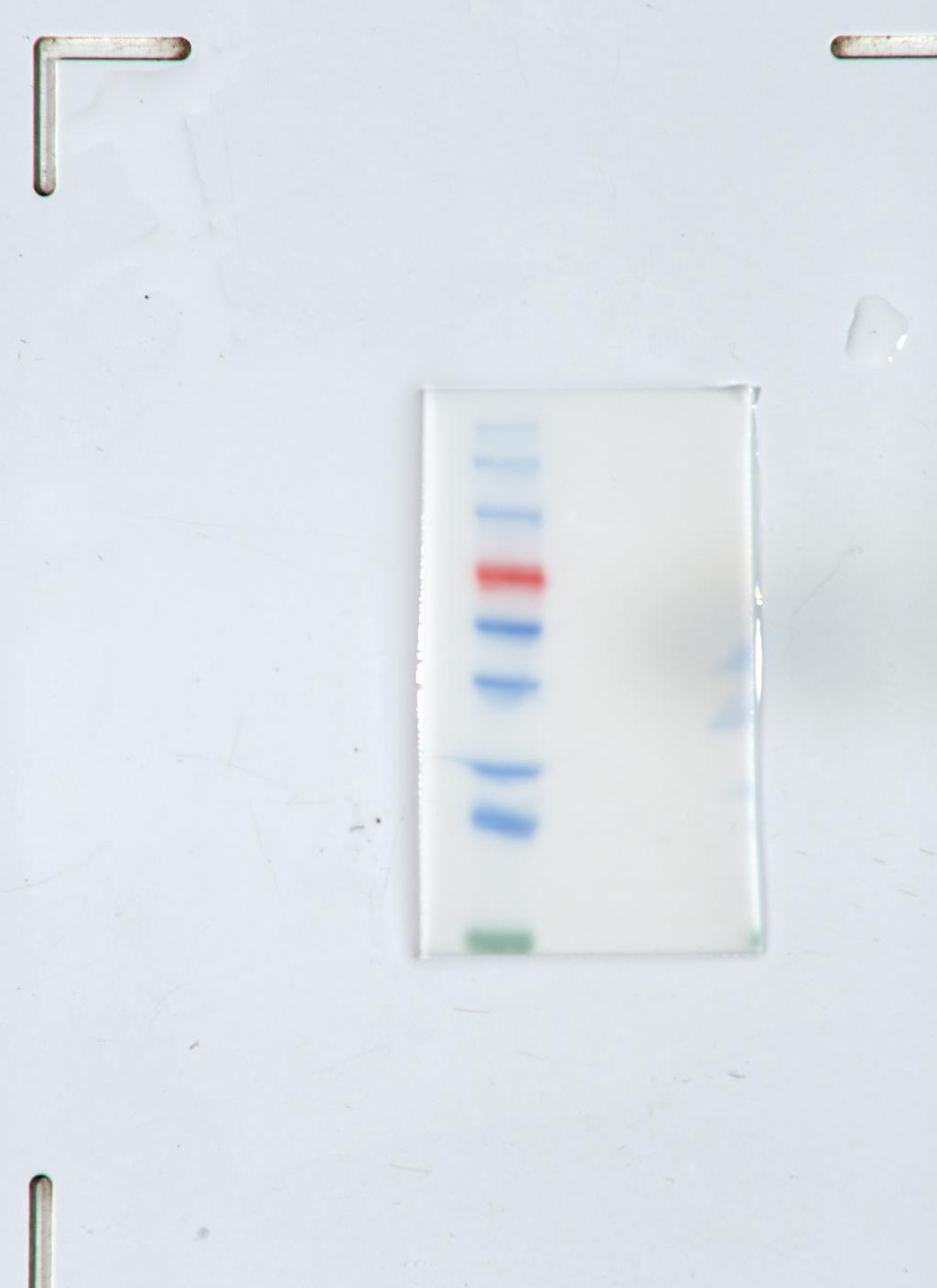

Supplement: Figure 2—figure supplement 1—source data 2. [file elife-108737-fig2-figsupp1-data2.zip › Figure 2—figure supplement 1—source data 2/α-BtRDP-R2-marker.jpg]

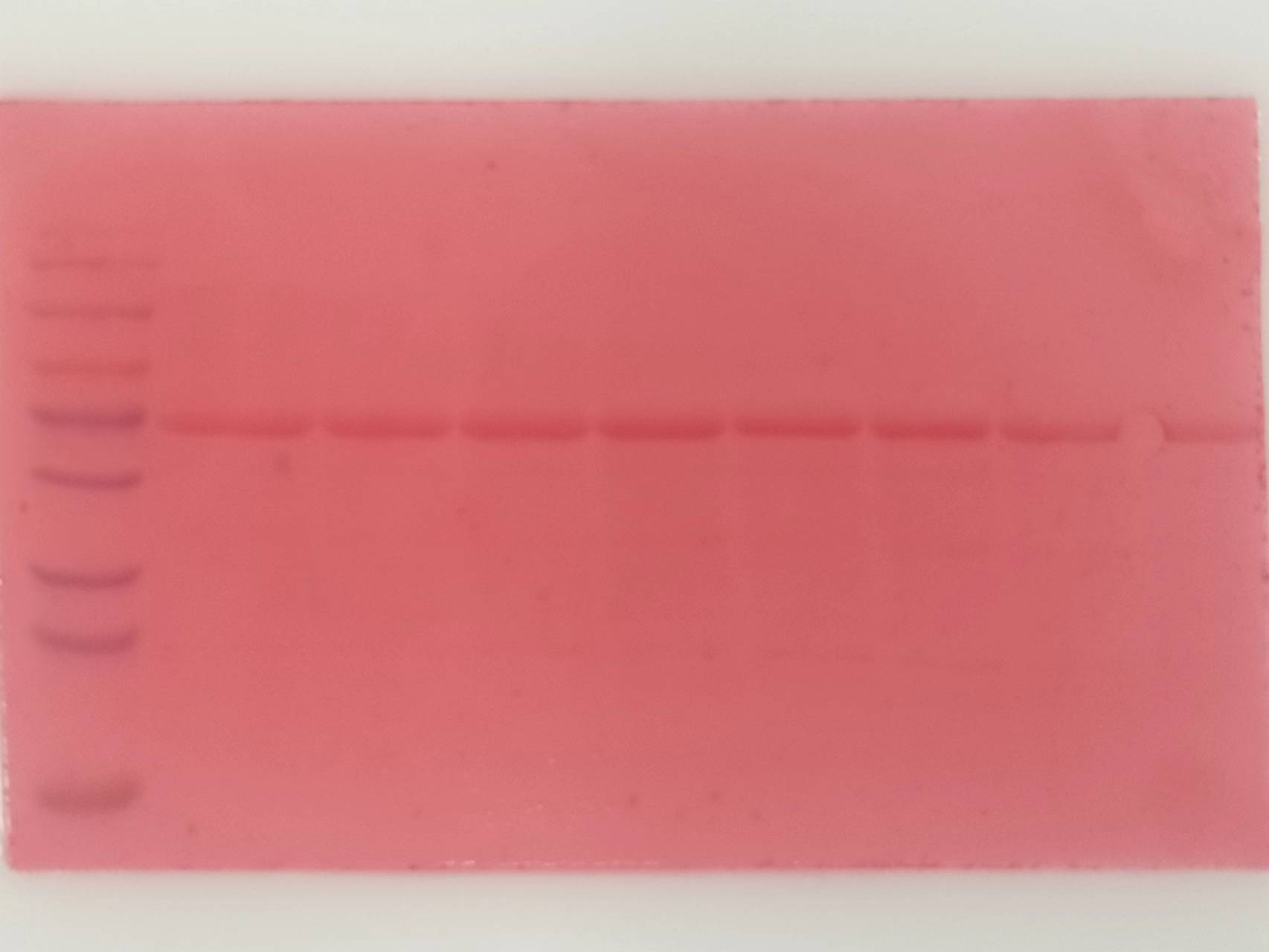

Supplement: Figure 3—source data 2. [file elife-108737-fig3-data2.zip › Figure 3—source data 2/RbCL.jpg]

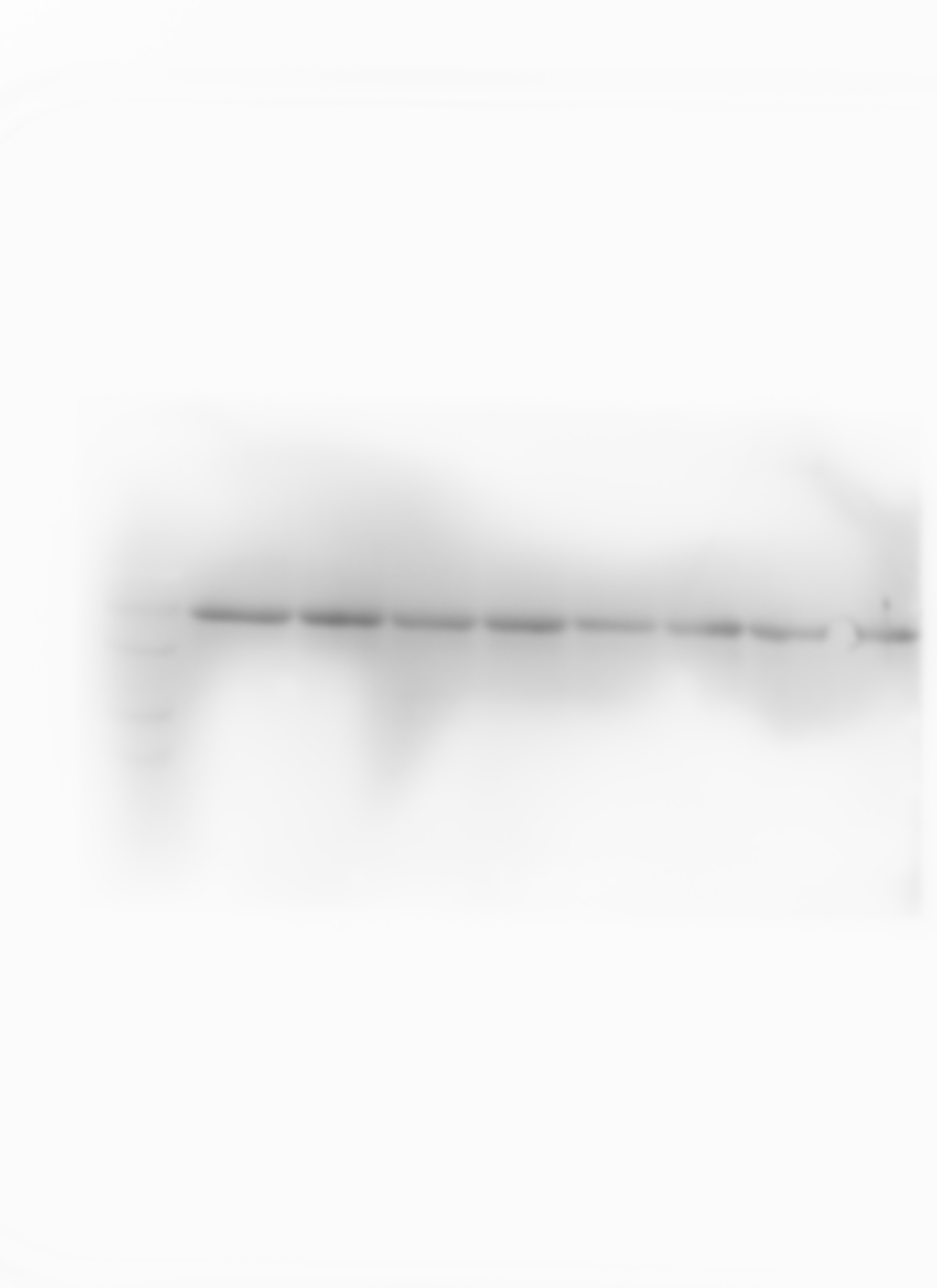

Supplement: Figure 3—source data 2. [file elife-108737-fig3-data2.zip › Figure 3—source data 2/α-NtRLP4-blot.tif]

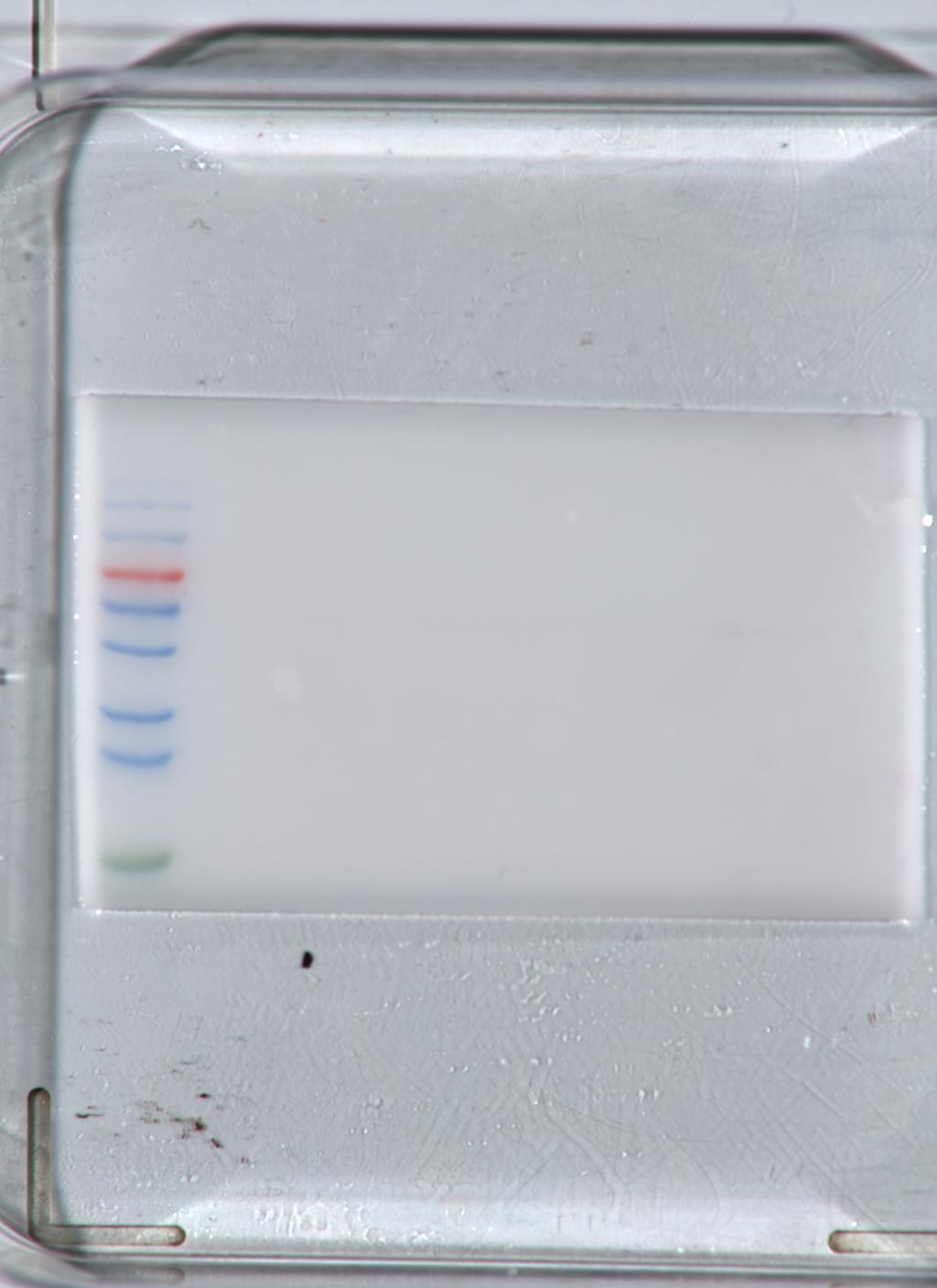

Supplement: Figure 3—source data 2. [file elife-108737-fig3-data2.zip › Figure 3—source data 2/α-NtRLP4-marker.jpg]

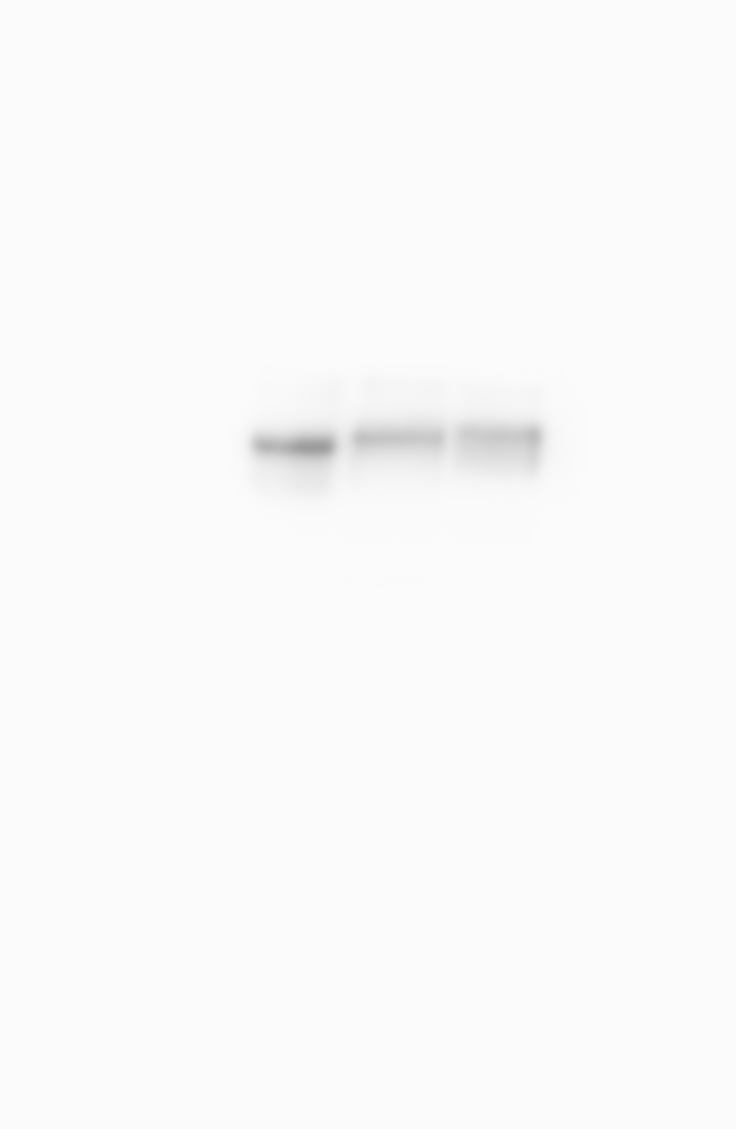

Supplement: Figure 3—source data 4. [file elife-108737-fig3-data4.zip › Figure 3—source data 4/input-flag-blot.tif]

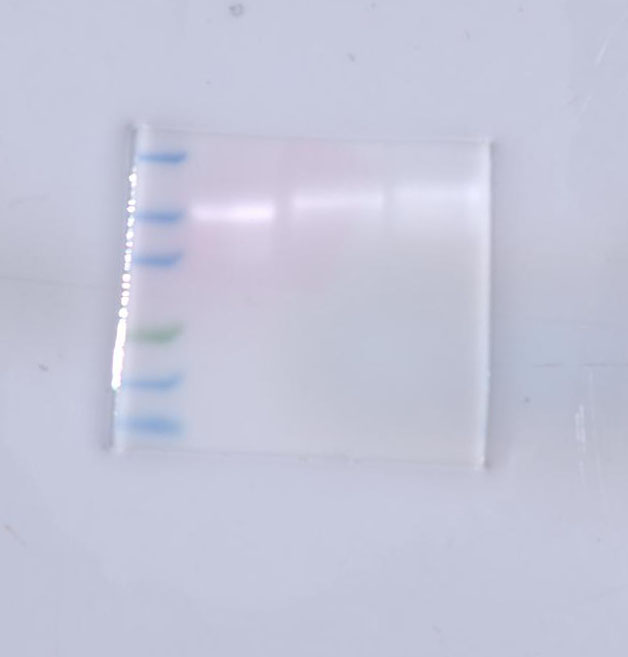

Supplement: Figure 3—source data 4. [file elife-108737-fig3-data4.zip › Figure 3—source data 4/input-flag-marker.jpg]

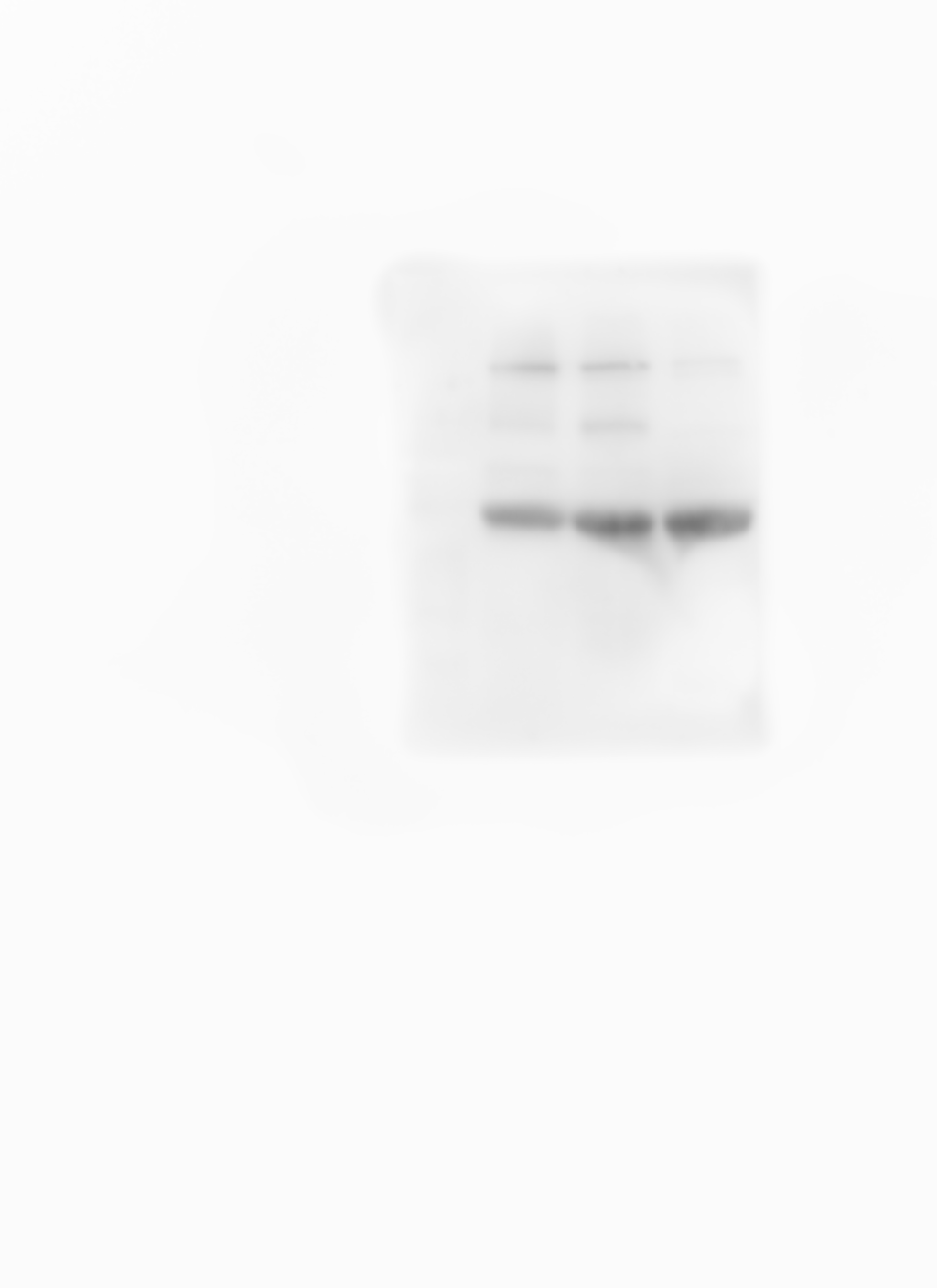

Supplement: Figure 3—source data 4. [file elife-108737-fig3-data4.zip › Figure 3—source data 4/input-myc-blot.tif]

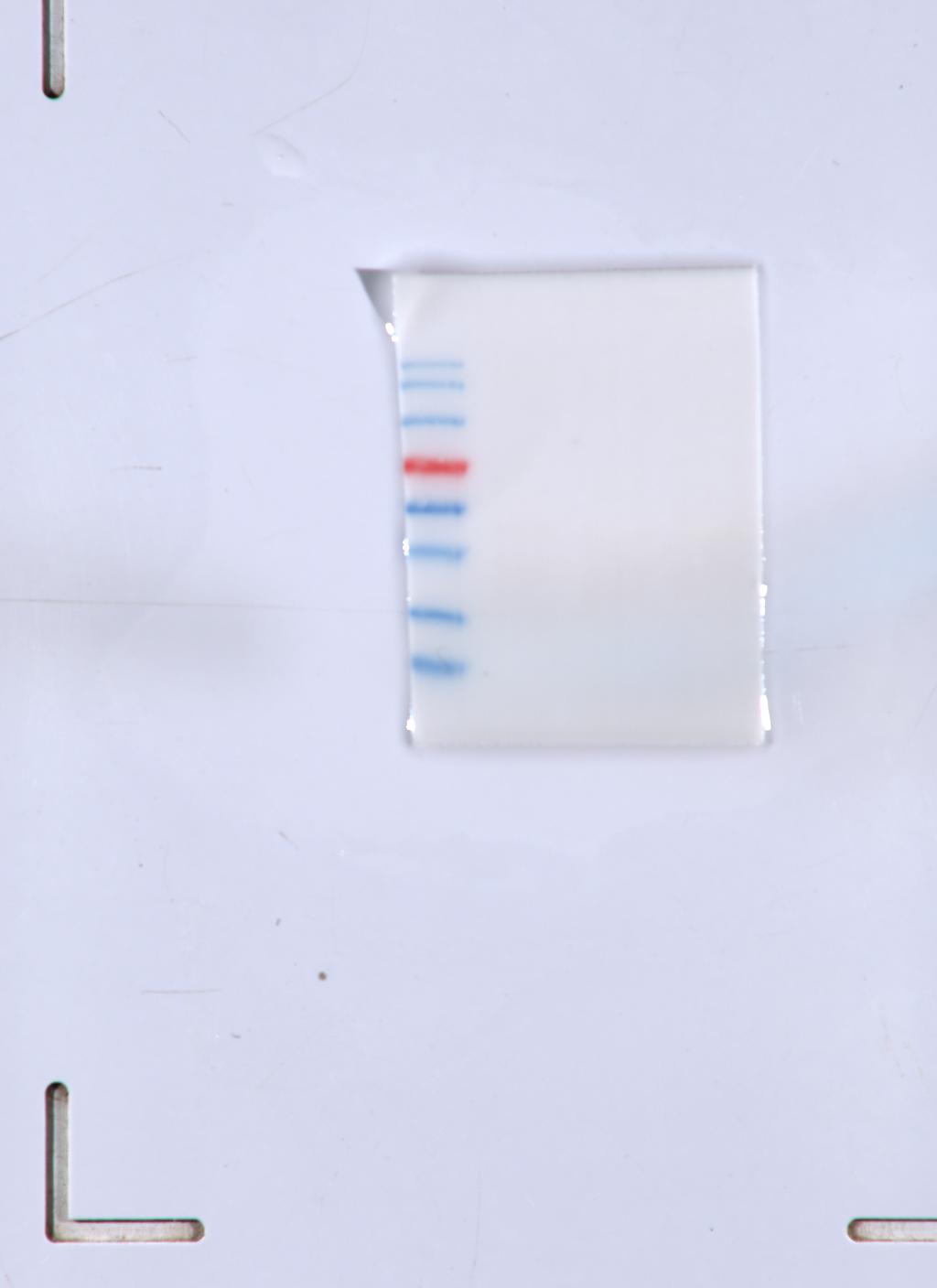

Supplement: Figure 3—source data 4. [file elife-108737-fig3-data4.zip › Figure 3—source data 4/input-myc-marker.jpg]

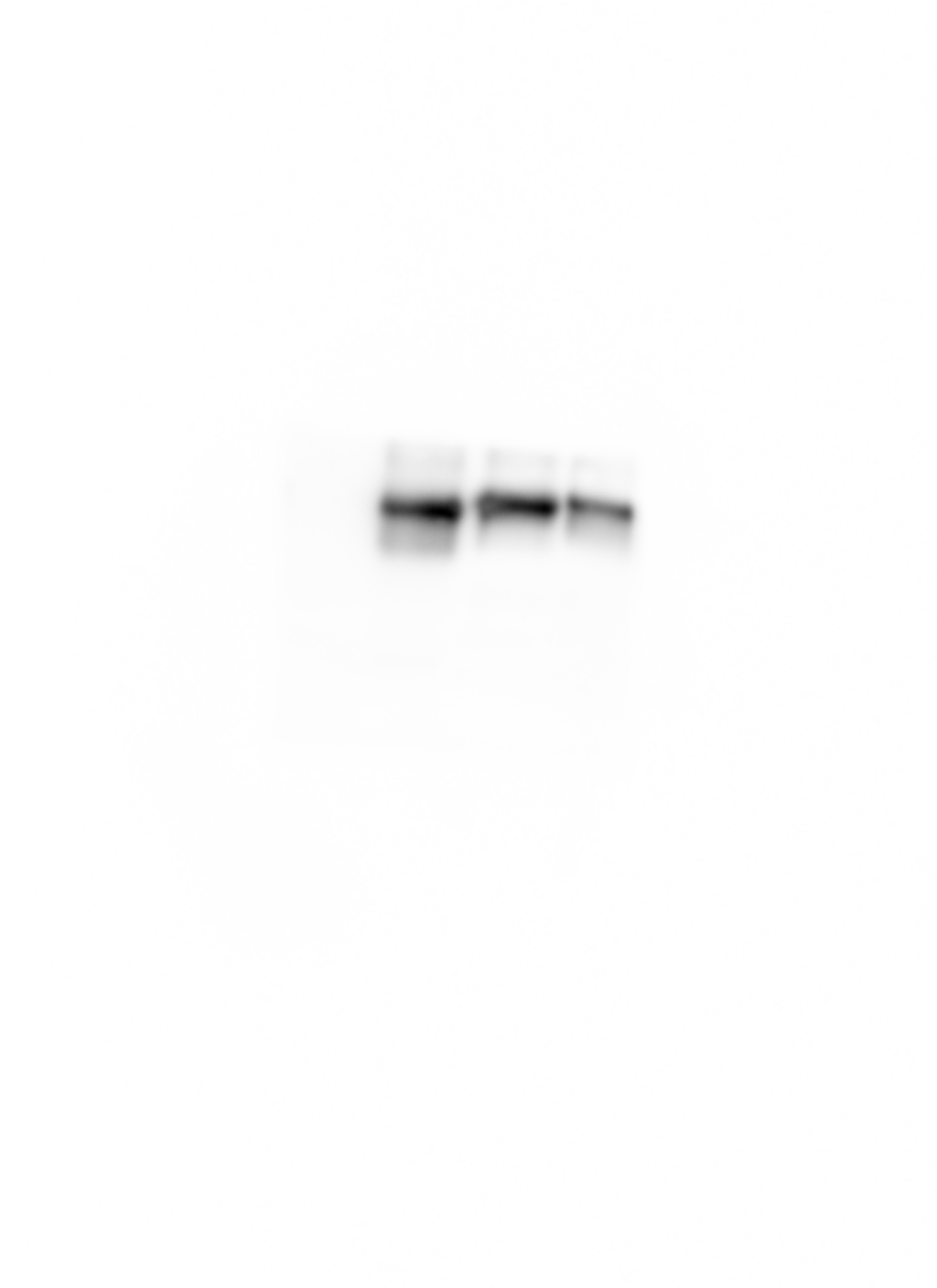

Supplement: Figure 3—source data 4. [file elife-108737-fig3-data4.zip › Figure 3—source data 4/IP-flag-blot.tif]

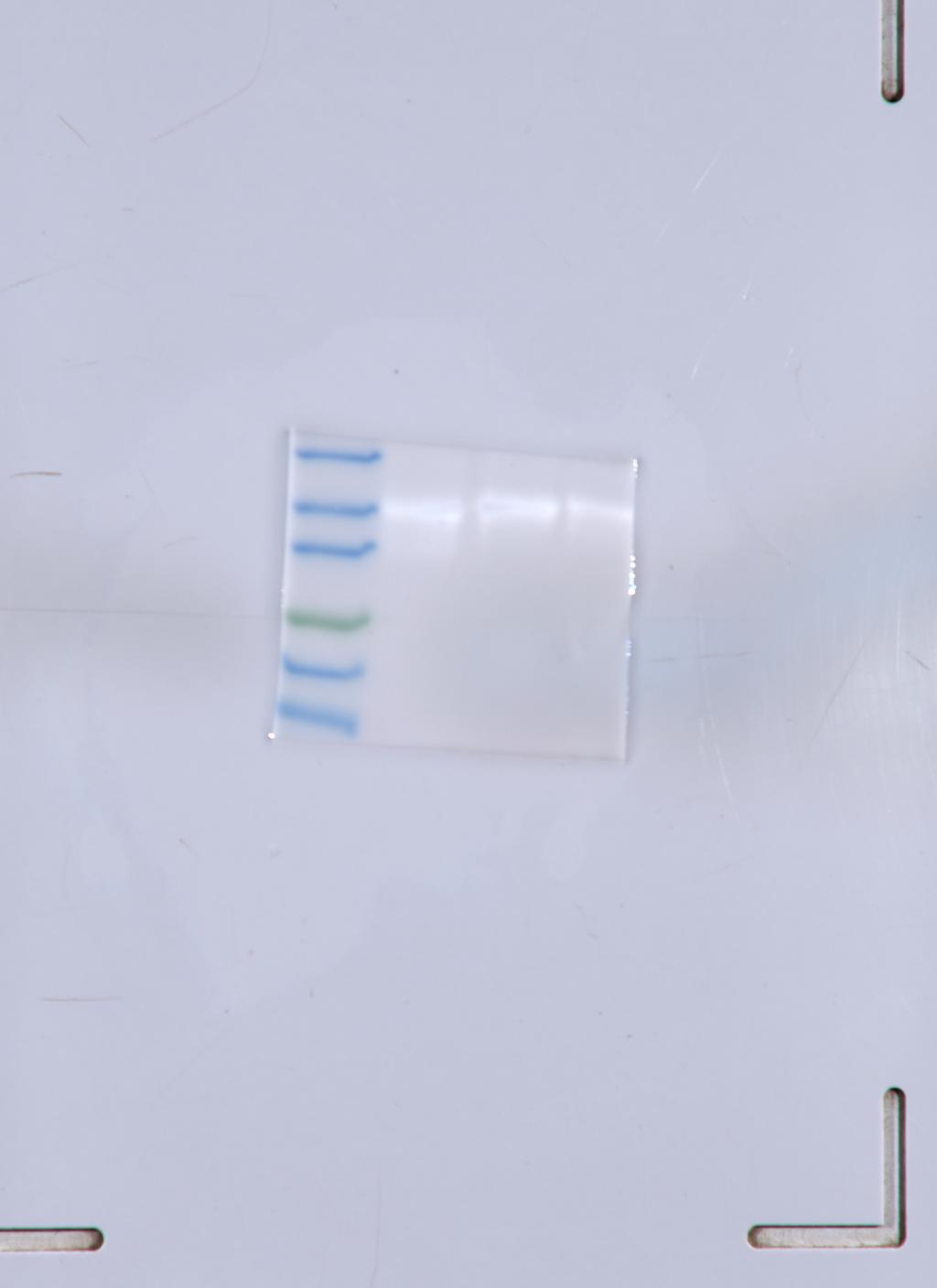

Supplement: Figure 3—source data 4. [file elife-108737-fig3-data4.zip › Figure 3—source data 4/IP-flag-marker.jpg]

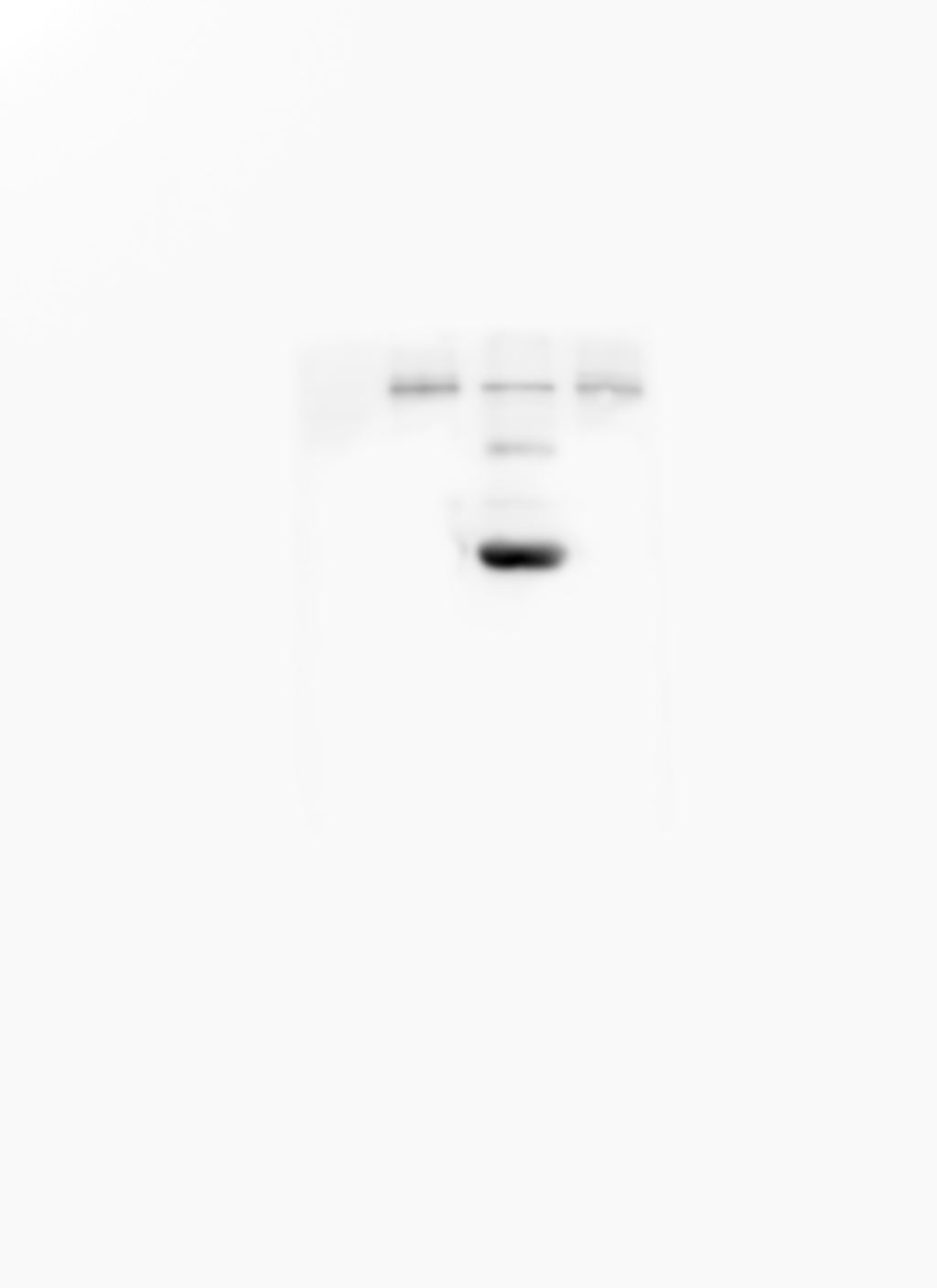

Supplement: Figure 3—source data 4. [file elife-108737-fig3-data4.zip › Figure 3—source data 4/IP-myc-blot.tif]

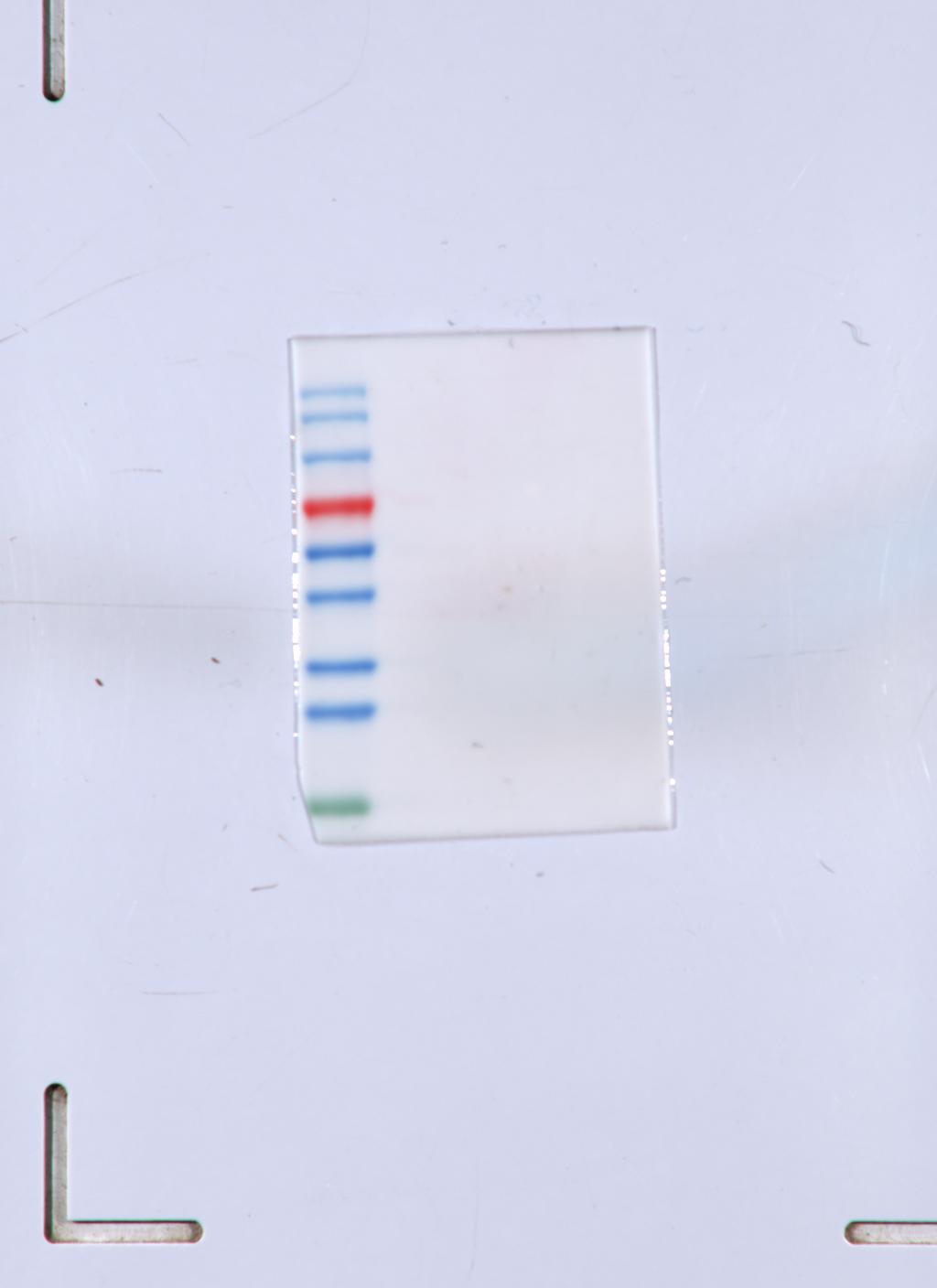

Supplement: Figure 3—source data 4. [file elife-108737-fig3-data4.zip › Figure 3—source data 4/IP-myc-marker.jpg]

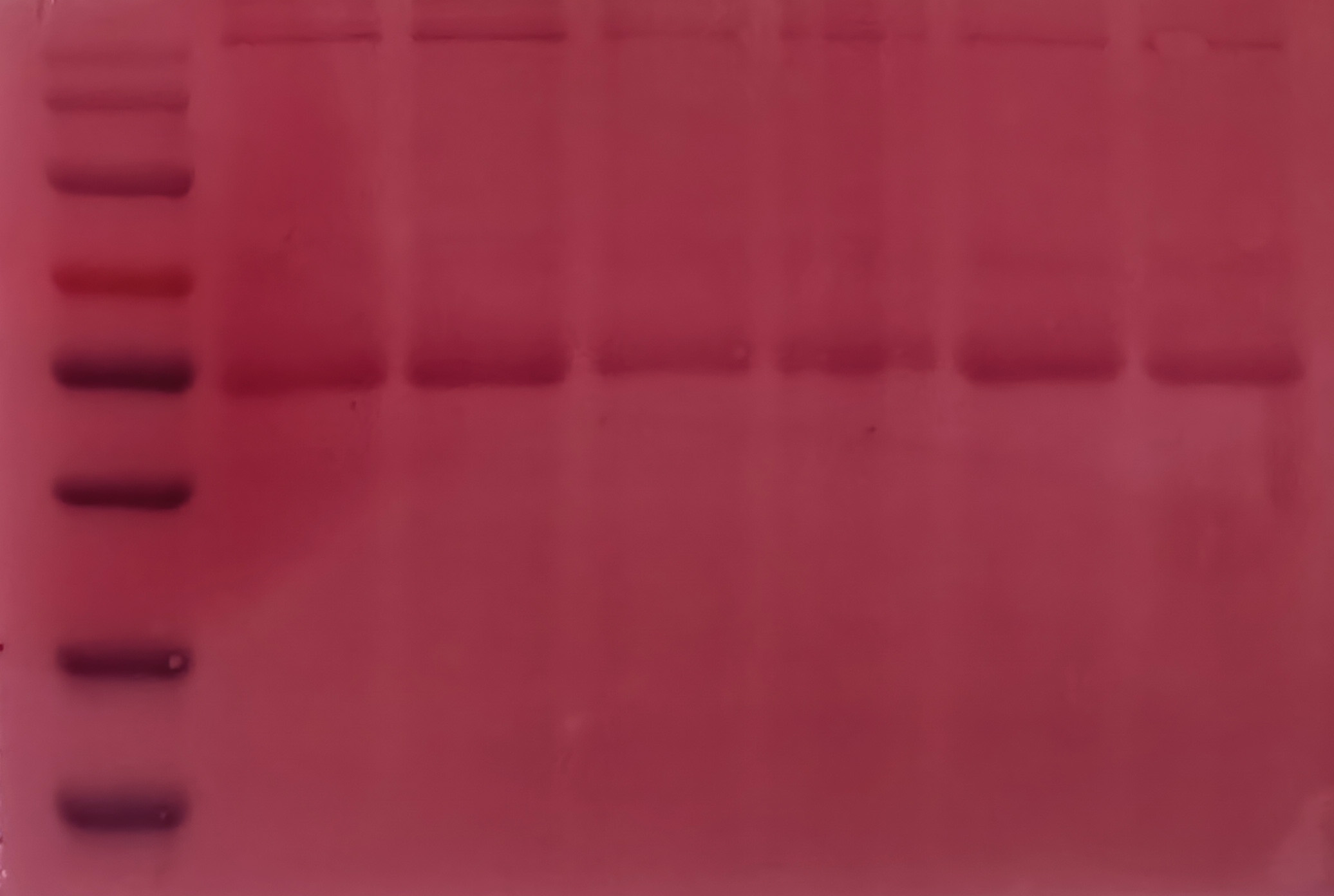

Supplement: Figure 3—source data 6. [file elife-108737-fig3-data6.zip › Figure 3—source data 6/RbCL.jpg]

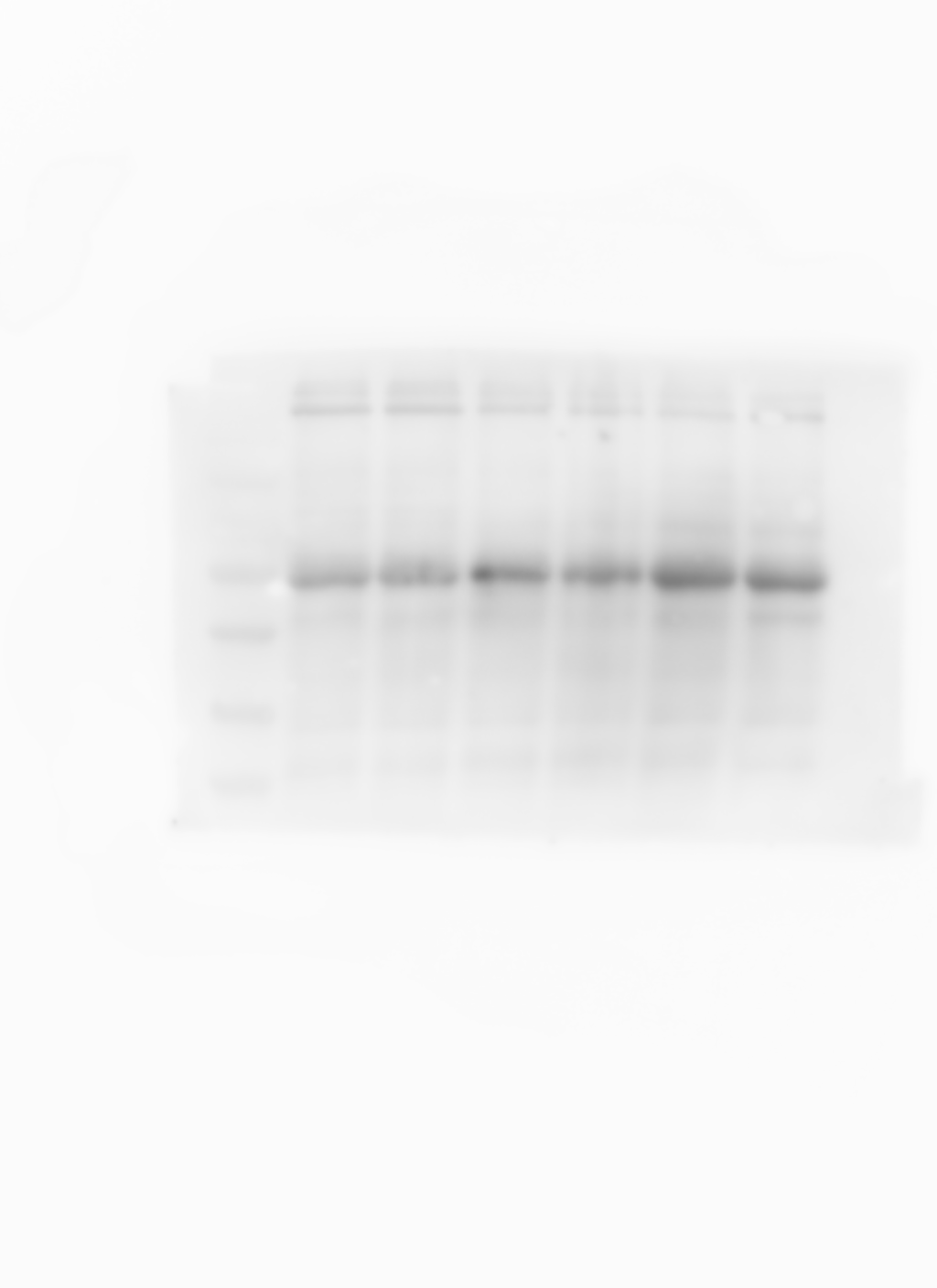

Supplement: Figure 3—source data 6. [file elife-108737-fig3-data6.zip › Figure 3—source data 6/α-NtRLP4-blot.tif]

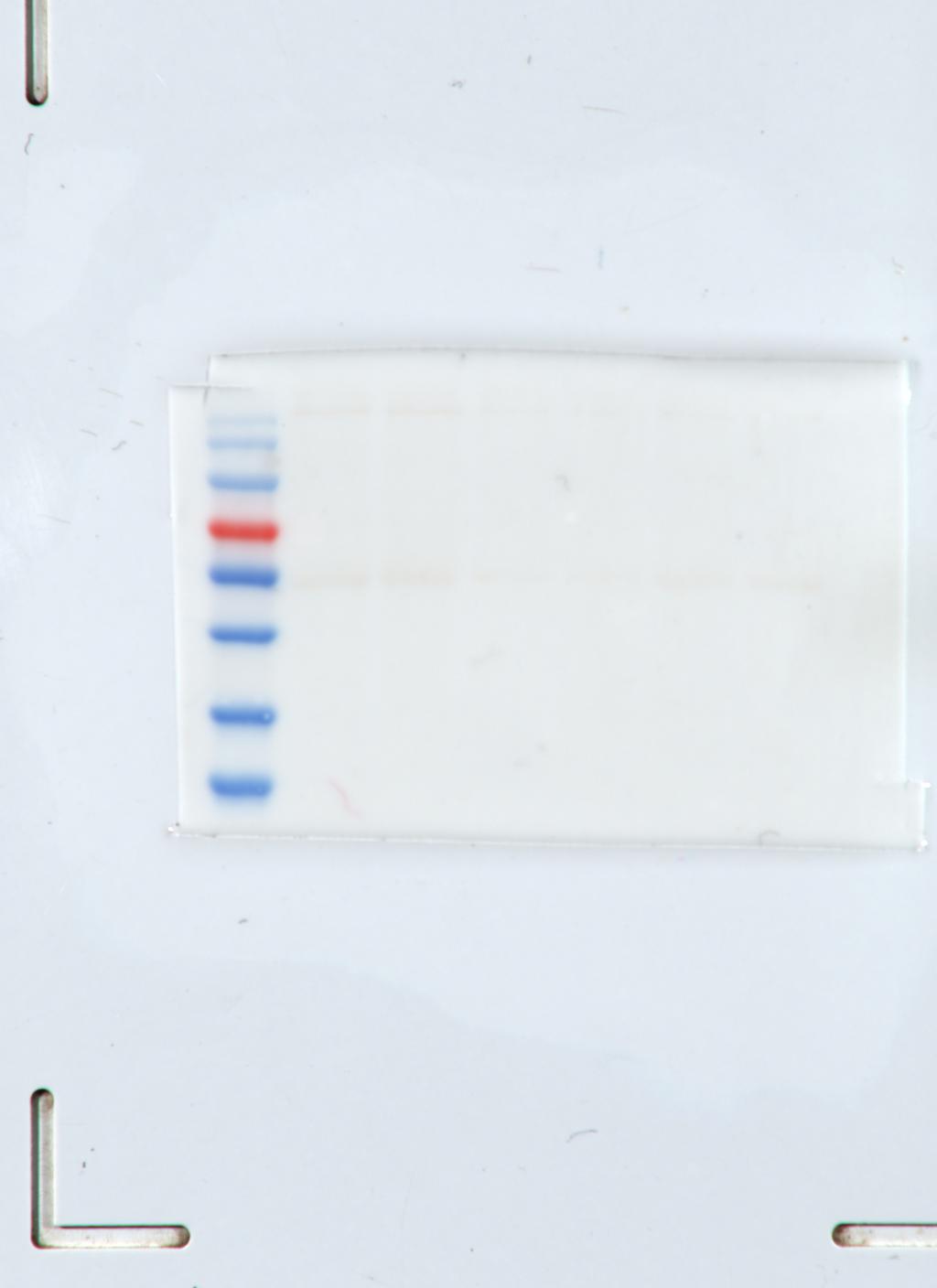

Supplement: Figure 3—source data 6. [file elife-108737-fig3-data6.zip › Figure 3—source data 6/α-NtRLP4-marker.jpg]

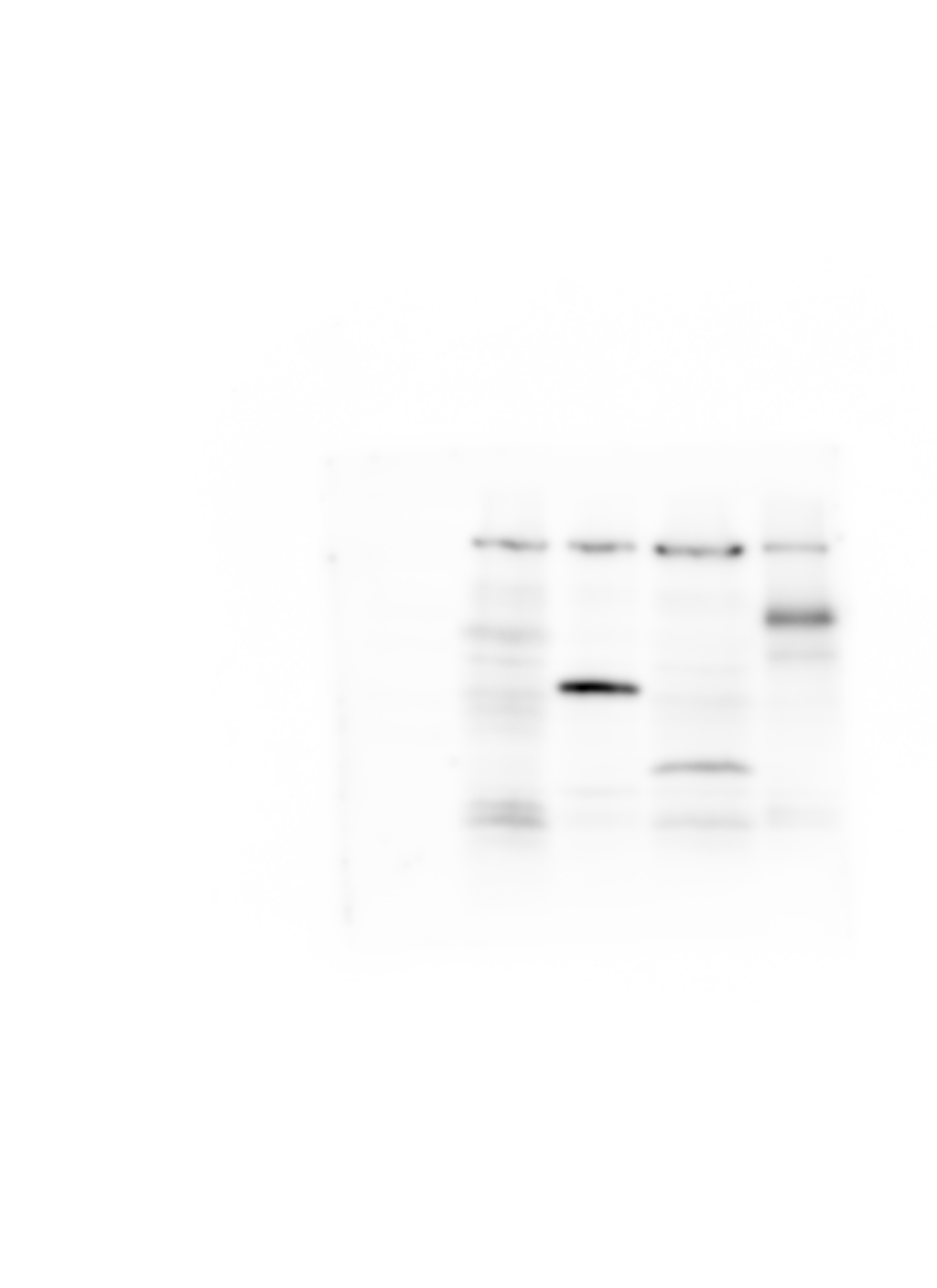

Supplement: Figure 3—figure supplement 3—source data 2. [file elife-108737-fig3-figsupp3-data2.zip › Figure 3—figure supplement 3—source data 2/α-myc-blot.tif]

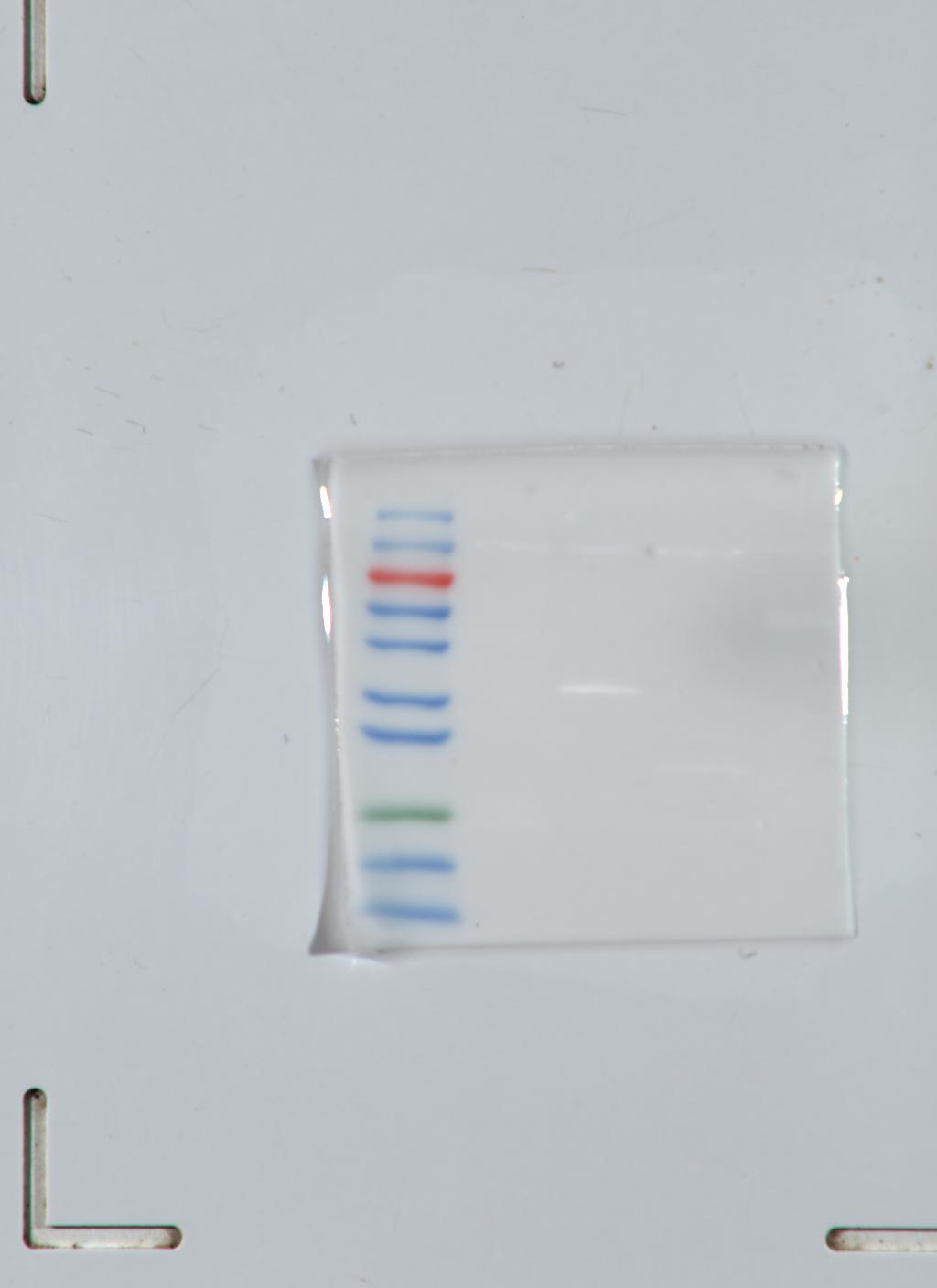

Supplement: Figure 3—figure supplement 3—source data 2. [file elife-108737-fig3-figsupp3-data2.zip › Figure 3—figure supplement 3—source data 2/α-myc-marker.jpg]

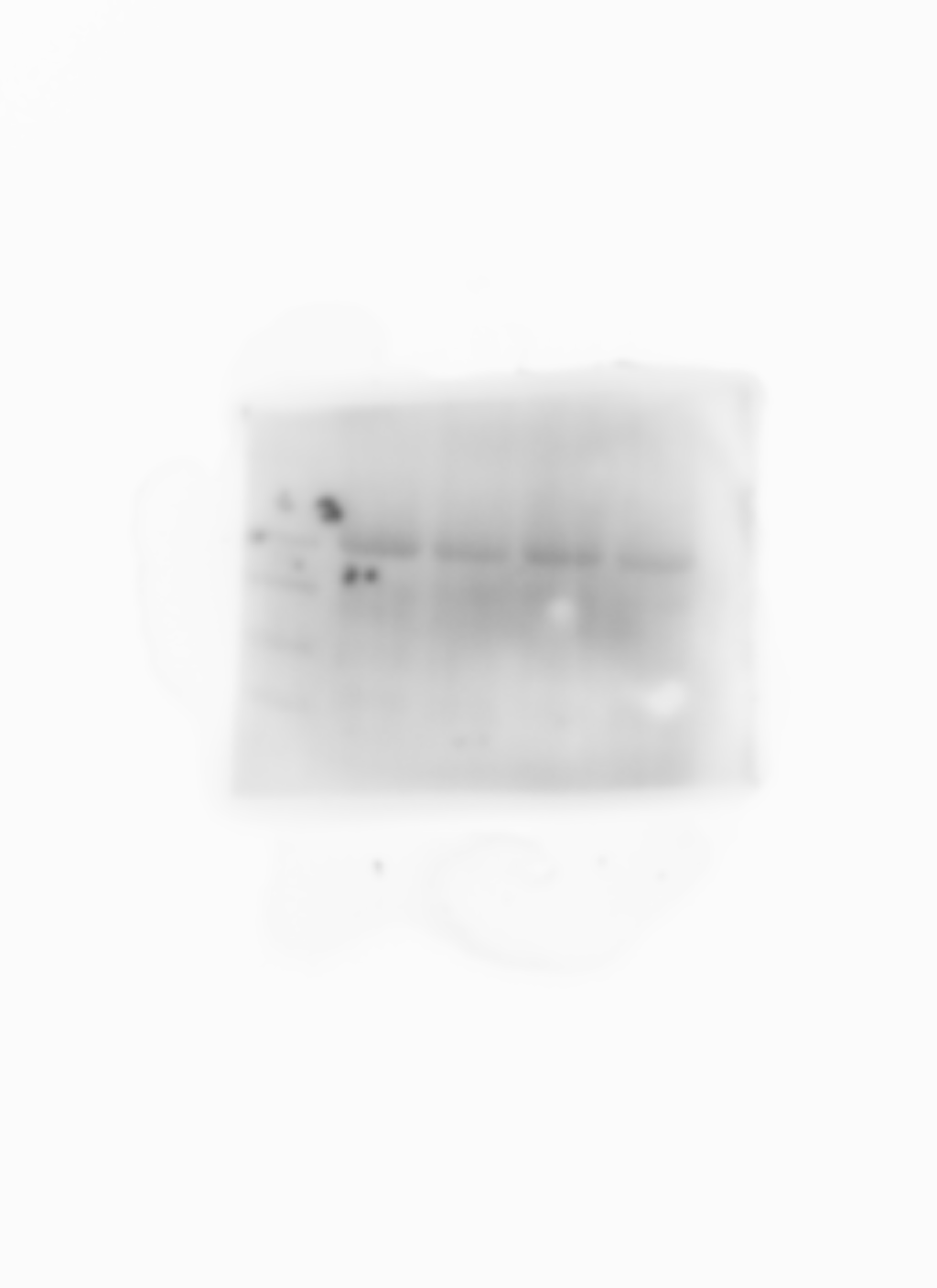

Supplement: Figure 3—figure supplement 3—source data 4. [file elife-108737-fig3-figsupp3-data4.zip › Figure 3—figure supplement 3—source data 4/input-NtRLP4-blot.tif]

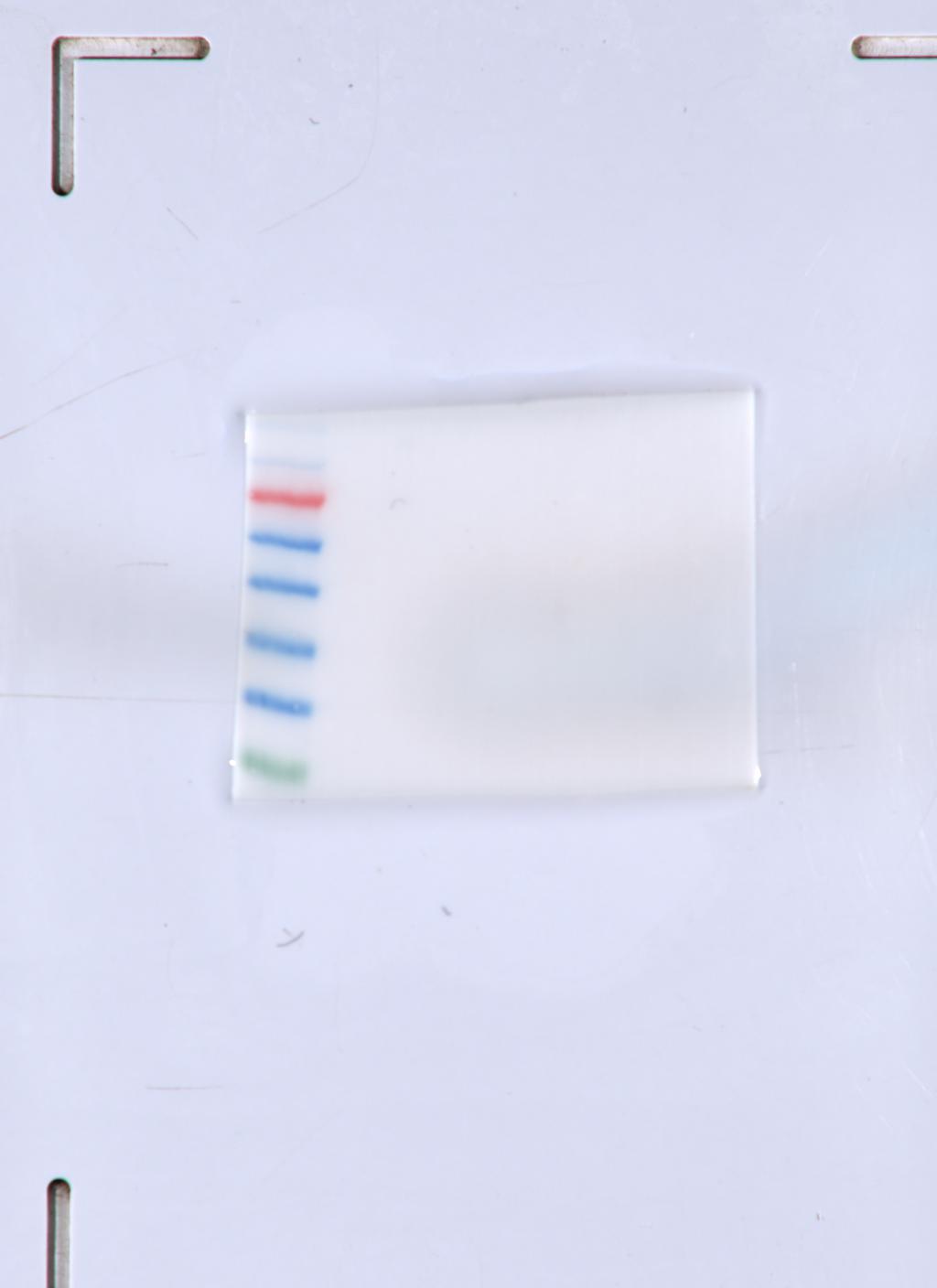

Supplement: Figure 3—figure supplement 3—source data 4. [file elife-108737-fig3-figsupp3-data4.zip › Figure 3—figure supplement 3—source data 4/input-NtRLP4-marker.jpg]

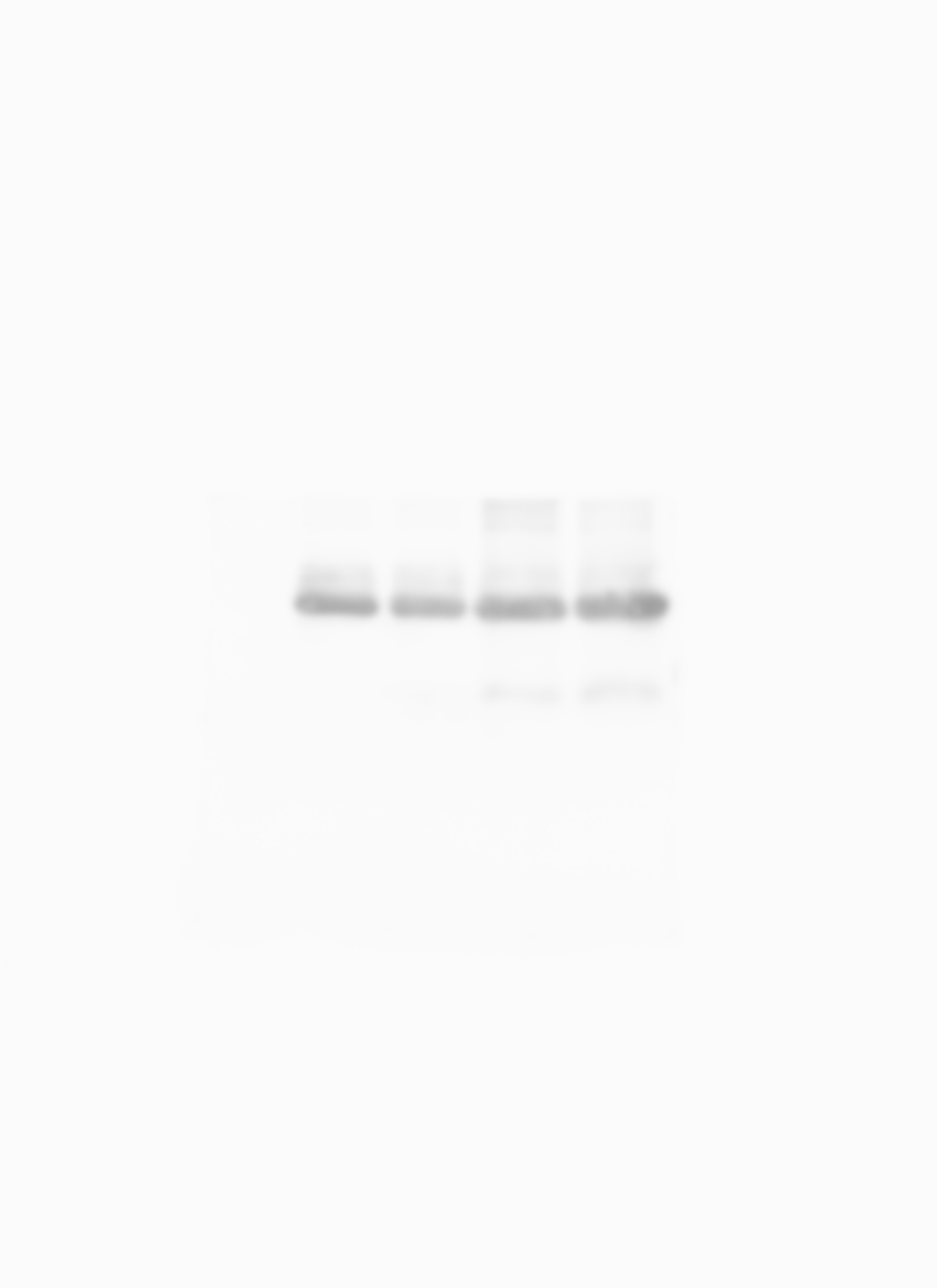

Supplement: Figure 3—figure supplement 3—source data 4. [file elife-108737-fig3-figsupp3-data4.zip › Figure 3—figure supplement 3—source data 4/IP-flag-blot.tif]

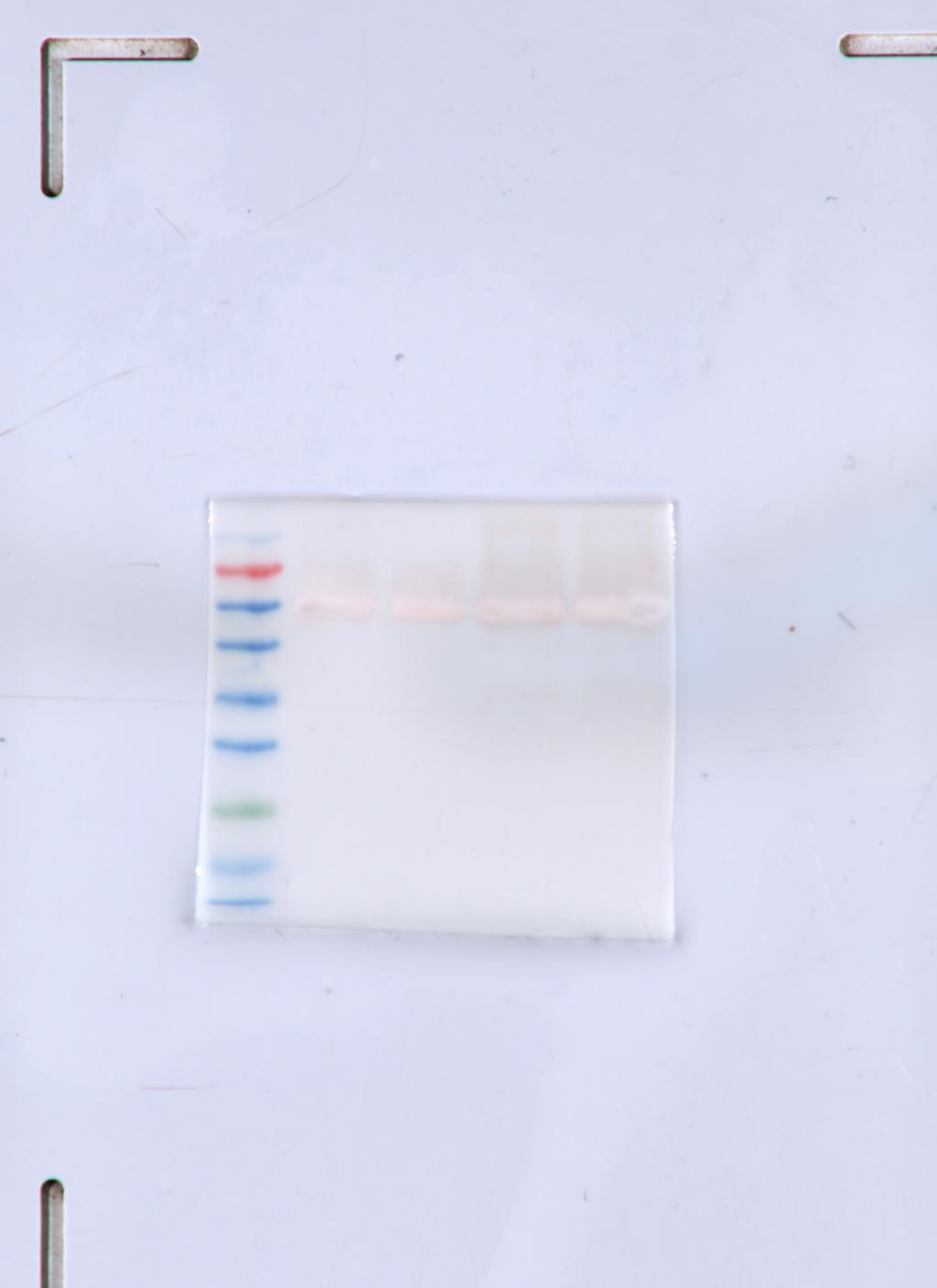

Supplement: Figure 3—figure supplement 3—source data 4. [file elife-108737-fig3-figsupp3-data4.zip › Figure 3—figure supplement 3—source data 4/ip-flag-marker.jpg]

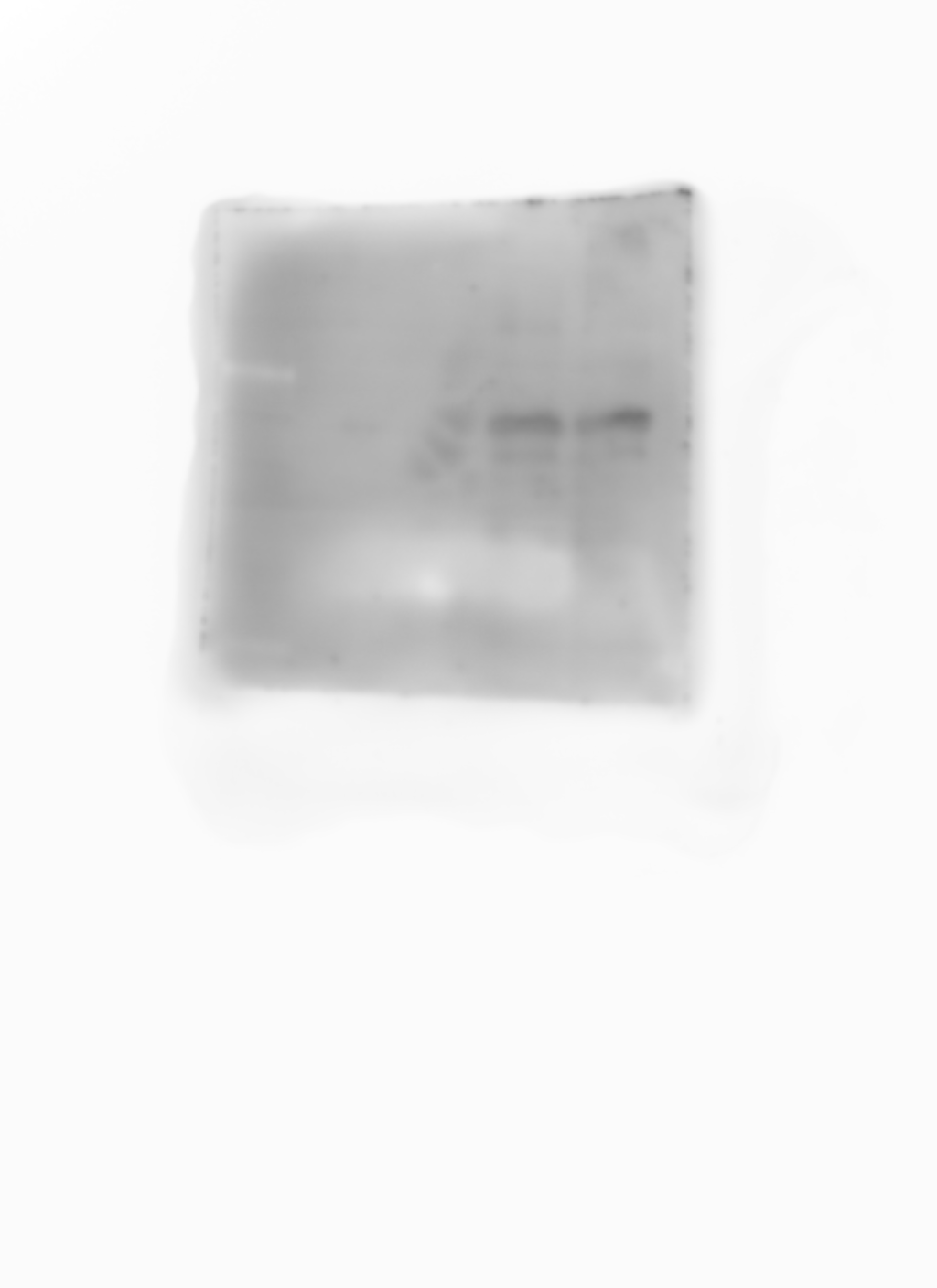

Supplement: Figure 3—figure supplement 3—source data 4. [file elife-108737-fig3-figsupp3-data4.zip › Figure 3—figure supplement 3—source data 4/IP-NtRLP4-blot.tif]

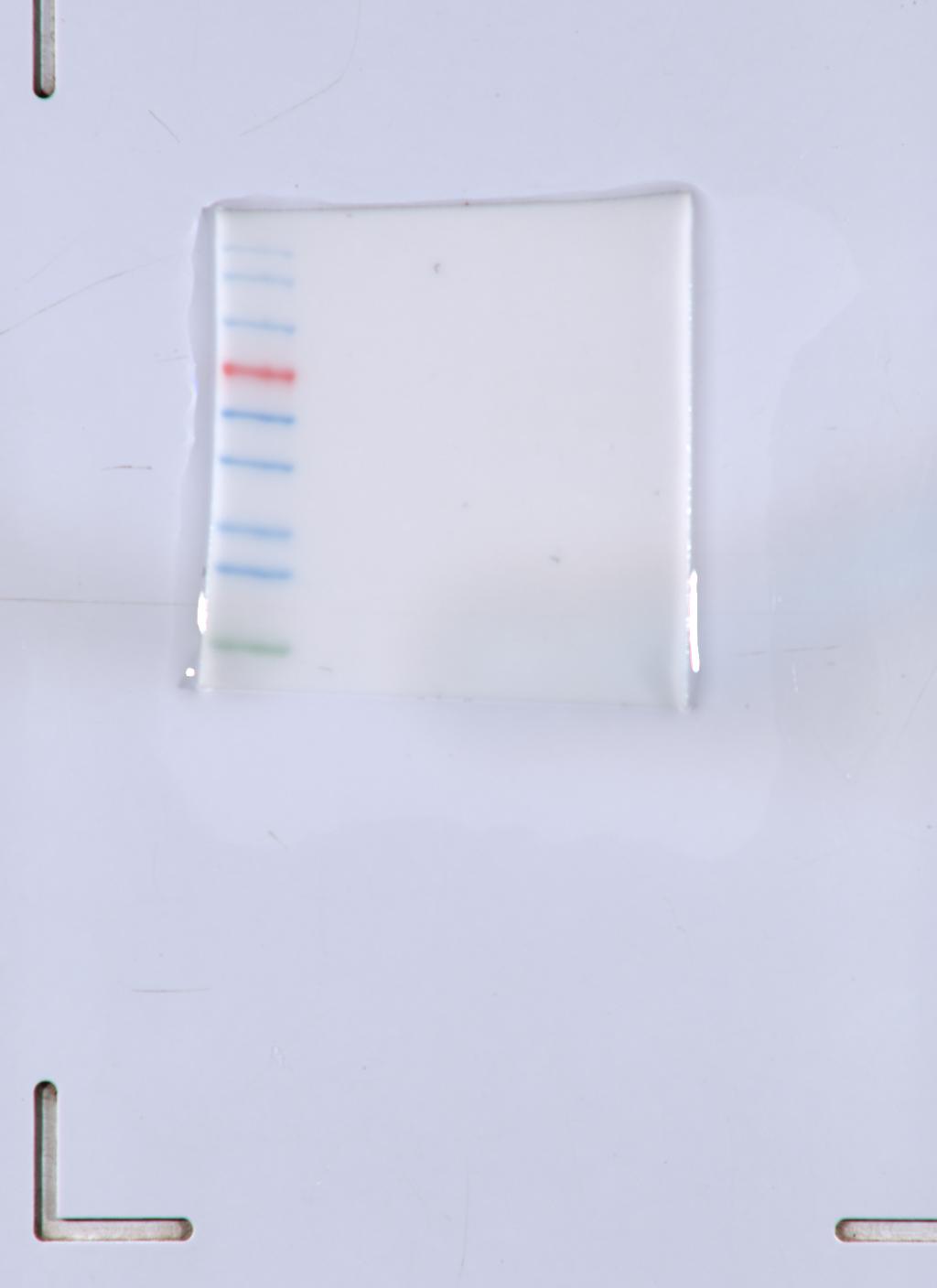

Supplement: Figure 3—figure supplement 3—source data 4. [file elife-108737-fig3-figsupp3-data4.zip › Figure 3—figure supplement 3—source data 4/IP-NtRLP4-marker.jpg]

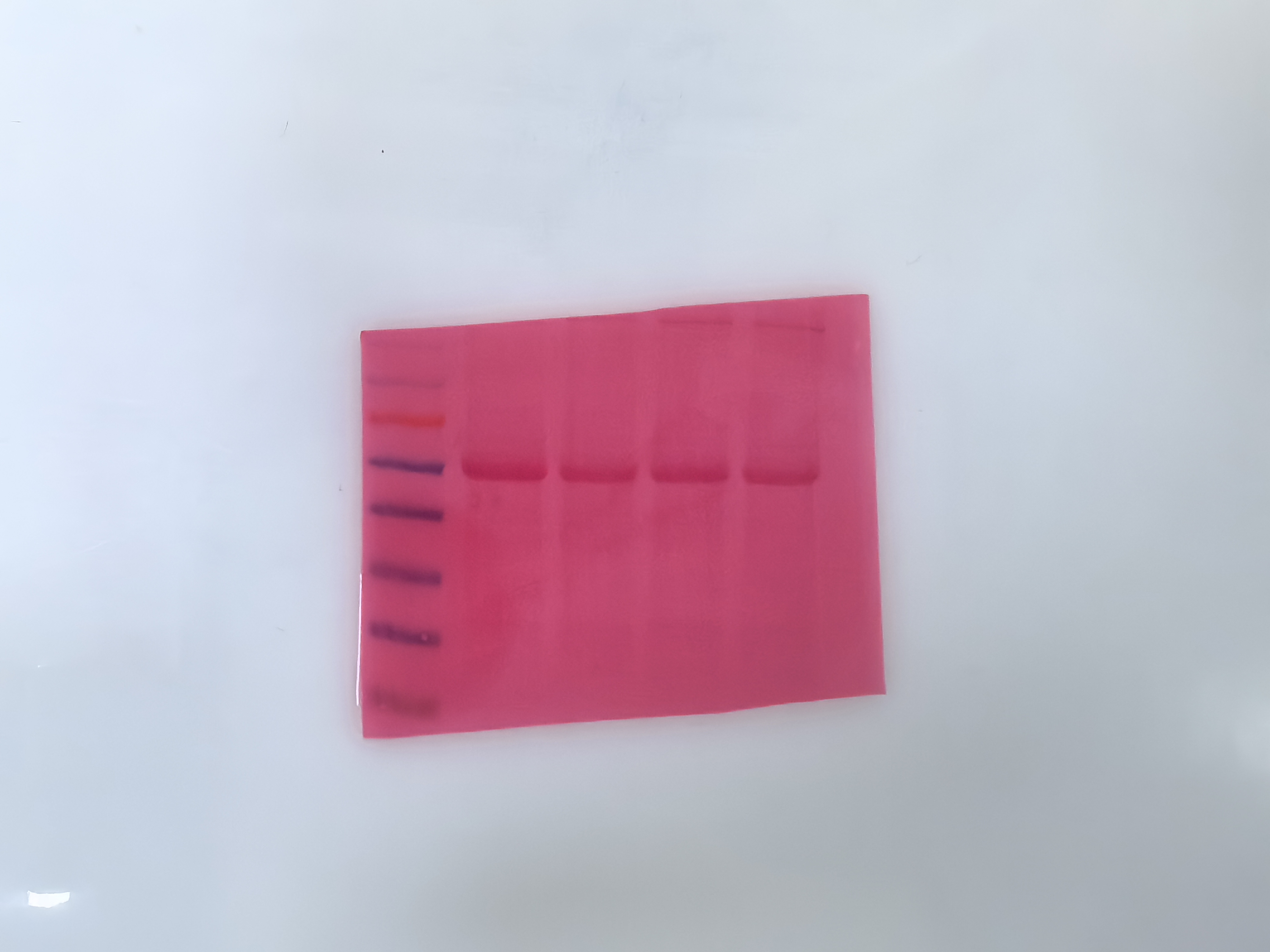

Supplement: Figure 3—figure supplement 3—source data 4. [file elife-108737-fig3-figsupp3-data4.zip › Figure 3—figure supplement 3—source data 4/RbcL.jpg]

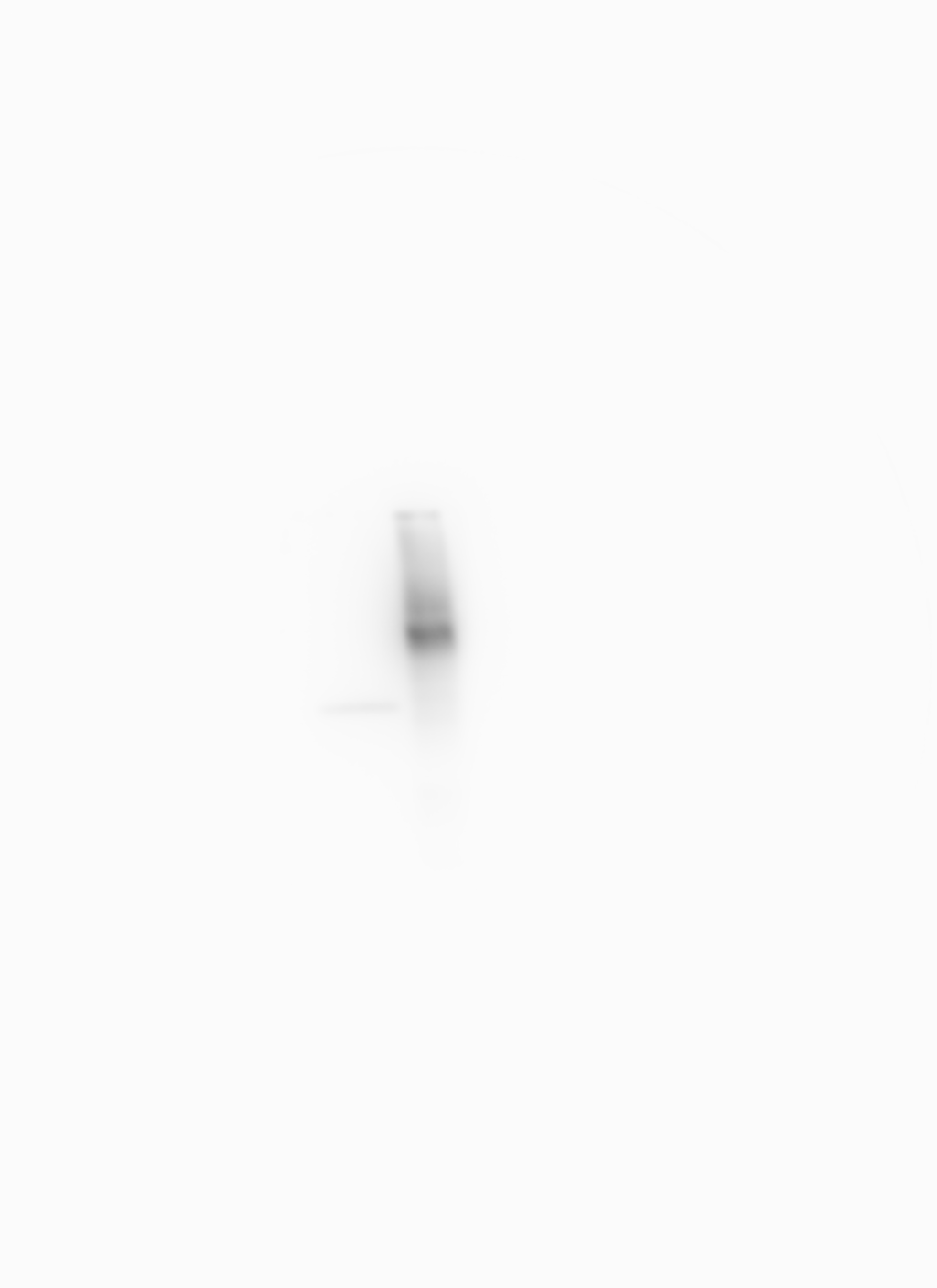

Supplement: Figure 3—figure supplement 4—source data 2. [file elife-108737-fig3-figsupp4-data2.zip › Figure 3—figure supplement 4—source data 2/input-flag-blot.tif]

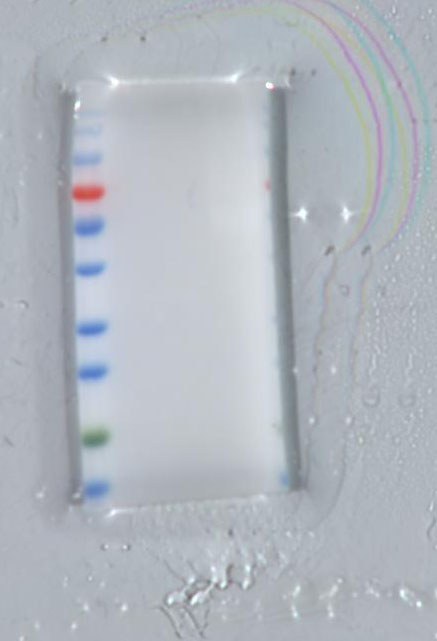

Supplement: Figure 3—figure supplement 4—source data 2. [file elife-108737-fig3-figsupp4-data2.zip › Figure 3—figure supplement 4—source data 2/input-flag-marker.jpg]

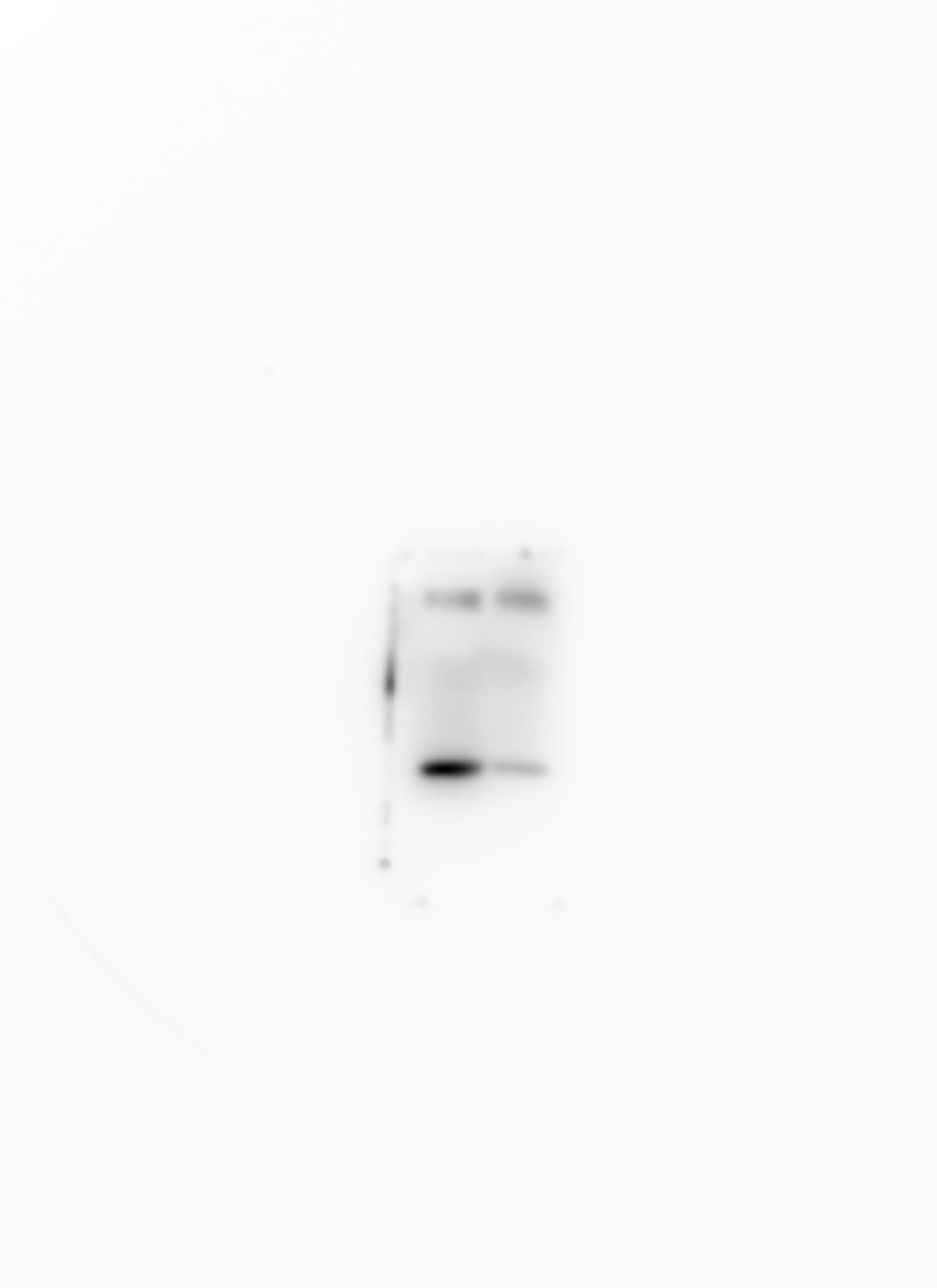

Supplement: Figure 3—figure supplement 4—source data 2. [file elife-108737-fig3-figsupp4-data2.zip › Figure 3—figure supplement 4—source data 2/input-myc-blot.tif]

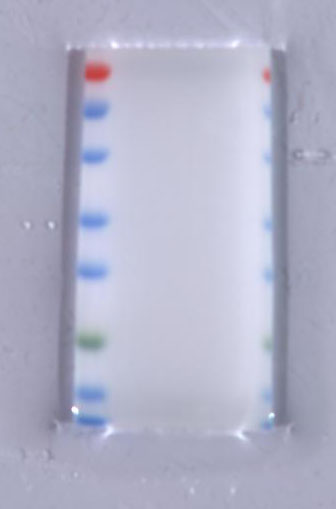

Supplement: Figure 3—figure supplement 4—source data 2. [file elife-108737-fig3-figsupp4-data2.zip › Figure 3—figure supplement 4—source data 2/input-myc-marker.jpg]

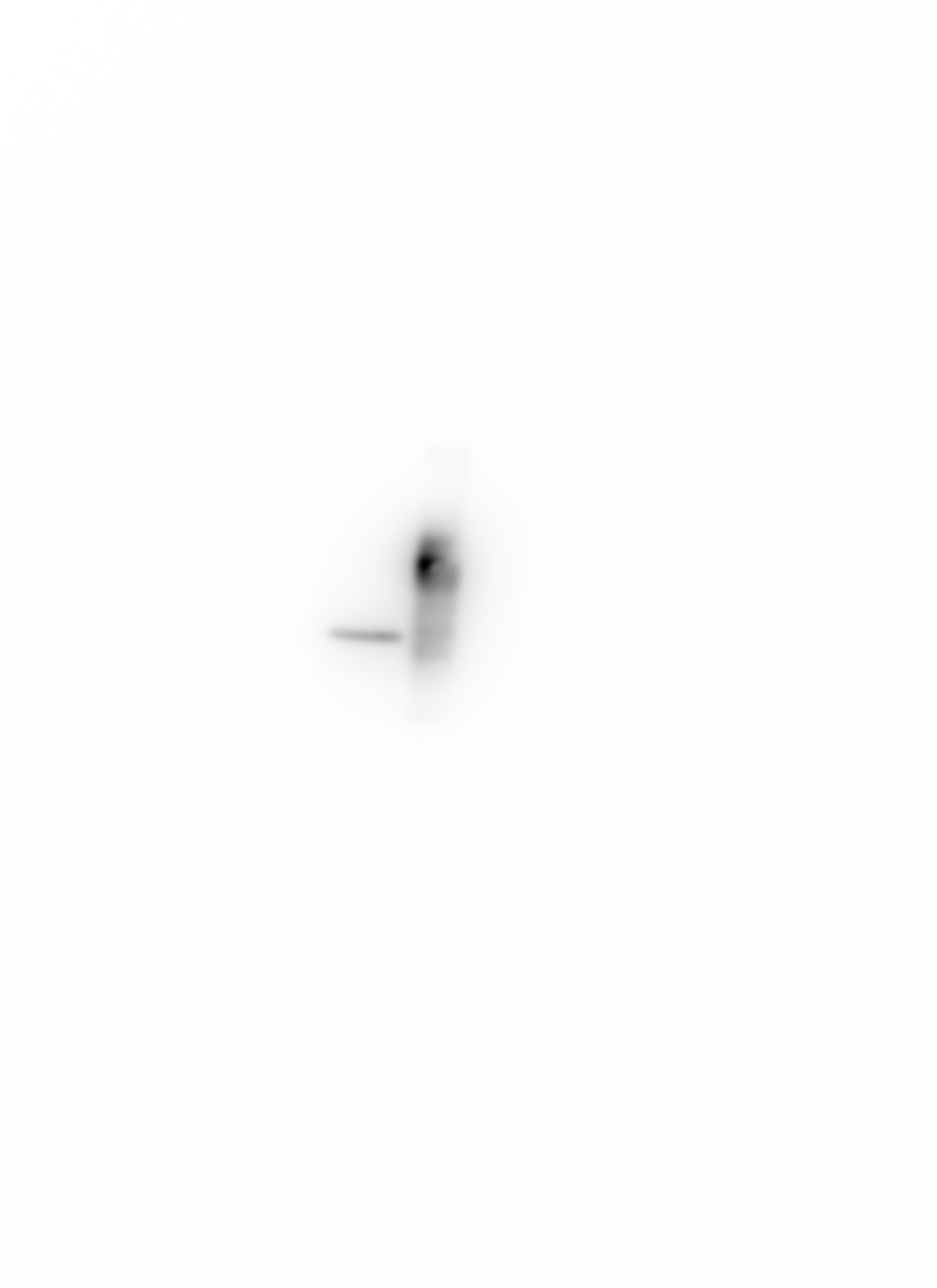

Supplement: Figure 3—figure supplement 4—source data 2. [file elife-108737-fig3-figsupp4-data2.zip › Figure 3—figure supplement 4—source data 2/IP-flag-blot.tif]

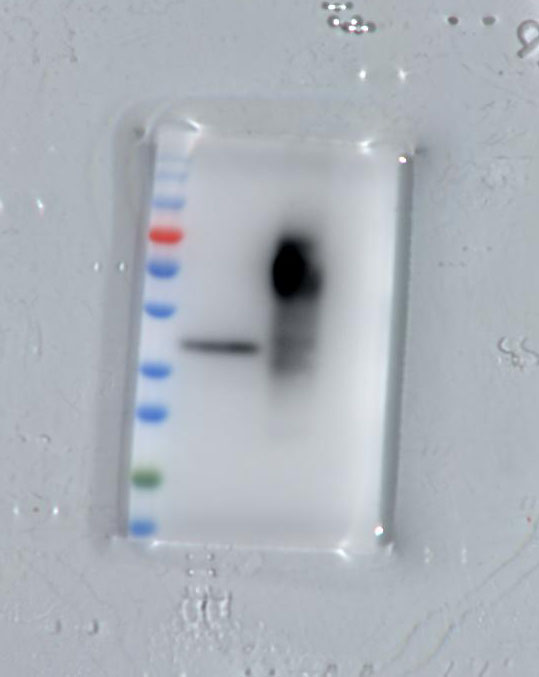

Supplement: Figure 3—figure supplement 4—source data 2. [file elife-108737-fig3-figsupp4-data2.zip › Figure 3—figure supplement 4—source data 2/IP-flag-marker.jpg]

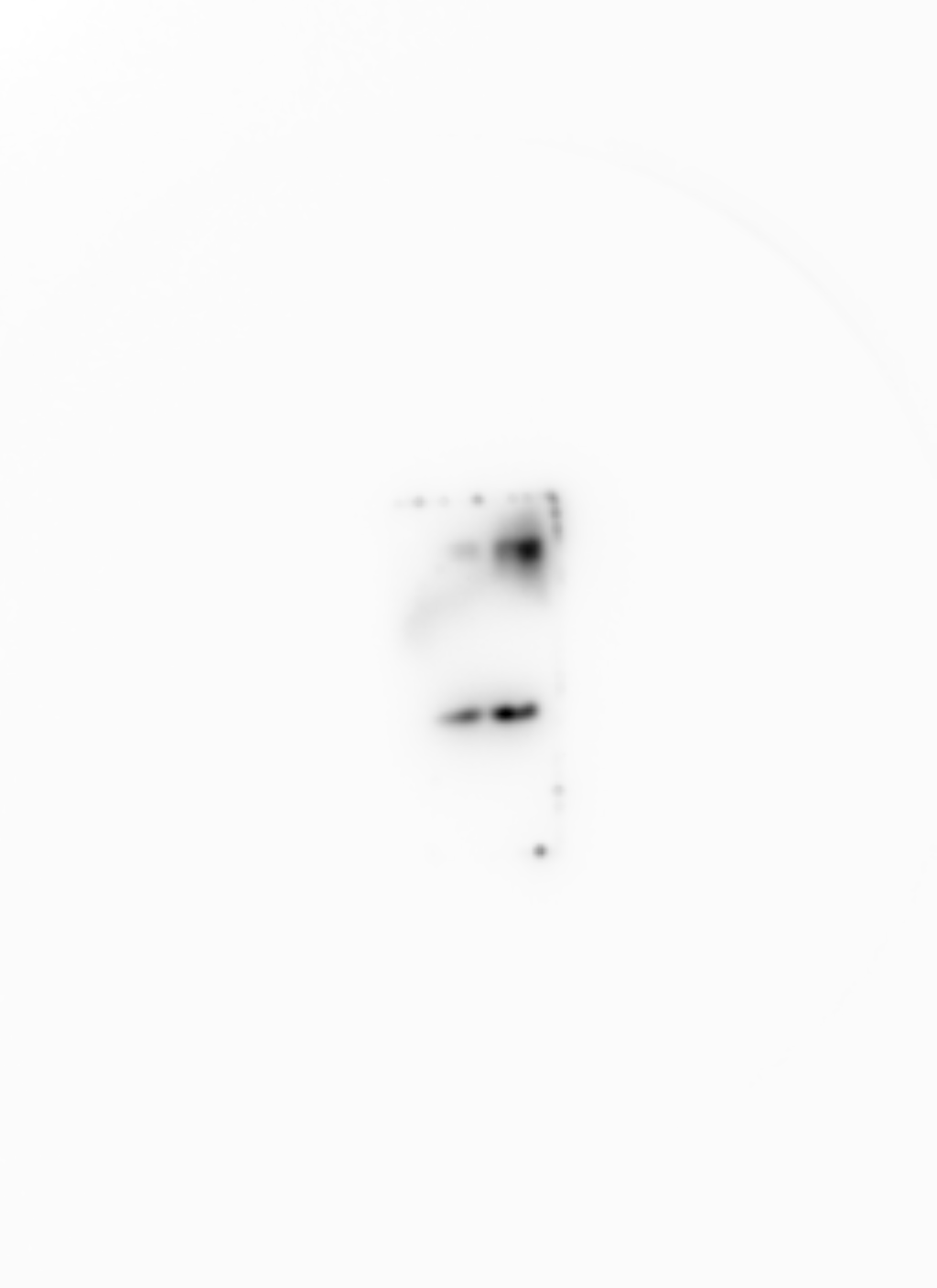

Supplement: Figure 3—figure supplement 4—source data 2. [file elife-108737-fig3-figsupp4-data2.zip › Figure 3—figure supplement 4—source data 2/IP-myc-blot.tif]

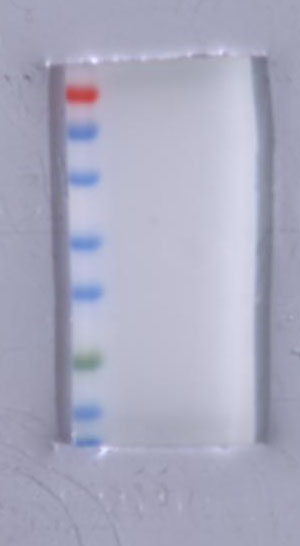

Supplement: Figure 3—figure supplement 4—source data 2. [file elife-108737-fig3-figsupp4-data2.zip › Figure 3—figure supplement 4—source data 2/IP-myc-marker.jpg]

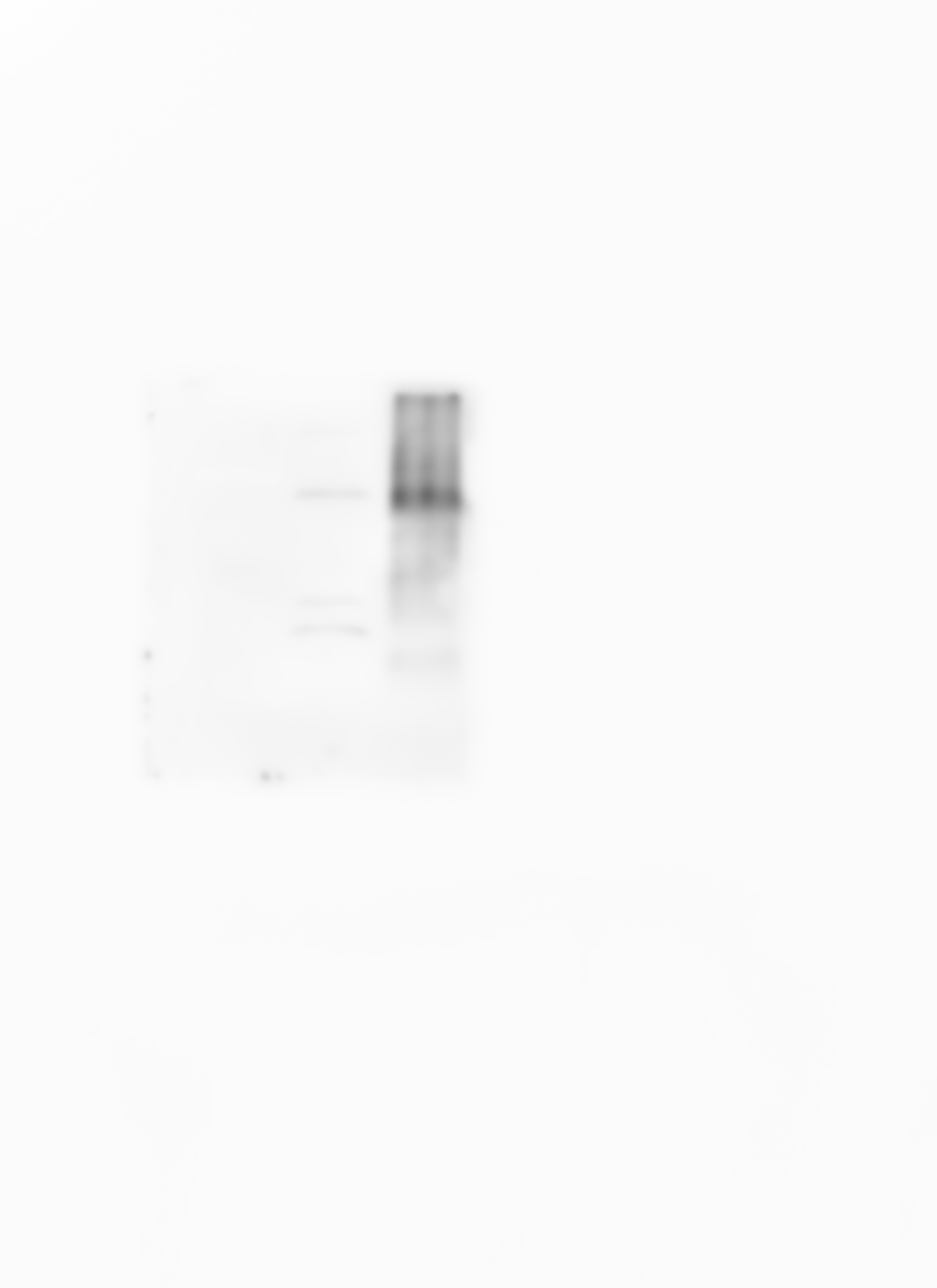

Supplement: Figure 3—figure supplement 4—source data 4. [file elife-108737-fig3-figsupp4-data4.zip › Figure 3—figure supplement 4—source data 4/input-flag-blot.tif]

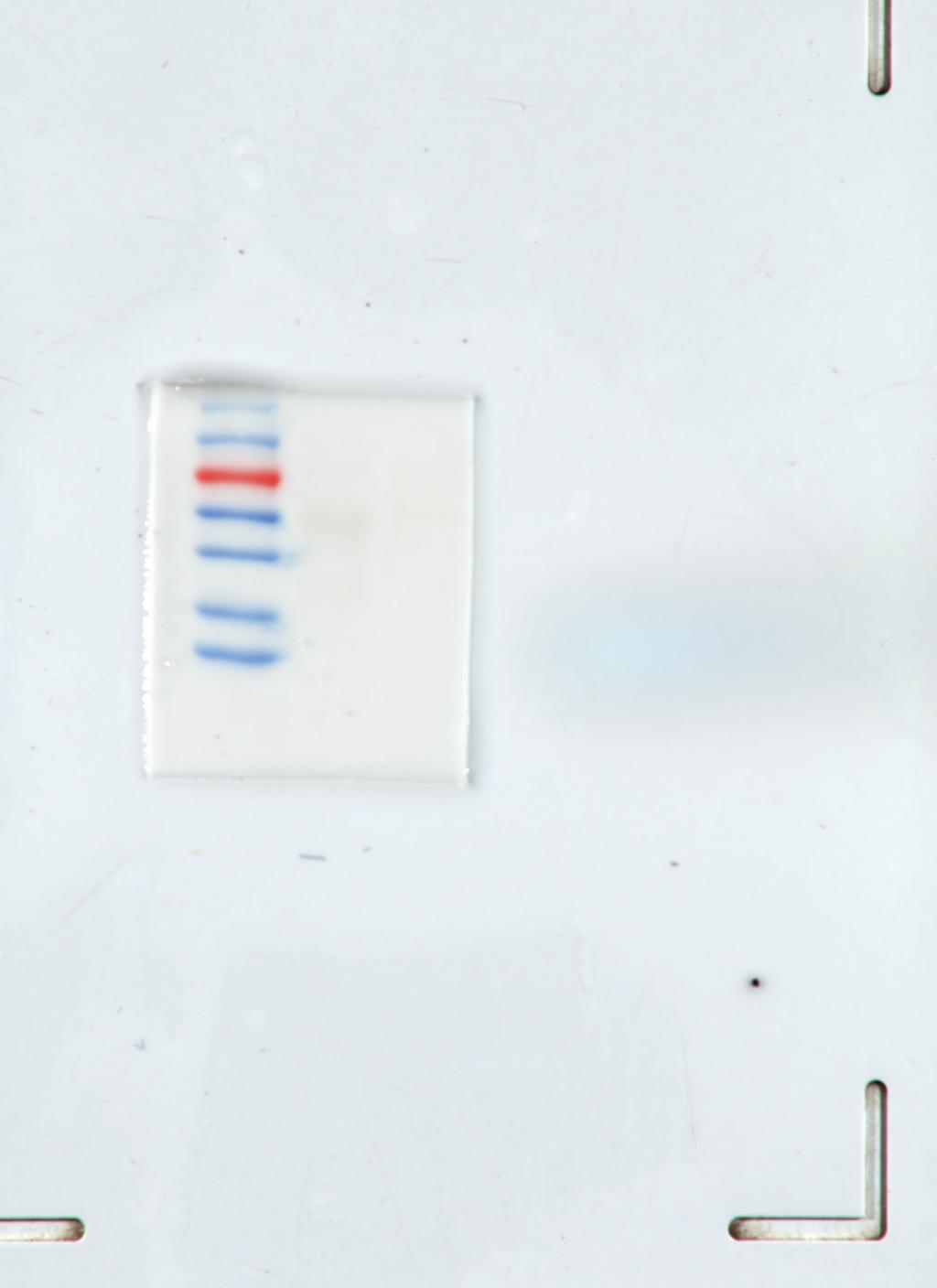

Supplement: Figure 3—figure supplement 4—source data 4. [file elife-108737-fig3-figsupp4-data4.zip › Figure 3—figure supplement 4—source data 4/input-flag-marker.jpg]

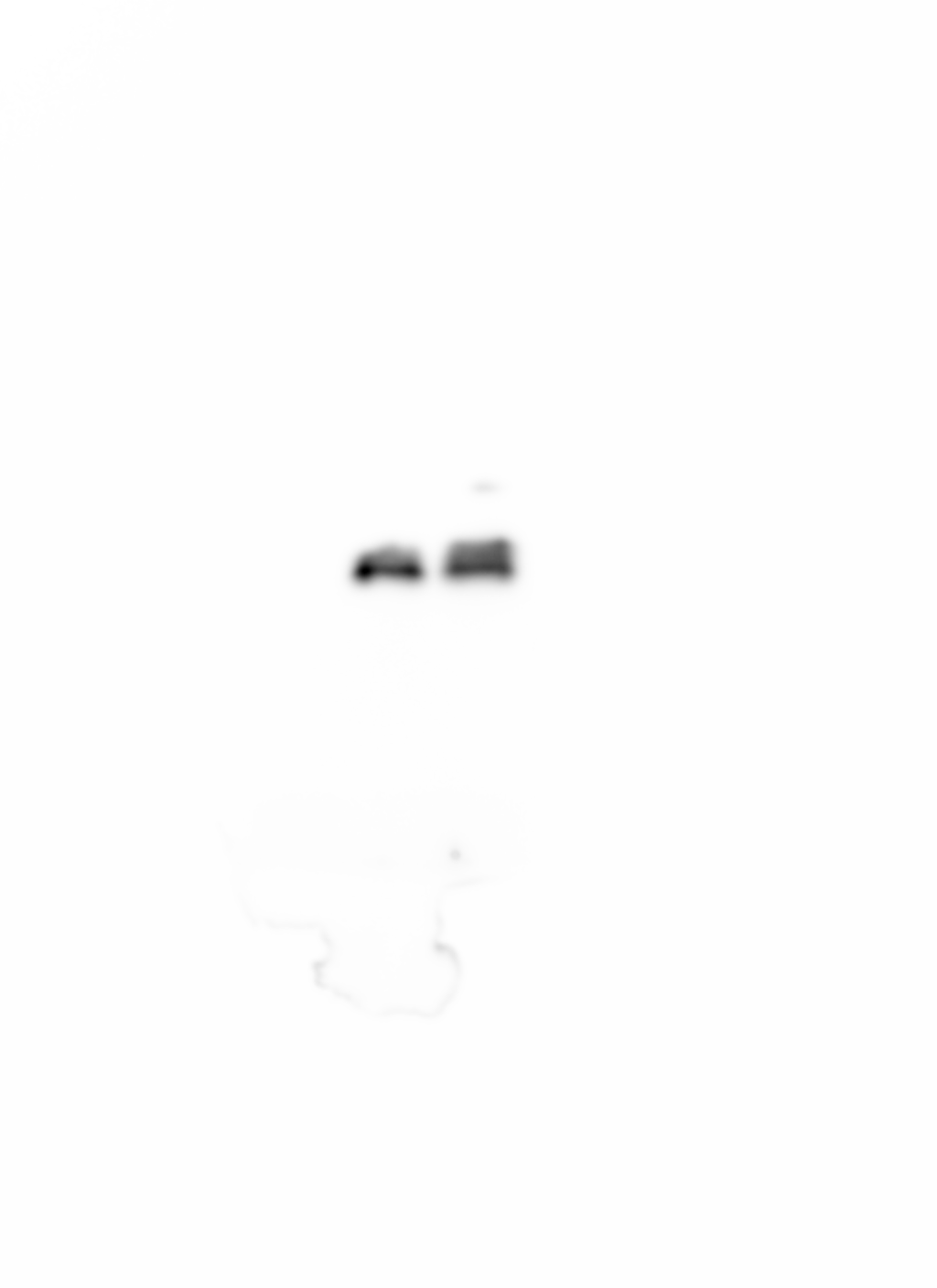

Supplement: Figure 3—figure supplement 4—source data 4. [file elife-108737-fig3-figsupp4-data4.zip › Figure 3—figure supplement 4—source data 4/input-myc-blot.tif]

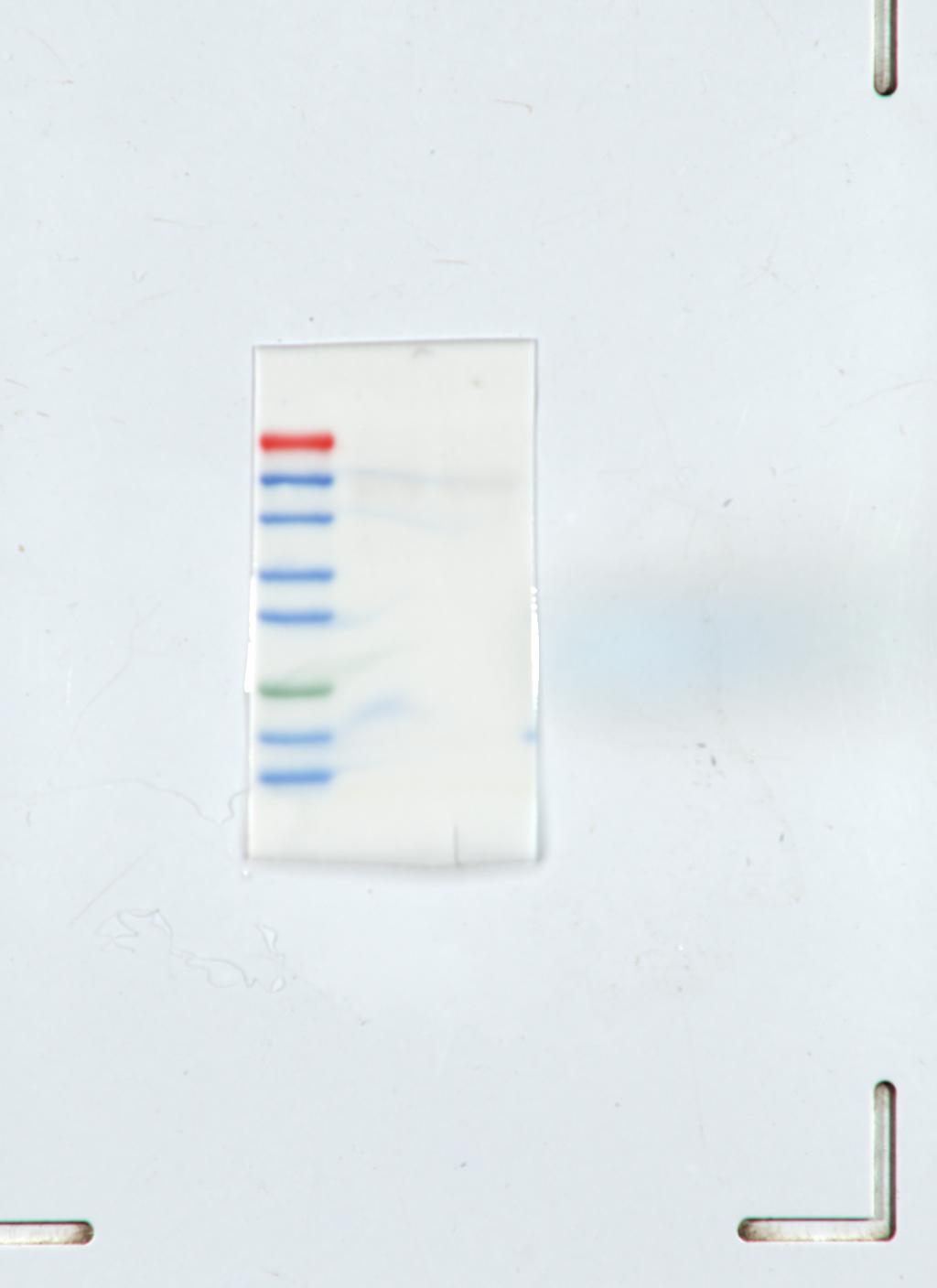

Supplement: Figure 3—figure supplement 4—source data 4. [file elife-108737-fig3-figsupp4-data4.zip › Figure 3—figure supplement 4—source data 4/input-myc-marker.jpg]

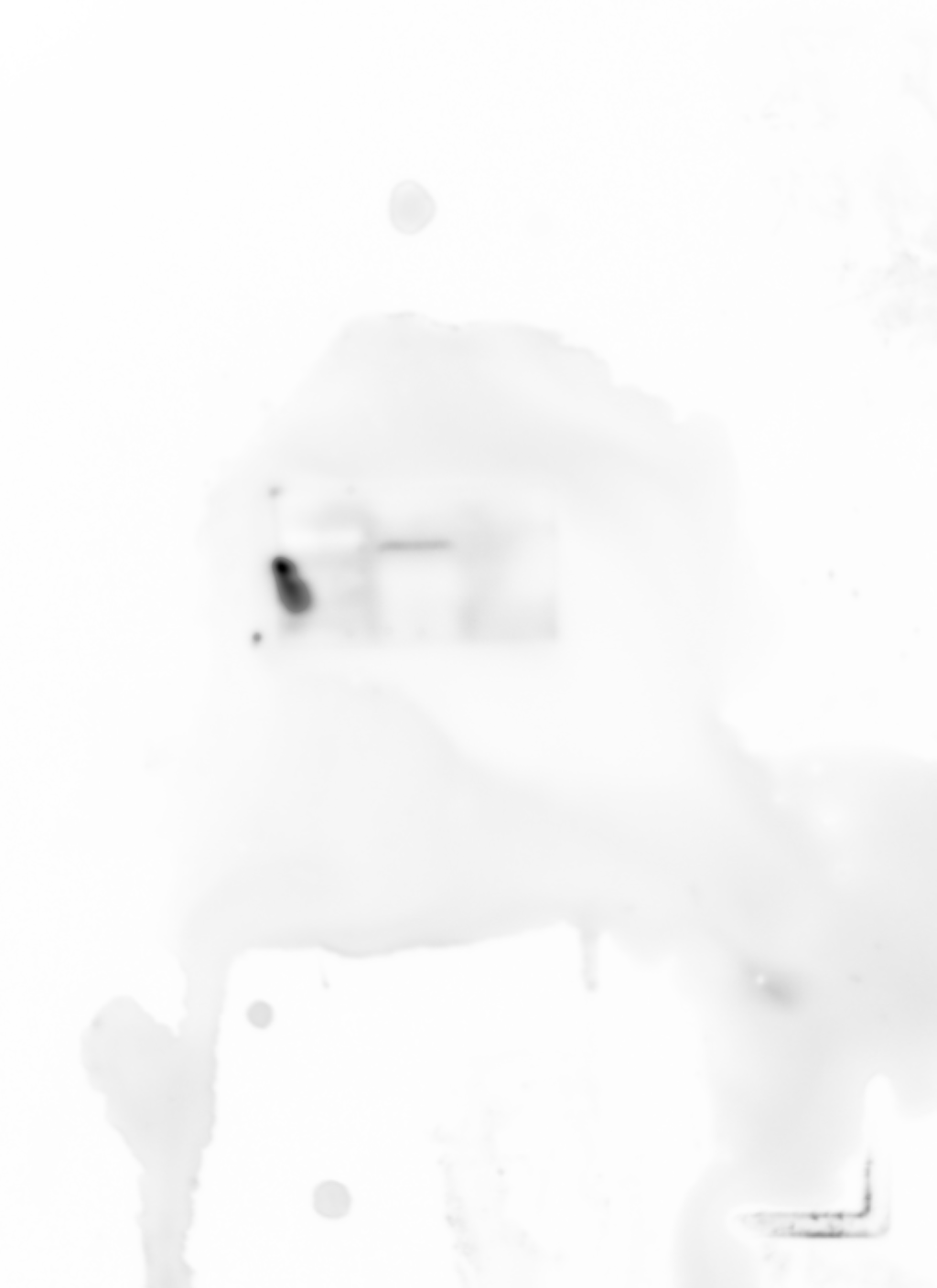

Supplement: Figure 3—figure supplement 4—source data 4. [file elife-108737-fig3-figsupp4-data4.zip › Figure 3—figure supplement 4—source data 4/IP-flag-blot.tif]

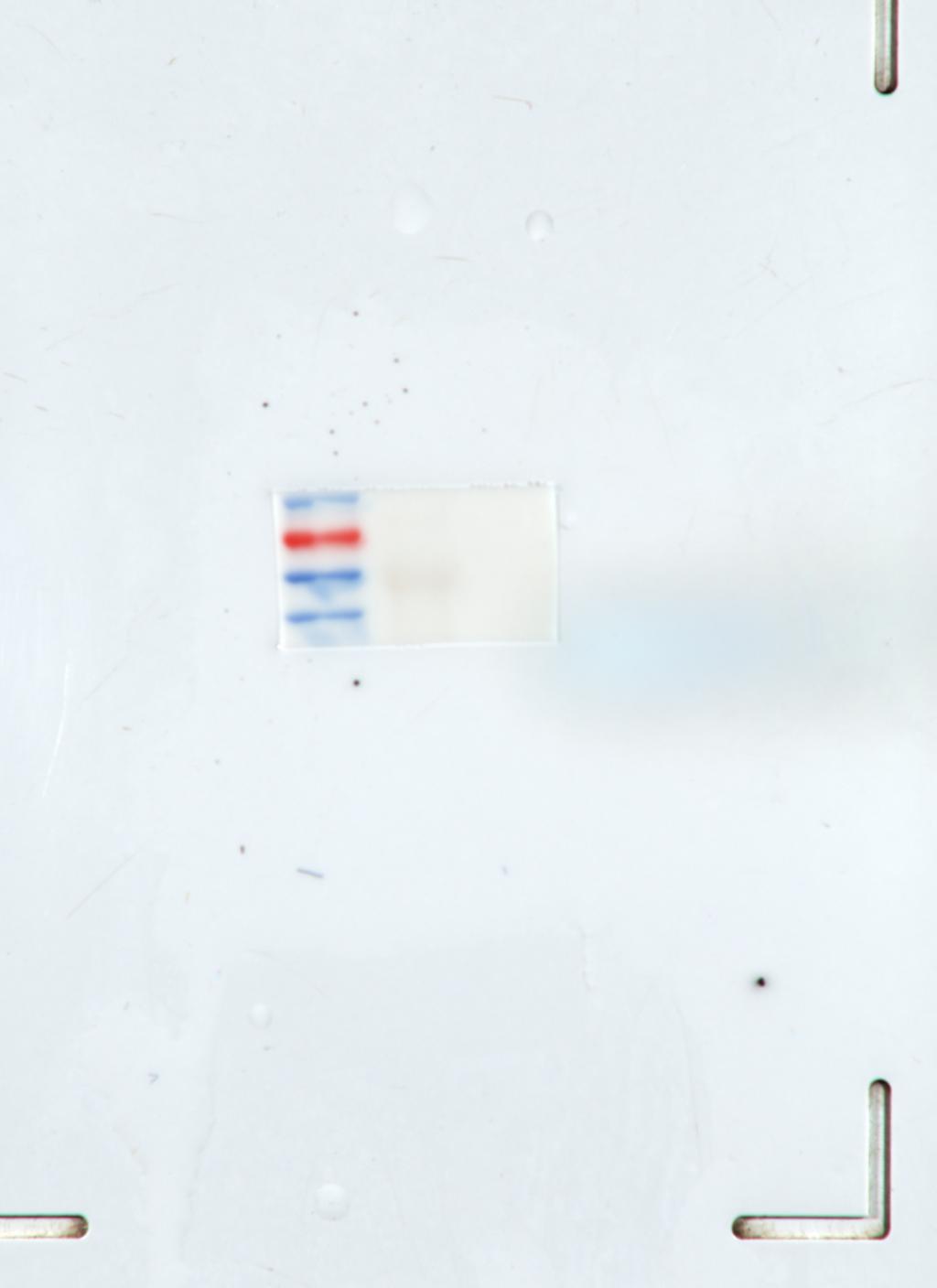

Supplement: Figure 3—figure supplement 4—source data 4. [file elife-108737-fig3-figsupp4-data4.zip › Figure 3—figure supplement 4—source data 4/ip-flag-marker.jpg]

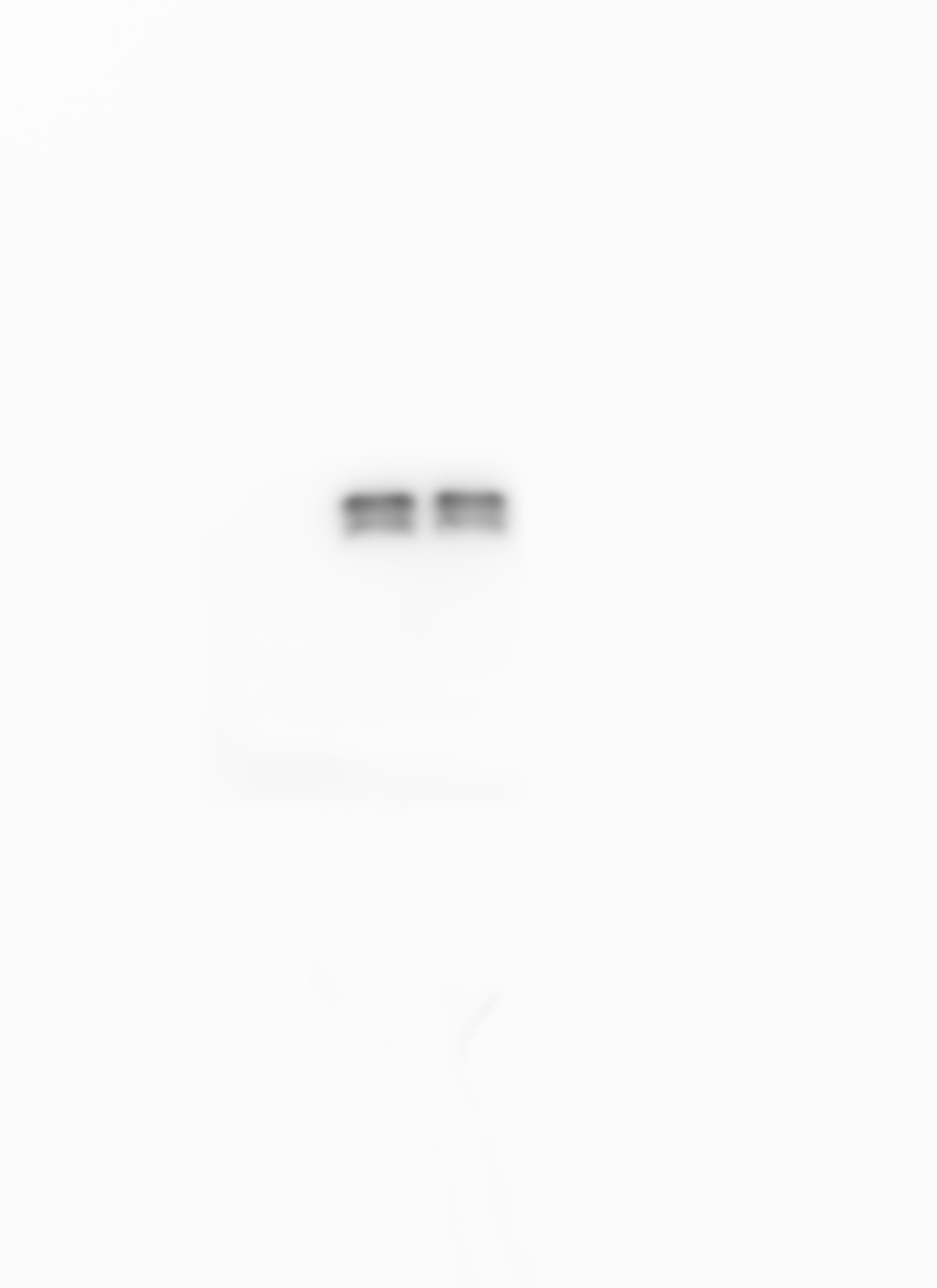

Supplement: Figure 3—figure supplement 4—source data 4. [file elife-108737-fig3-figsupp4-data4.zip › Figure 3—figure supplement 4—source data 4/IP-myc-blot.tif]

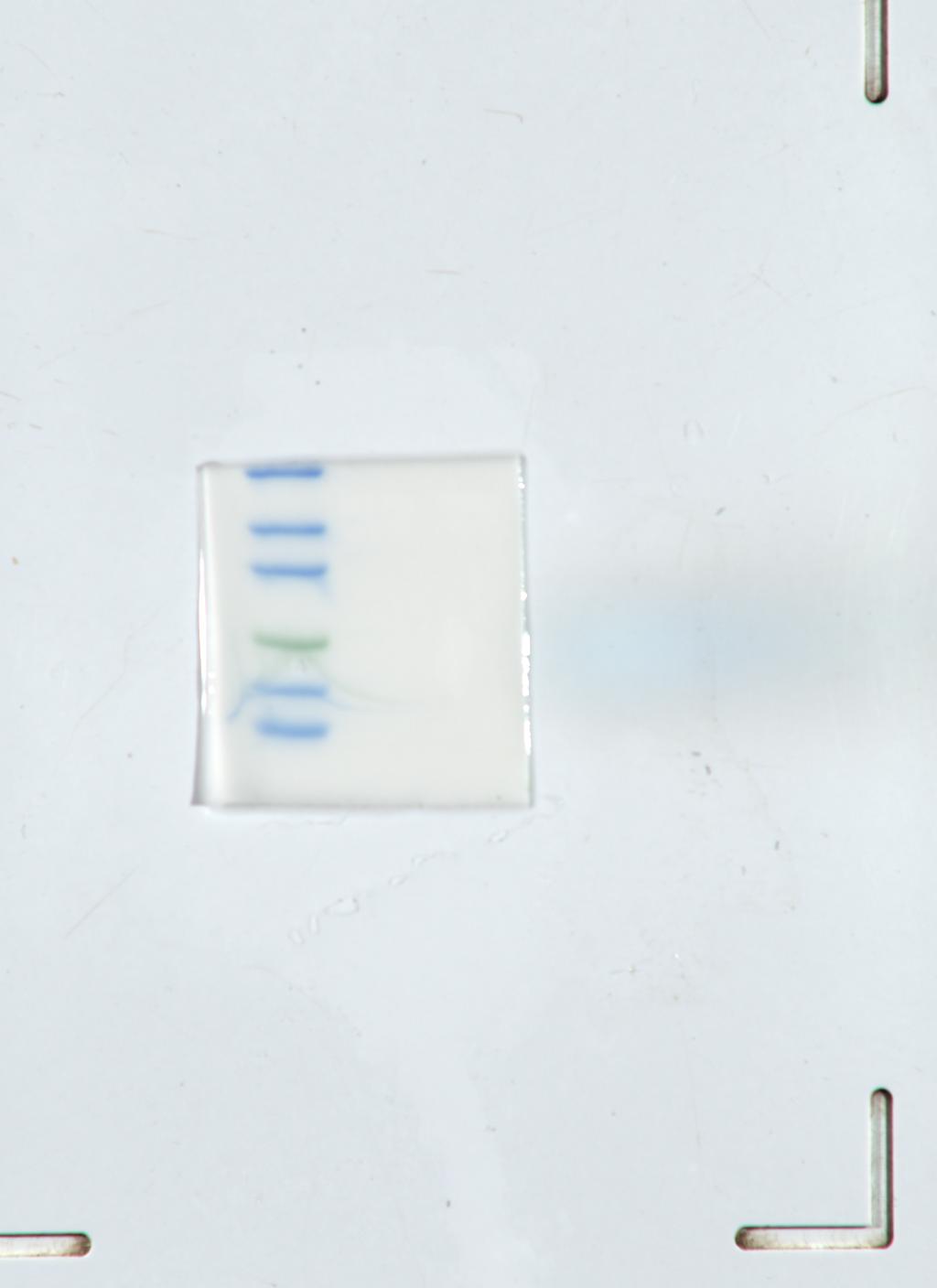

Supplement: Figure 3—figure supplement 4—source data 4. [file elife-108737-fig3-figsupp4-data4.zip › Figure 3—figure supplement 4—source data 4/IP-myc-marker.jpg]

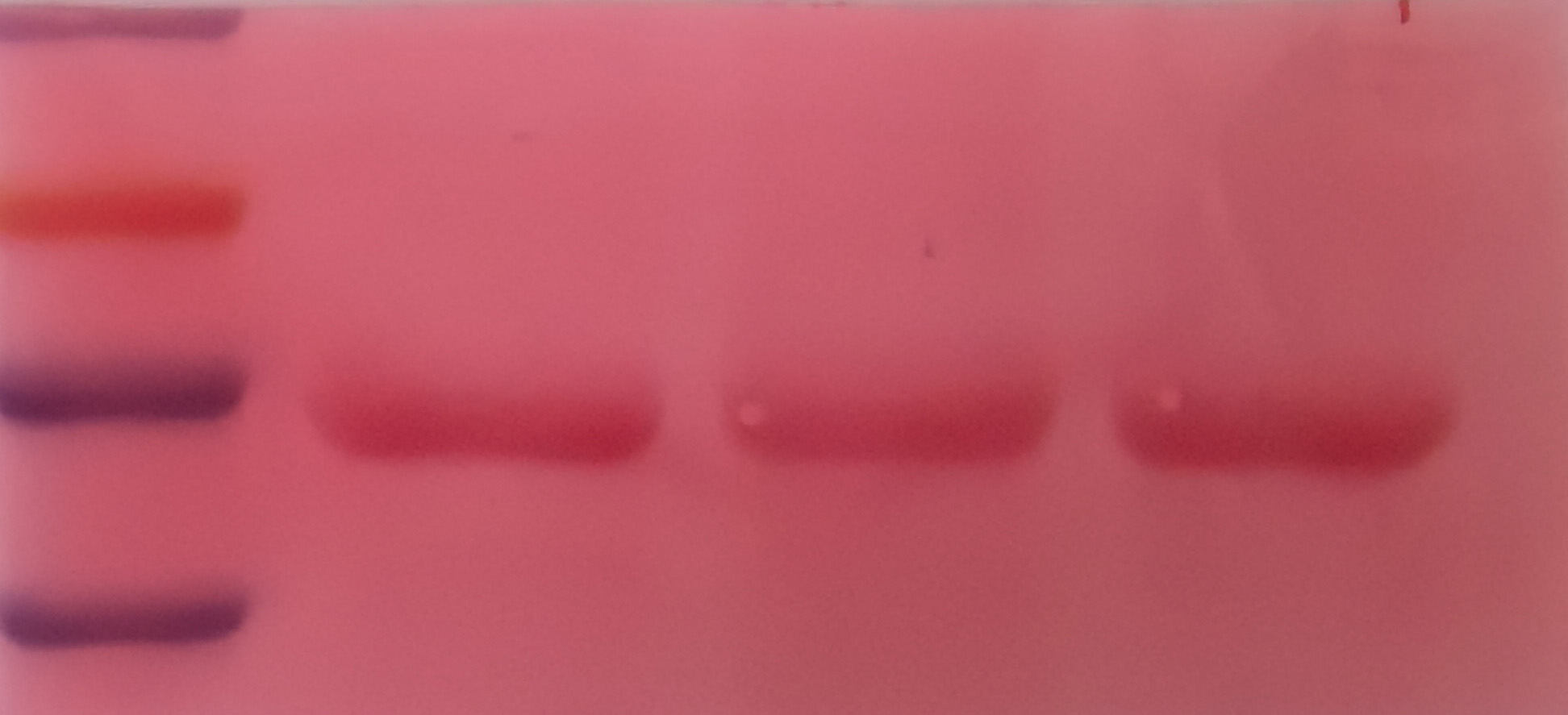

Supplement: Figure 4—source data 2. [file elife-108737-fig4-data2.zip › Figure 4—source data 2/RbCL-left.jpg]

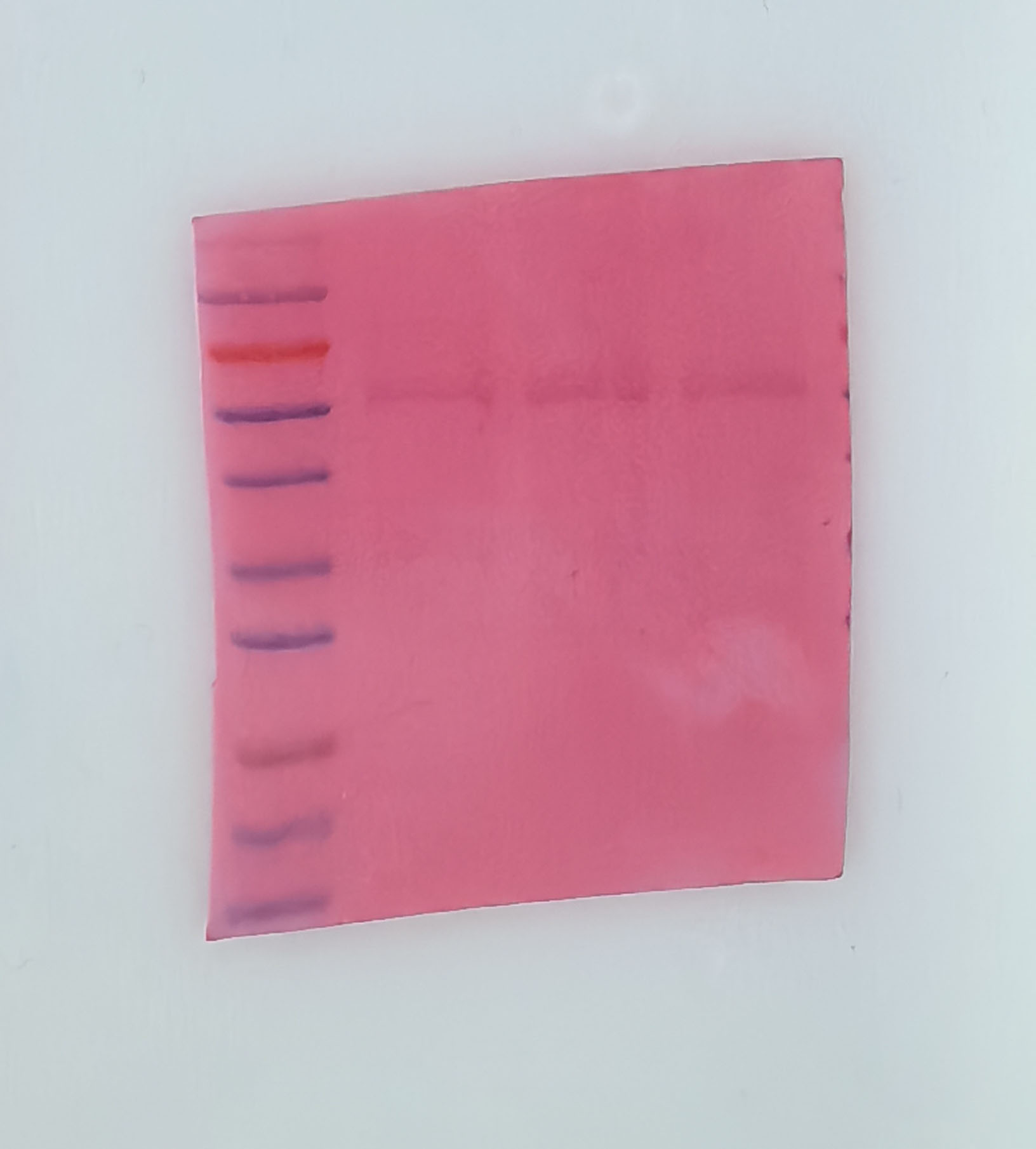

Supplement: Figure 4—source data 2. [file elife-108737-fig4-data2.zip › Figure 4—source data 2/RbCL-right.jpg]

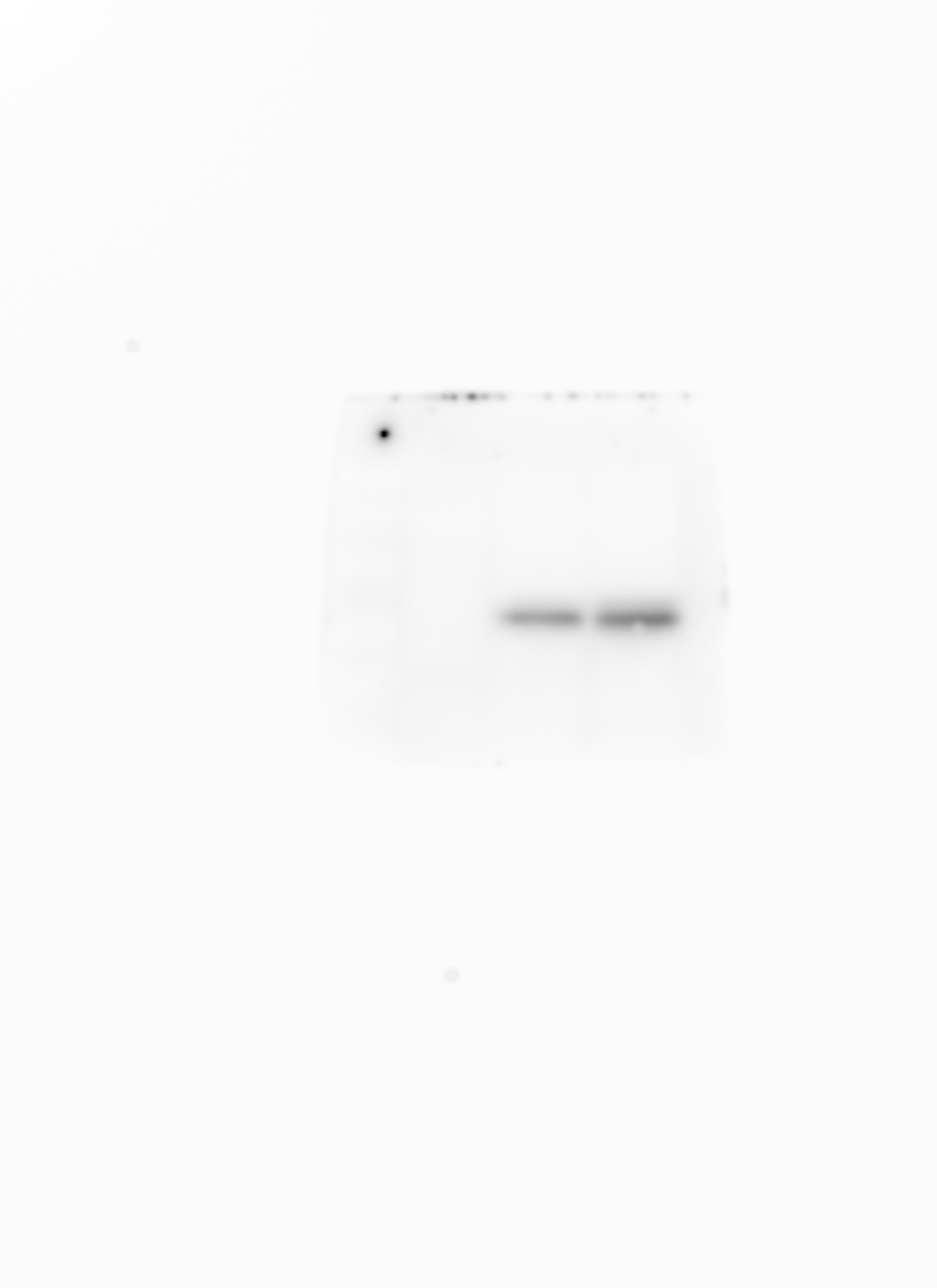

Supplement: Figure 4—source data 2. [file elife-108737-fig4-data2.zip › Figure 4—source data 2/α-flag-blot-left.tif]

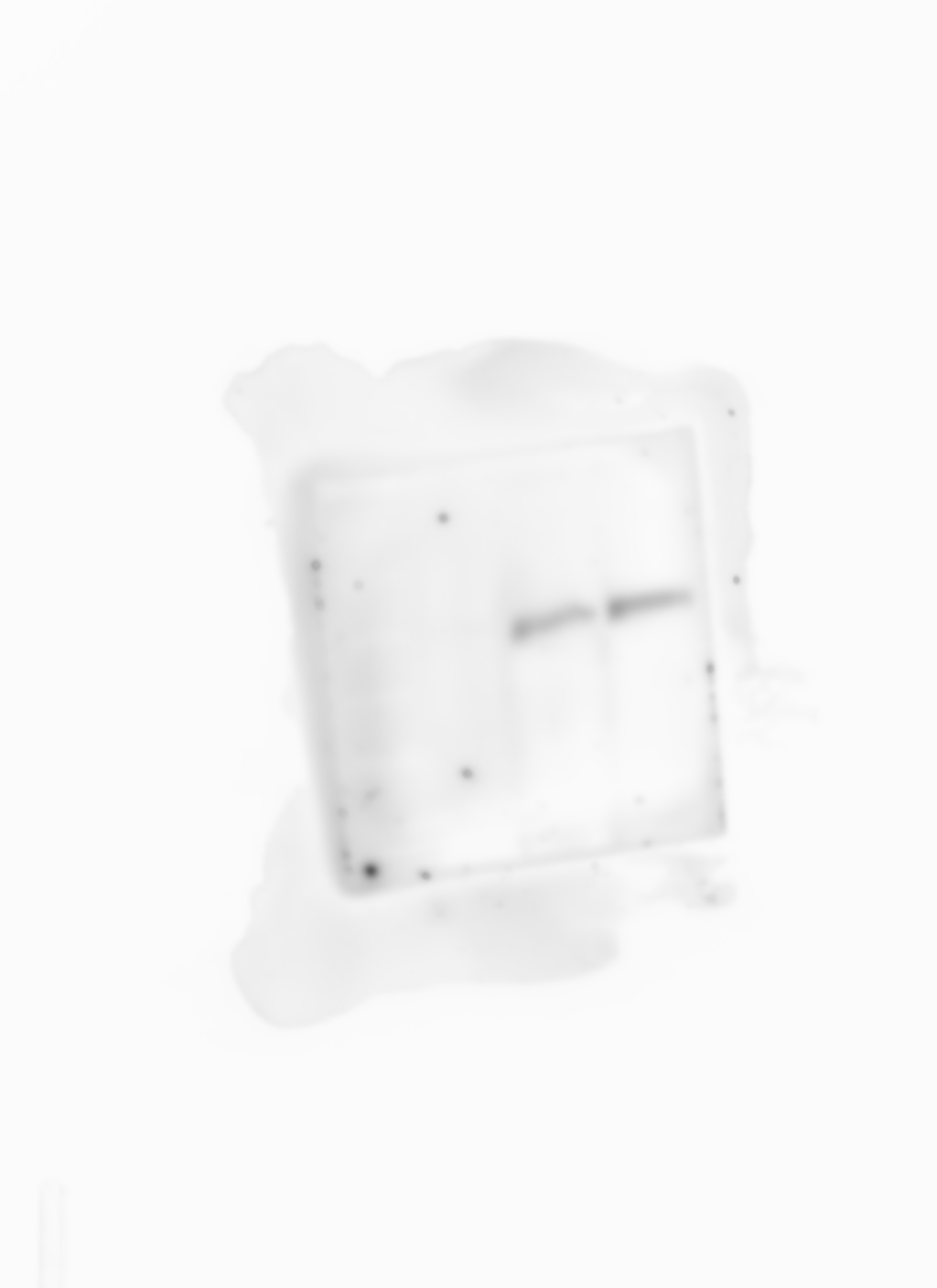

Supplement: Figure 4—source data 2. [file elife-108737-fig4-data2.zip › Figure 4—source data 2/α-flag-blot-right.tif]

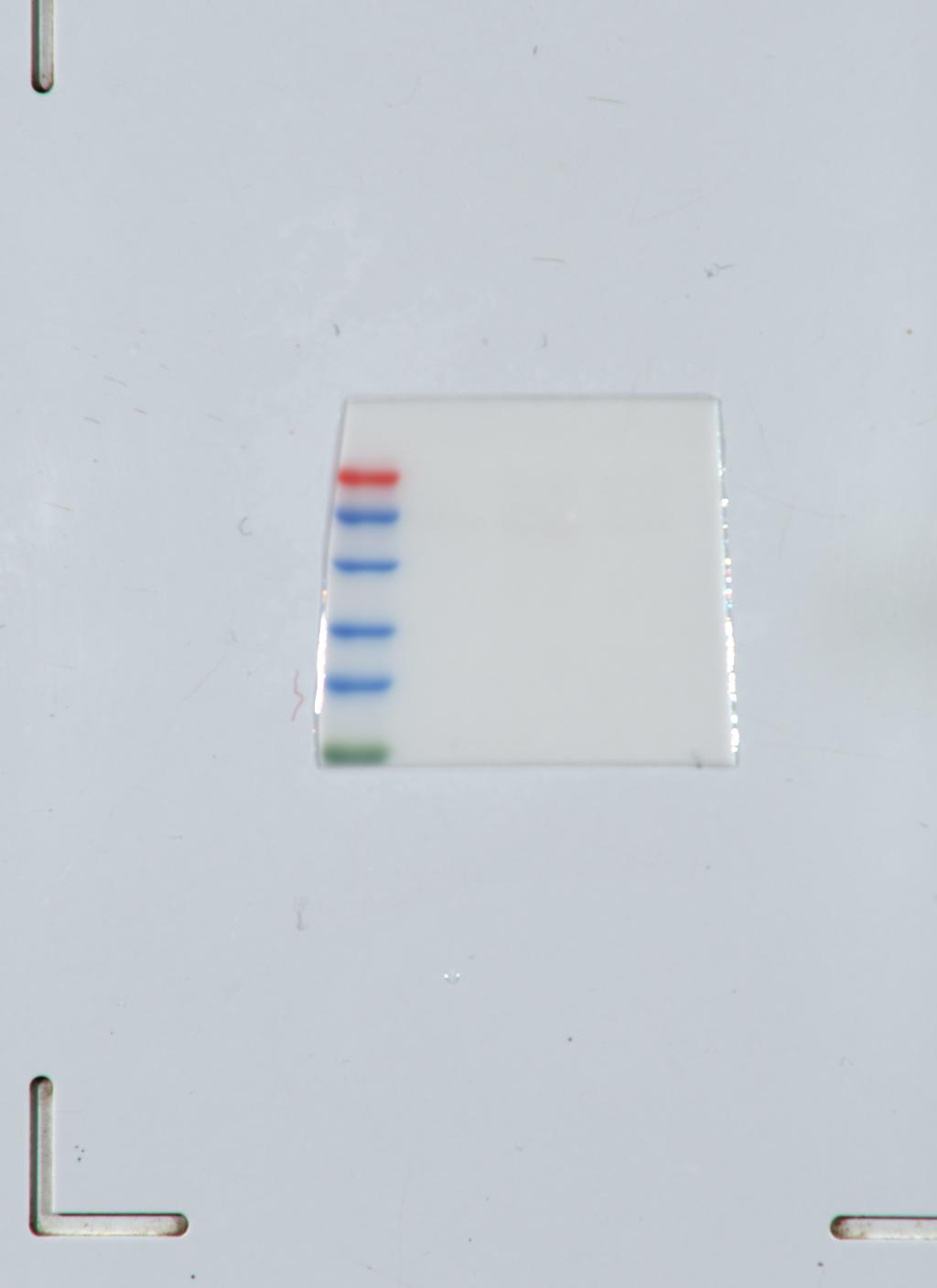

Supplement: Figure 4—source data 2. [file elife-108737-fig4-data2.zip › Figure 4—source data 2/α-flag-marker-left.jpg]

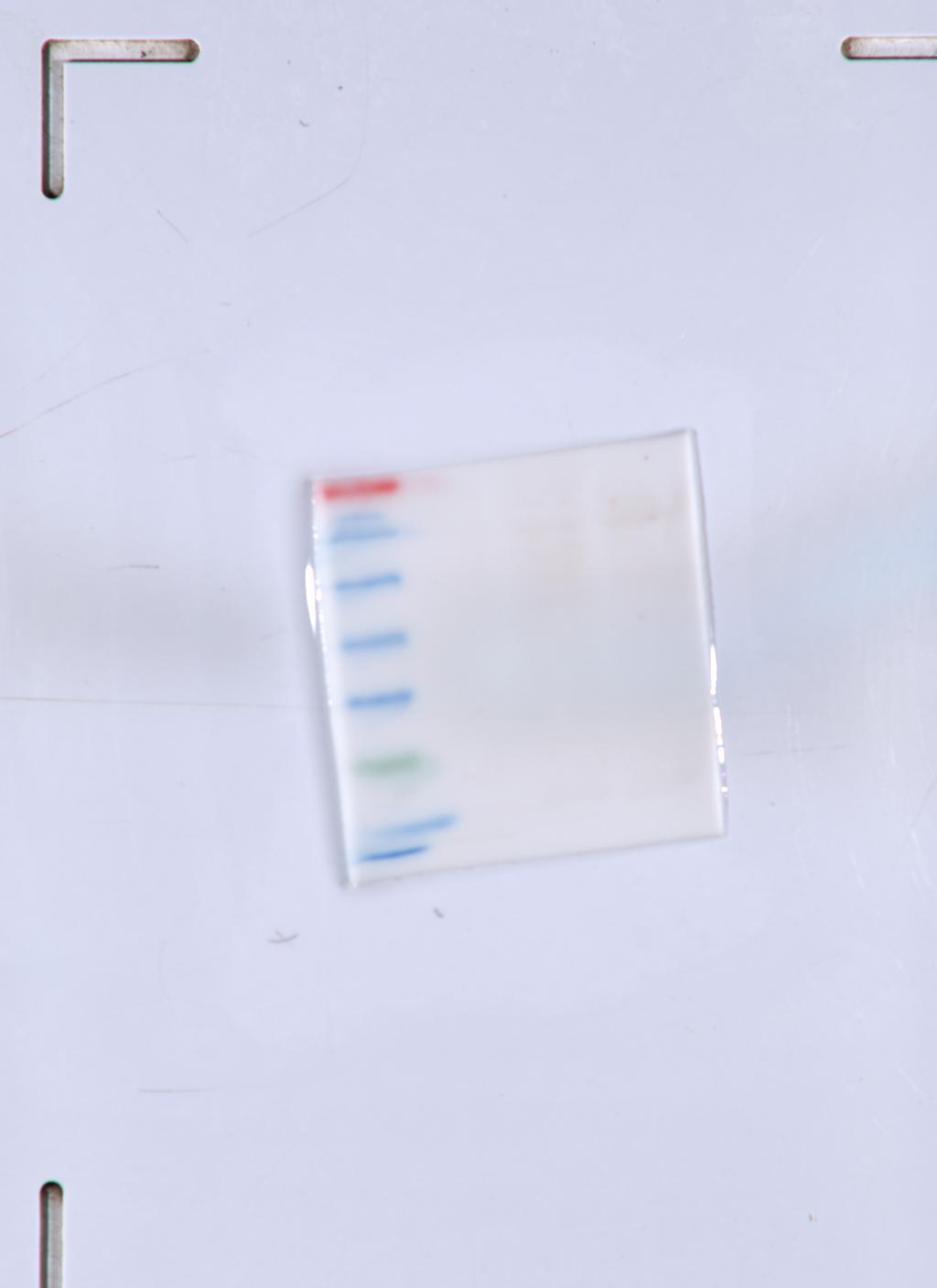

Supplement: Figure 4—source data 2. [file elife-108737-fig4-data2.zip › Figure 4—source data 2/α-flag-marker-right.jpg]

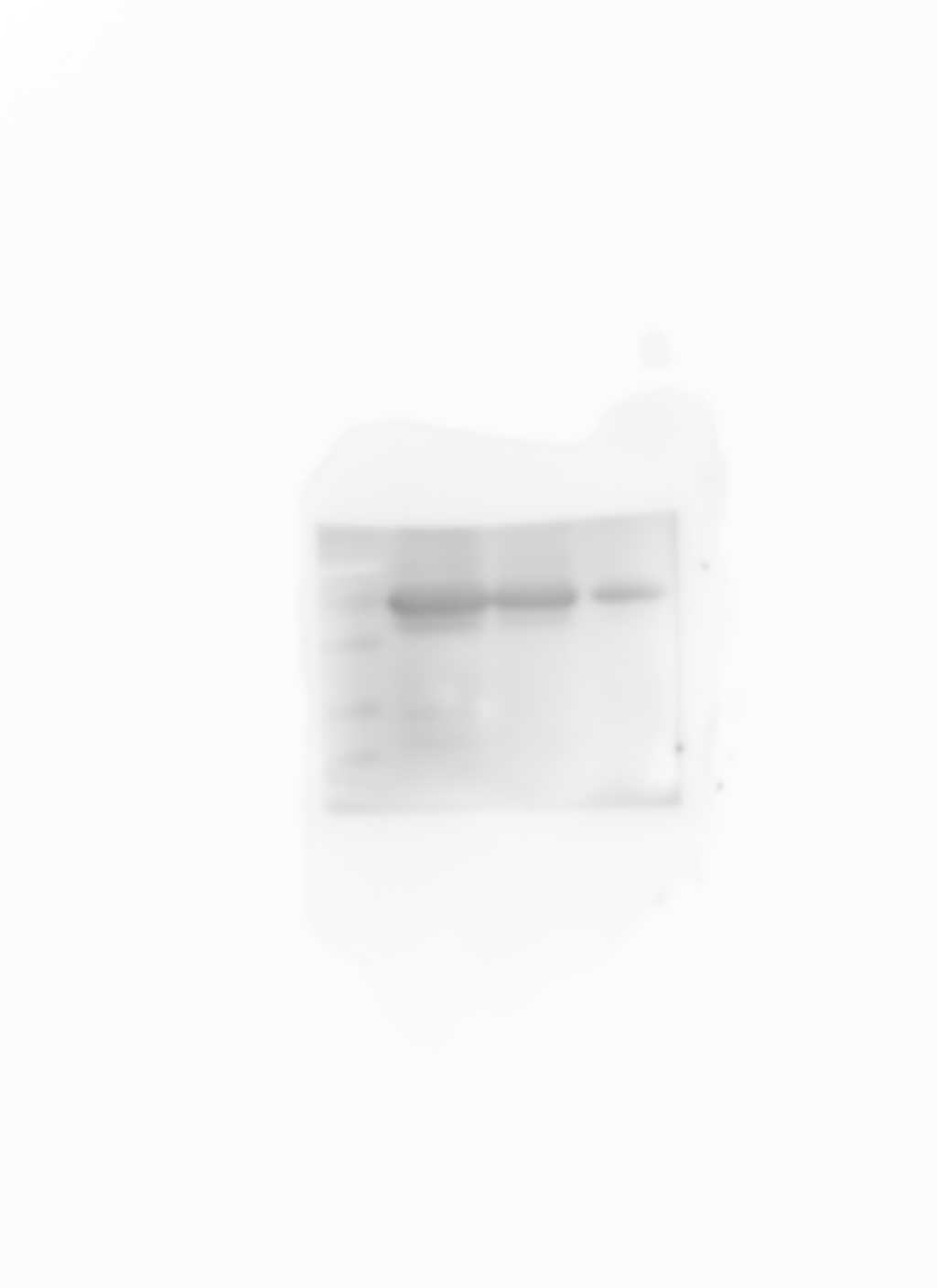

Supplement: Figure 4—source data 2. [file elife-108737-fig4-data2.zip › Figure 4—source data 2/α-NtRLP4-blot-left.tif]

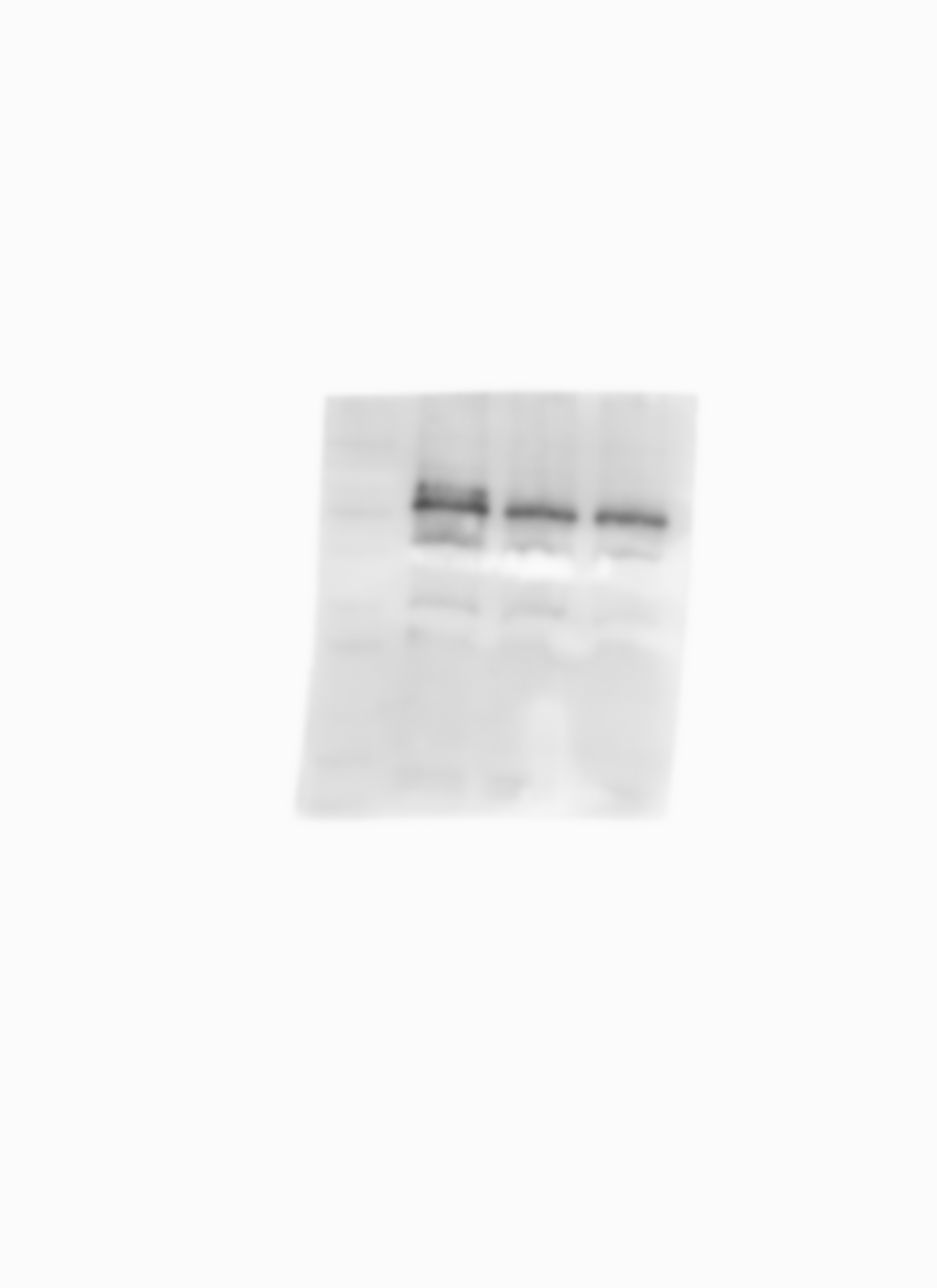

Supplement: Figure 4—source data 2. [file elife-108737-fig4-data2.zip › Figure 4—source data 2/α-NtRLP4-blot-right.tif]

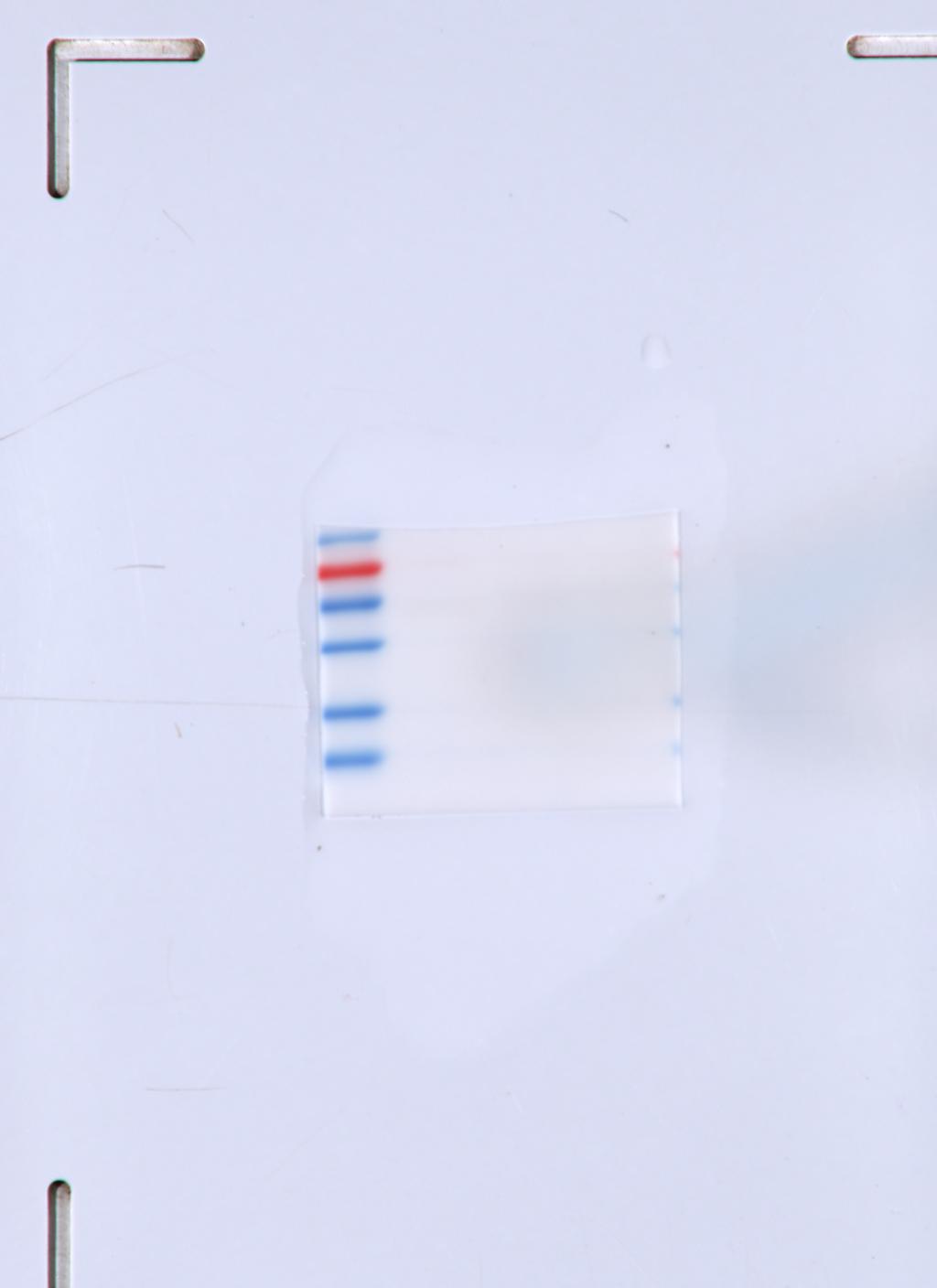

Supplement: Figure 4—source data 2. [file elife-108737-fig4-data2.zip › Figure 4—source data 2/α-NtRLP4-marker-left.jpg]

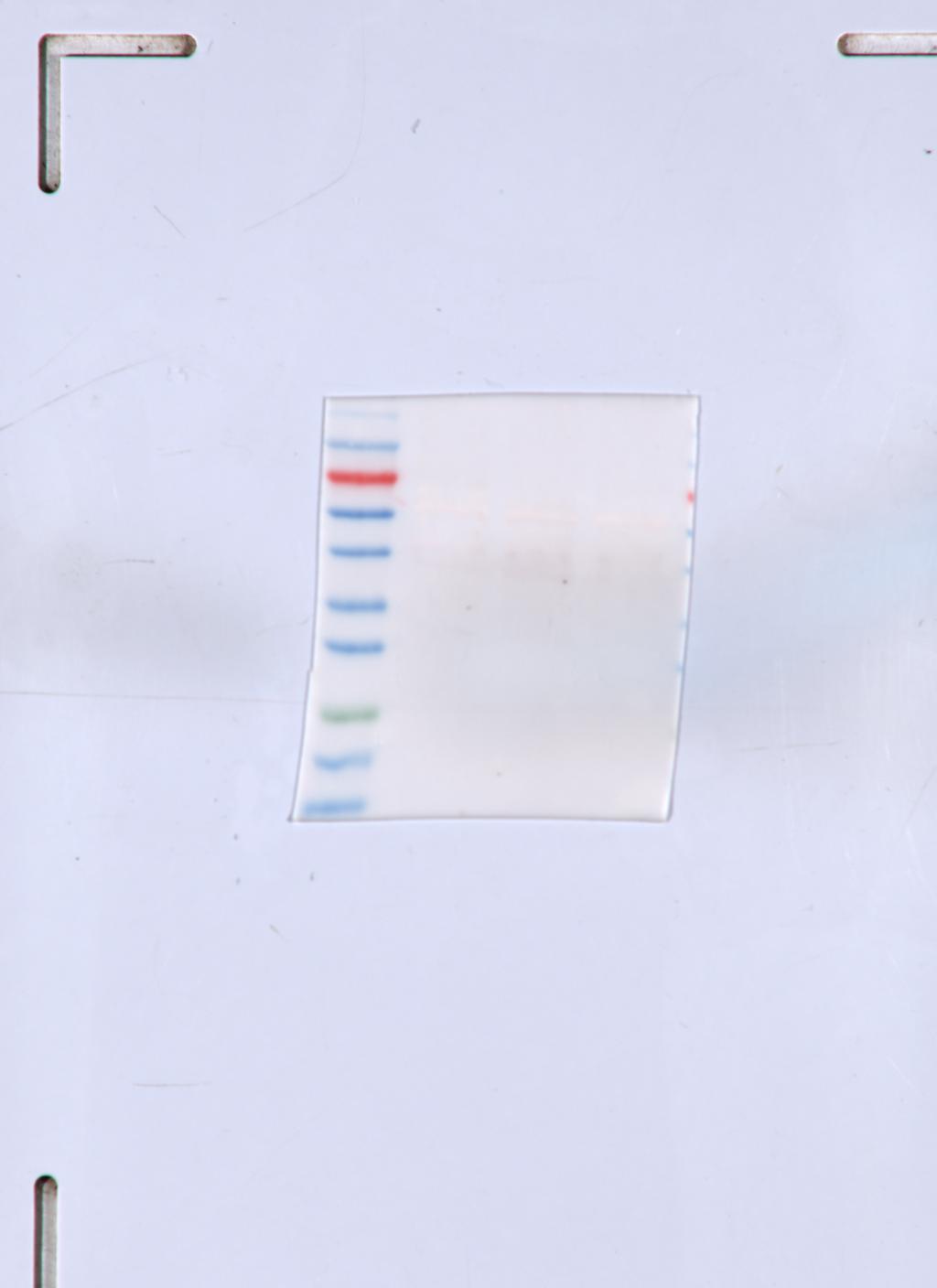

Supplement: Figure 4—source data 2. [file elife-108737-fig4-data2.zip › Figure 4—source data 2/α-NtRLP4-marker-right.jpg]

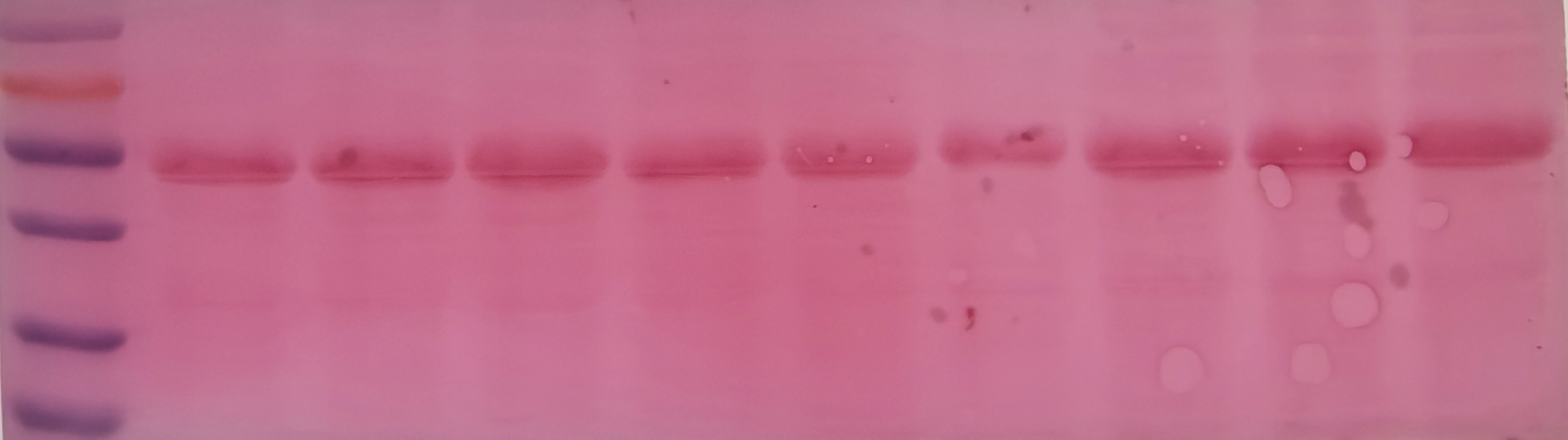

Supplement: Figure 4—source data 4. [file elife-108737-fig4-data4.zip › Figure 4—source data 4/RbCL.jpg]

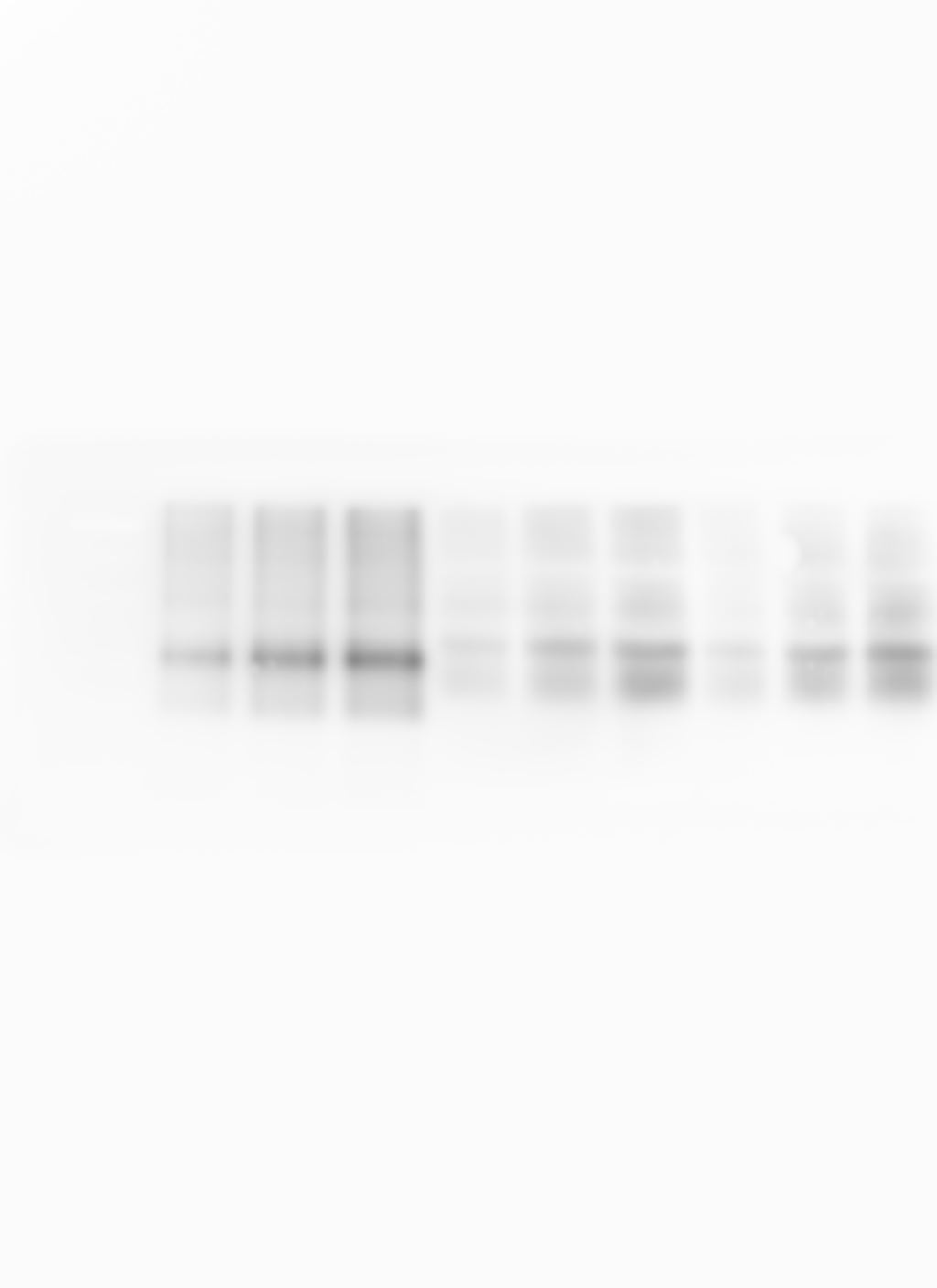

Supplement: Figure 4—source data 4. [file elife-108737-fig4-data4.zip › Figure 4—source data 4/α-flag-blot.tif]

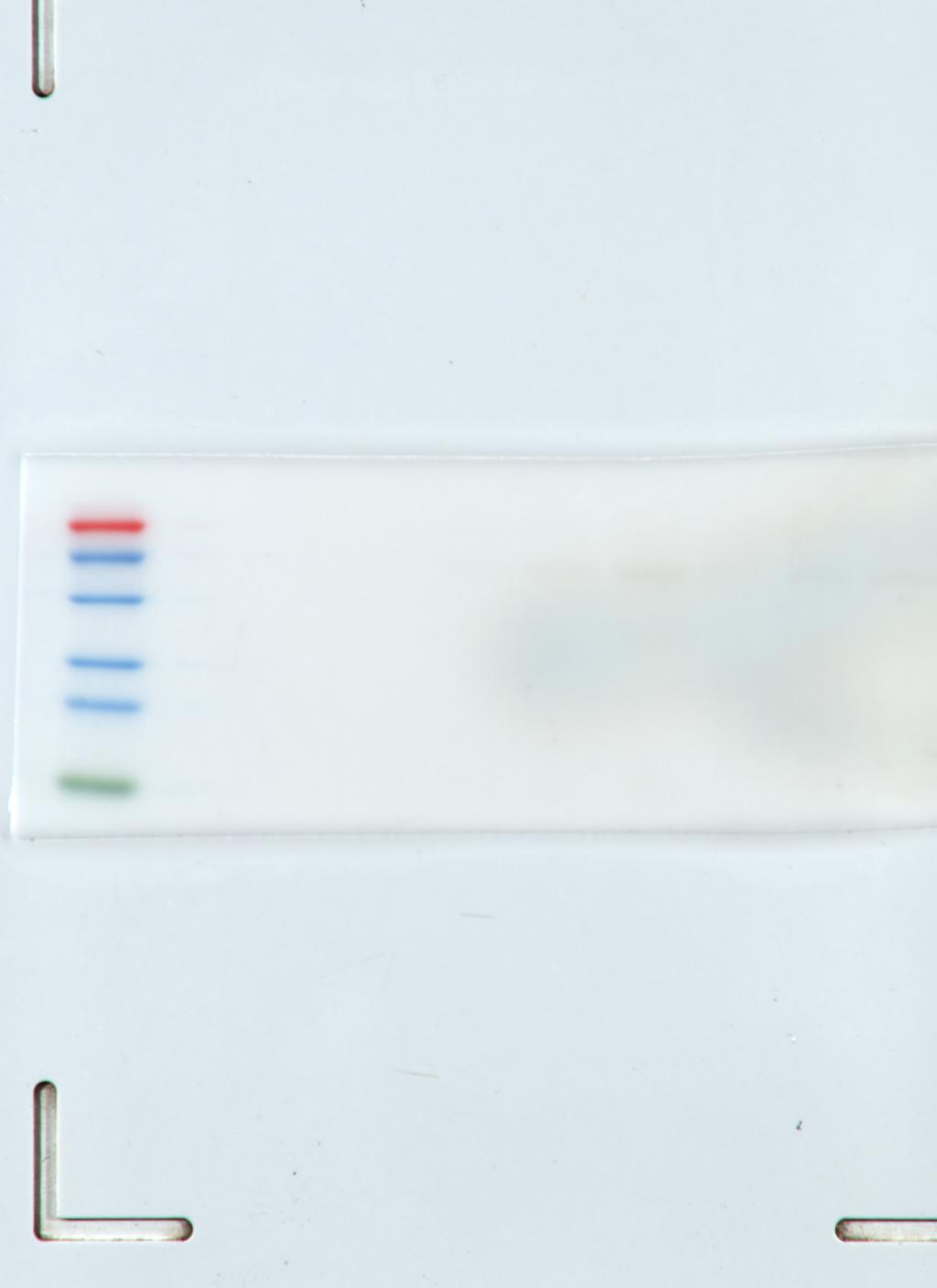

Supplement: Figure 4—source data 4. [file elife-108737-fig4-data4.zip › Figure 4—source data 4/α-flag-marker.jpg]

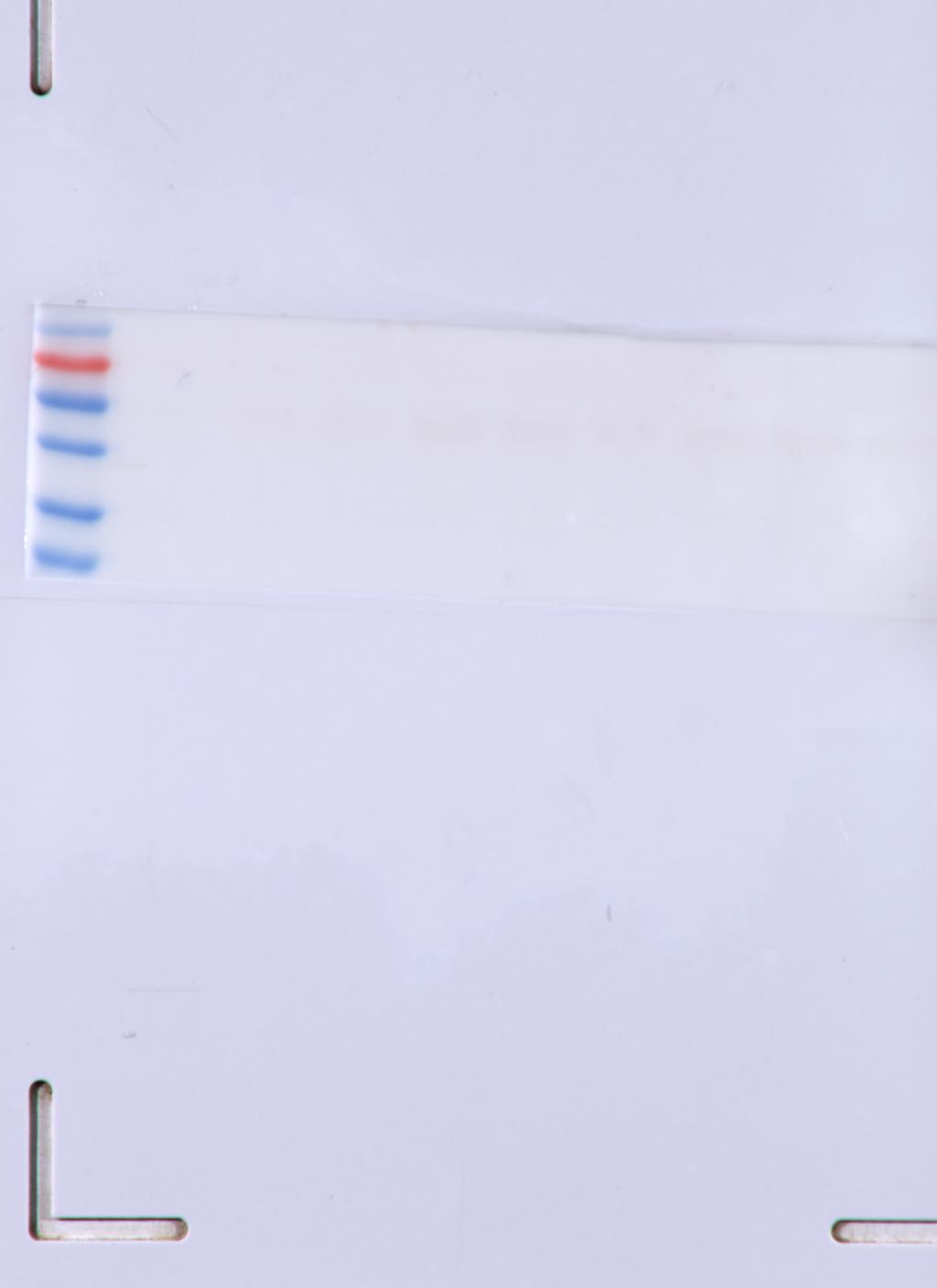

Supplement: Figure 4—source data 4. [file elife-108737-fig4-data4.zip › Figure 4—source data 4/α-myc-marker.jpg]

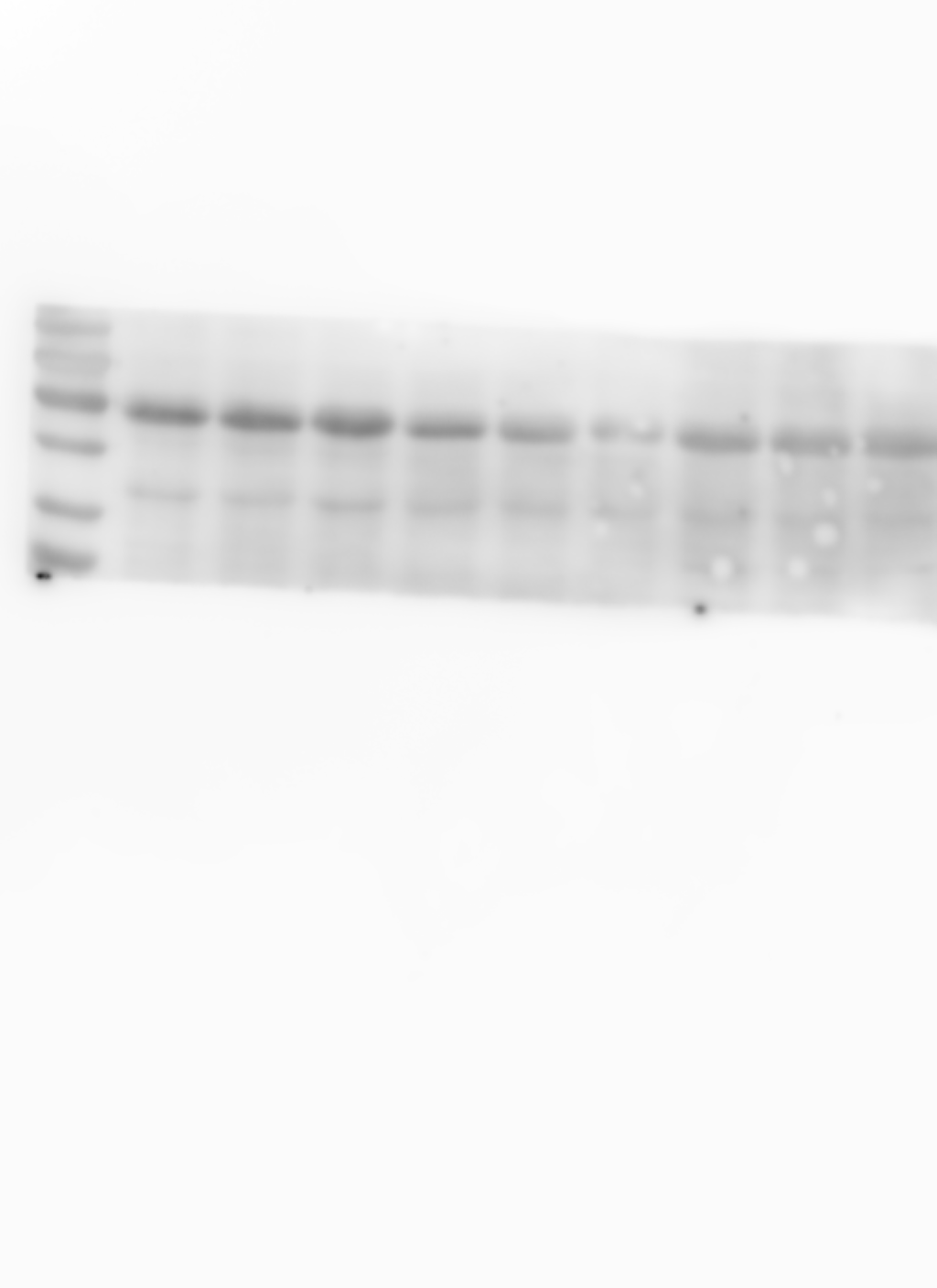

Supplement: Figure 4—source data 4. [file elife-108737-fig4-data4.zip › Figure 4—source data 4/α-myc-marker.tif]

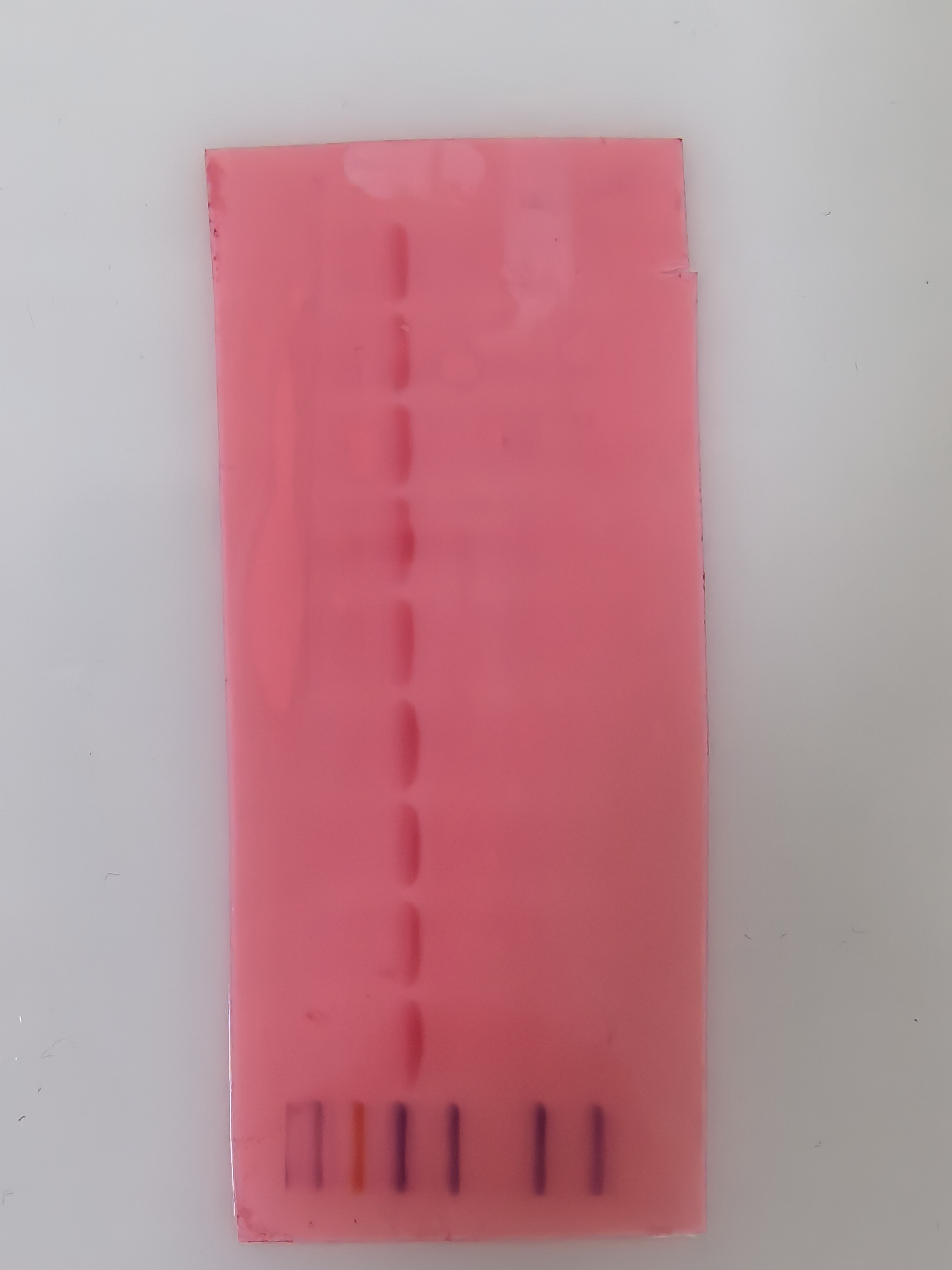

Supplement: Figure 4—source data 6. [file elife-108737-fig4-data6.zip › Figure 4—source data 6/RbCL.jpg]

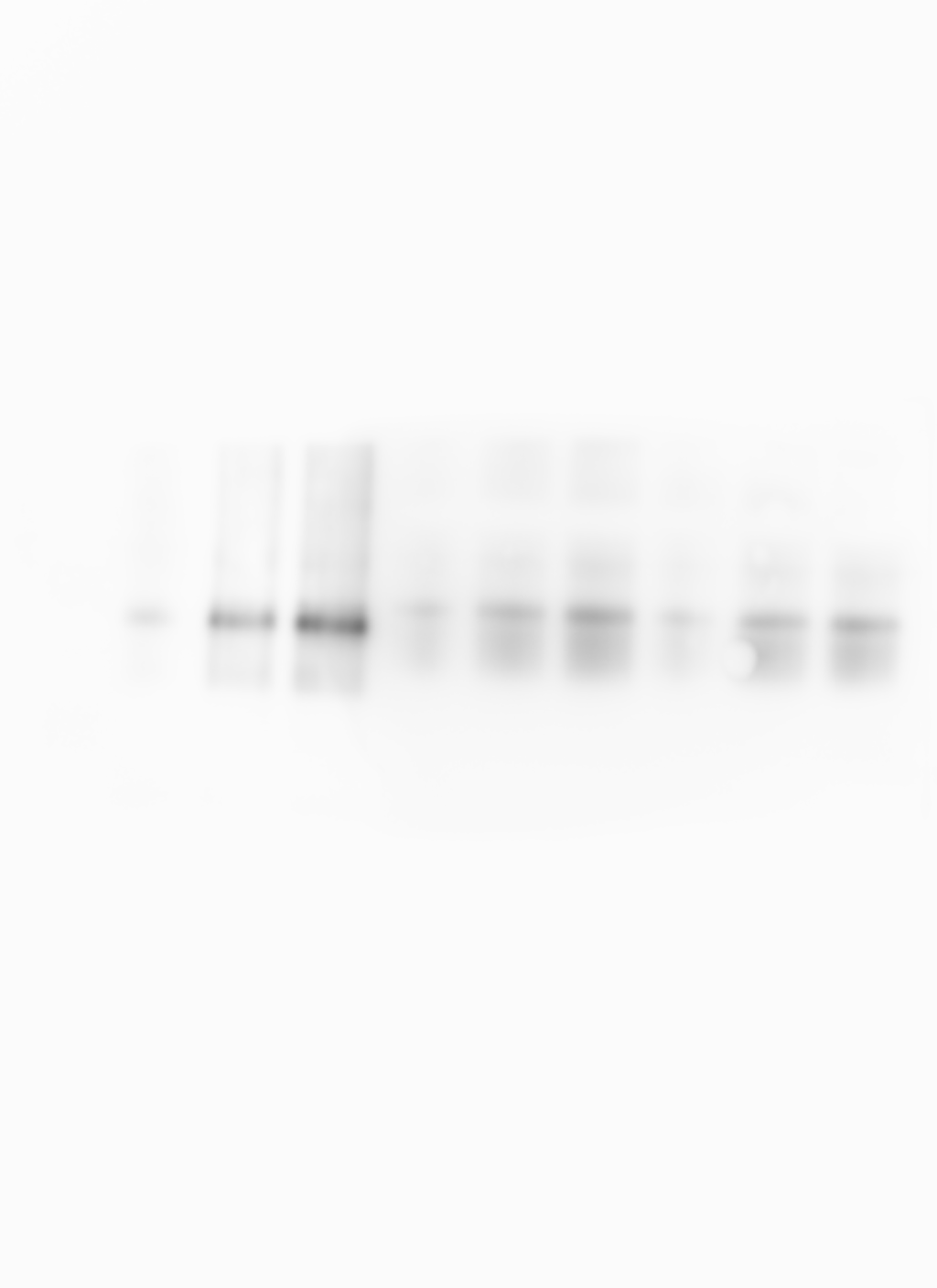

Supplement: Figure 4—source data 6. [file elife-108737-fig4-data6.zip › Figure 4—source data 6/α-flag-blot.tif]

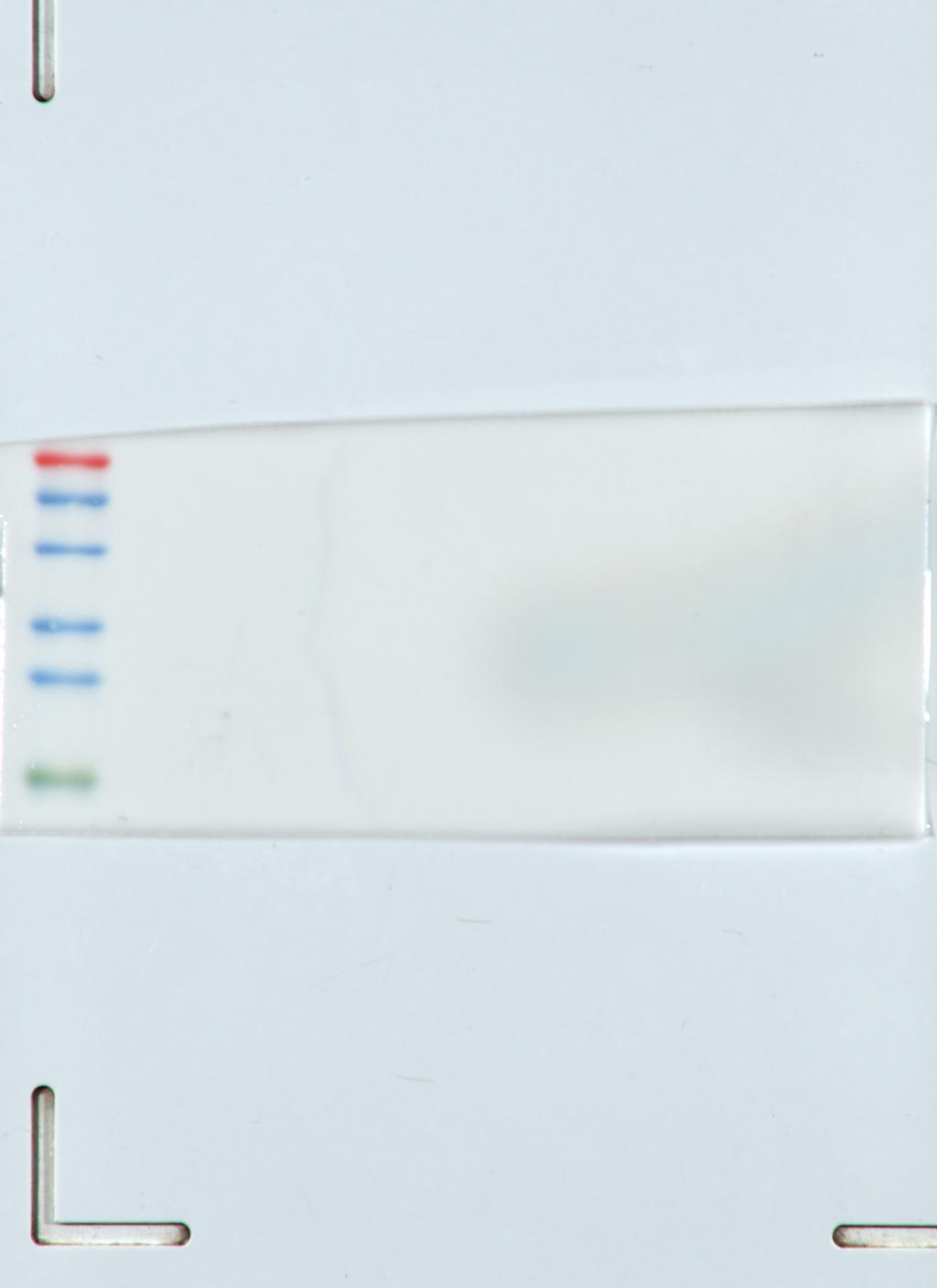

Supplement: Figure 4—source data 6. [file elife-108737-fig4-data6.zip › Figure 4—source data 6/α-flag-marker.jpg]

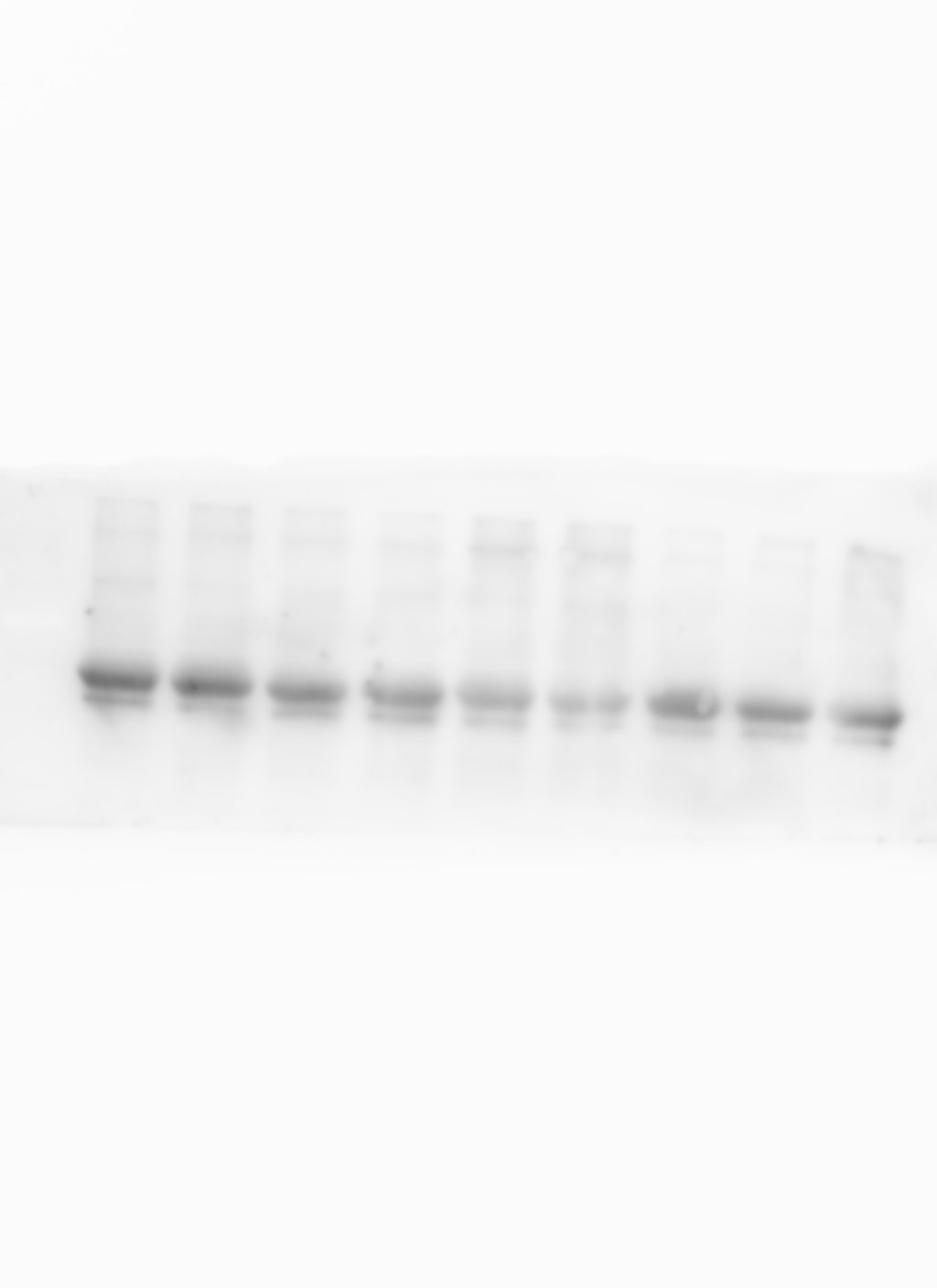

Supplement: Figure 4—source data 6. [file elife-108737-fig4-data6.zip › Figure 4—source data 6/α-myc-blot.tif]

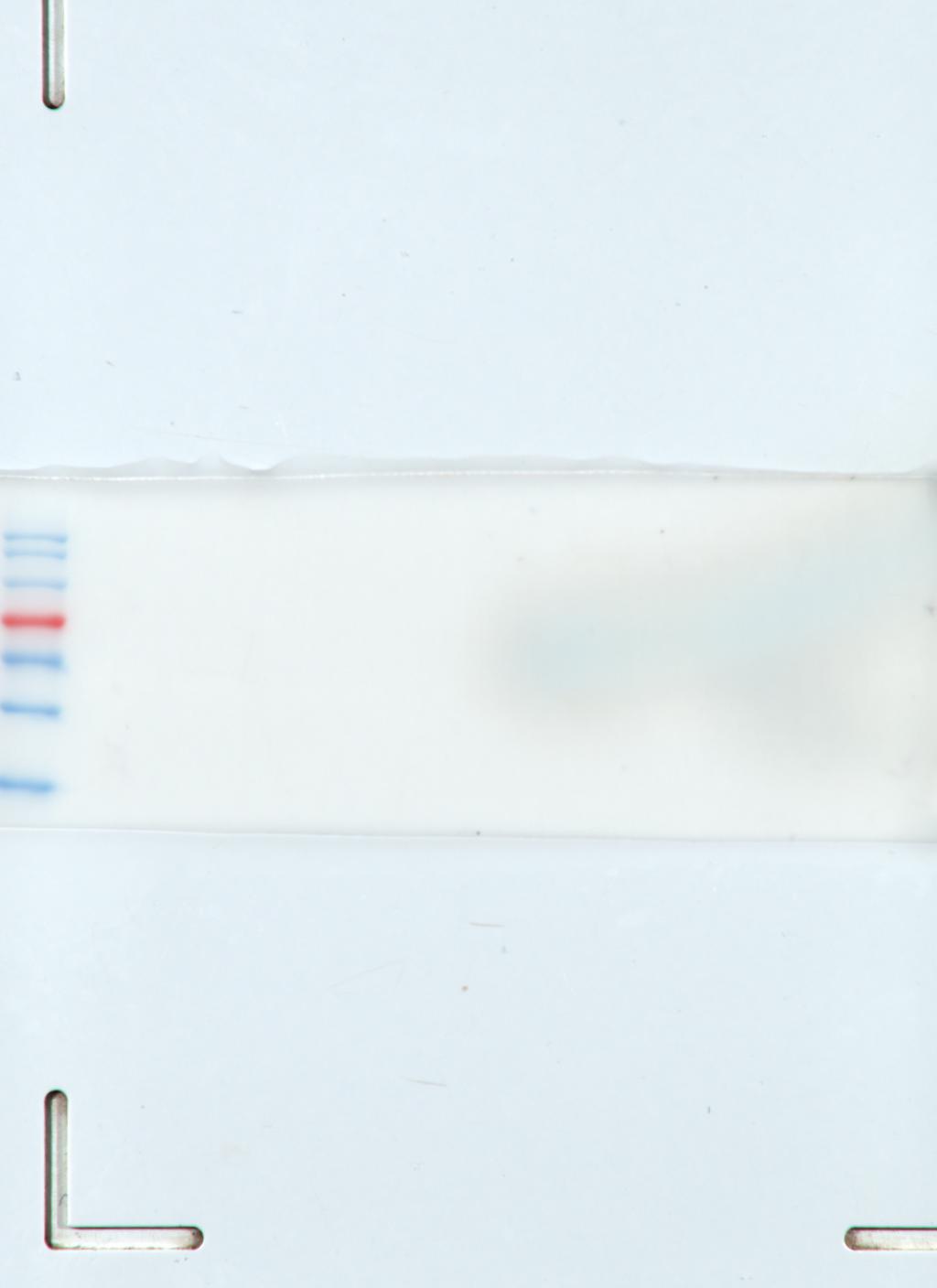

Supplement: Figure 4—source data 6. [file elife-108737-fig4-data6.zip › Figure 4—source data 6/α-myc-marker.jpg]

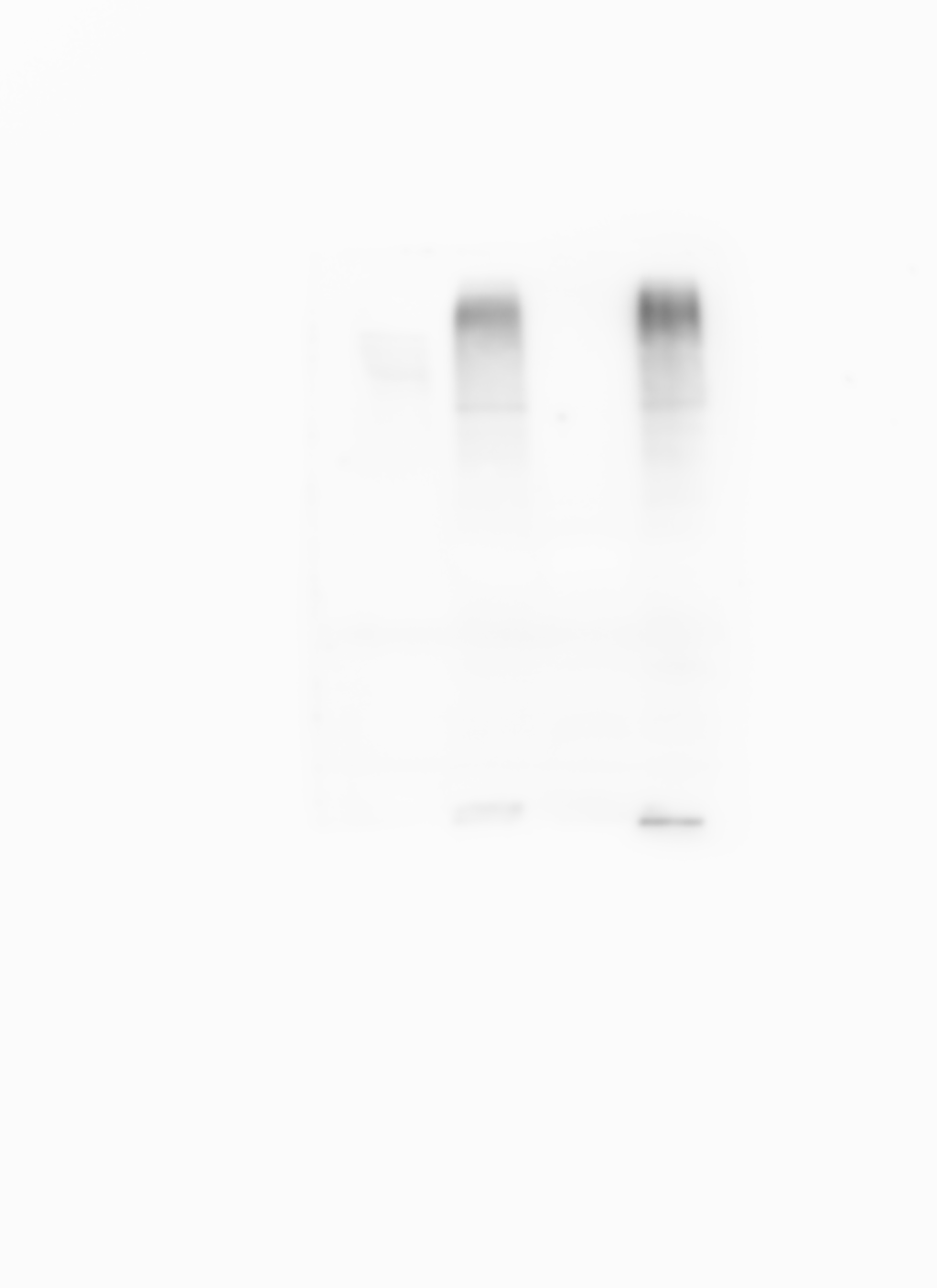

Supplement: Figure 4—source data 8. [file elife-108737-fig4-data8.zip › Figure 4—source data 8/input-HA-blot.tif]

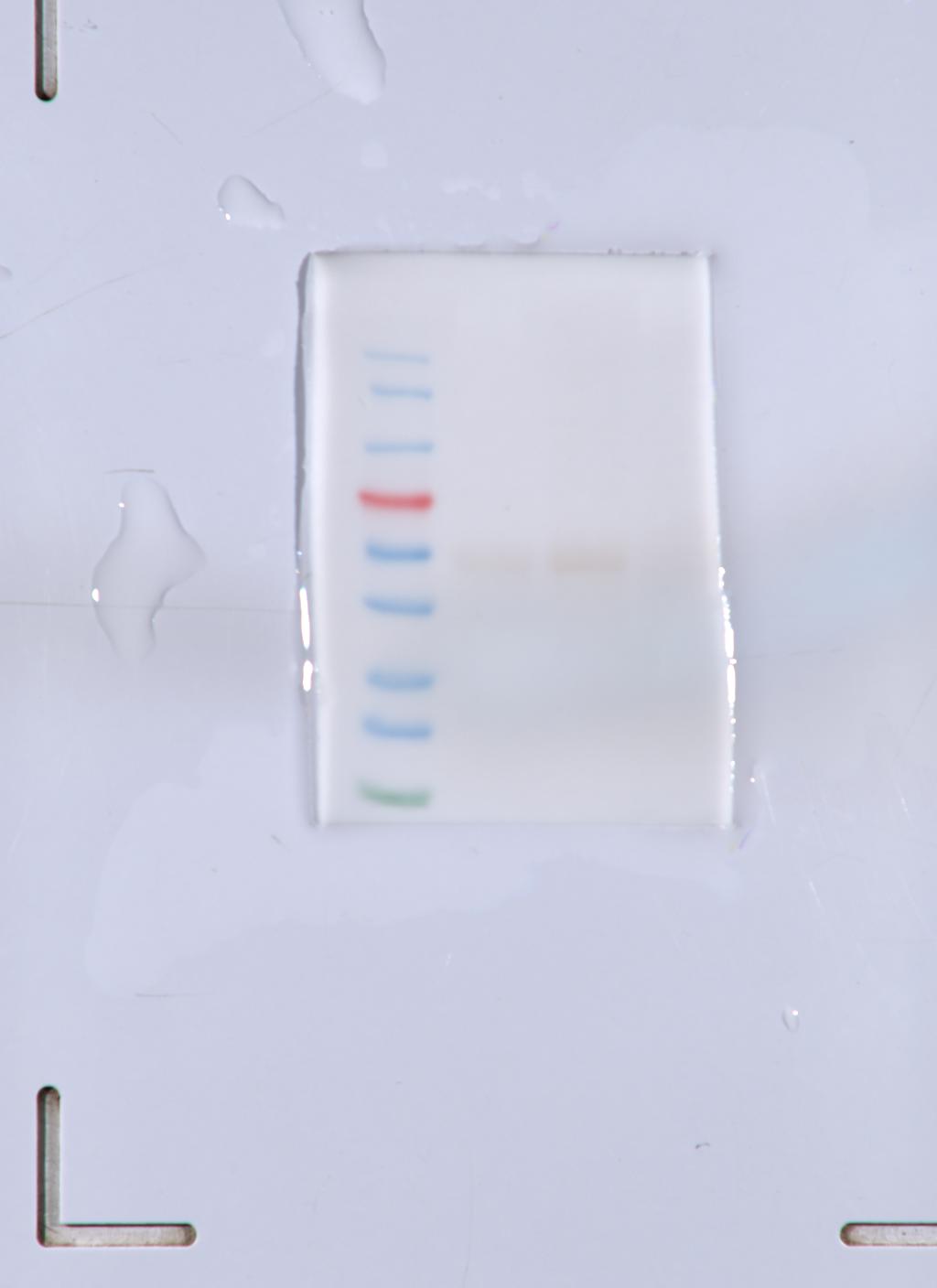

Supplement: Figure 4—source data 8. [file elife-108737-fig4-data8.zip › Figure 4—source data 8/input-HA-marker.jpg]

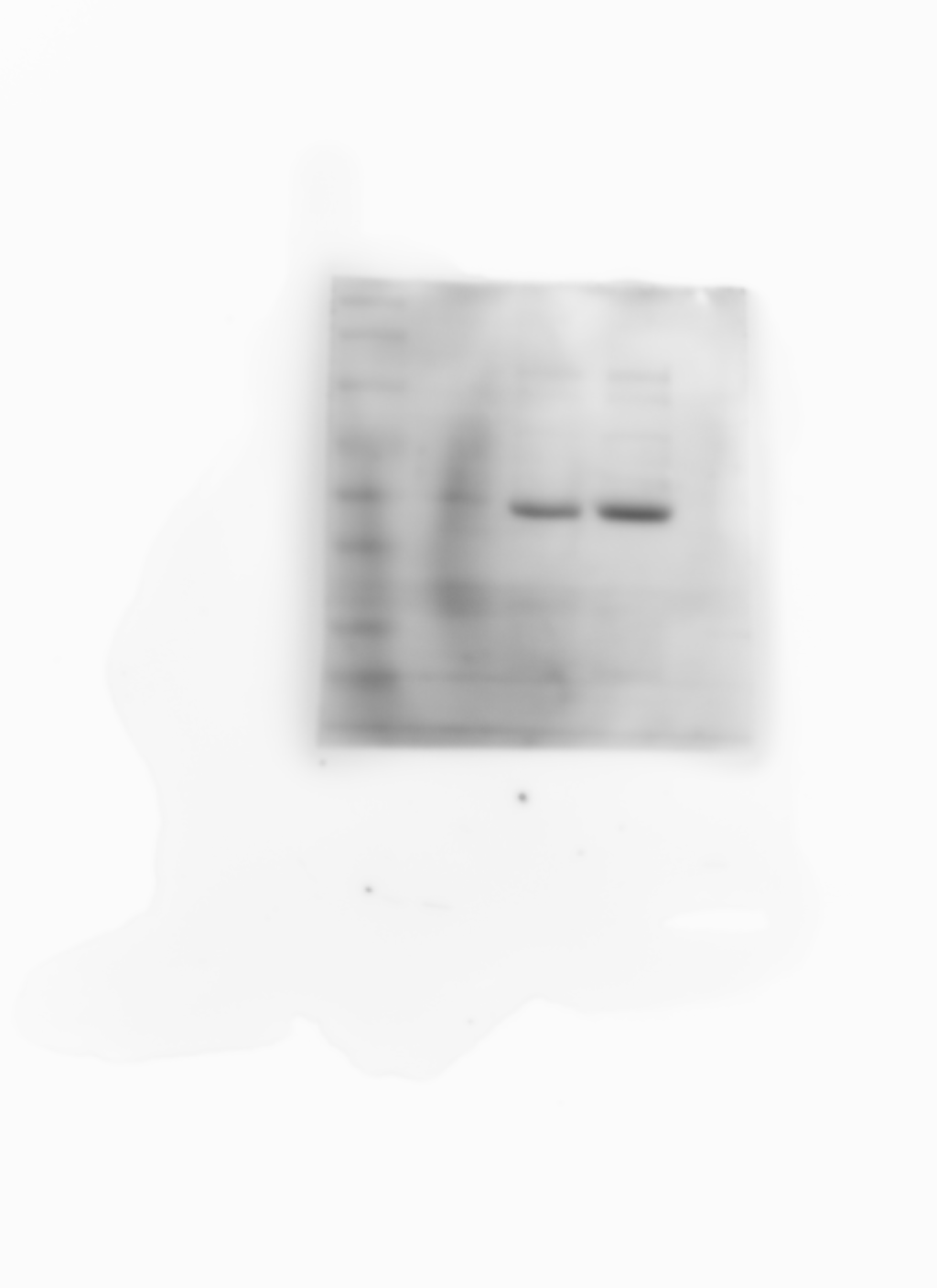

Supplement: Figure 4—source data 8. [file elife-108737-fig4-data8.zip › Figure 4—source data 8/input-myc-blot.tif]

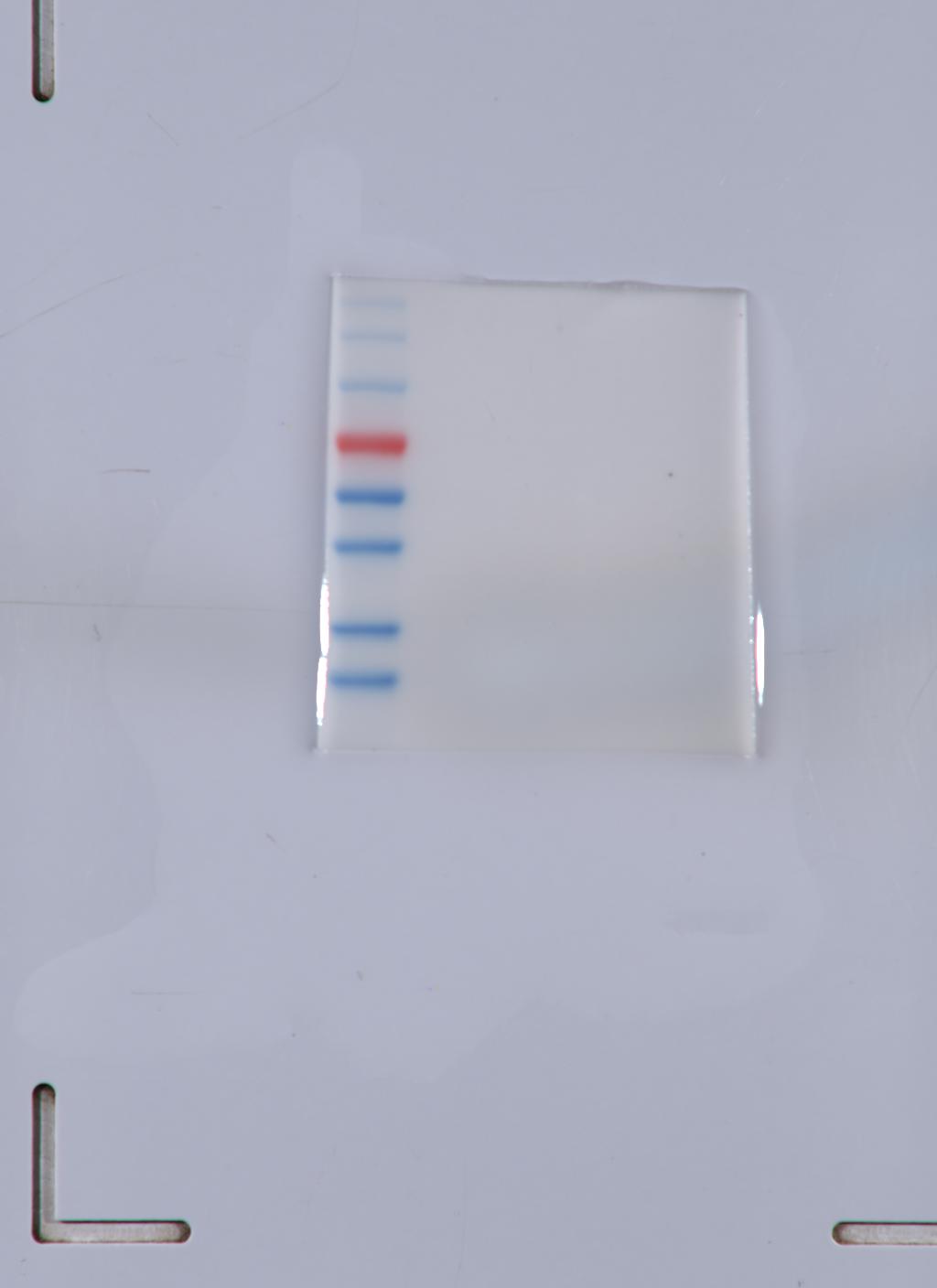

Supplement: Figure 4—source data 8. [file elife-108737-fig4-data8.zip › Figure 4—source data 8/input-myc-marker.jpg]

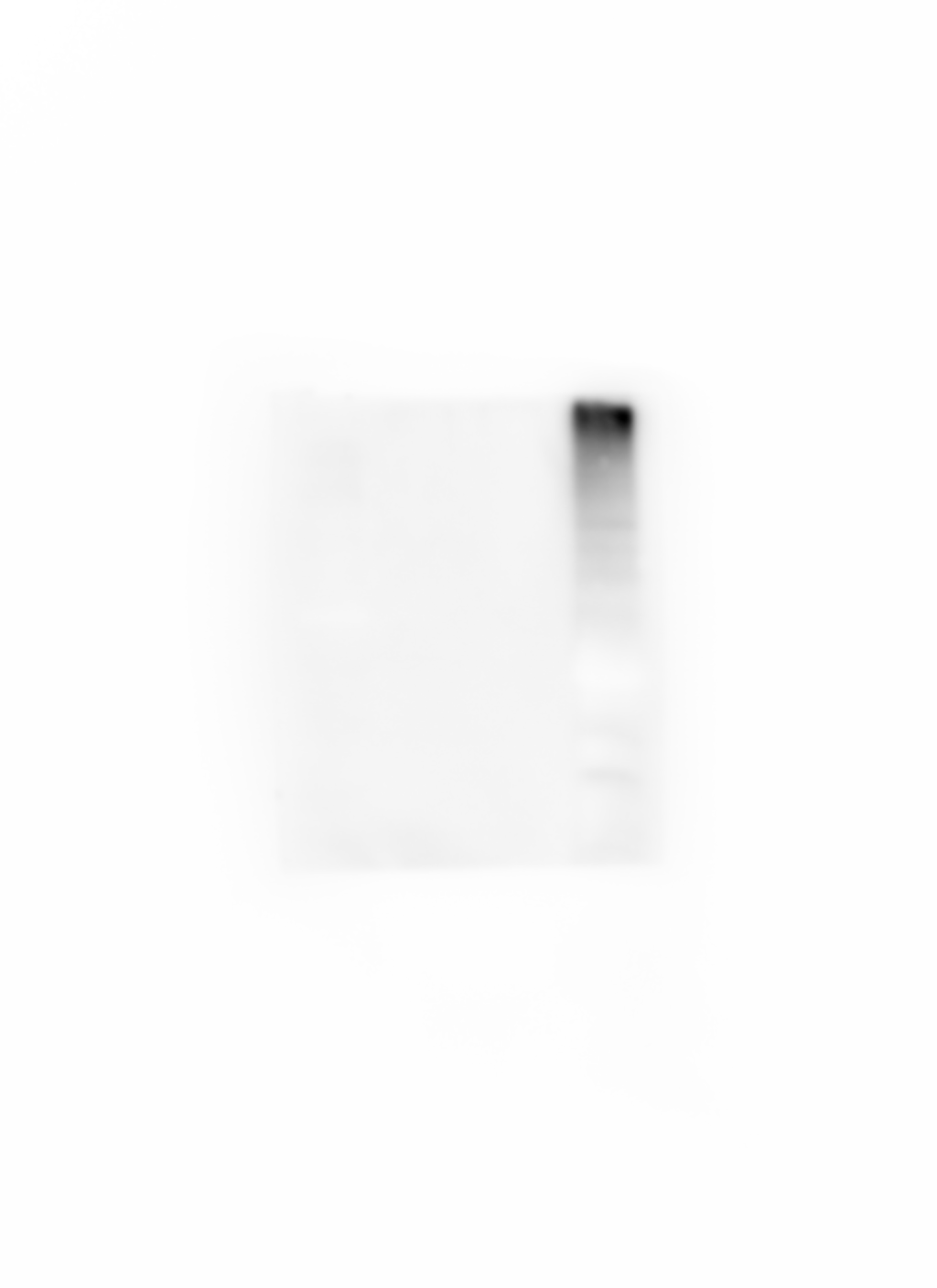

Supplement: Figure 4—source data 8. [file elife-108737-fig4-data8.zip › Figure 4—source data 8/IP-HA-blot.tif]

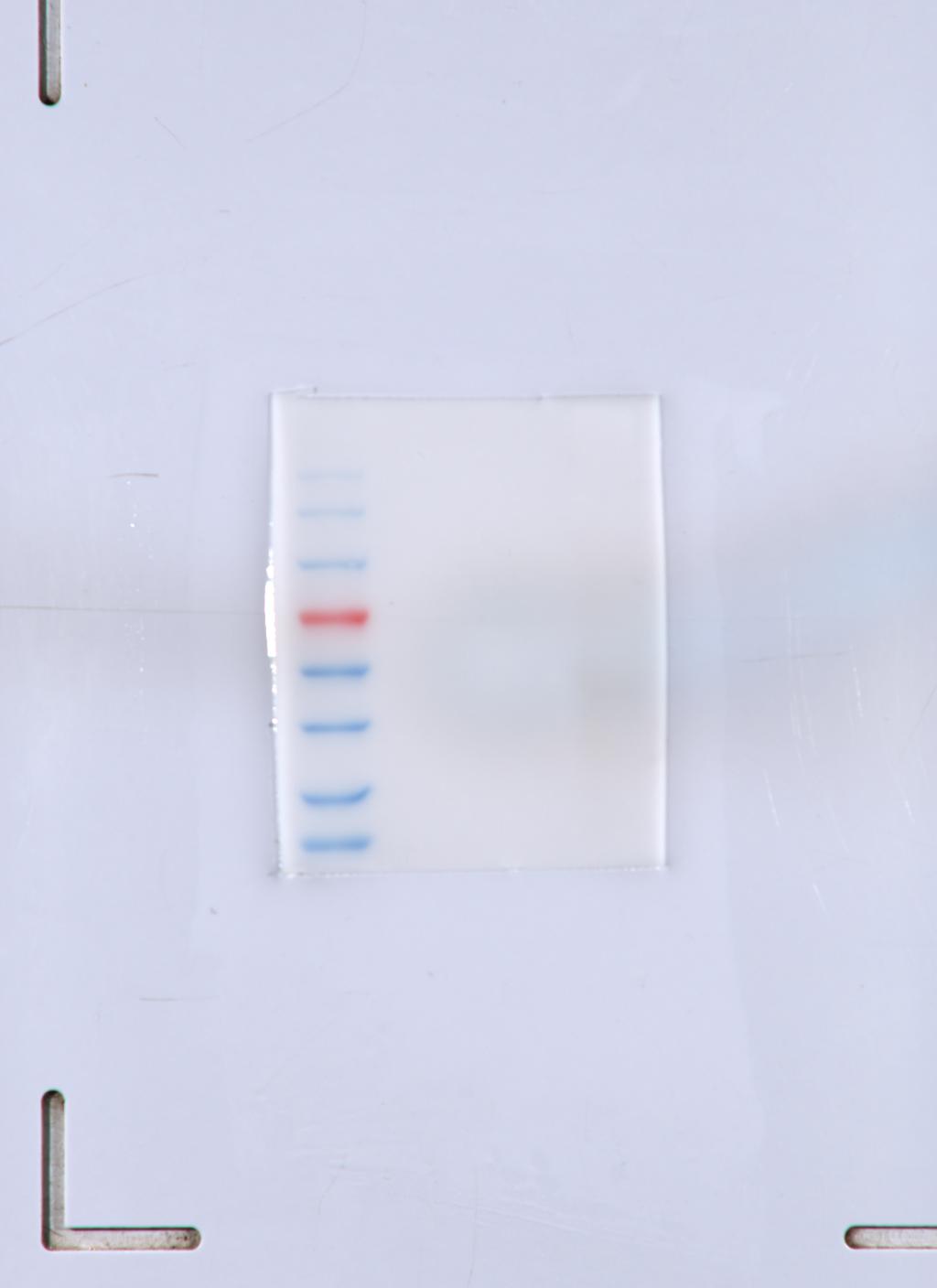

Supplement: Figure 4—source data 8. [file elife-108737-fig4-data8.zip › Figure 4—source data 8/IP-HA-marker.jpg]

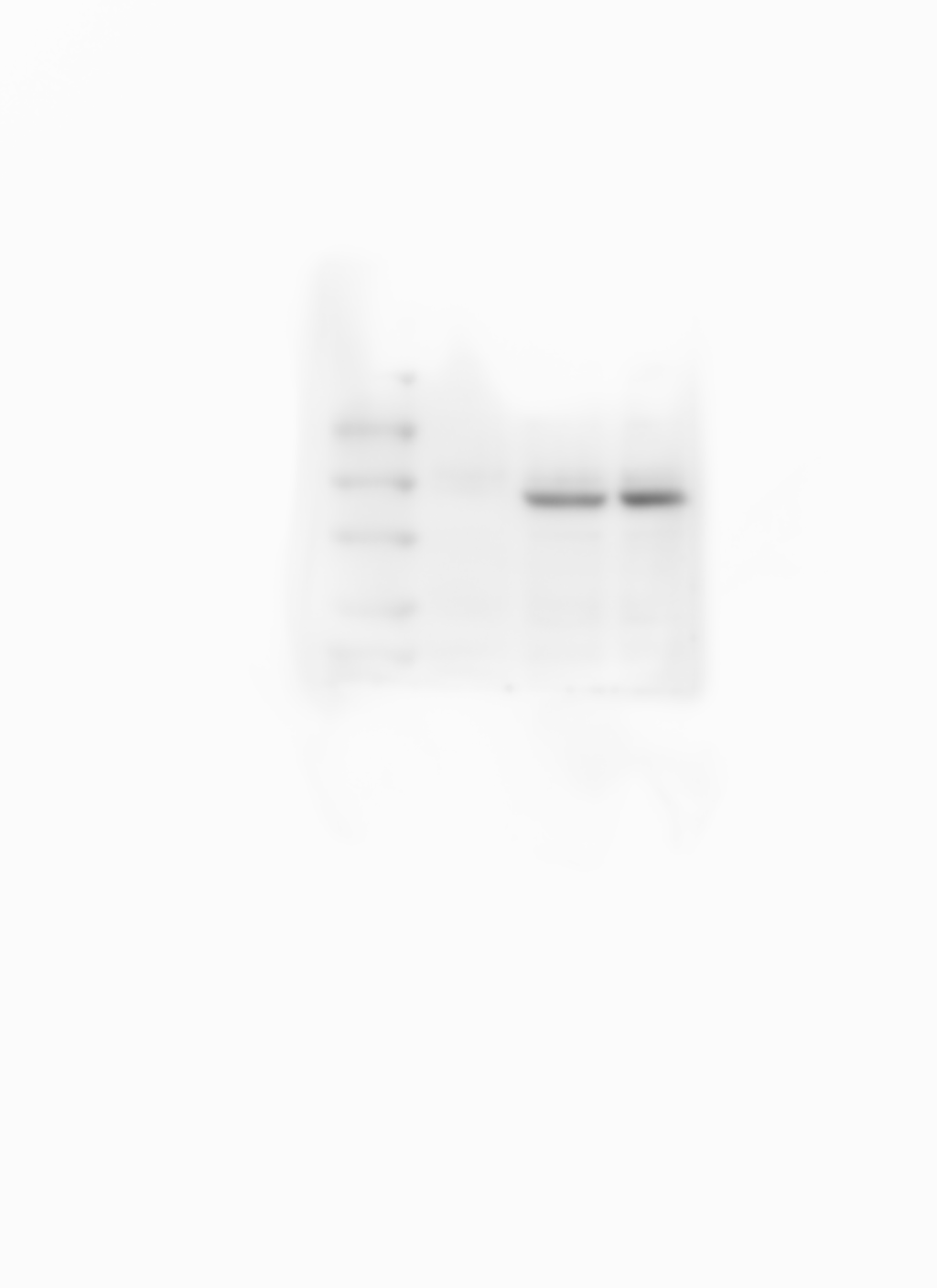

Supplement: Figure 4—source data 8. [file elife-108737-fig4-data8.zip › Figure 4—source data 8/IP-myc-blot.tif]

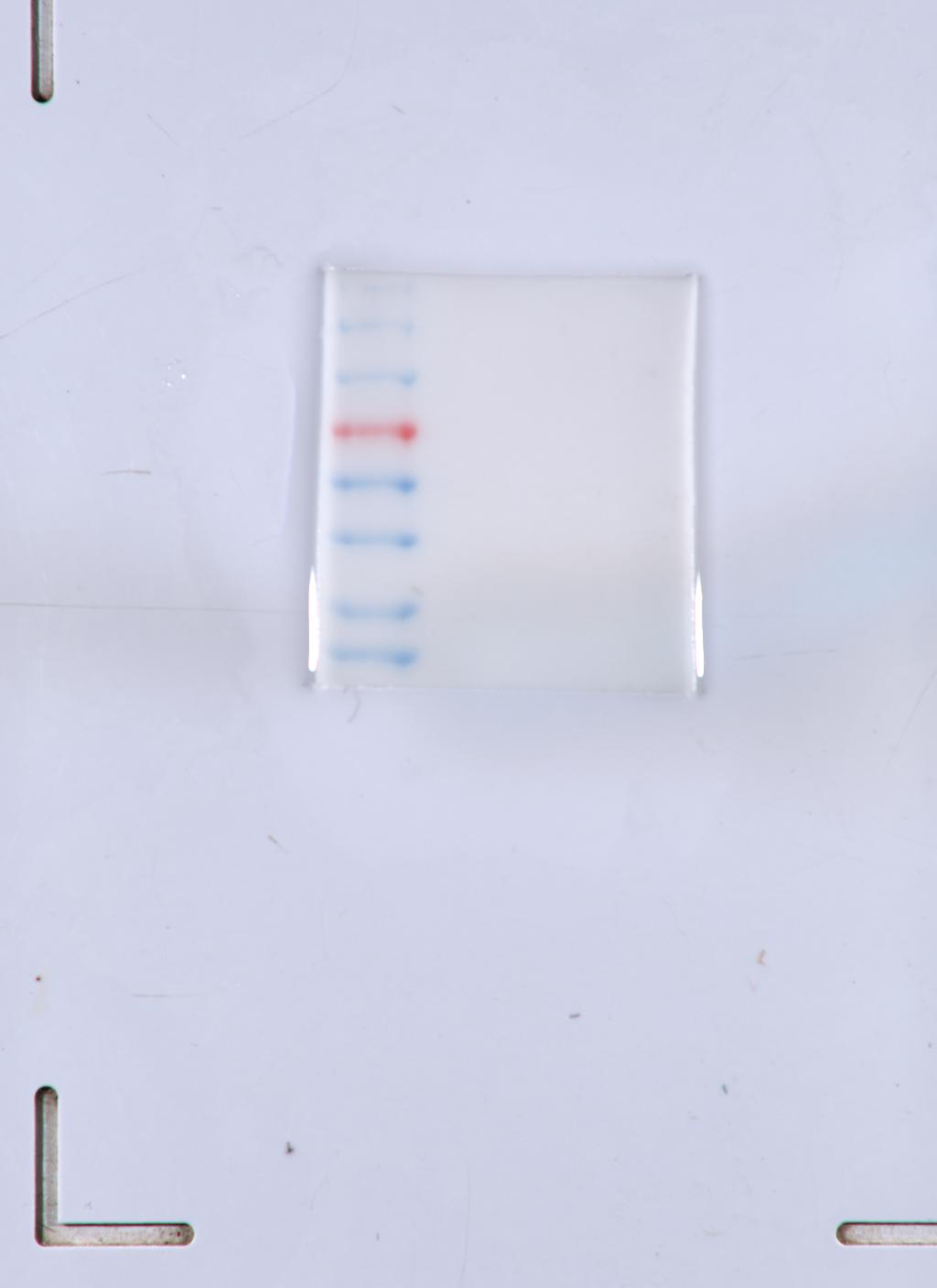

Supplement: Figure 4—source data 8. [file elife-108737-fig4-data8.zip › Figure 4—source data 8/IP-myc-marker.jpg]

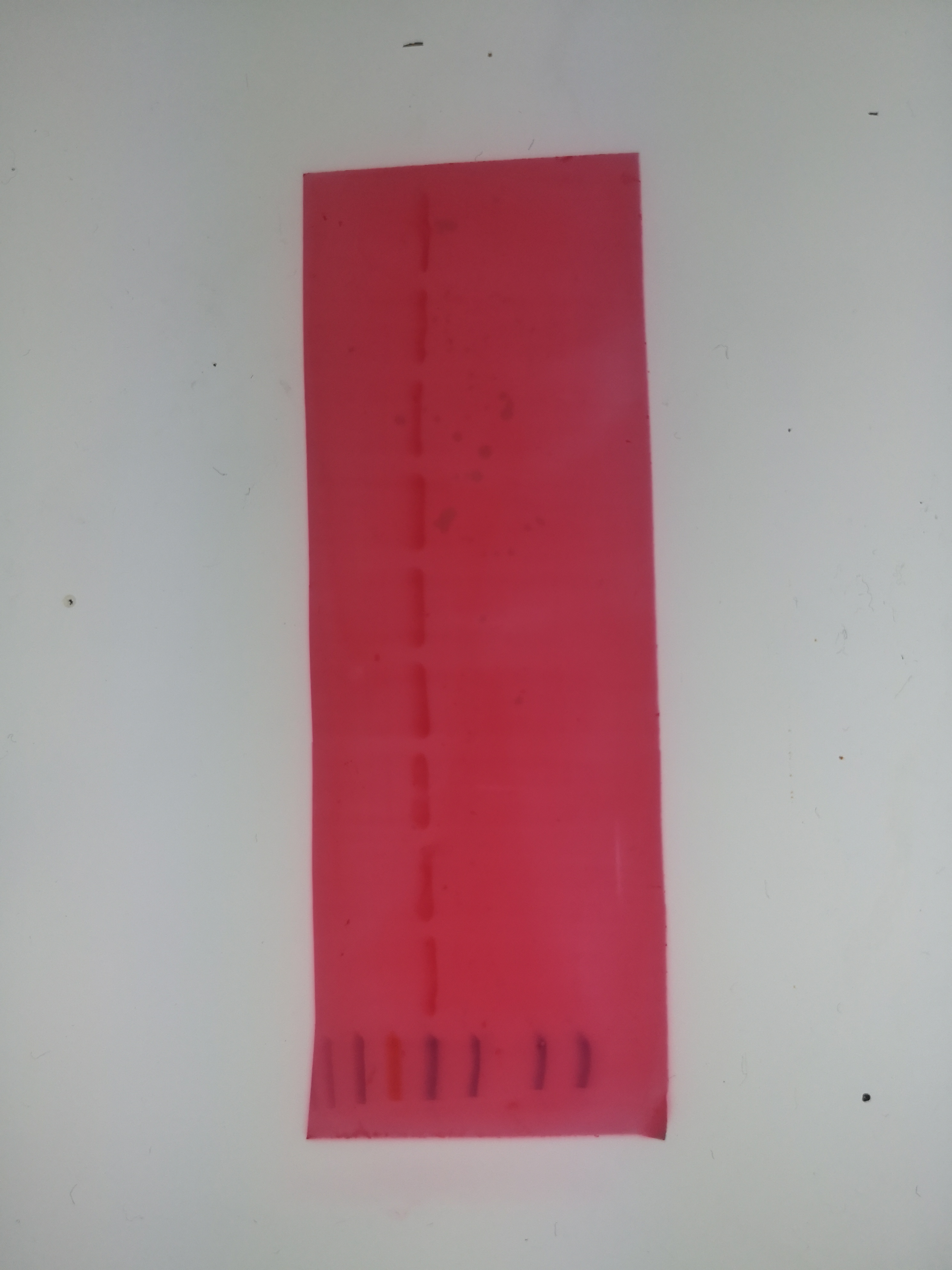

Supplement: Figure 4—figure supplement 4—source data 2. [file elife-108737-fig4-figsupp4-data2.zip › Figure 4—figure supplement 4—source data 2/RbCL.jpg]

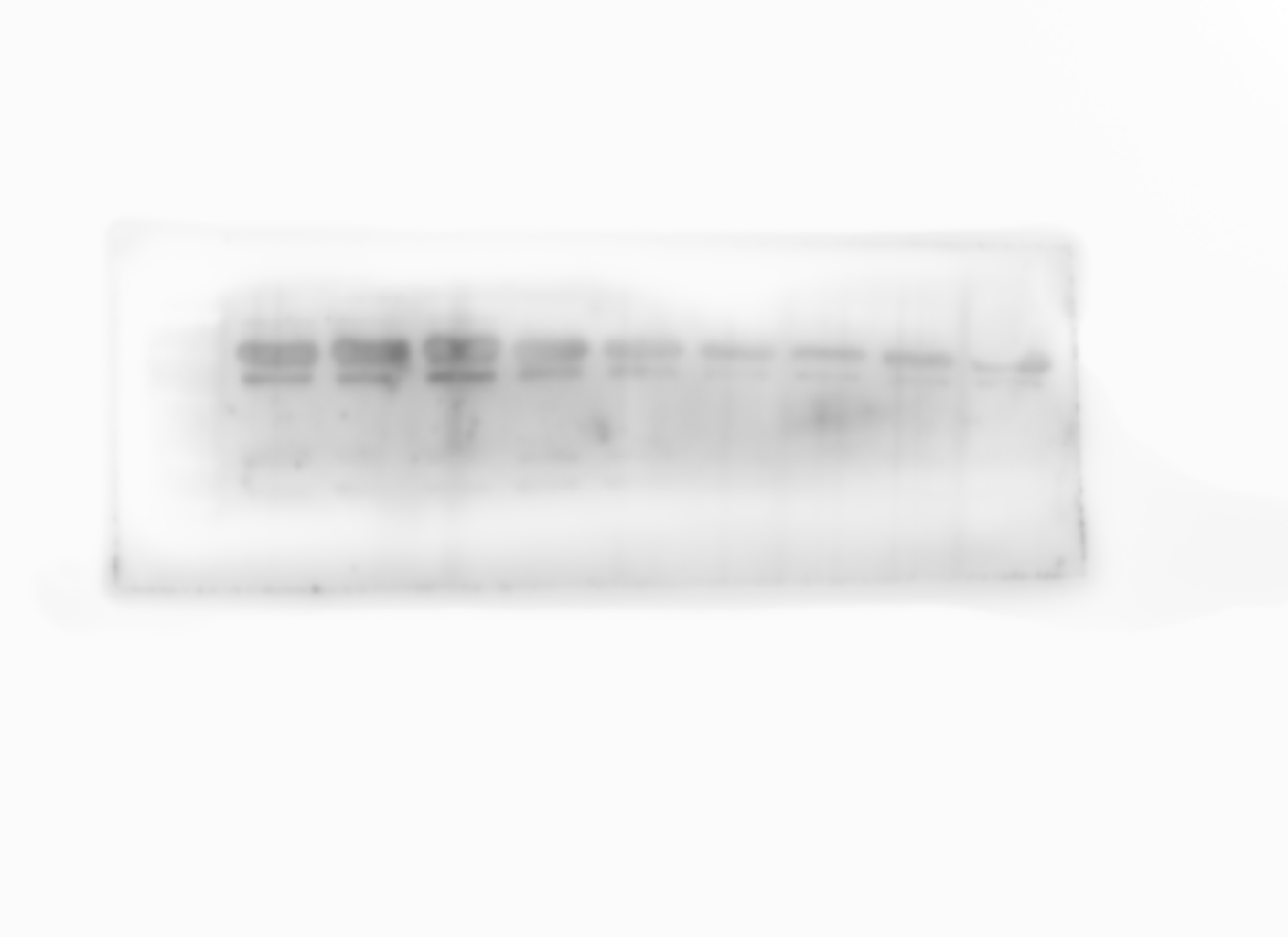

Supplement: Figure 4—figure supplement 4—source data 2. [file elife-108737-fig4-figsupp4-data2.zip › Figure 4—figure supplement 4—source data 2/α-NtRLP4-blot.tif]

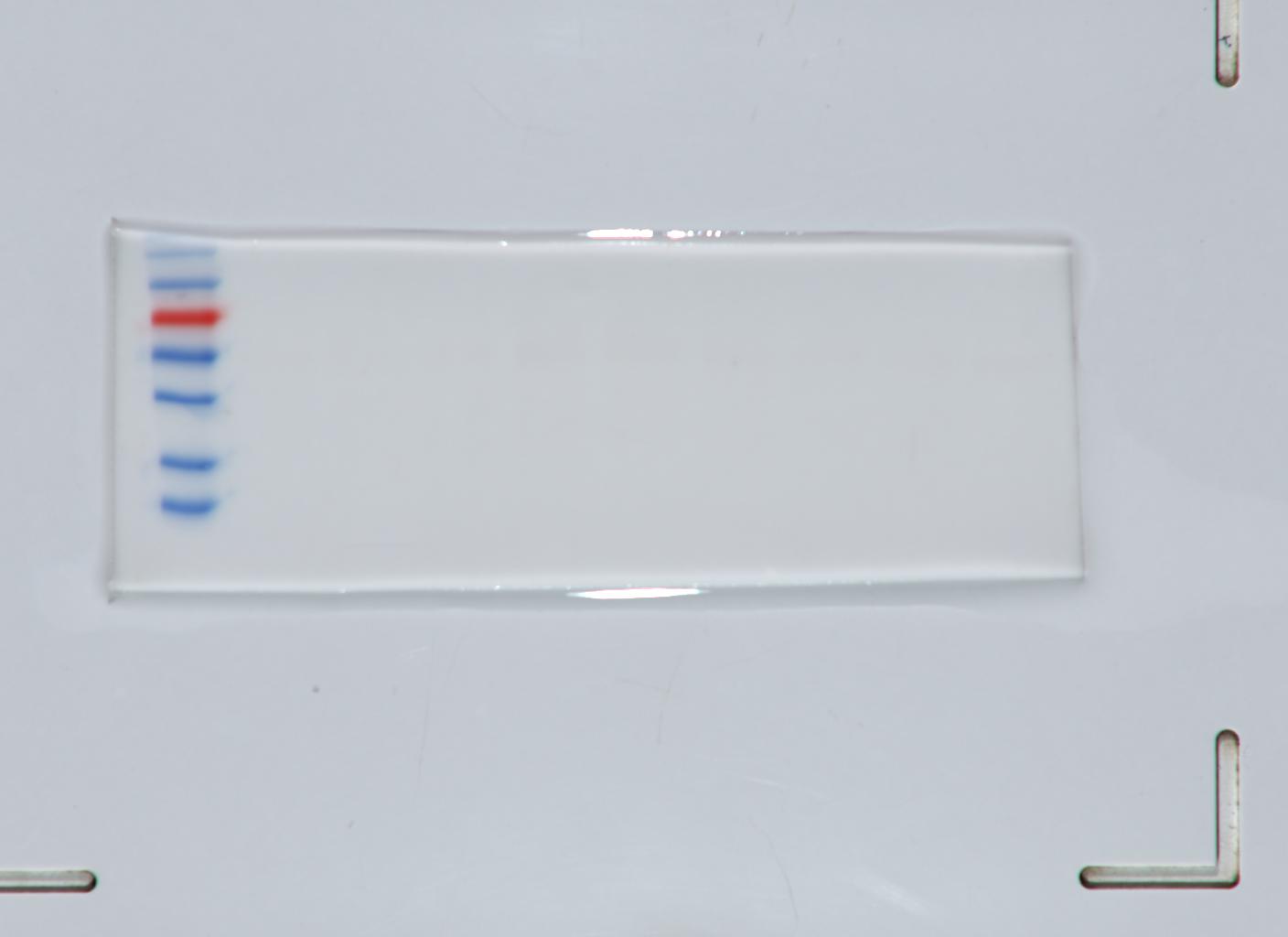

Supplement: Figure 4—figure supplement 4—source data 2. [file elife-108737-fig4-figsupp4-data2.zip › Figure 4—figure supplement 4—source data 2/α-NtRLP4-marker.jpg]

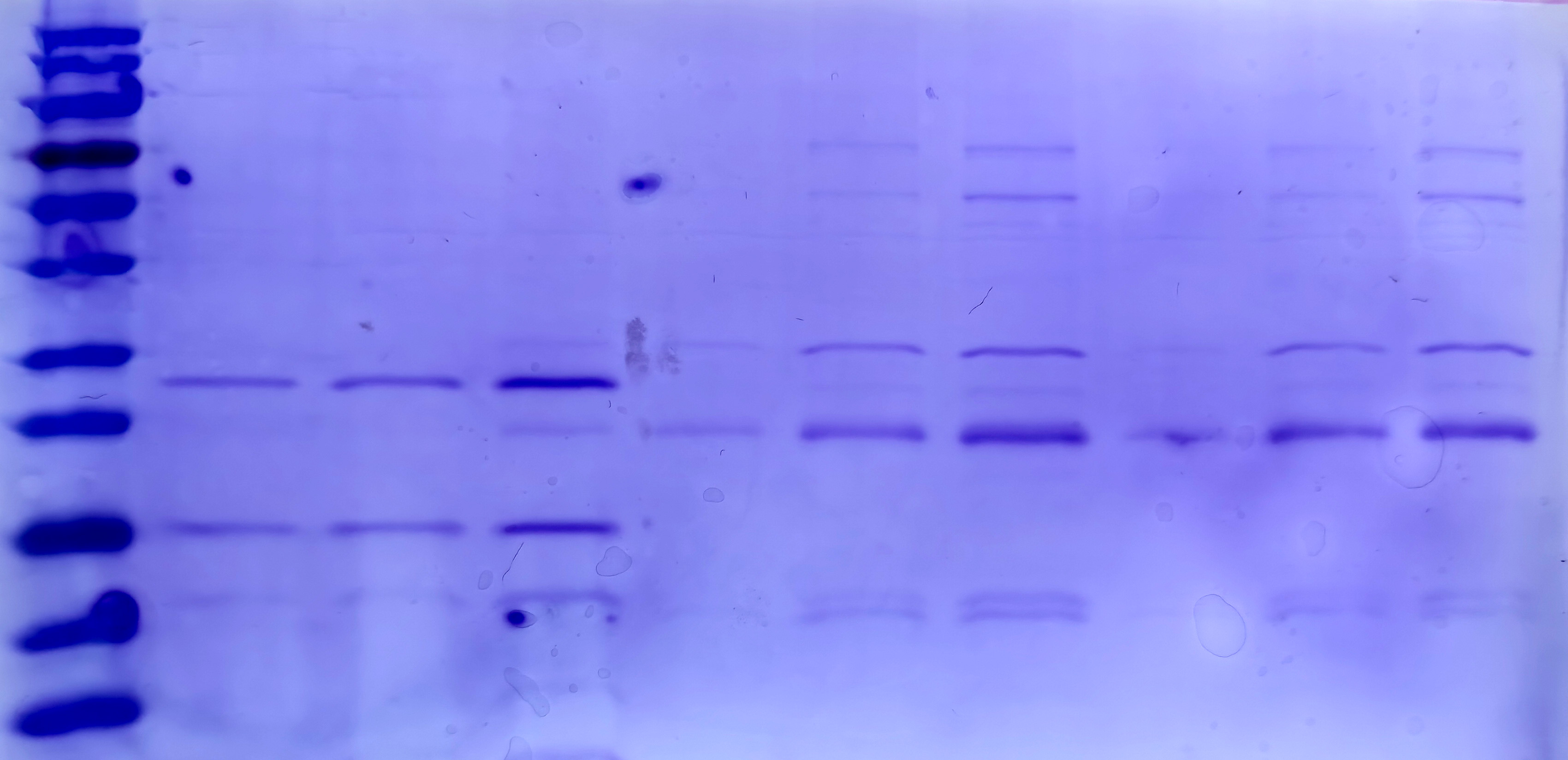

Supplement: Figure 4—figure supplement 7—source data 2. [file elife-108737-fig4-figsupp7-data2.zip › Figure 4—figure supplement 7—source data 2/CCB.jpg]

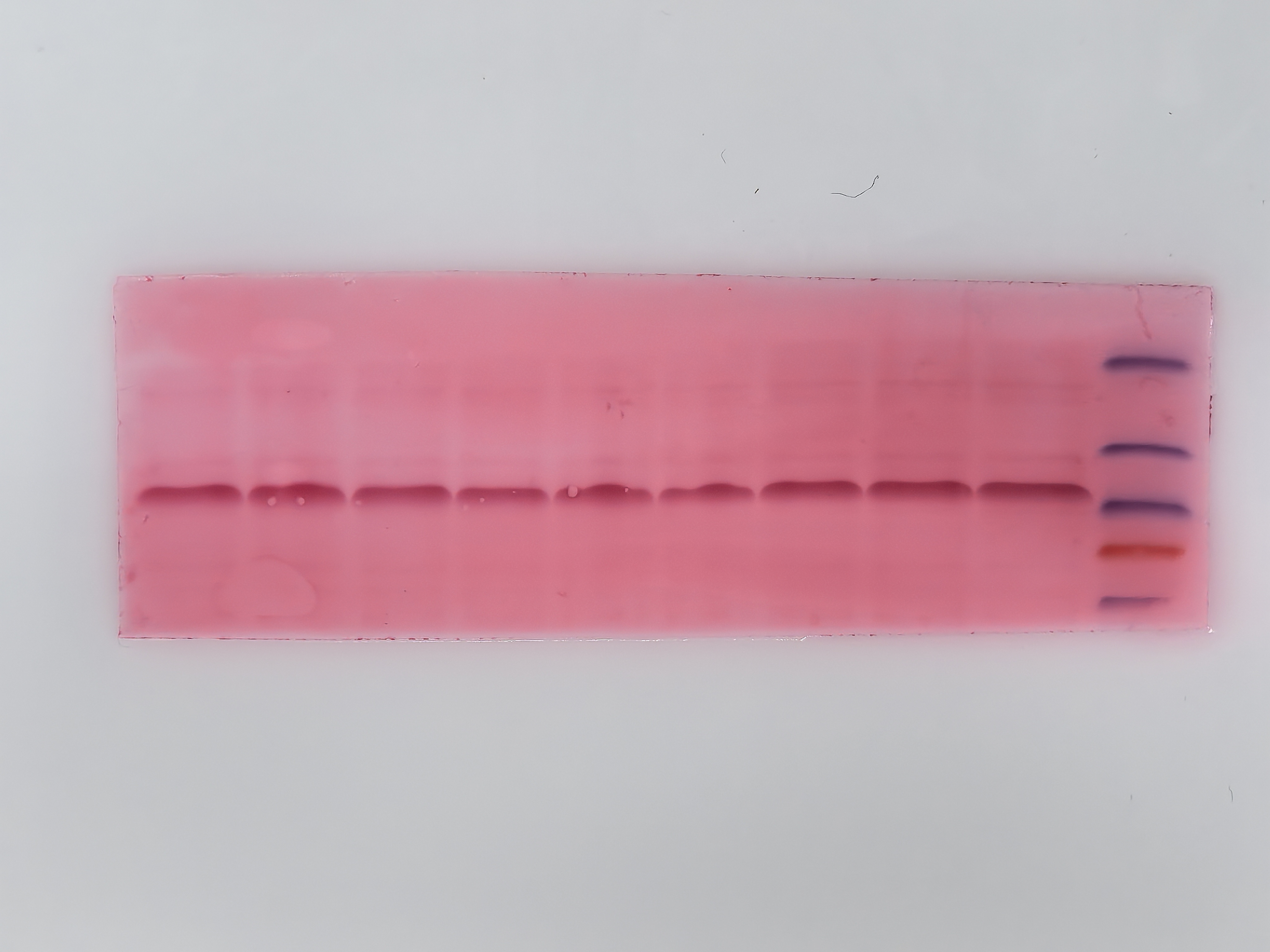

Supplement: Figure 4—figure supplement 7—source data 2. [file elife-108737-fig4-figsupp7-data2.zip › Figure 4—figure supplement 7—source data 2/RbCL.jpg]

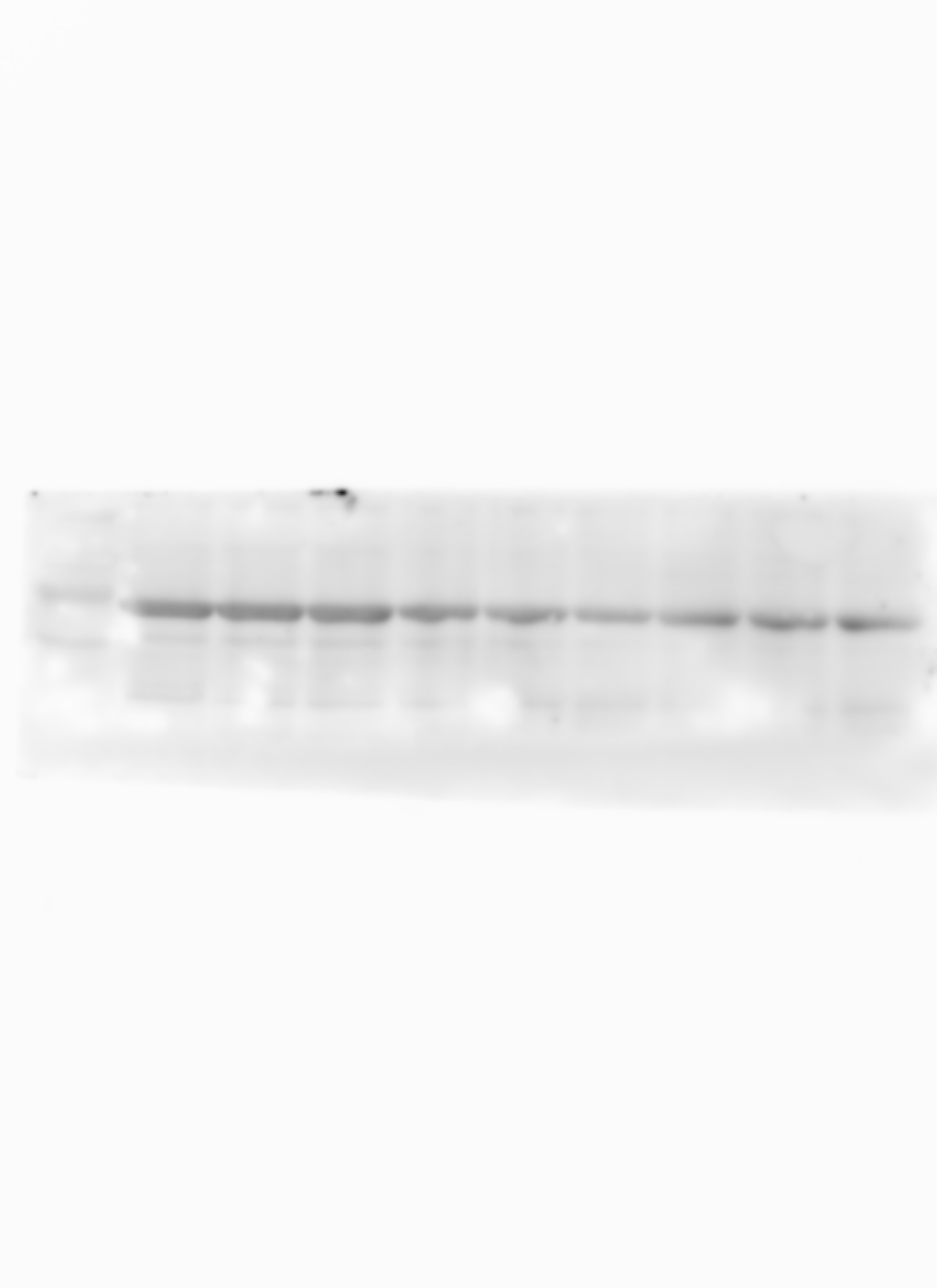

Supplement: Figure 4—figure supplement 7—source data 2. [file elife-108737-fig4-figsupp7-data2.zip › Figure 4—figure supplement 7—source data 2/α-myc-blot.tif]

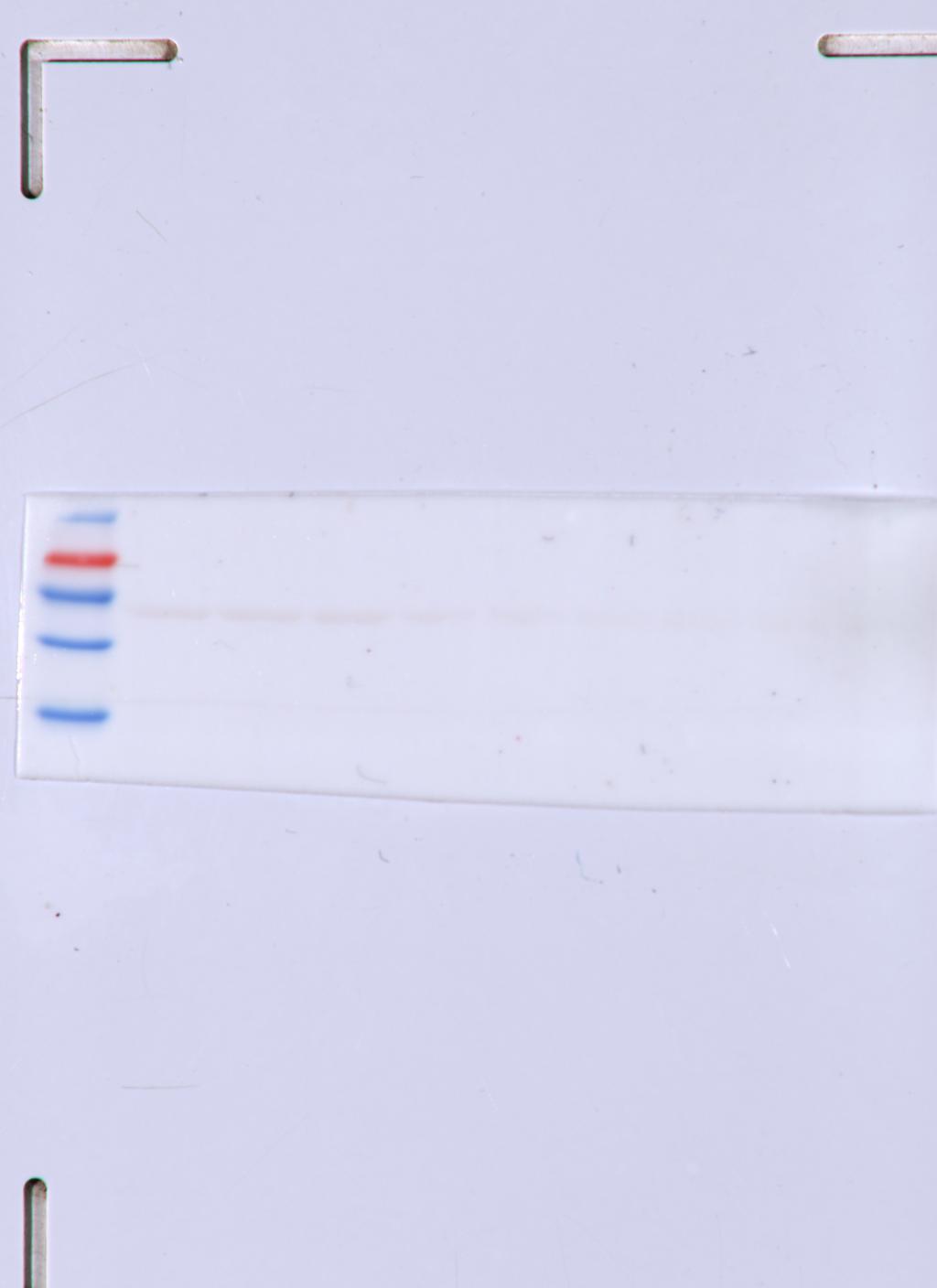

Supplement: Figure 4—figure supplement 7—source data 2. [file elife-108737-fig4-figsupp7-data2.zip › Figure 4—figure supplement 7—source data 2/α-myc-marker.jpg]

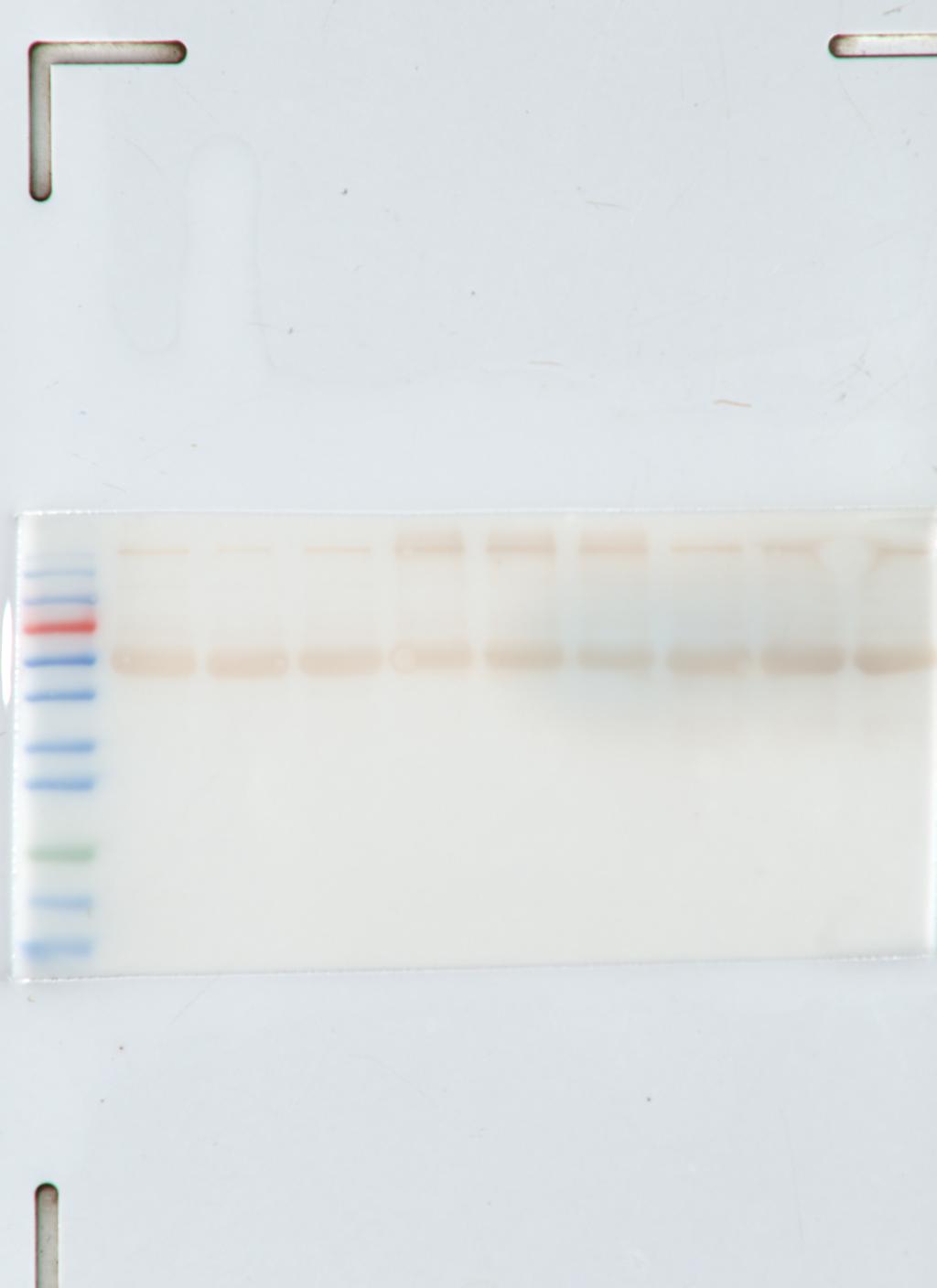

Supplement: Figure 4—figure supplement 8—source data 2. [file elife-108737-fig4-figsupp8-data2.zip › Figure 4—figure supplement 8—source data 2/flag.jpg]
