# Supplementary material for: Herbivorous insects independently evolved salivary effectors to regulate plant immunity by destabilizing the malectin-LRR RLP NtRLP4
Source: eLife. 2026 May 5;14:RP108737. doi: 10.7554/eLife.108737 (PMC13143284; doi:10.7554/eLife.108737)
Supplement: Figure 1—source data 3. [file elife-108737-fig1-data3.zip › Figure 1—source data 3.pptx]

## Slide 1
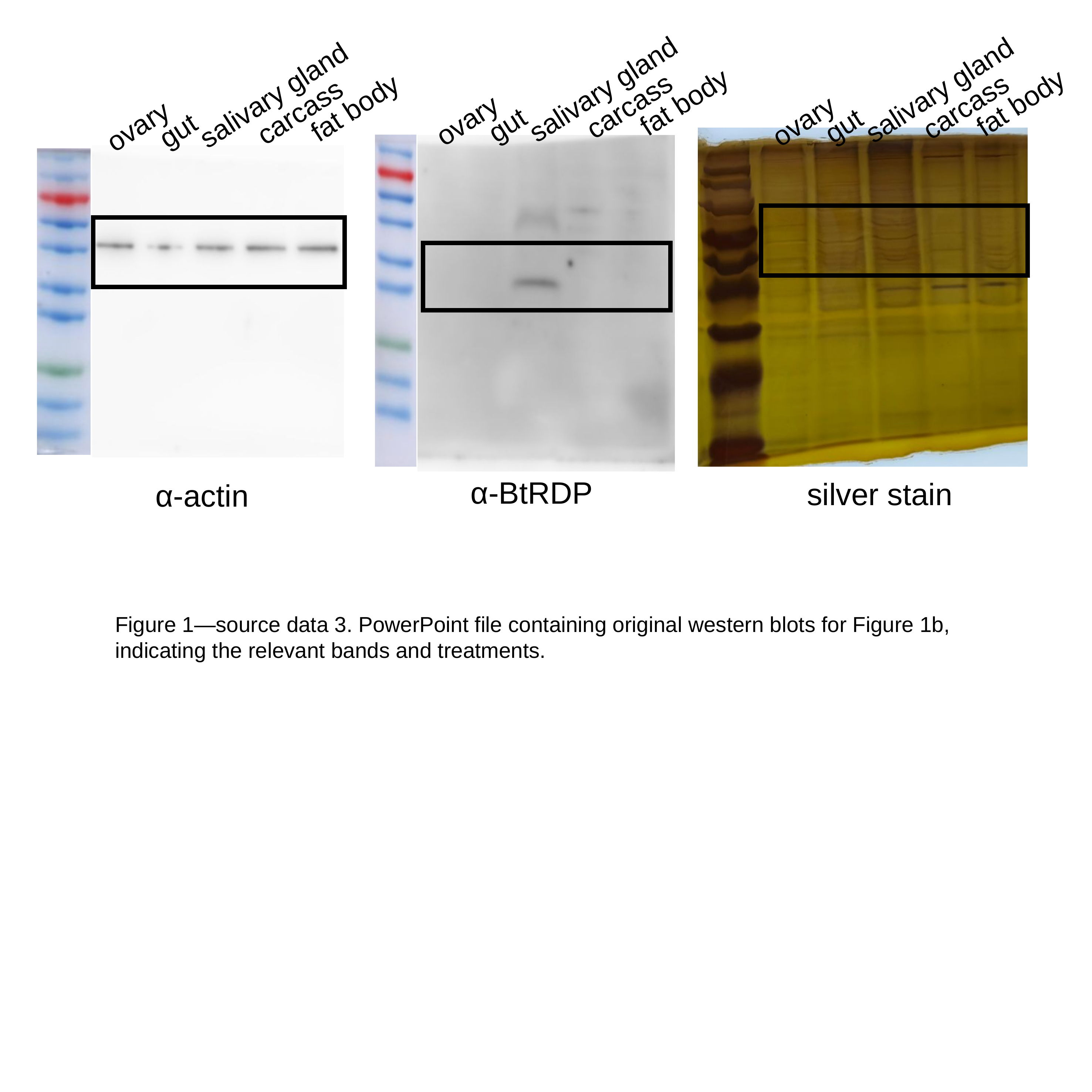

salivary gland
salivary gland
salivary gland
fat body
fat body
carcass
carcass
fat body
gut
gut
carcass
ovary
ovary
gut
ovary
α-BtRDP
silver stain
α-actin
Figure 1—source data 3. PowerPoint file containing original western blots for Figure 1b, indicating the relevant bands and treatments.
