# Supplementary material for: Herbivorous insects independently evolved salivary effectors to regulate plant immunity by destabilizing the malectin-LRR RLP NtRLP4
Source: eLife. 2026 May 5;14:RP108737. doi: 10.7554/eLife.108737 (PMC13143284; doi:10.7554/eLife.108737)
Supplement: Figure 3—source data 1. [file elife-108737-fig3-data1.zip › Figure 3—source data 1.pptx]

## Slide 1
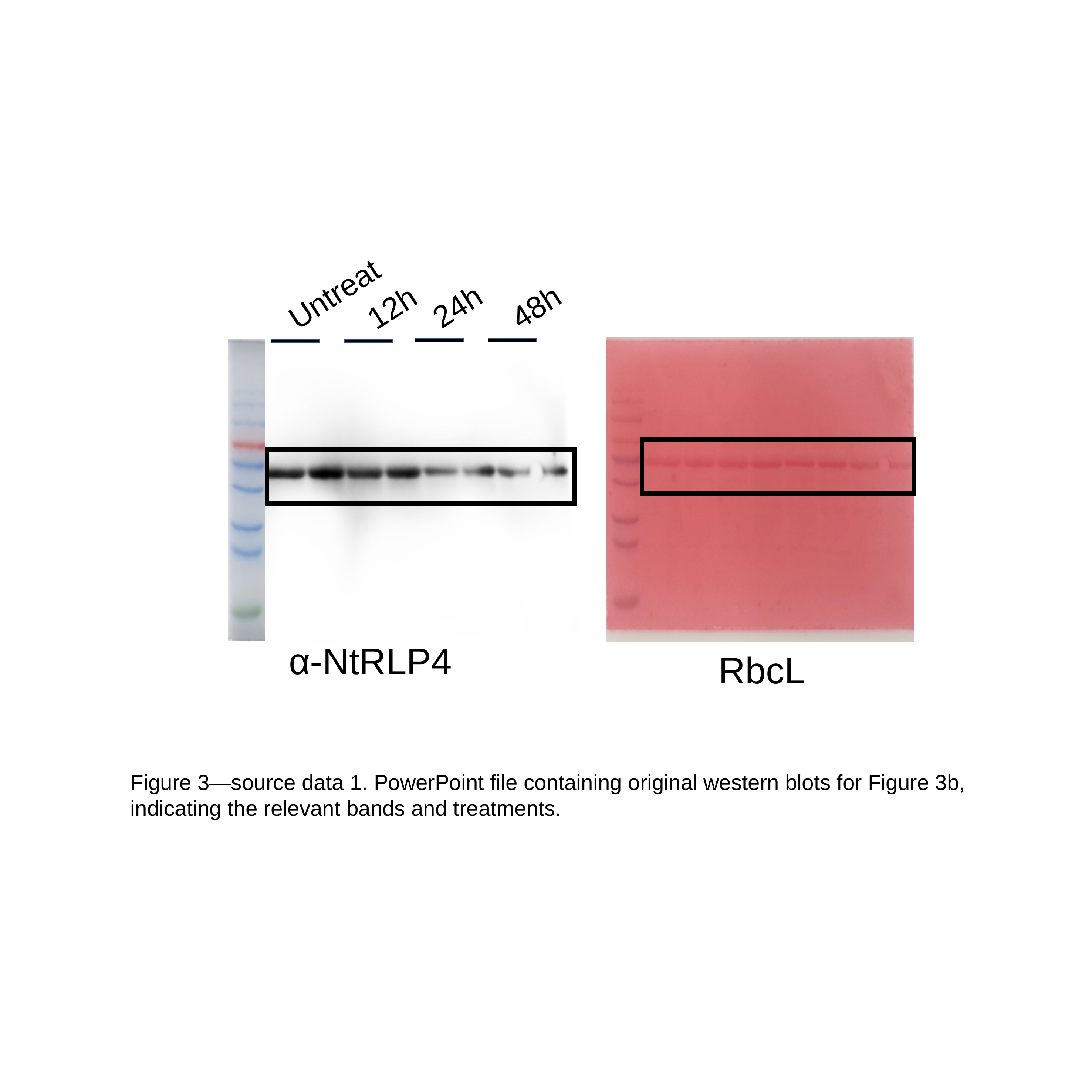

24h
48h
Untreat
12h
α-NtRLP4
RbcL
Figure 3—source data 1. PowerPoint file containing original western blots for Figure 3b, indicating the relevant bands and treatments.
