# Supplementary material for: Herbivorous insects independently evolved salivary effectors to regulate plant immunity by destabilizing the malectin-LRR RLP NtRLP4
Source: eLife. 2026 May 5;14:RP108737. doi: 10.7554/eLife.108737 (PMC13143284; doi:10.7554/eLife.108737)
Supplement: Figure 3—figure supplement 3—source data 3. [file elife-108737-fig3-figsupp3-data3.zip › Figure 3—figure supplement 3—source data 3.pptx]

## Slide 1
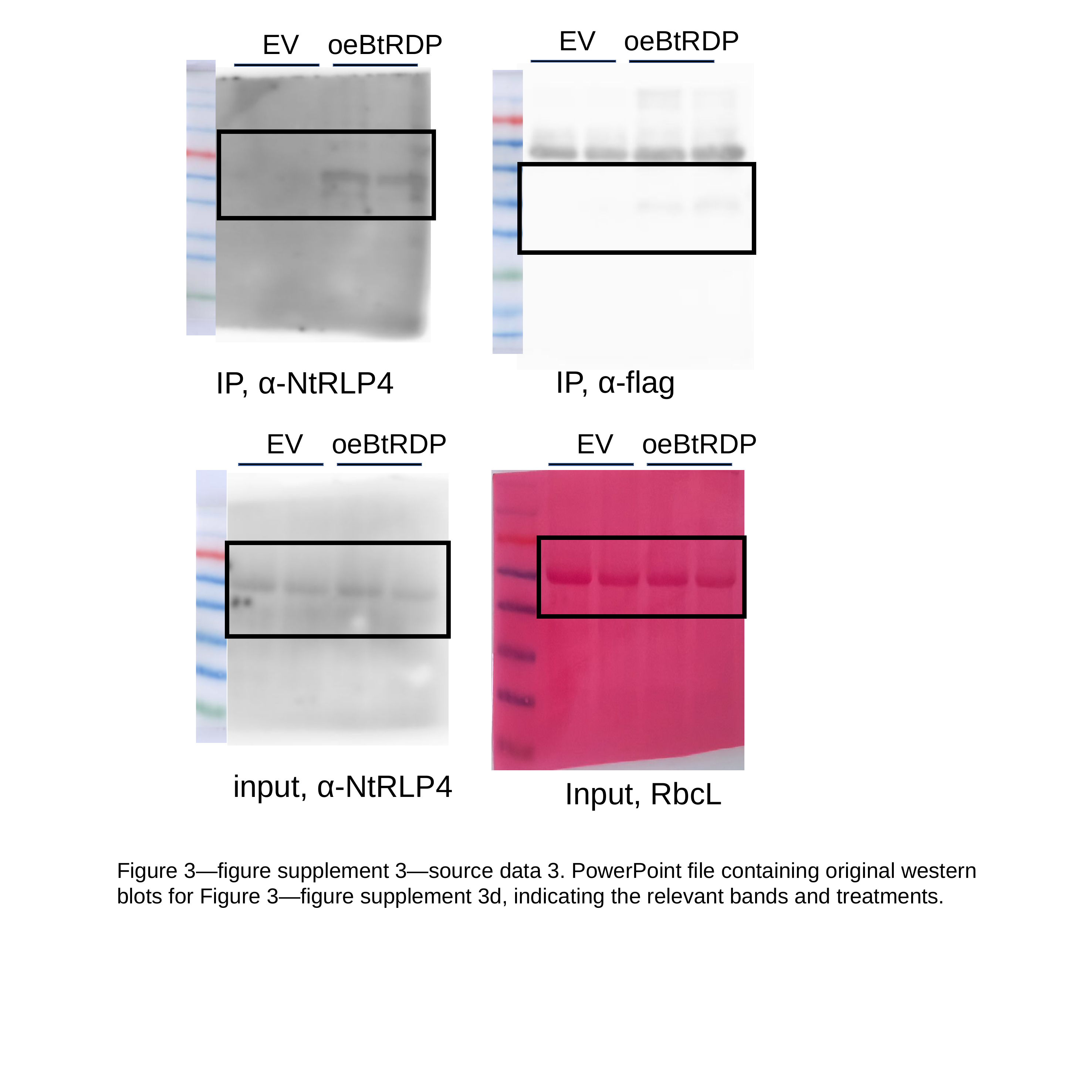

EV
oeBtRDP
EV
oeBtRDP
IP, α-flag
IP, α-NtRLP4
EV
oeBtRDP
EV
oeBtRDP
input, α-NtRLP4
Input, RbcL
Figure 3—figure supplement 3—source data 3. PowerPoint file containing original western blots for Figure 3—figure supplement 3d, indicating the relevant bands and treatments.
