# Supplementary material for: Herbivorous insects independently evolved salivary effectors to regulate plant immunity by destabilizing the malectin-LRR RLP NtRLP4
Source: eLife. 2026 May 5;14:RP108737. doi: 10.7554/eLife.108737 (PMC13143284; doi:10.7554/eLife.108737)
Supplement: Figure 4—source data 1. [file elife-108737-fig4-data1.zip › Figure 4—source data 1.pptx]

## Slide 1
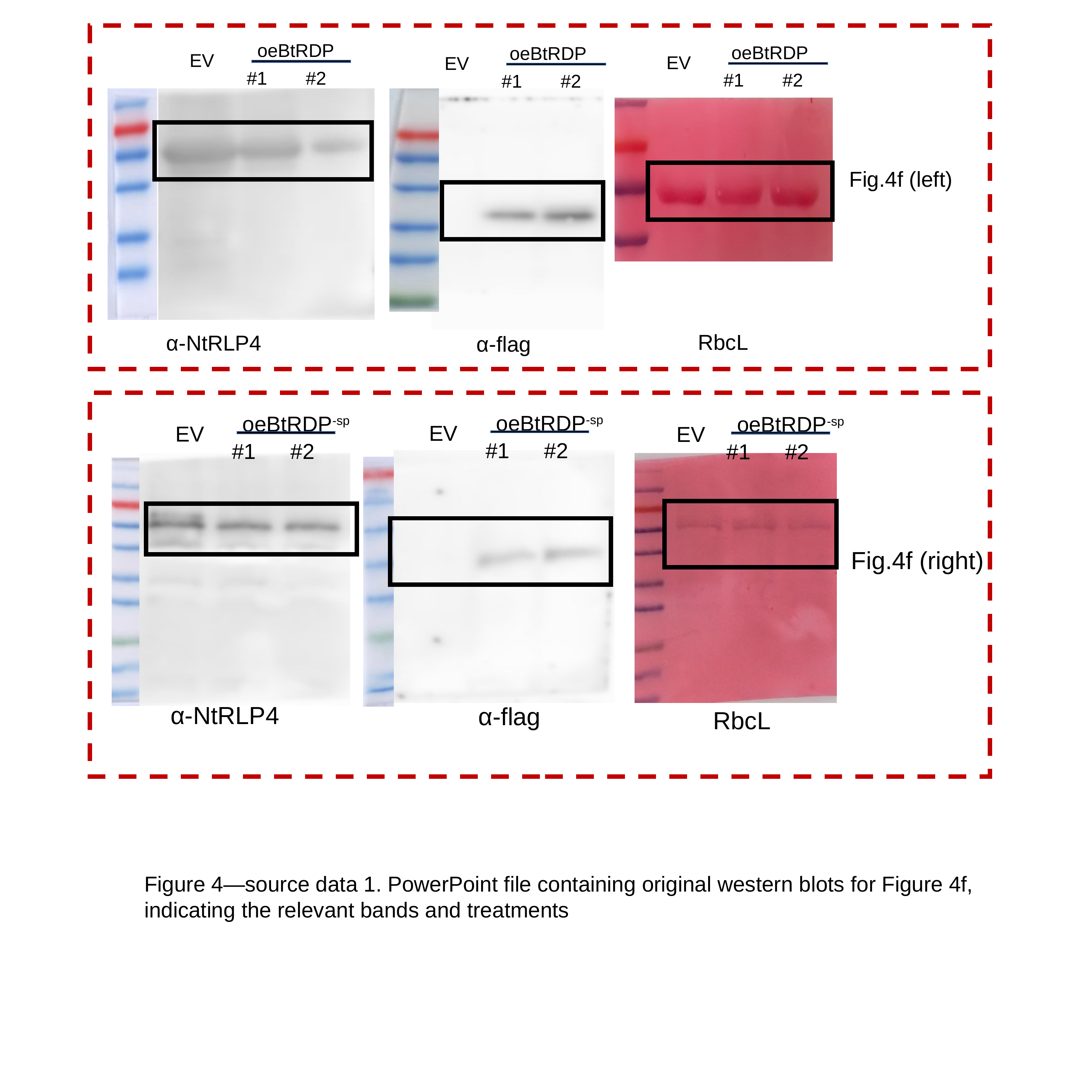

oeBtRDP
oeBtRDP
oeBtRDP
EV
EV
EV
#2
#1
#1
#2
#2
#1
Fig.4f (left)
RbcL
α-NtRLP4
α-flag
oeBtRDP-sp
oeBtRDP-sp
oeBtRDP-sp
EV
EV
EV
#2
#1
#2
#1
#2
#1
Fig.4f (right)
α-NtRLP4
α-flag
RbcL
Figure 4—source data 1. PowerPoint file containing original western blots for Figure 4f, indicating the relevant bands and treatments
