# Supplementary material for: Herbivorous insects independently evolved salivary effectors to regulate plant immunity by destabilizing the malectin-LRR RLP NtRLP4
Source: eLife. 2026 May 5;14:RP108737. doi: 10.7554/eLife.108737 (PMC13143284; doi:10.7554/eLife.108737)
Supplement: Figure 4—source data 5. [file elife-108737-fig4-data5.zip › Figure 4—source data 5.pptx]

## Slide 1
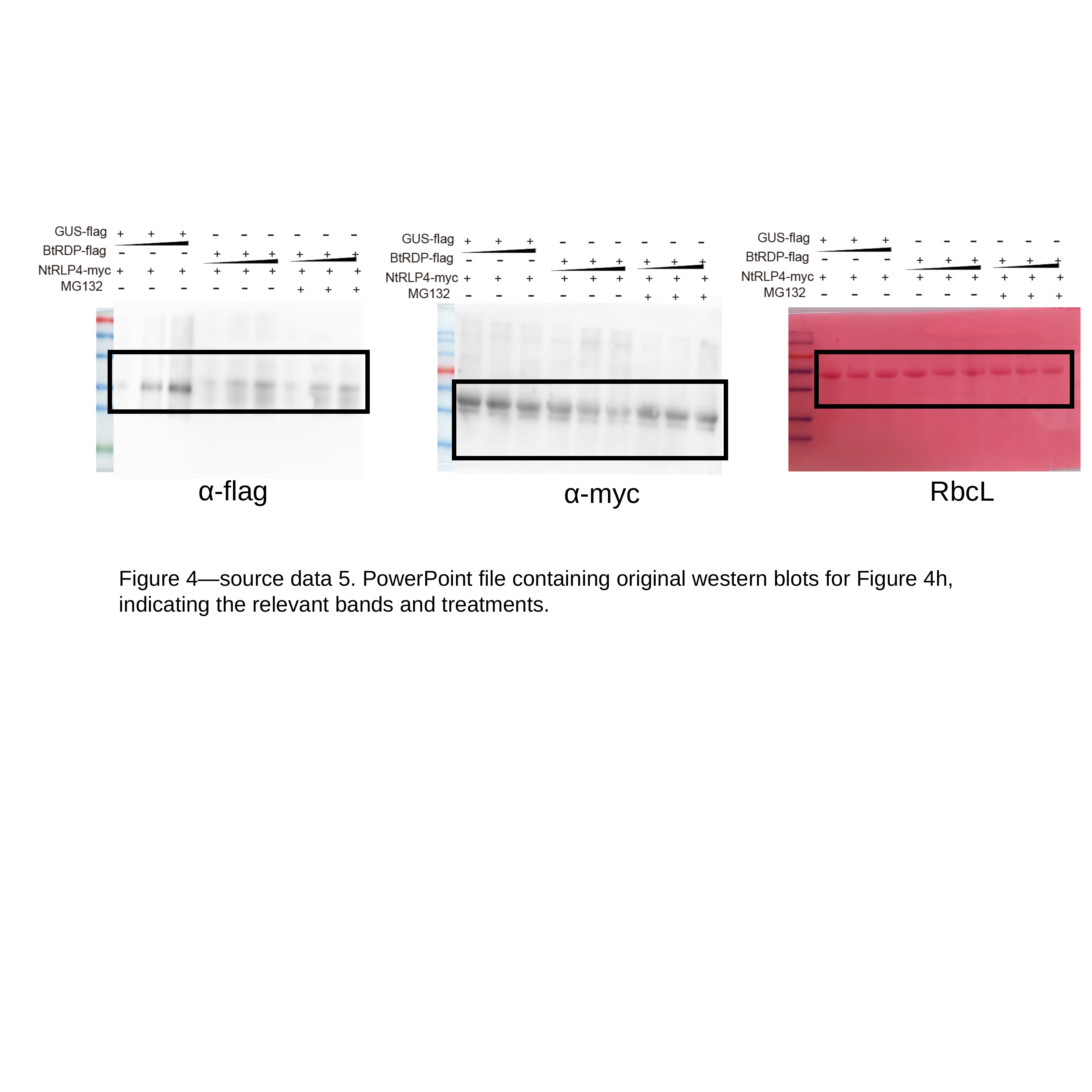

α-flag
RbcL
α-myc
Figure 4—source data 5. PowerPoint file containing original western blots for Figure 4h, indicating the relevant bands and treatments.
