# Supplementary material for: Herbivorous insects independently evolved salivary effectors to regulate plant immunity by destabilizing the malectin-LRR RLP NtRLP4
Source: eLife. 2026 May 5;14:RP108737. doi: 10.7554/eLife.108737 (PMC13143284; doi:10.7554/eLife.108737)
Supplement: Figure 4—figure supplement 8—source data 1. [file elife-108737-fig4-figsupp8-data1.zip › Figure 4—figure supplement 8—source data 1.pptx]

## Slide 1
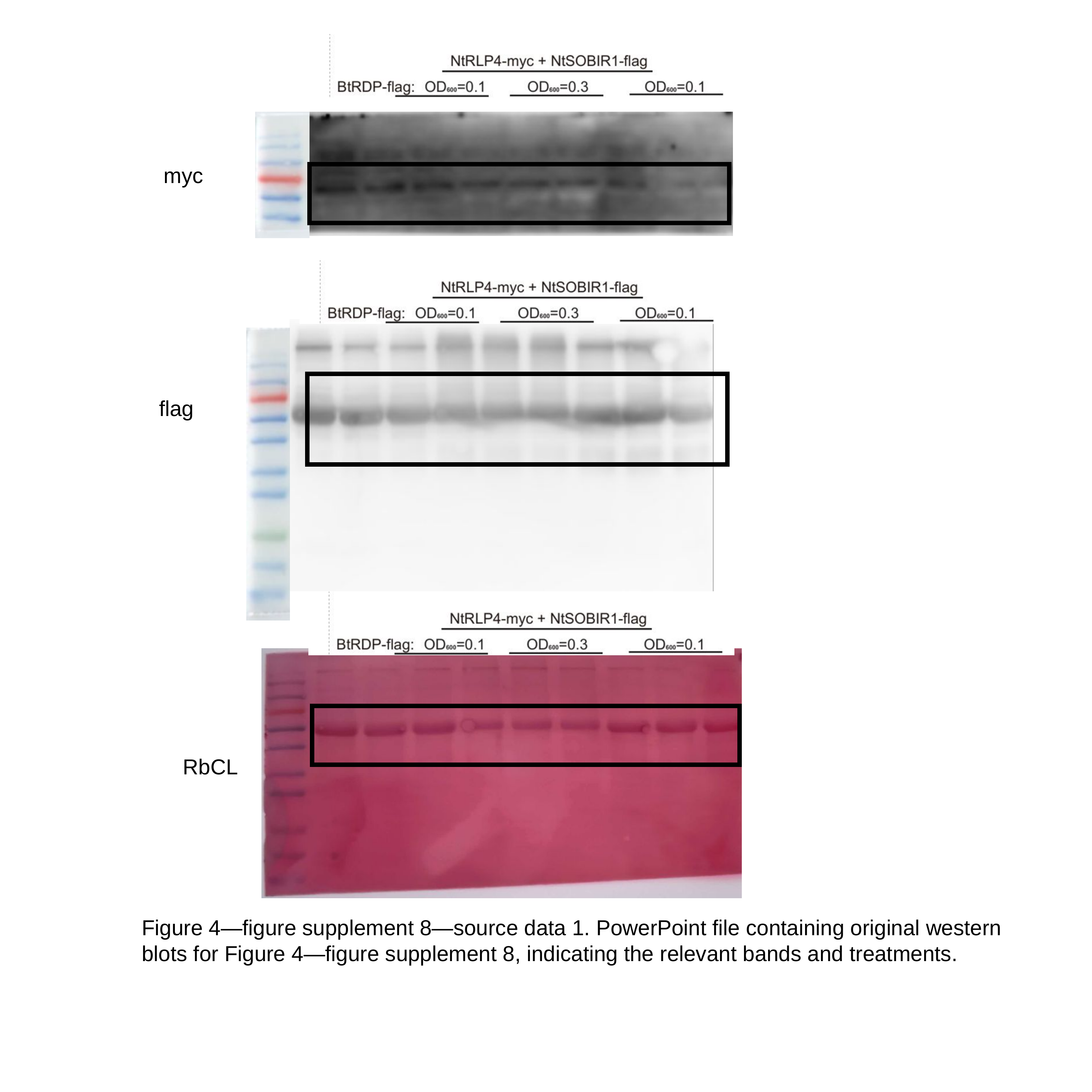

myc
flag
RbCL
Figure 4—figure supplement 8—source data 1. PowerPoint file containing original western blots for Figure 4—figure supplement 8, indicating the relevant bands and treatments.
