# Supplementary figures and images for: Herbivorous insects independently evolved salivary effectors to regulate plant immunity by destabilizing the malectin-LRR RLP NtRLP4 (part 2 of 2)
Source: eLife. 2026 May 5;14:RP108737. doi: 10.7554/eLife.108737 (PMC13143284; doi:10.7554/eLife.108737)

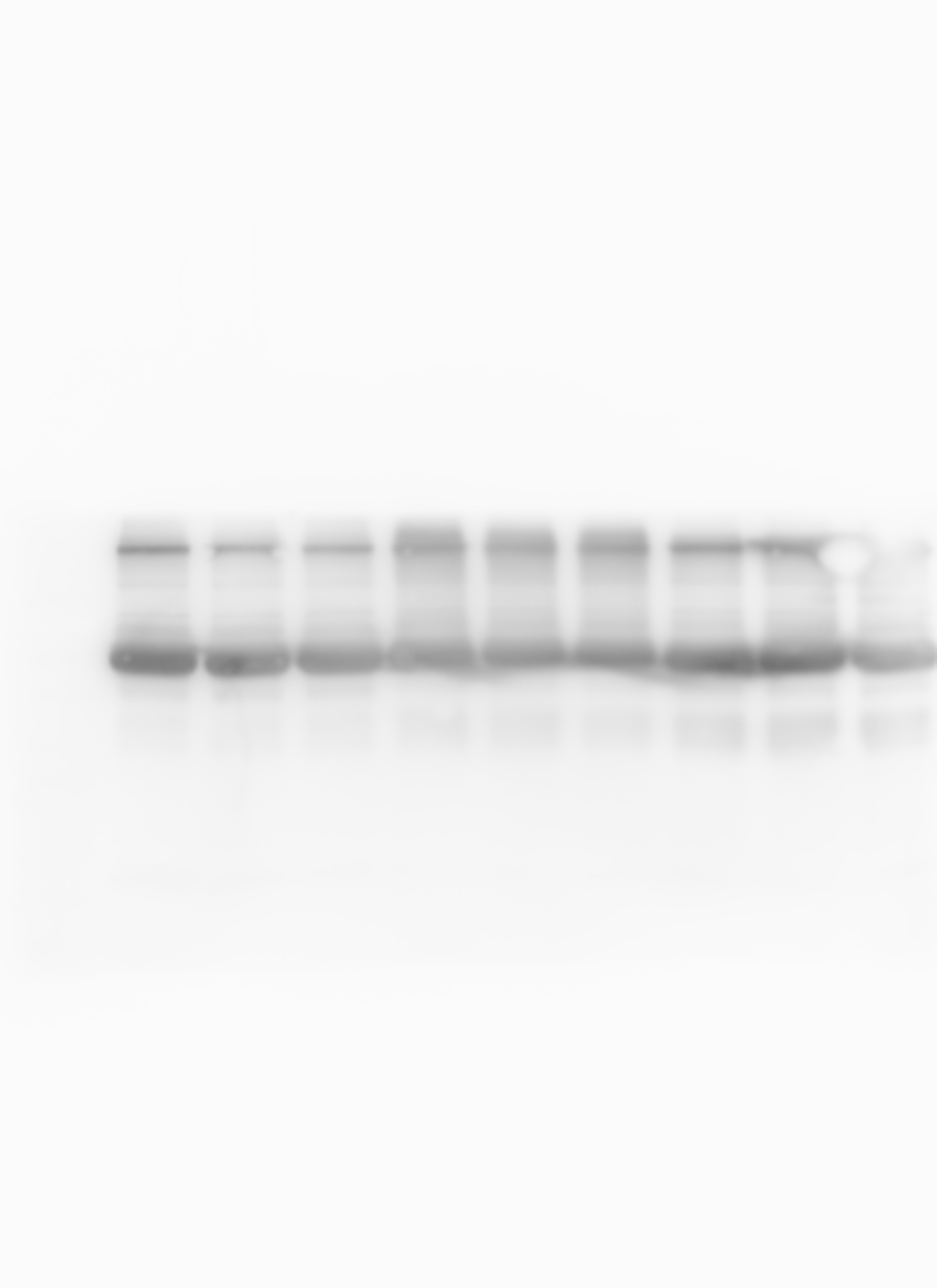

Supplement: Figure 4—figure supplement 8—source data 2. [file elife-108737-fig4-figsupp8-data2.zip › Figure 4—figure supplement 8—source data 2/flag.tif]

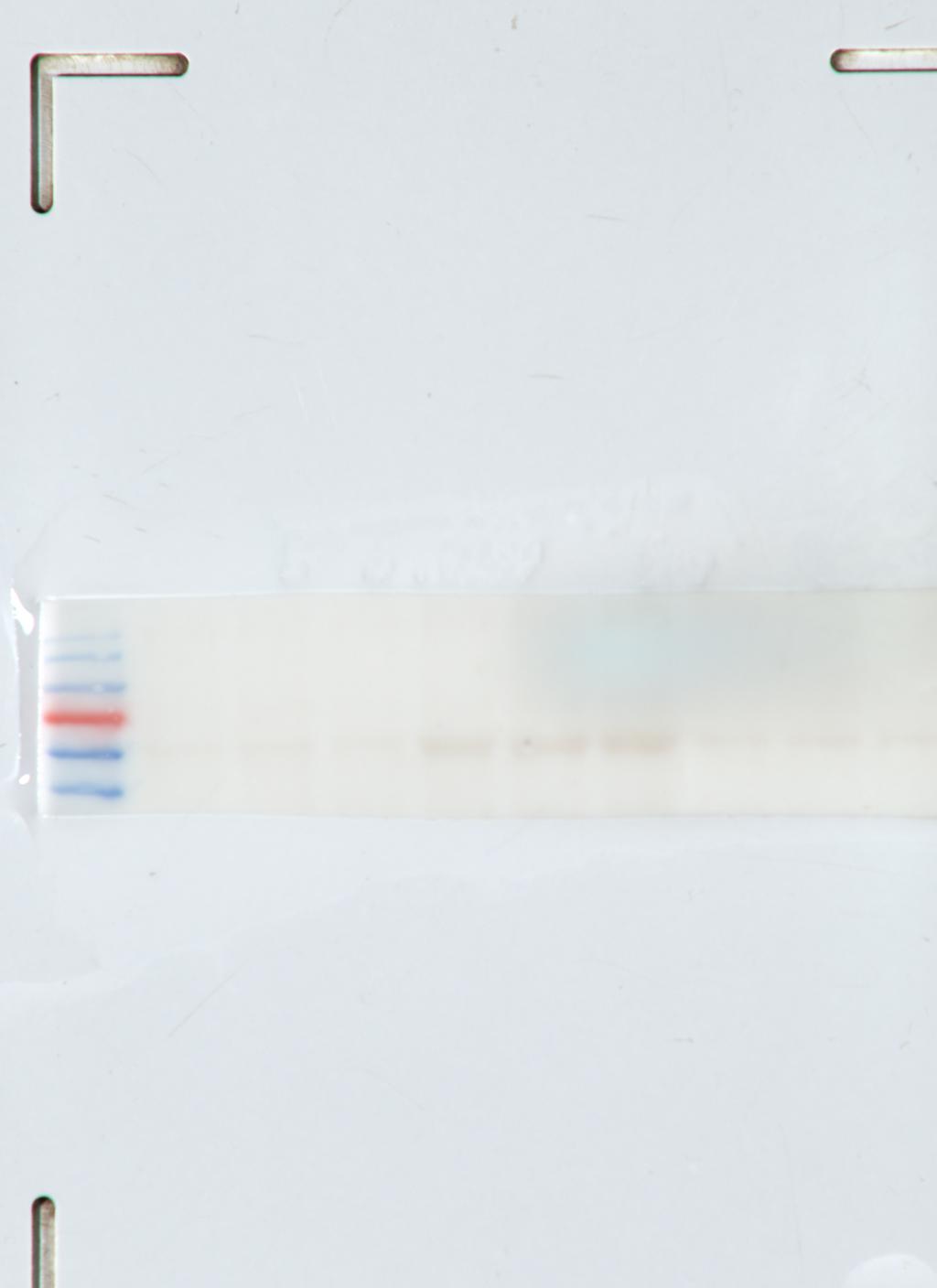

Supplement: Figure 4—figure supplement 8—source data 2. [file elife-108737-fig4-figsupp8-data2.zip › Figure 4—figure supplement 8—source data 2/myc.jpg]

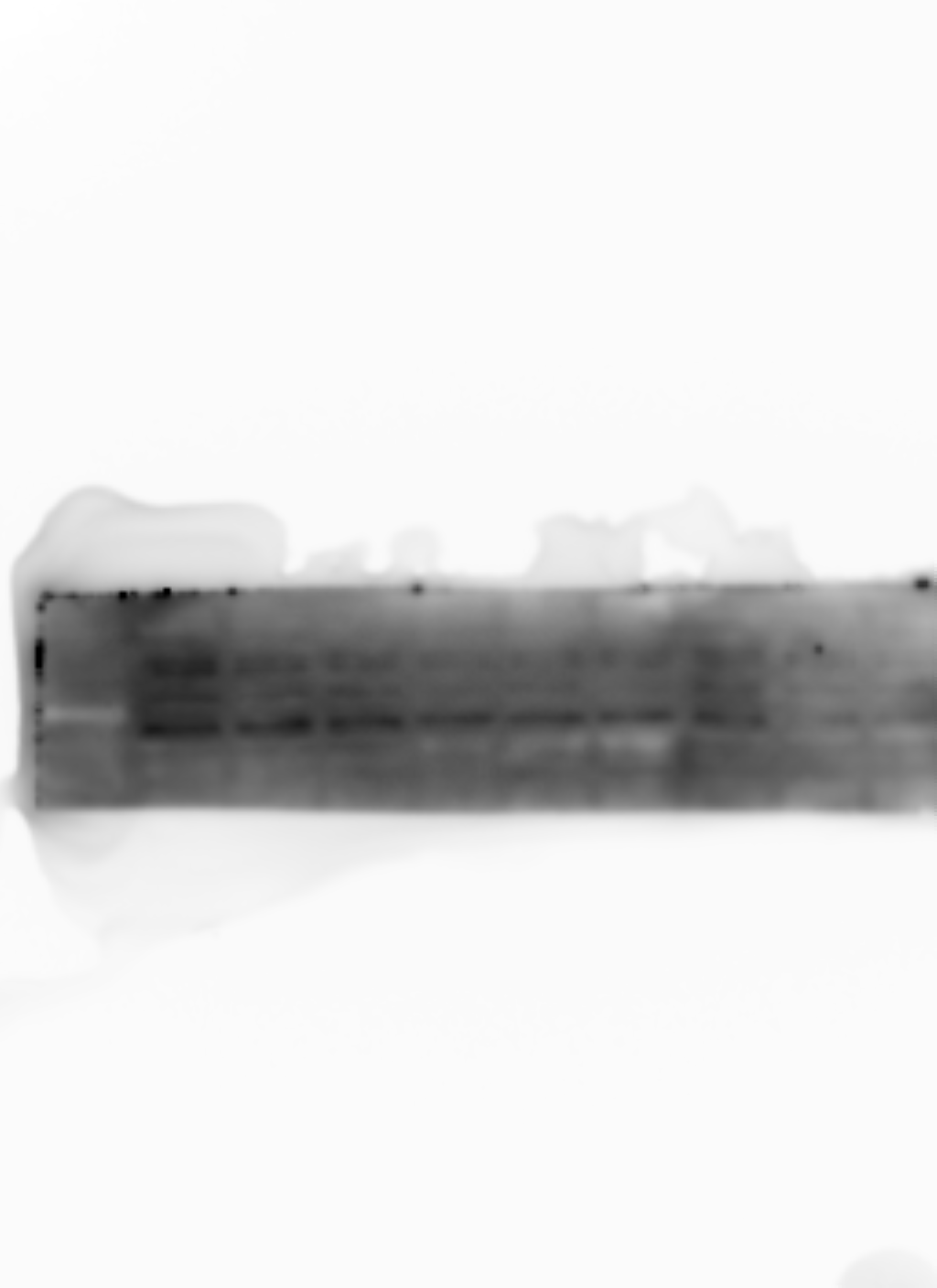

Supplement: Figure 4—figure supplement 8—source data 2. [file elife-108737-fig4-figsupp8-data2.zip › Figure 4—figure supplement 8—source data 2/myc.tif]

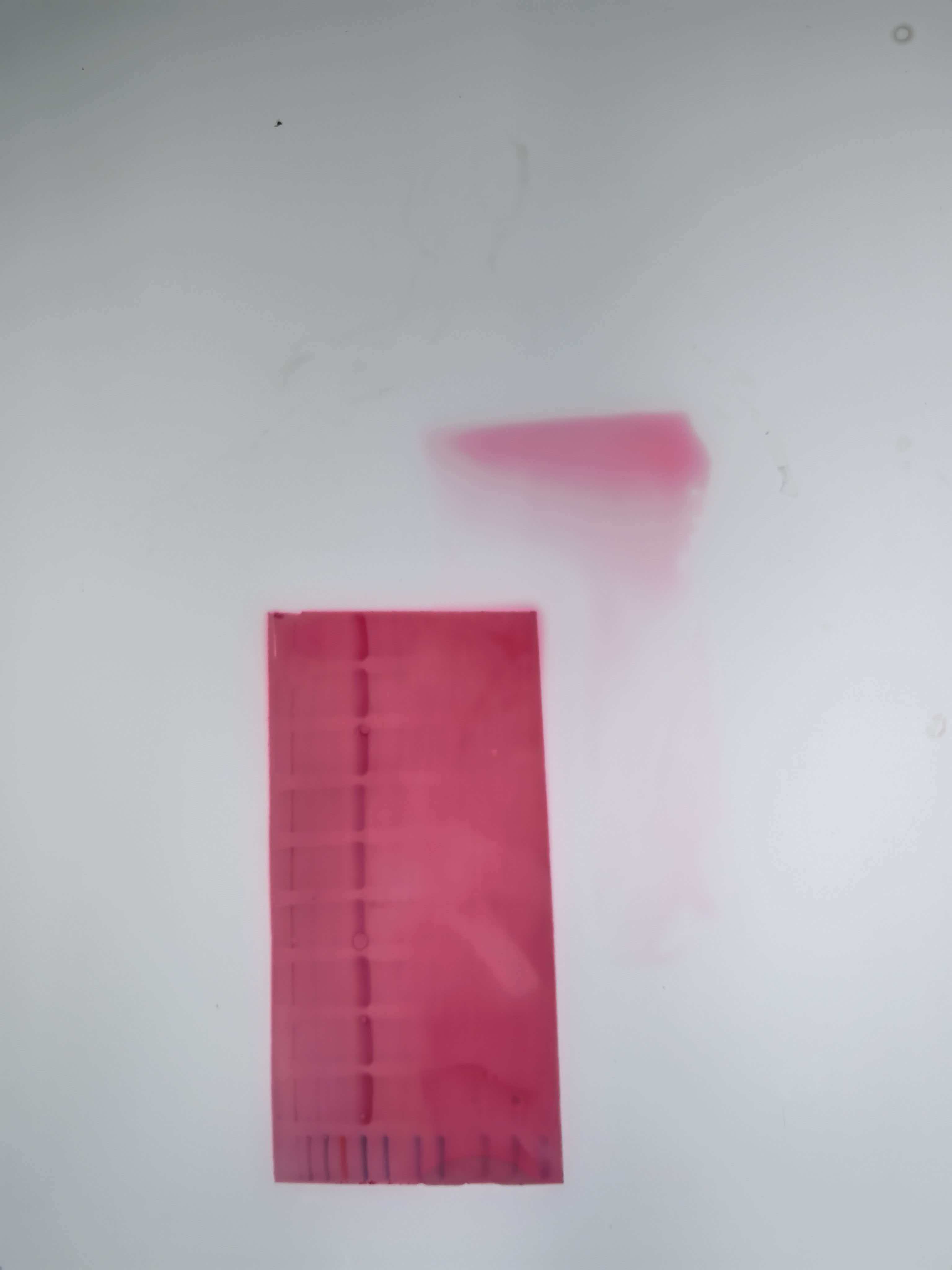

Supplement: Figure 4—figure supplement 8—source data 2. [file elife-108737-fig4-figsupp8-data2.zip › Figure 4—figure supplement 8—source data 2/RbCL-2.jpg]

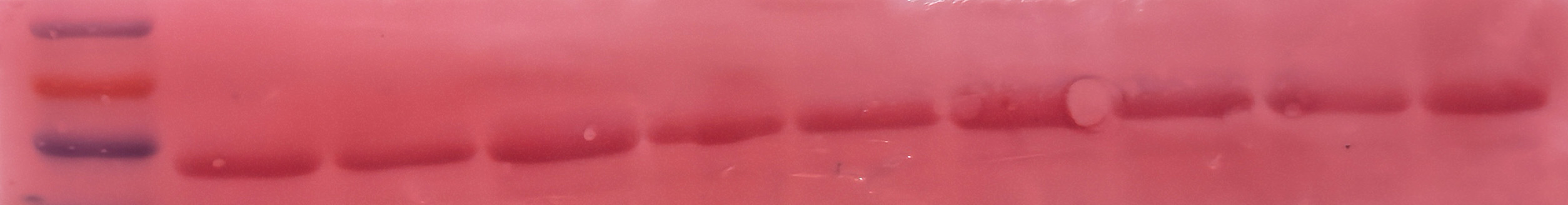

Supplement: Figure 4—figure supplement 10—source data 2. [file elife-108737-fig4-figsupp10-data2.zip › Figure 4—figure supplement 10—source data 2/RbCL.jpg]

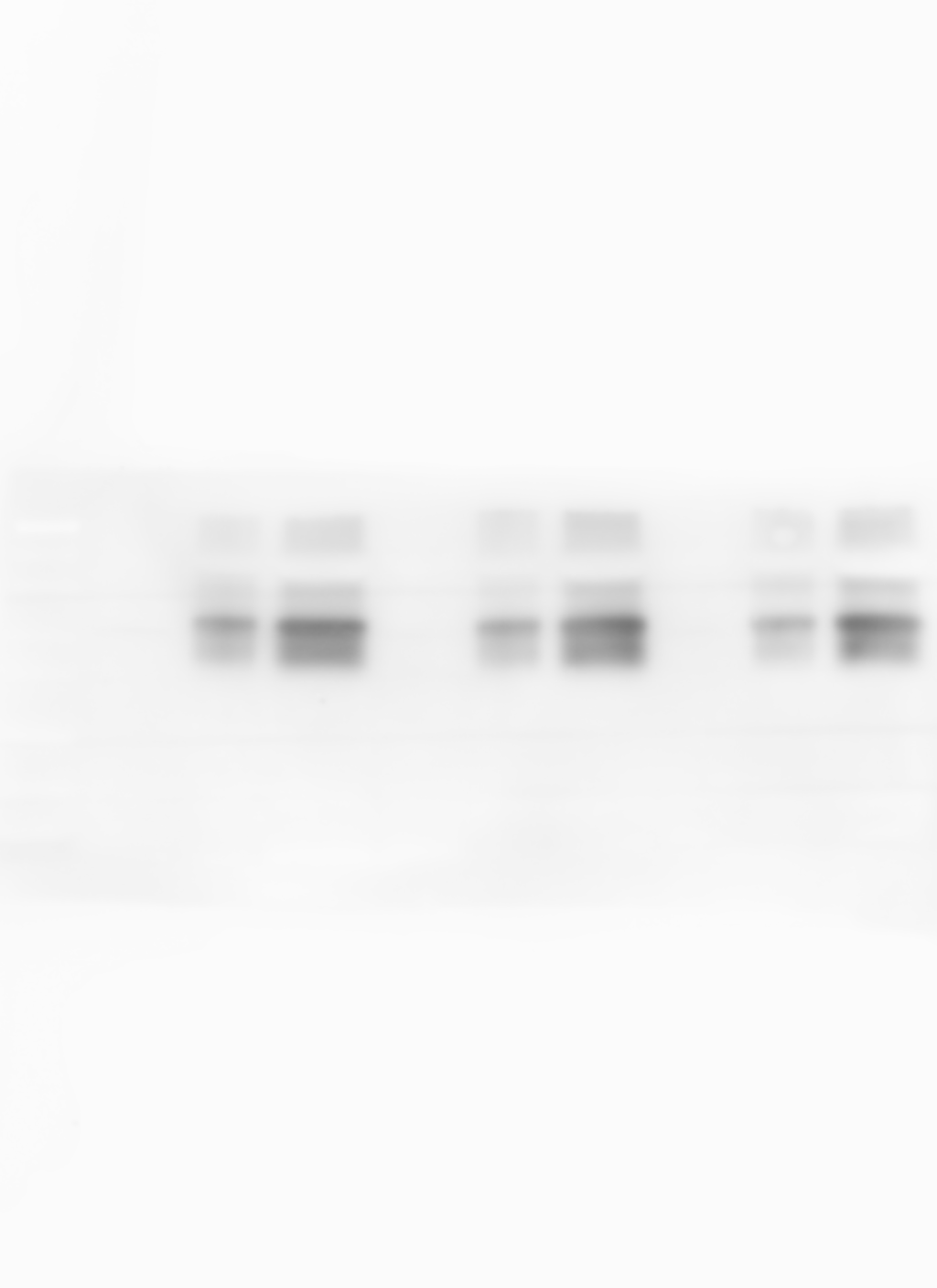

Supplement: Figure 4—figure supplement 10—source data 2. [file elife-108737-fig4-figsupp10-data2.zip › Figure 4—figure supplement 10—source data 2/α-flag-blot.tif]

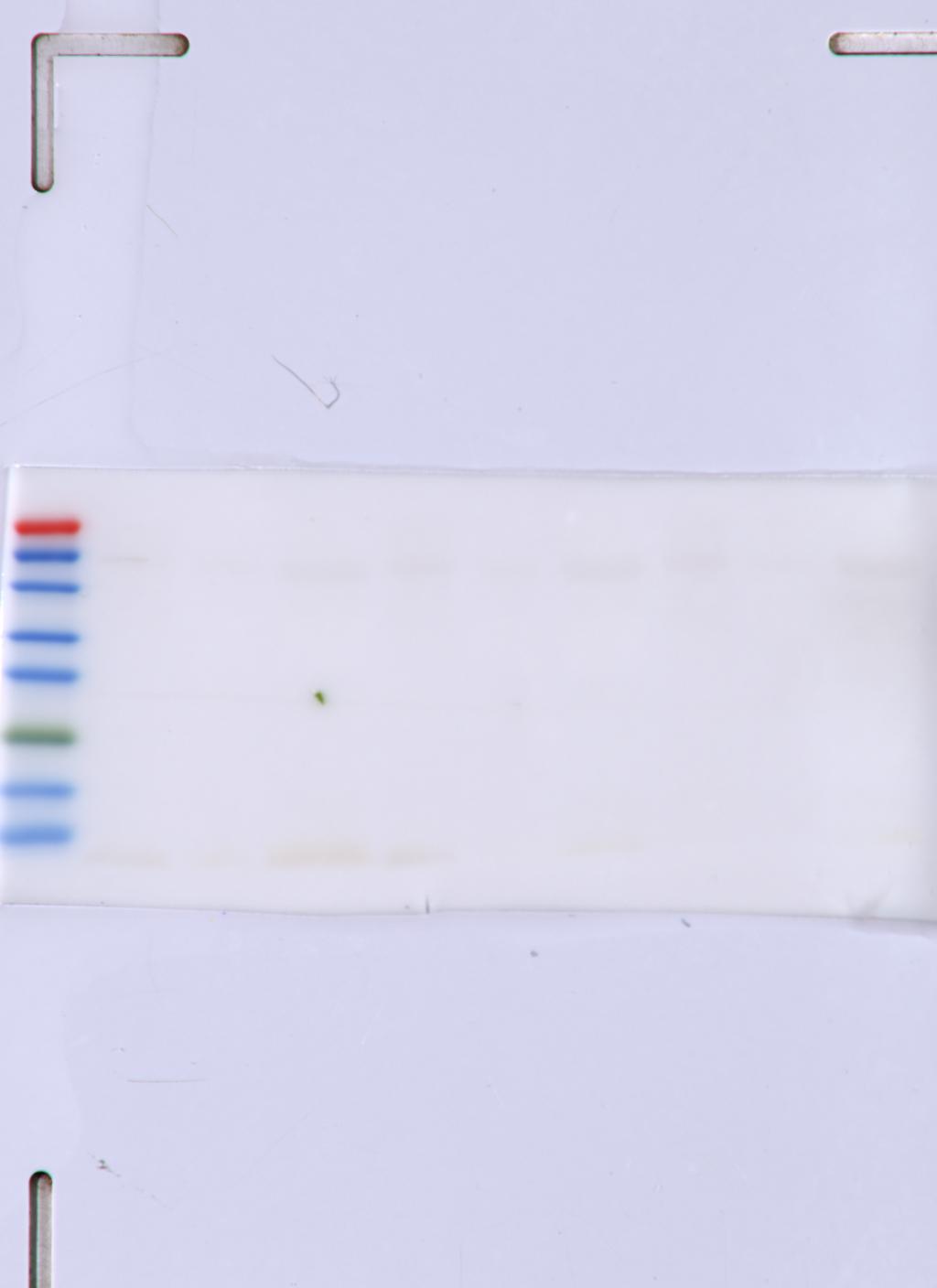

Supplement: Figure 4—figure supplement 10—source data 2. [file elife-108737-fig4-figsupp10-data2.zip › Figure 4—figure supplement 10—source data 2/α-flag-marker.jpg]

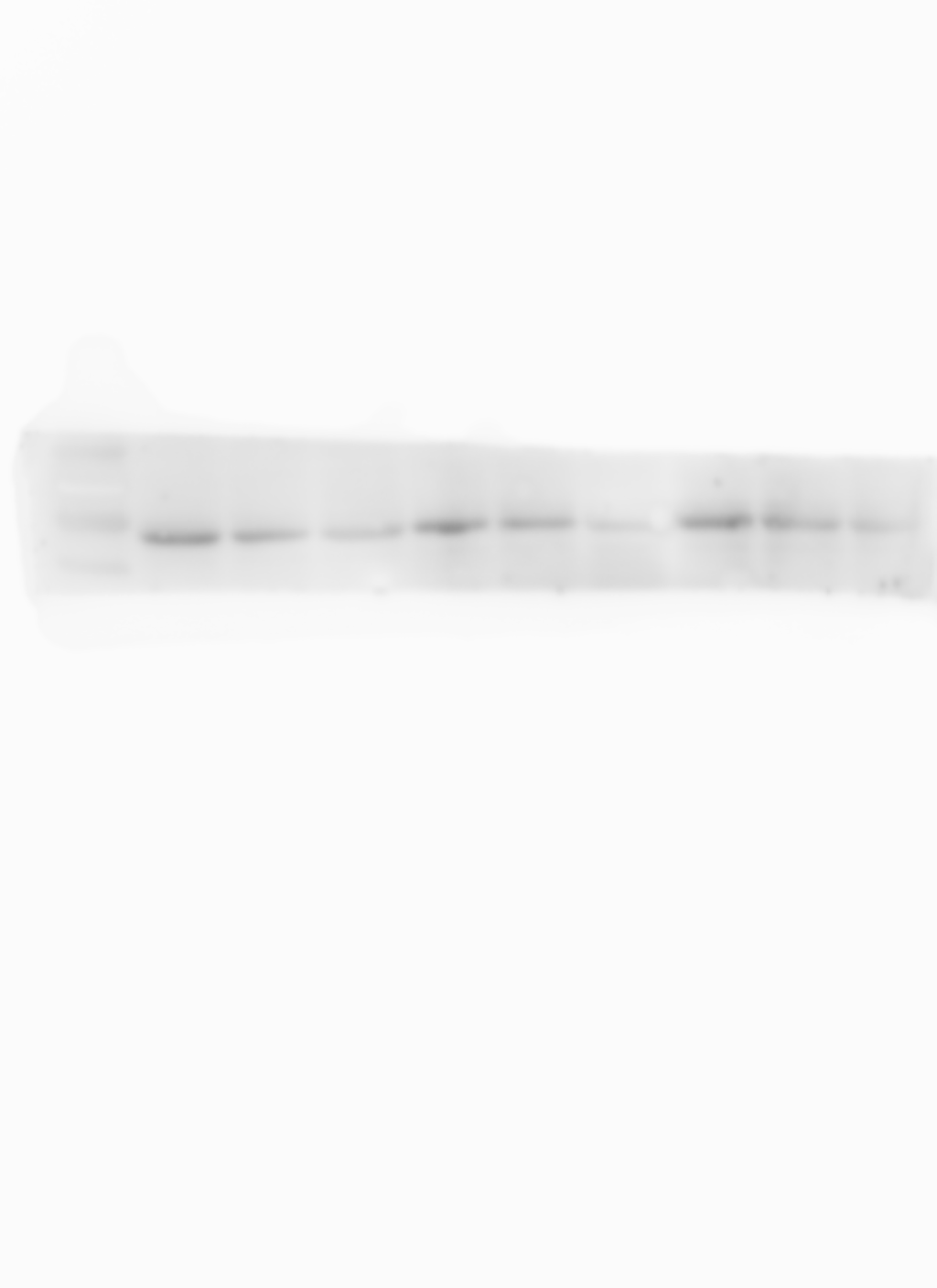

Supplement: Figure 4—figure supplement 10—source data 2. [file elife-108737-fig4-figsupp10-data2.zip › Figure 4—figure supplement 10—source data 2/α-myc-blot.tif]

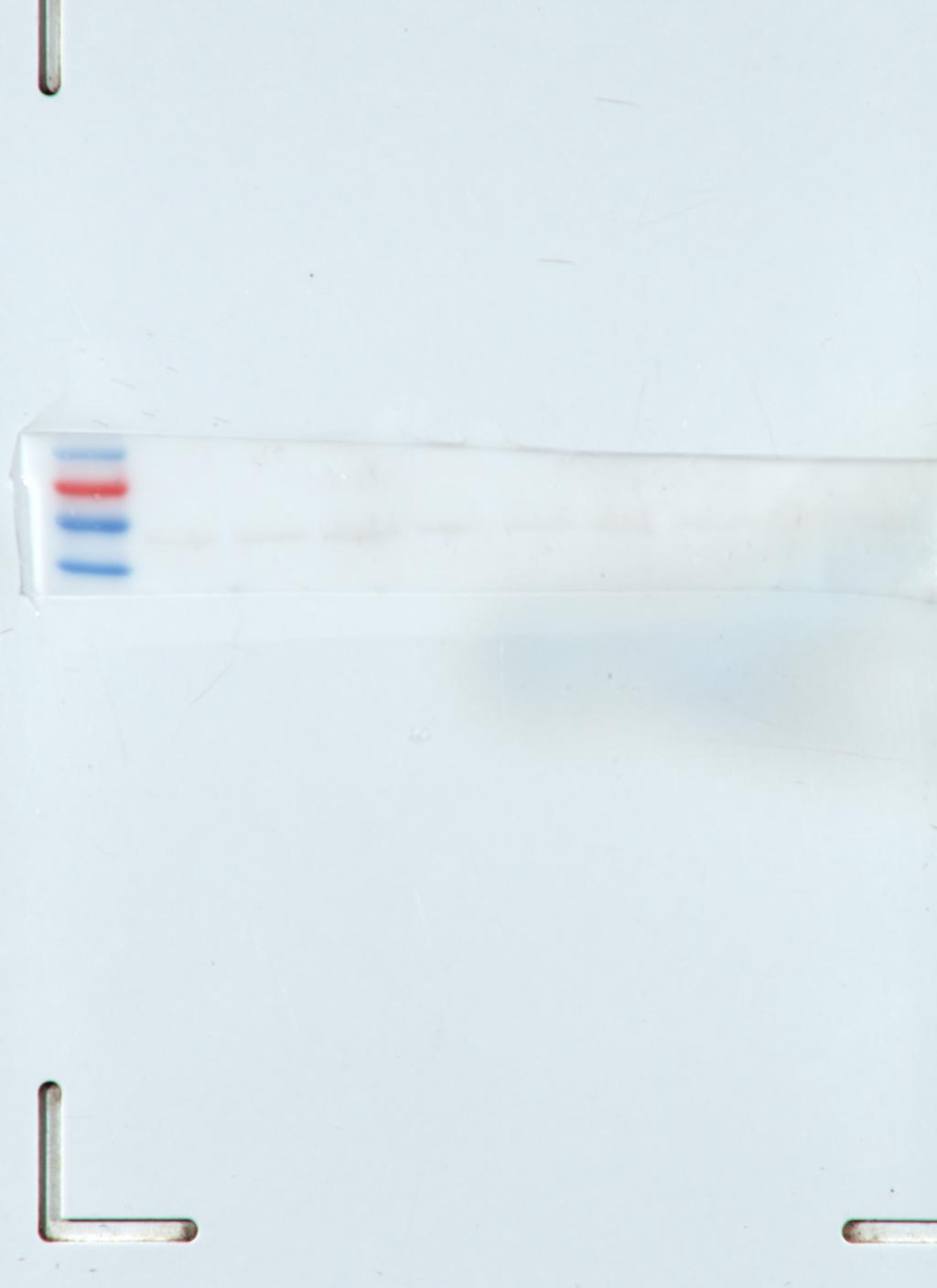

Supplement: Figure 4—figure supplement 10—source data 2. [file elife-108737-fig4-figsupp10-data2.zip › Figure 4—figure supplement 10—source data 2/α-myc-marker.jpg]

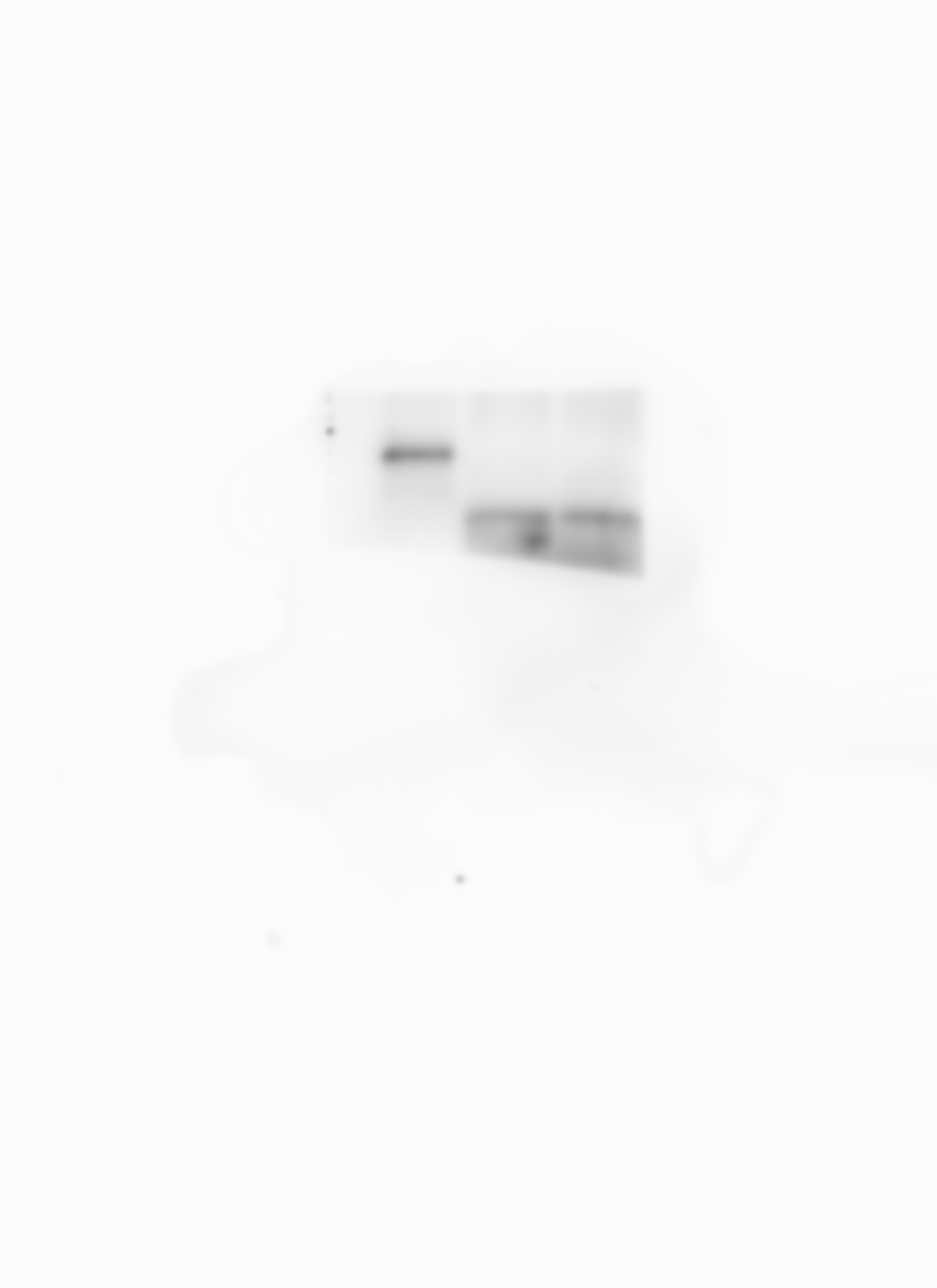

Supplement: Figure 5—source data 2. [file elife-108737-fig5-data2.zip › Figure 5—source data 2/input-flag-blot.tif]

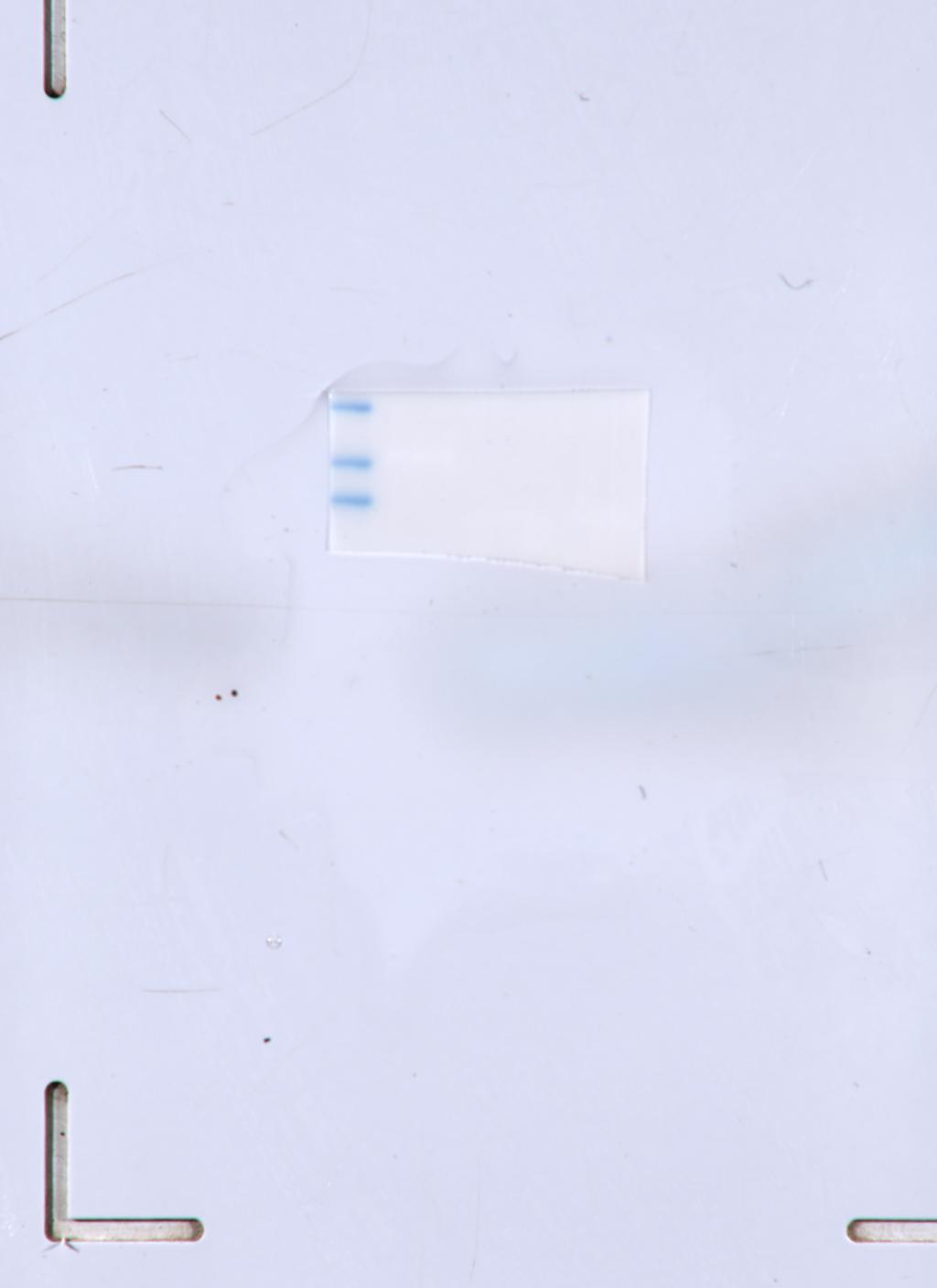

Supplement: Figure 5—source data 2. [file elife-108737-fig5-data2.zip › Figure 5—source data 2/input-flag-marker.jpg]

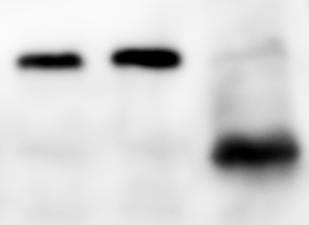

Supplement: Figure 5—source data 2. [file elife-108737-fig5-data2.zip › Figure 5—source data 2/input-myc-blot.tif]

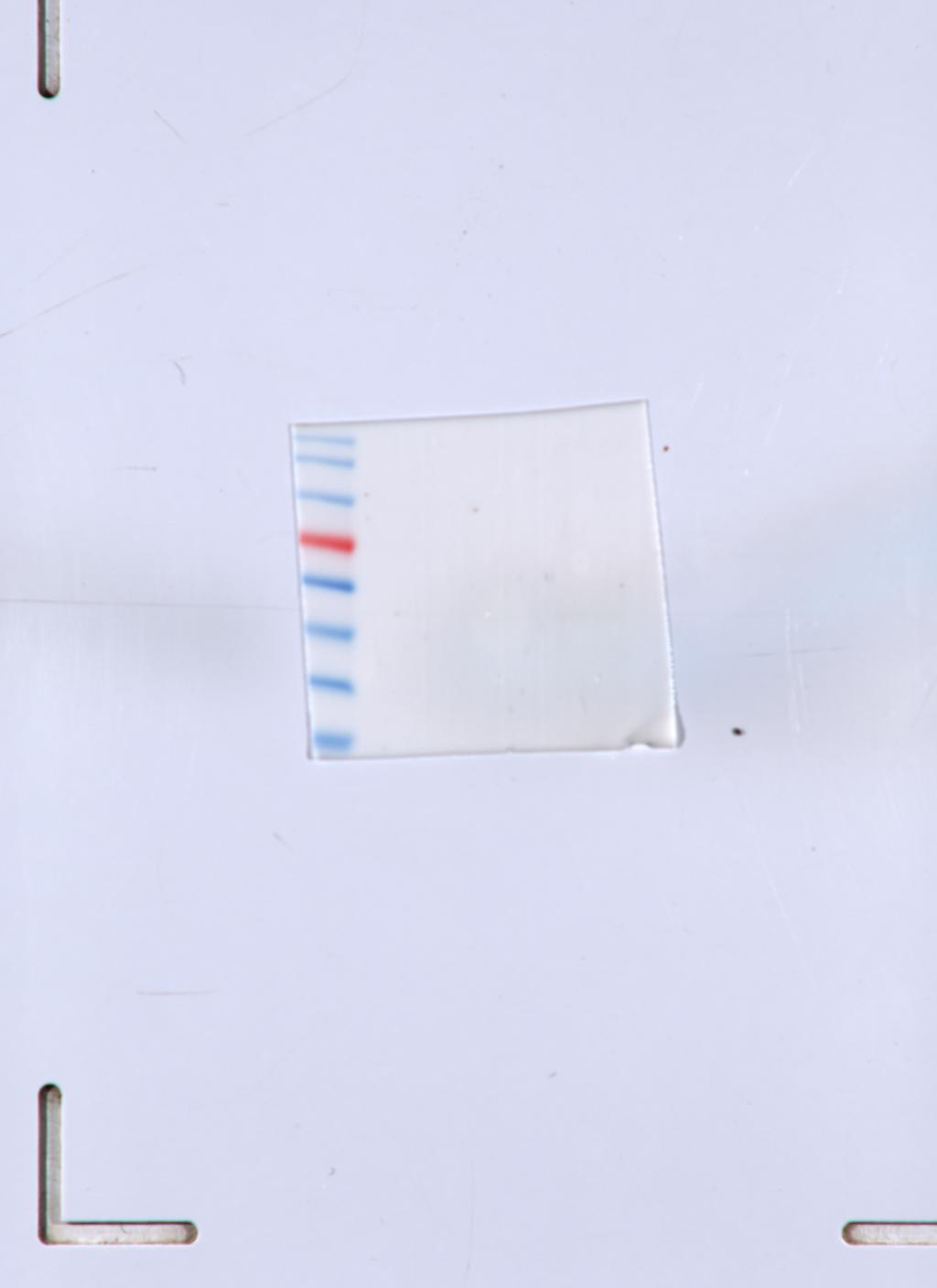

Supplement: Figure 5—source data 2. [file elife-108737-fig5-data2.zip › Figure 5—source data 2/input-myc-marker.jpg]

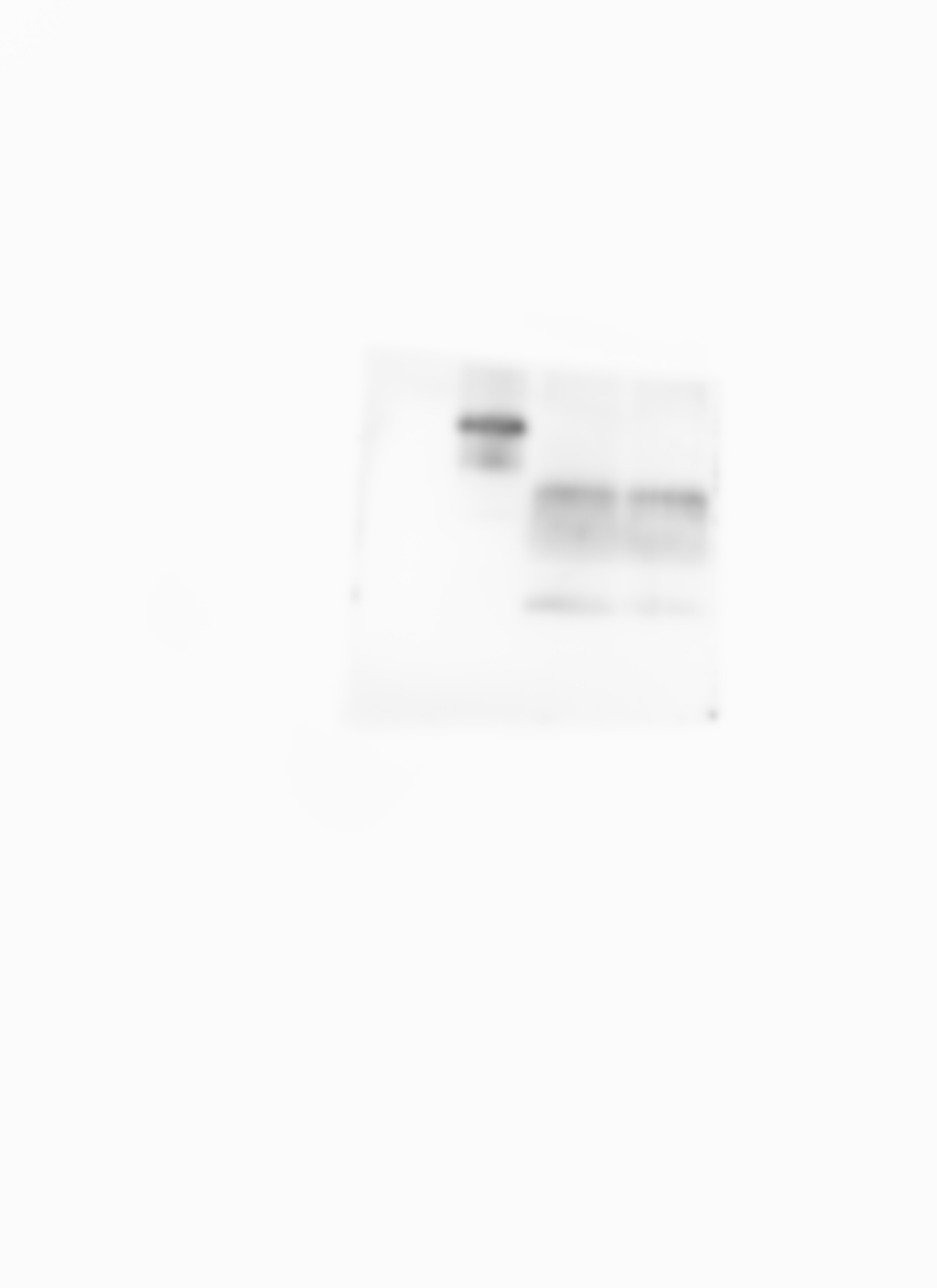

Supplement: Figure 5—source data 2. [file elife-108737-fig5-data2.zip › Figure 5—source data 2/IP-flag-blot.tif]

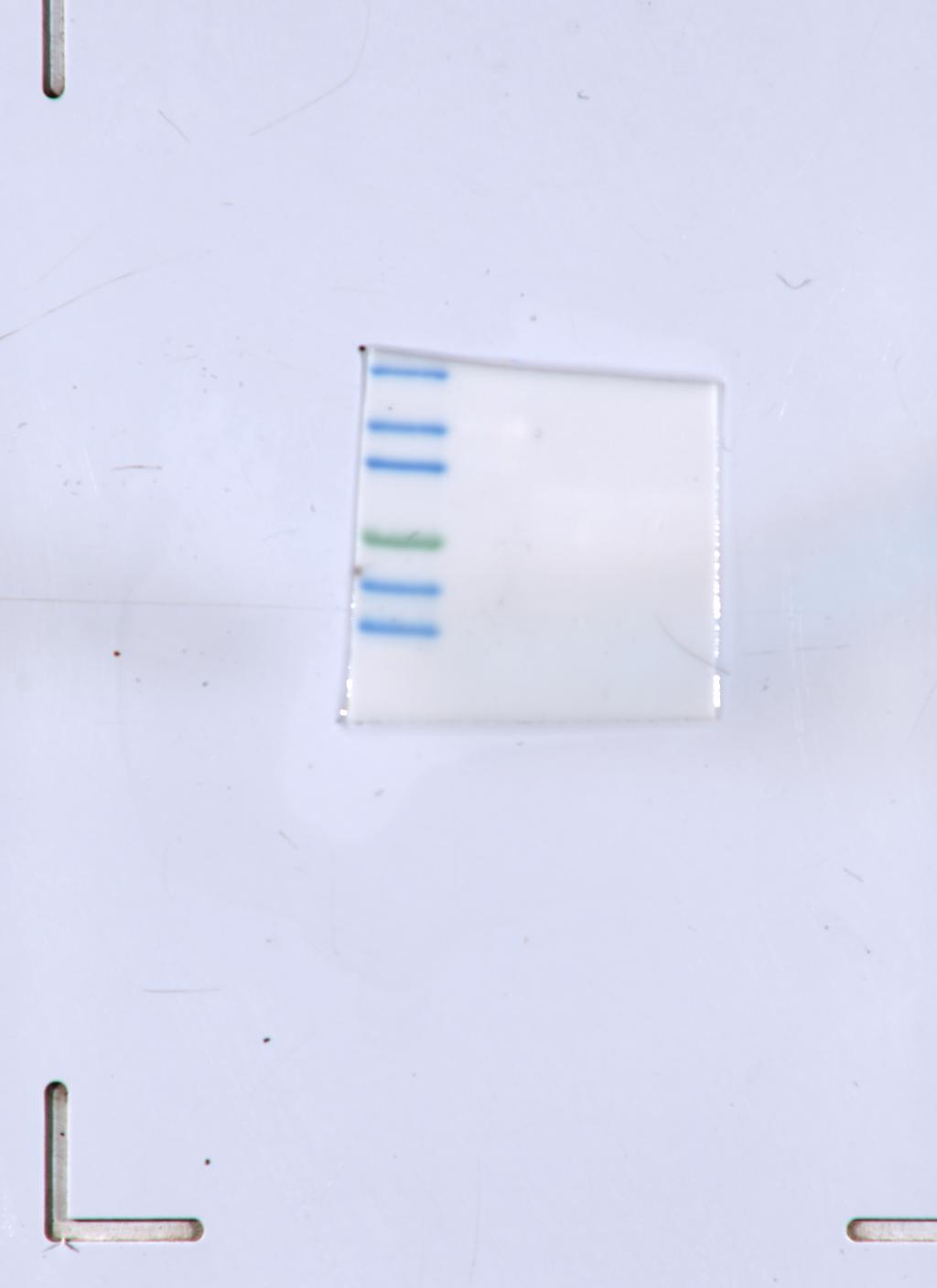

Supplement: Figure 5—source data 2. [file elife-108737-fig5-data2.zip › Figure 5—source data 2/IP-flag-marker.jpg]

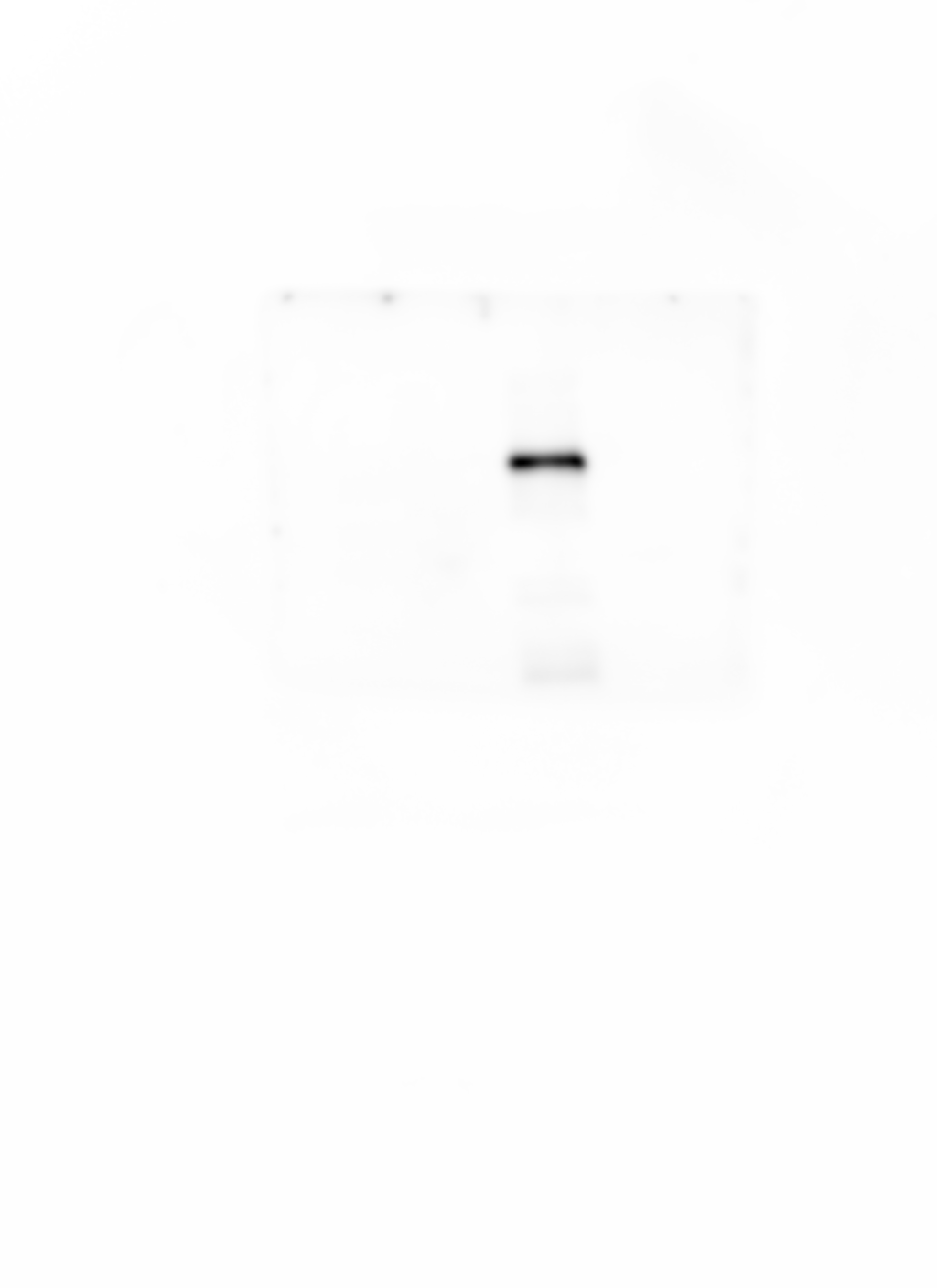

Supplement: Figure 5—source data 2. [file elife-108737-fig5-data2.zip › Figure 5—source data 2/IP-myc-blot.tif]

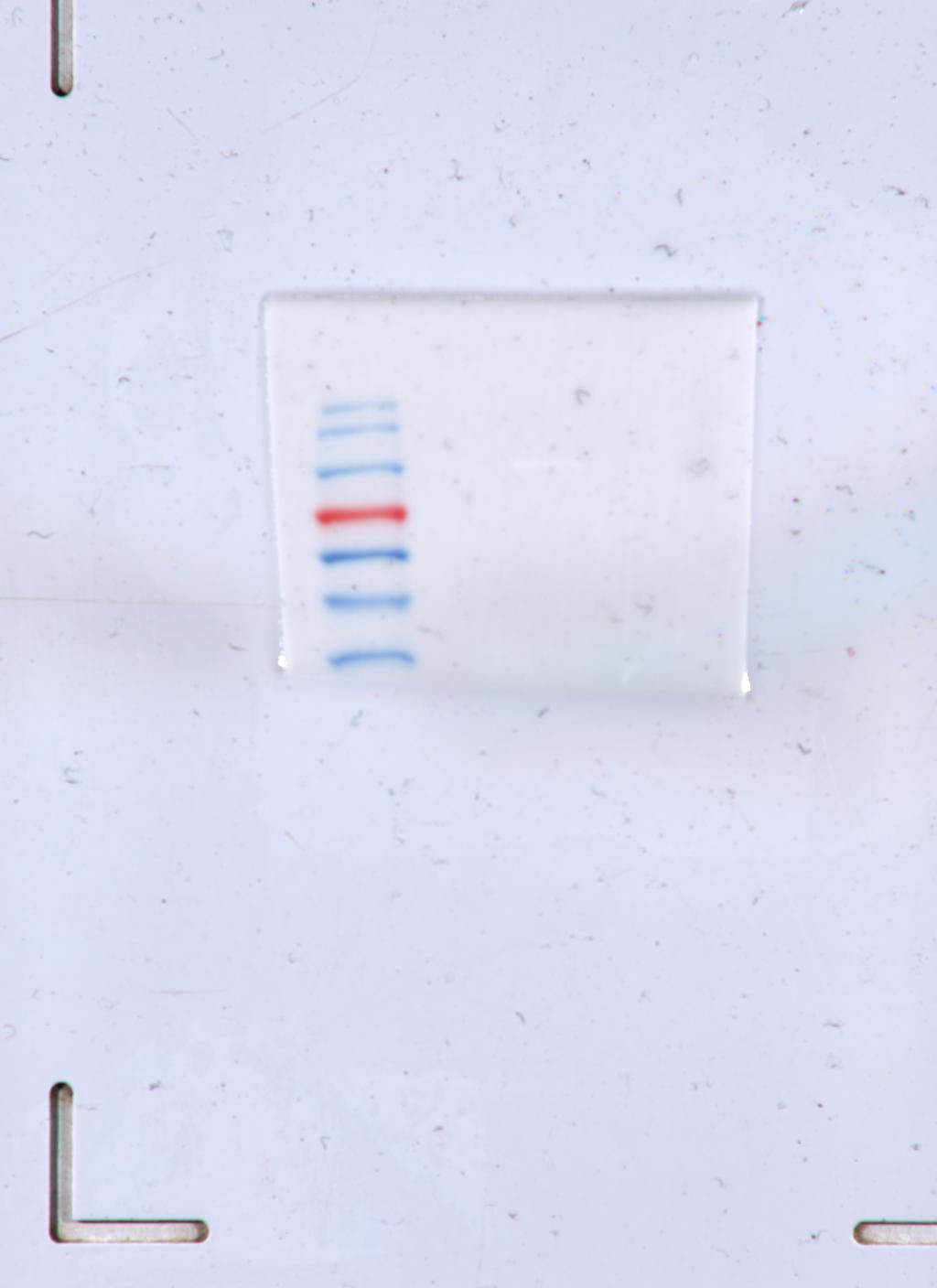

Supplement: Figure 5—source data 2. [file elife-108737-fig5-data2.zip › Figure 5—source data 2/IP-myc-marker.jpg]

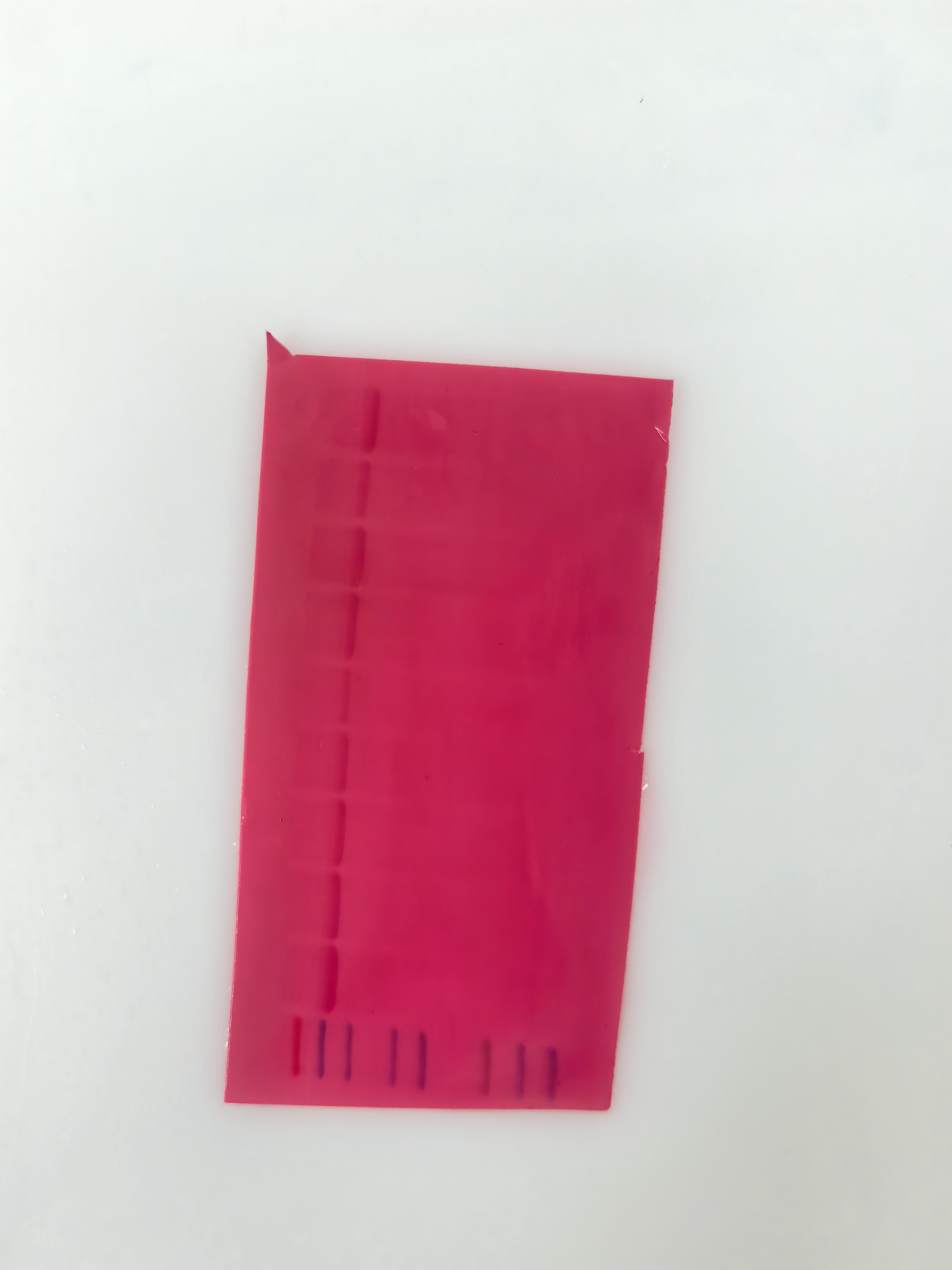

Supplement: Figure 5—source data 4. [file elife-108737-fig5-data4.zip › Figure 5—source data 4/RbCL.jpg]

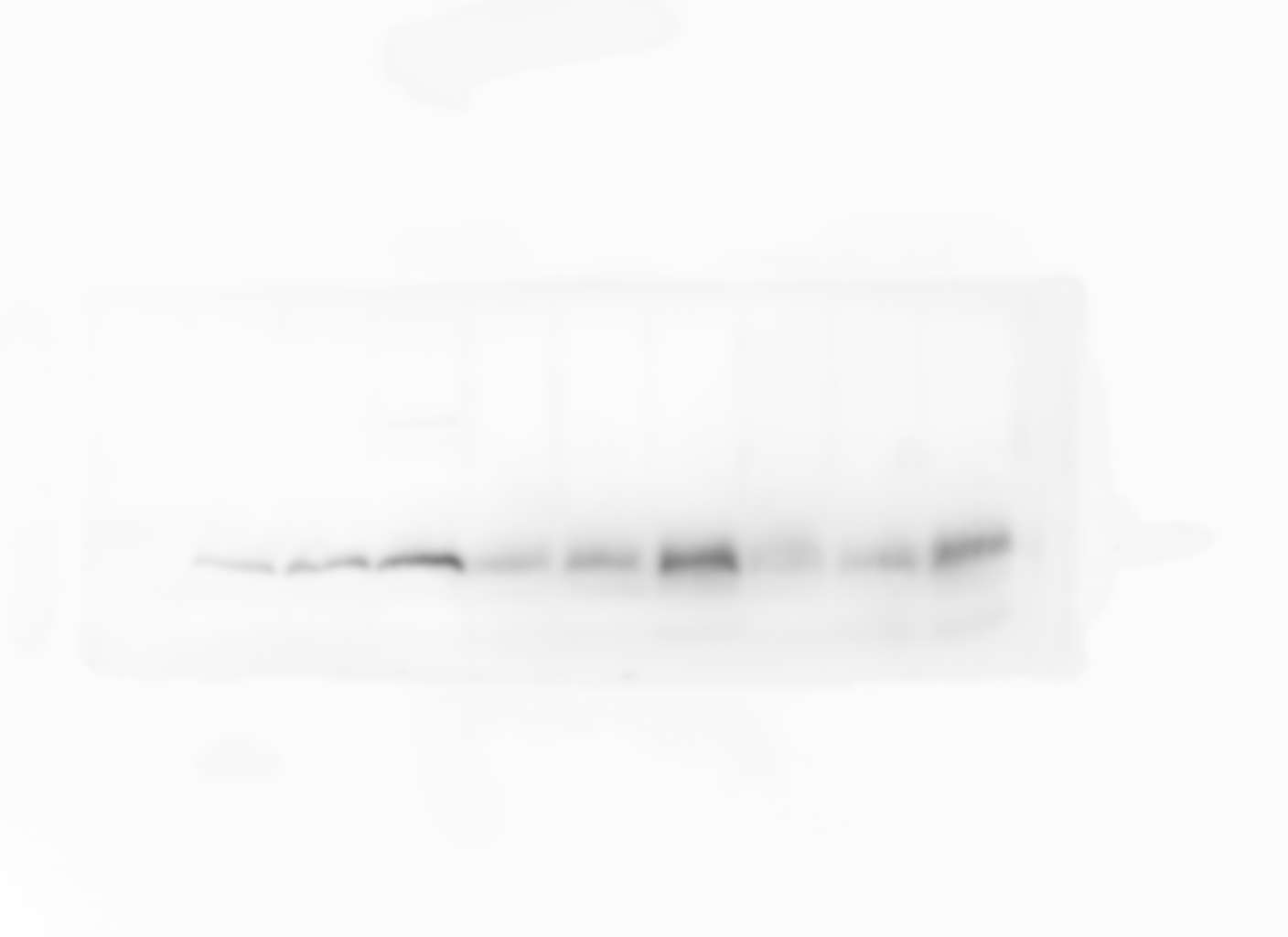

Supplement: Figure 5—source data 4. [file elife-108737-fig5-data4.zip › Figure 5—source data 4/α-flag-blot.tif]

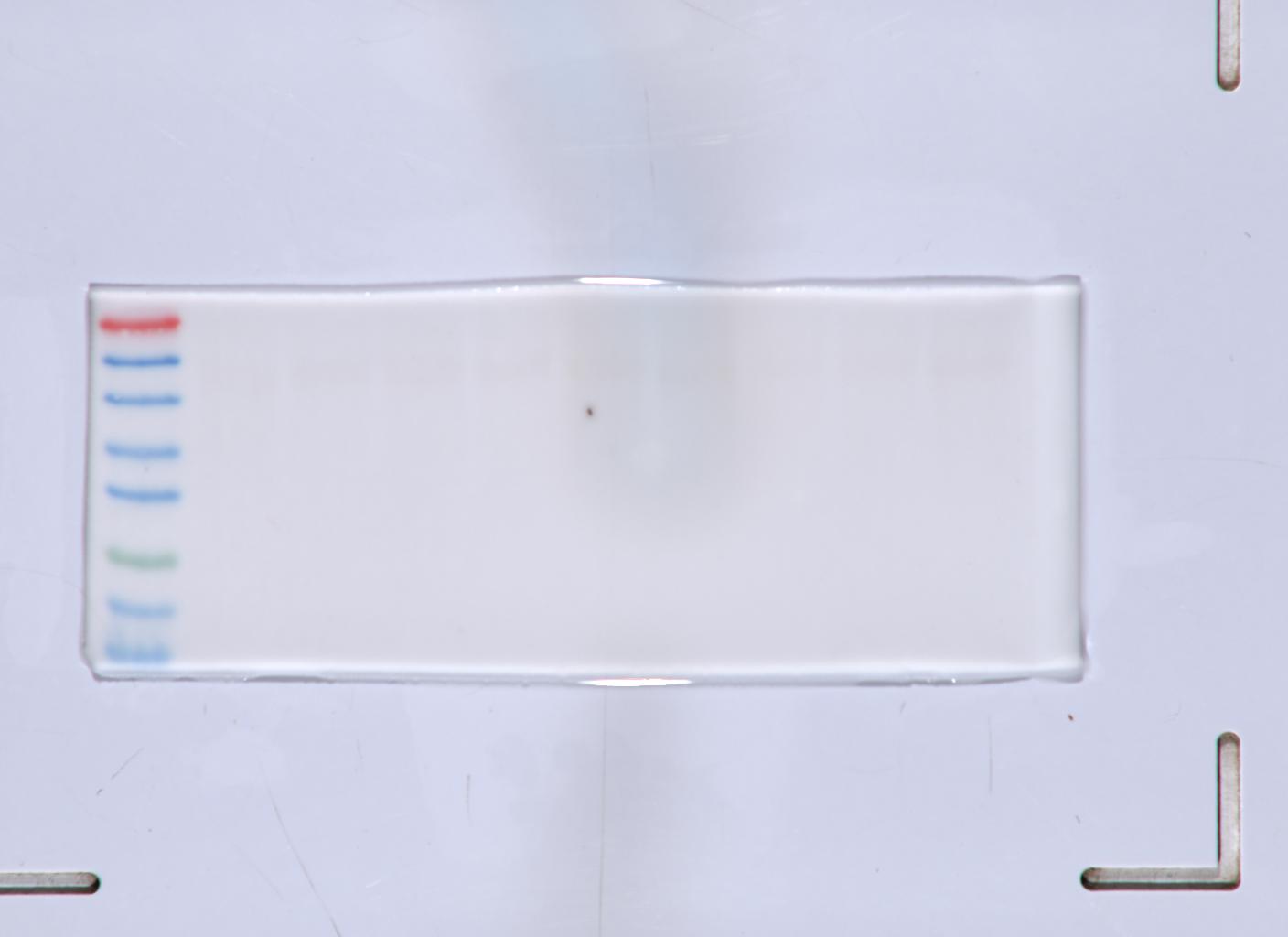

Supplement: Figure 5—source data 4. [file elife-108737-fig5-data4.zip › Figure 5—source data 4/α-flag-marker.jpg]

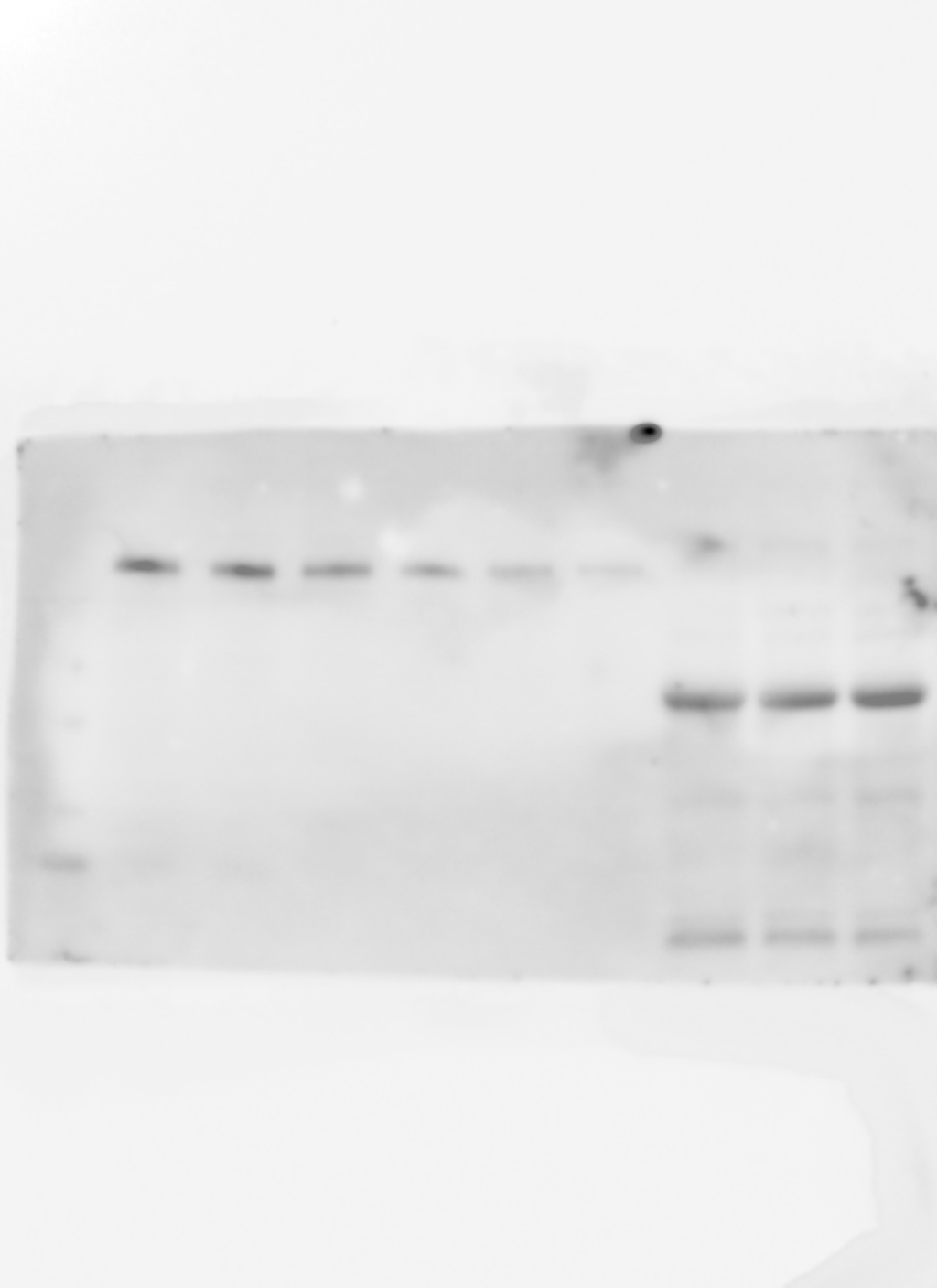

Supplement: Figure 5—source data 4. [file elife-108737-fig5-data4.zip › Figure 5—source data 4/α-myc-blot.tif]

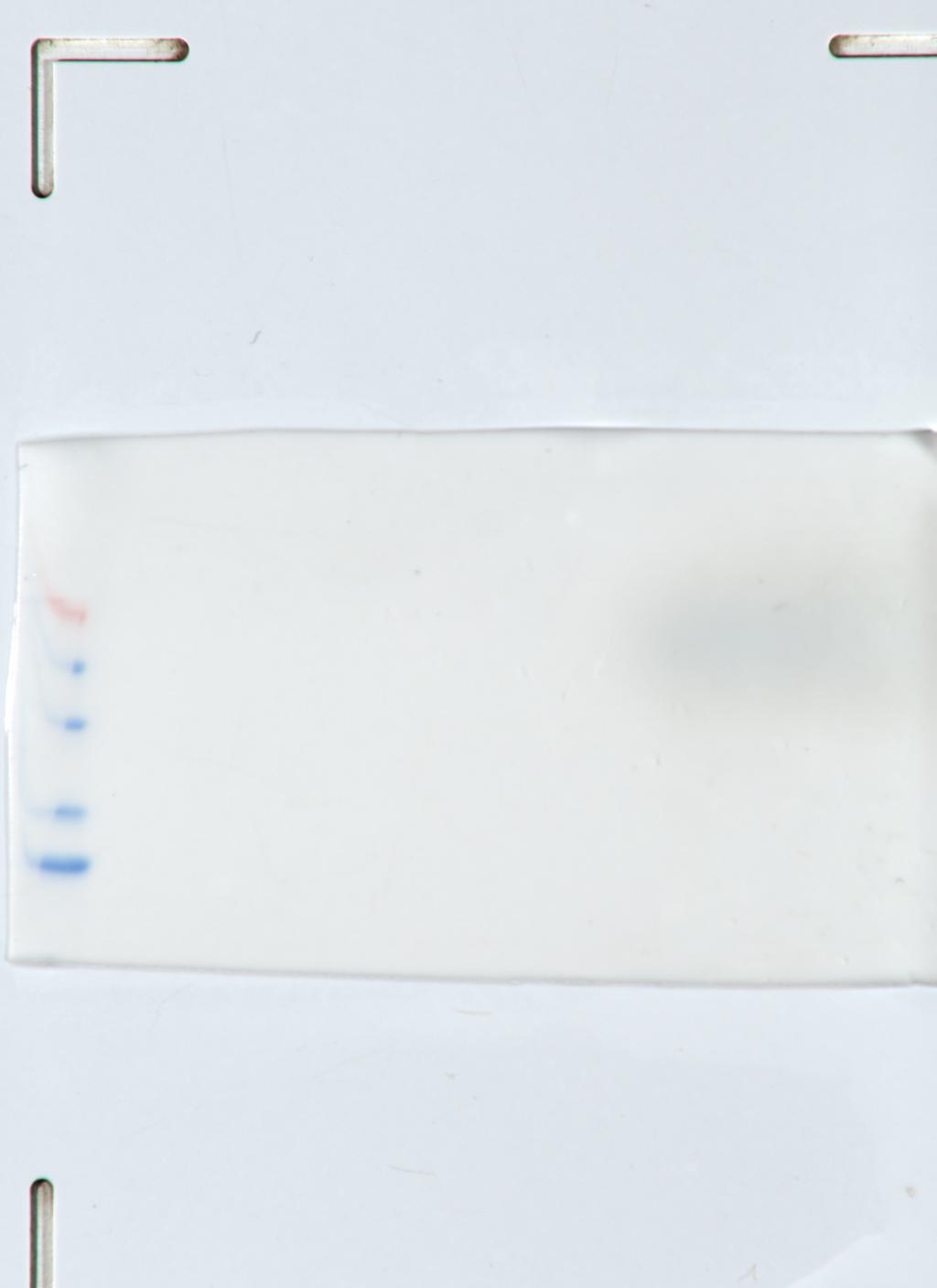

Supplement: Figure 5—source data 4. [file elife-108737-fig5-data4.zip › Figure 5—source data 4/α-myc-marker.jpg]

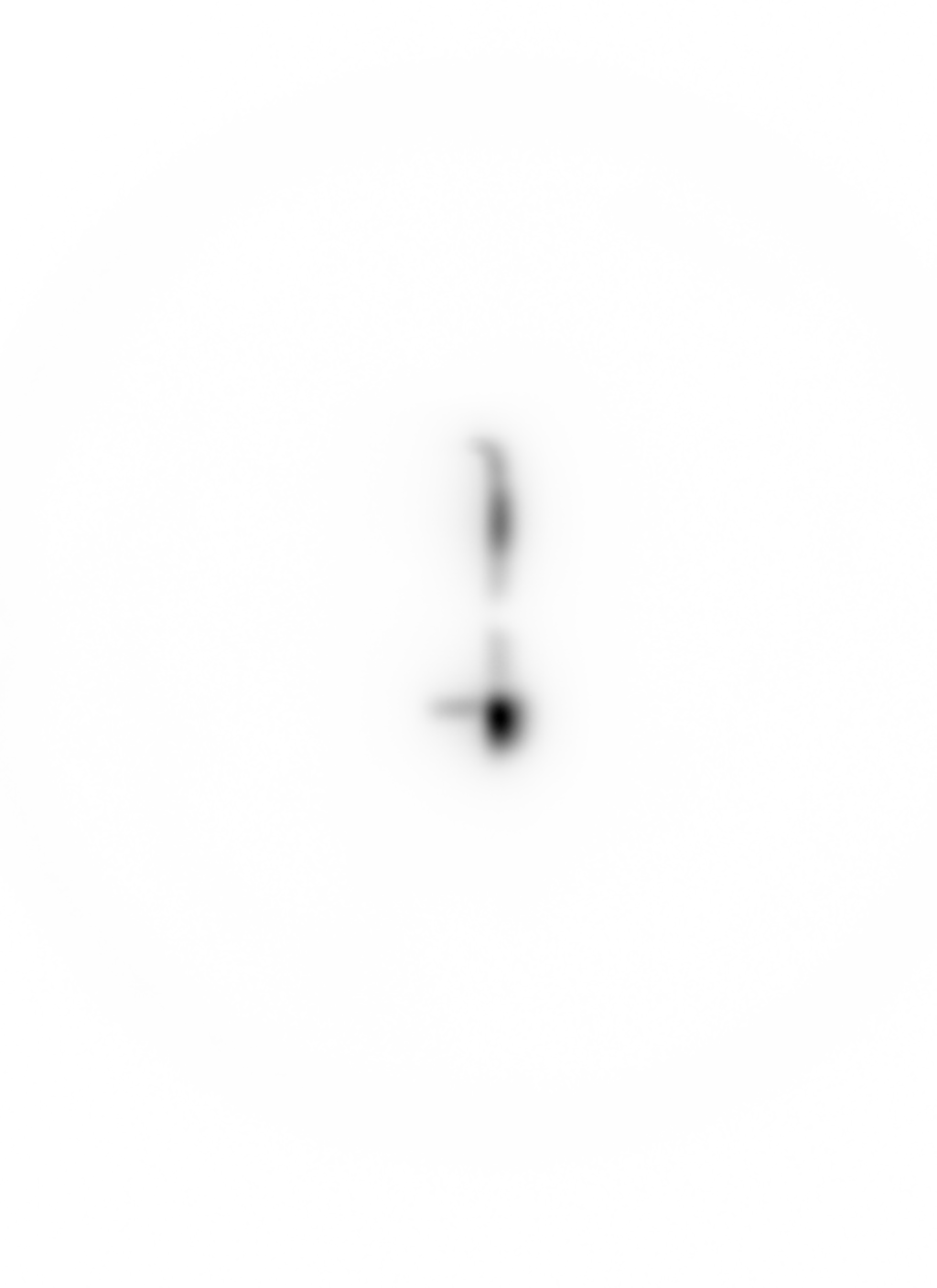

Supplement: Figure 5—figure supplement 2—source data 2. [file elife-108737-fig5-figsupp2-data2.zip › Figure 5—figure supplement 2—source data 2/input-flag-blot.tif]

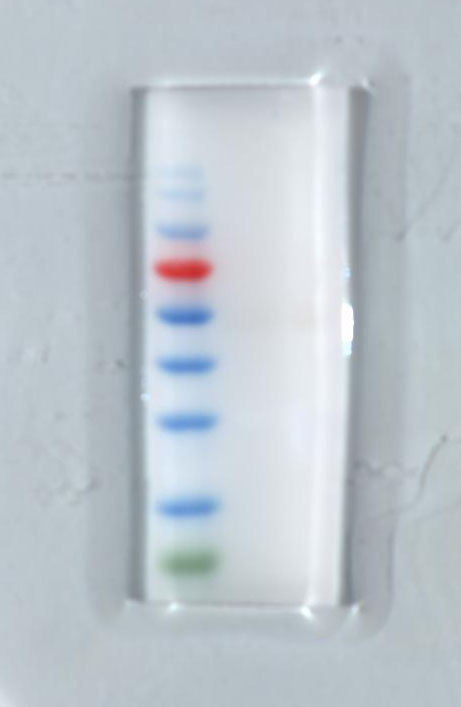

Supplement: Figure 5—figure supplement 2—source data 2. [file elife-108737-fig5-figsupp2-data2.zip › Figure 5—figure supplement 2—source data 2/input-flag-marker.jpg]

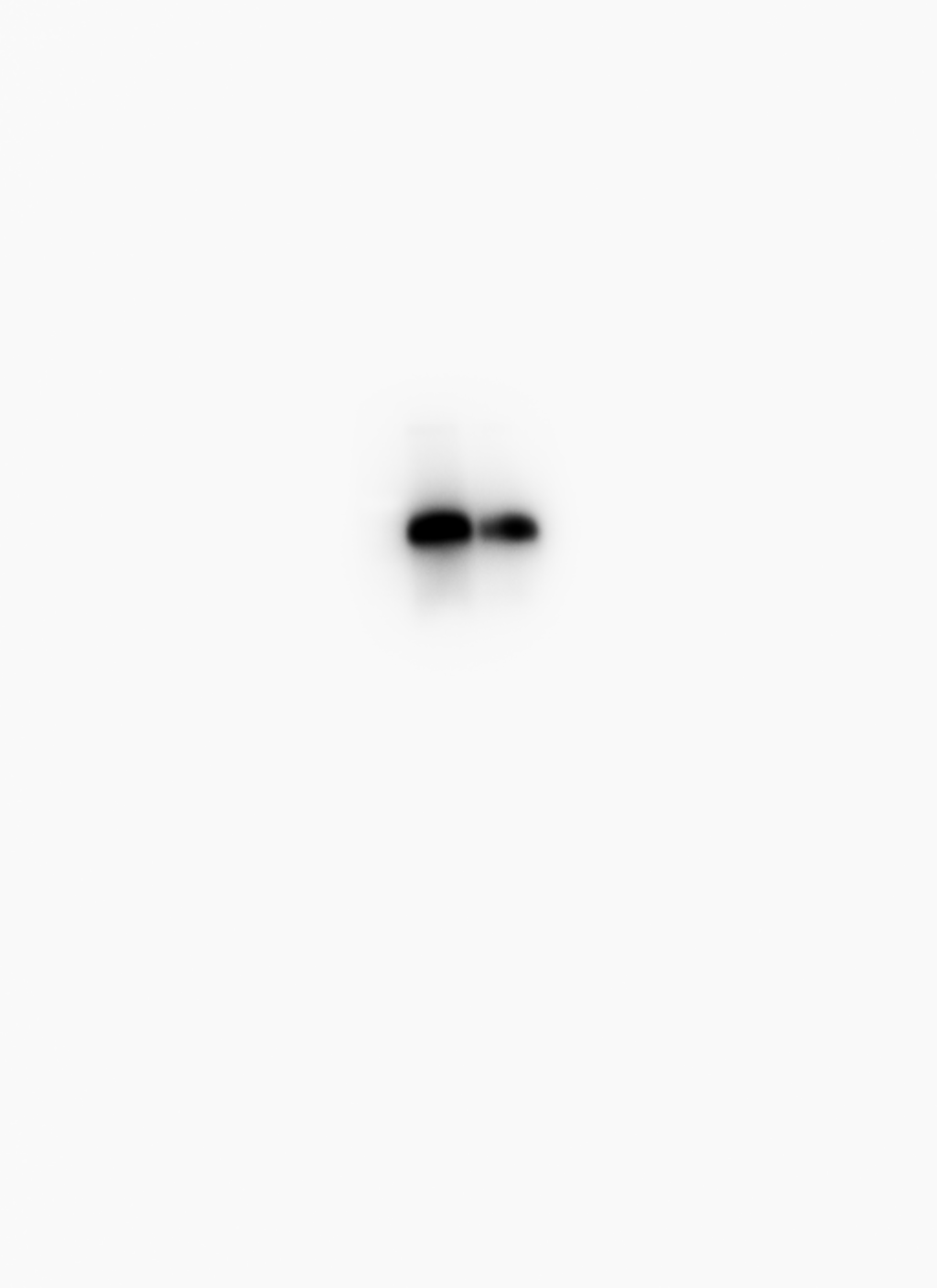

Supplement: Figure 5—figure supplement 2—source data 2. [file elife-108737-fig5-figsupp2-data2.zip › Figure 5—figure supplement 2—source data 2/input-myc-blot.tif]

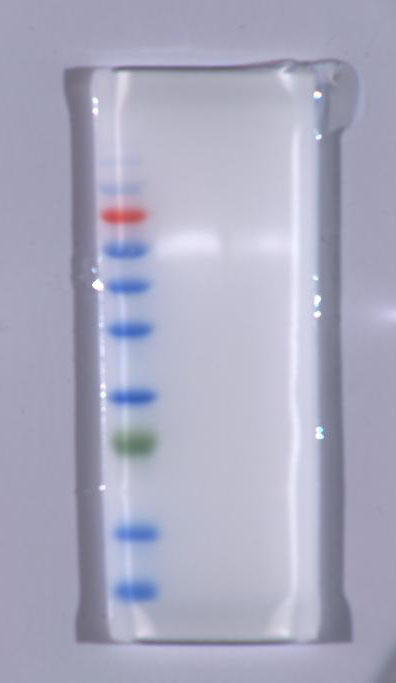

Supplement: Figure 5—figure supplement 2—source data 2. [file elife-108737-fig5-figsupp2-data2.zip › Figure 5—figure supplement 2—source data 2/input-myc-marker.jpg]

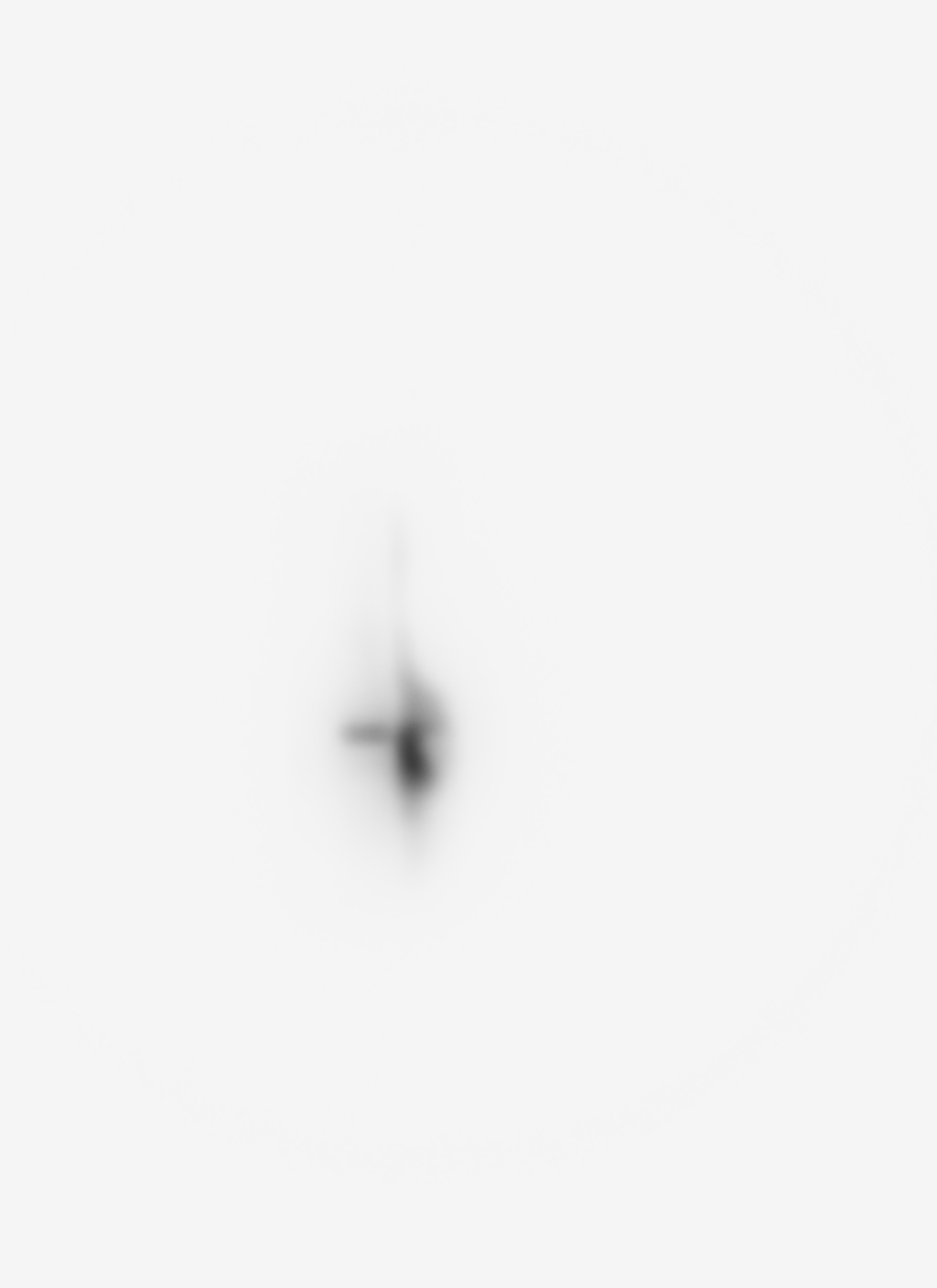

Supplement: Figure 5—figure supplement 2—source data 2. [file elife-108737-fig5-figsupp2-data2.zip › Figure 5—figure supplement 2—source data 2/IP-flag-blot.tif]

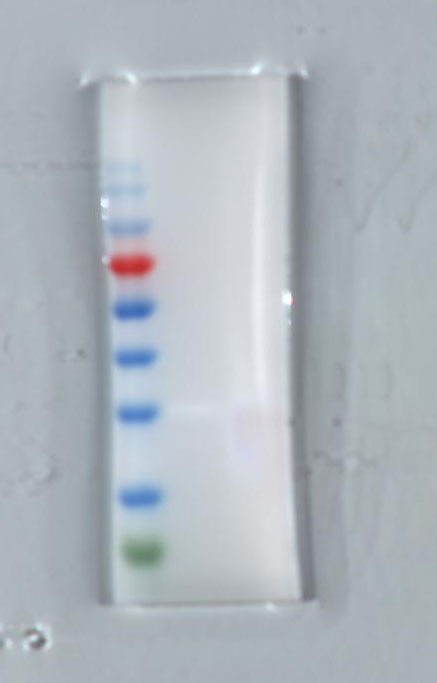

Supplement: Figure 5—figure supplement 2—source data 2. [file elife-108737-fig5-figsupp2-data2.zip › Figure 5—figure supplement 2—source data 2/IP-flag-marker.jpg]

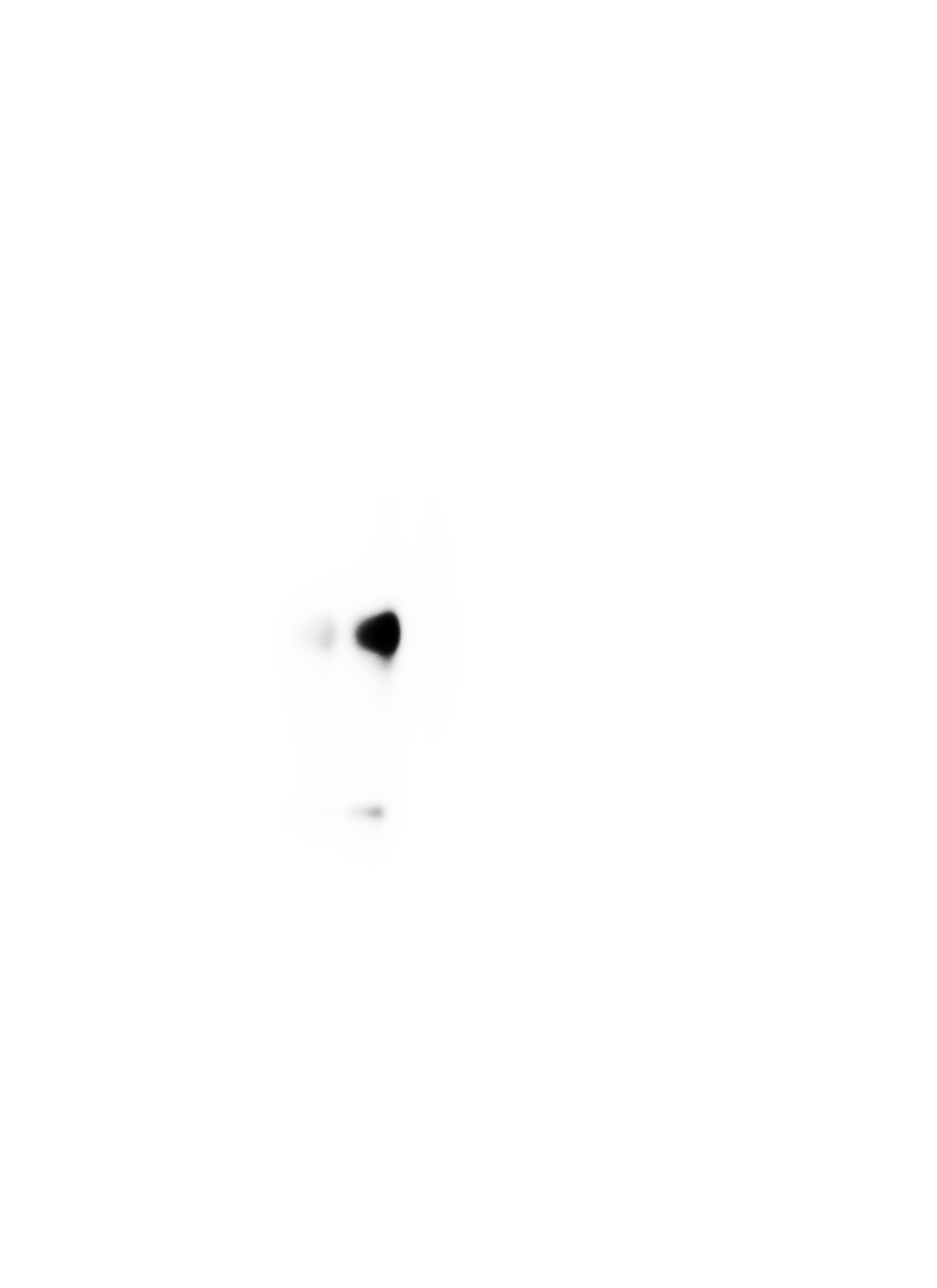

Supplement: Figure 5—figure supplement 2—source data 2. [file elife-108737-fig5-figsupp2-data2.zip › Figure 5—figure supplement 2—source data 2/IP-myc-blot.tif]

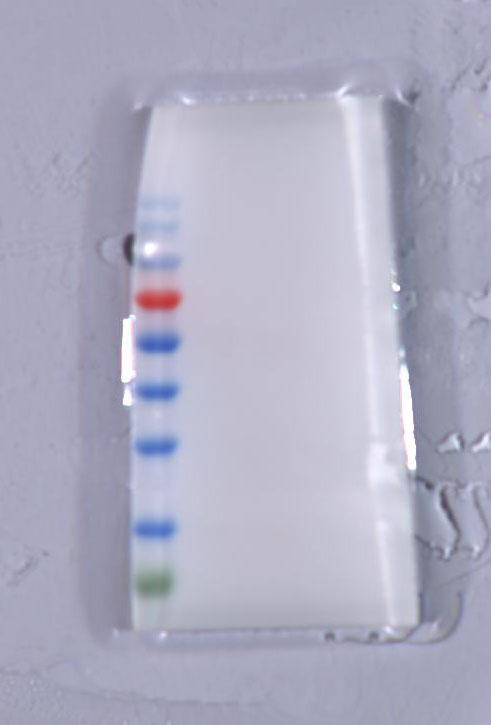

Supplement: Figure 5—figure supplement 2—source data 2. [file elife-108737-fig5-figsupp2-data2.zip › Figure 5—figure supplement 2—source data 2/IP-myc-marker.jpg]

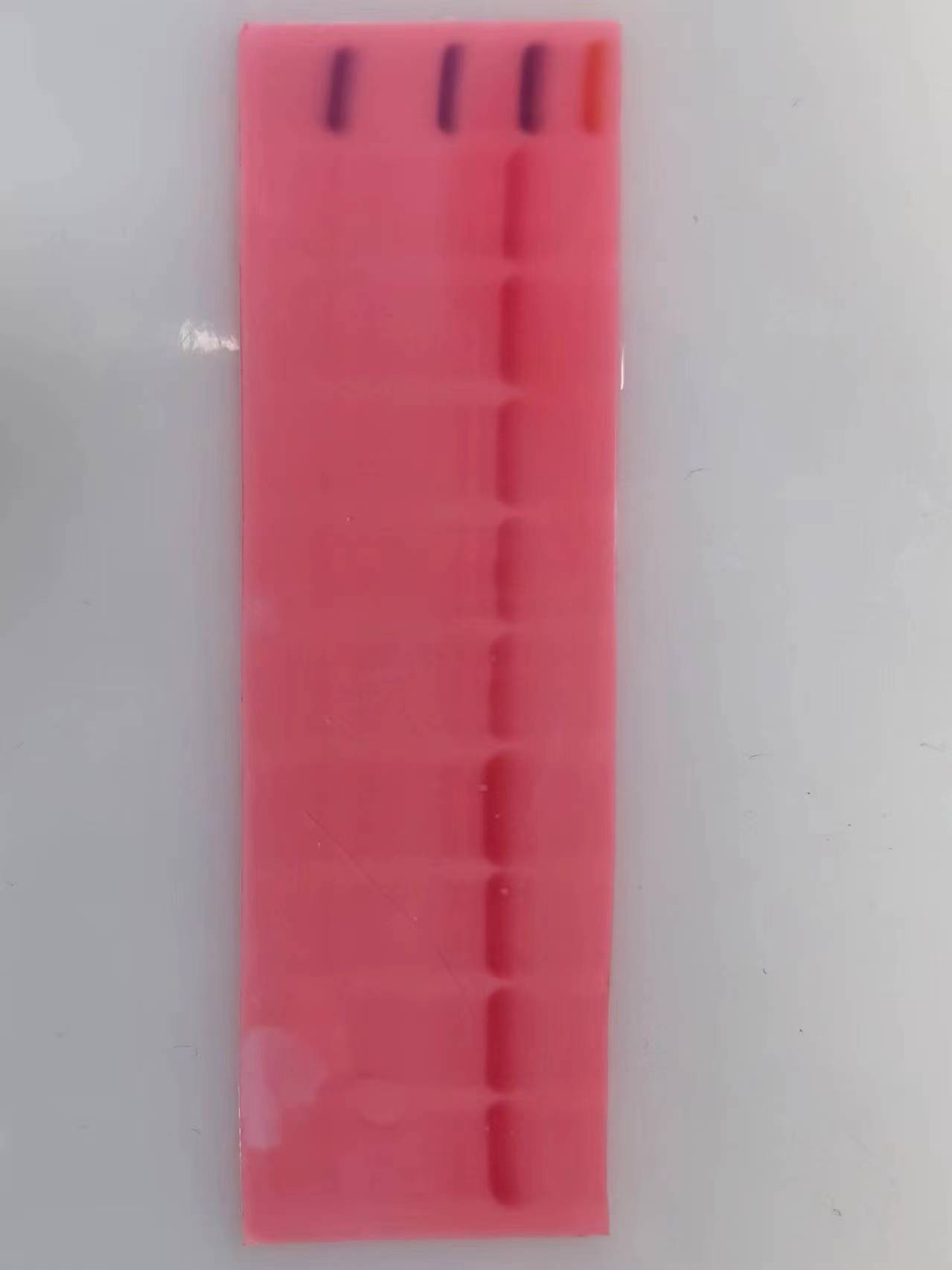

Supplement: Figure 5—figure supplement 2—source data 4. [file elife-108737-fig5-figsupp2-data4.zip › Figure 5—figure supplement 2—source data 4/RbCL.jpg]

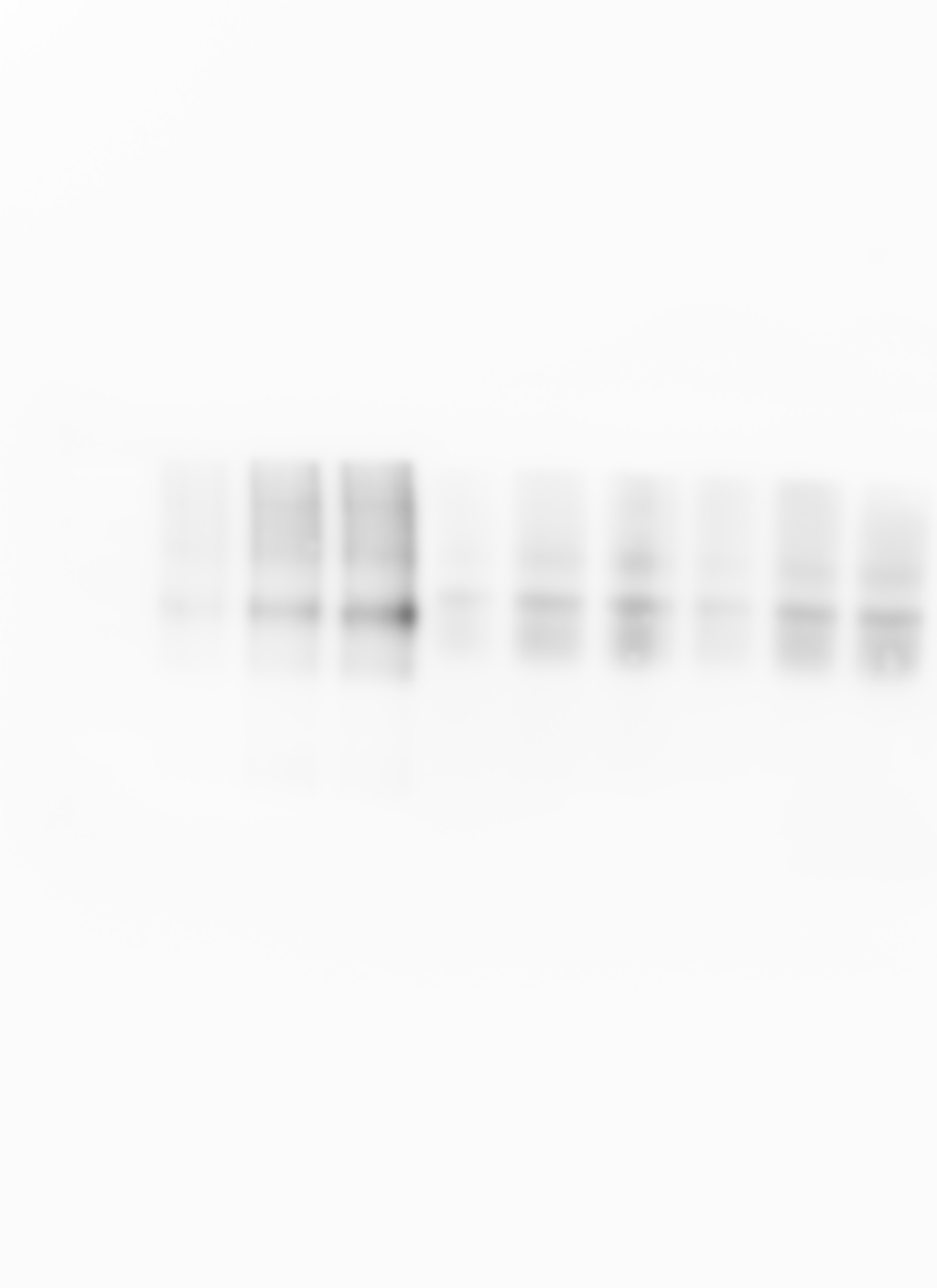

Supplement: Figure 5—figure supplement 2—source data 4. [file elife-108737-fig5-figsupp2-data4.zip › Figure 5—figure supplement 2—source data 4/α-flag-blot.tif]

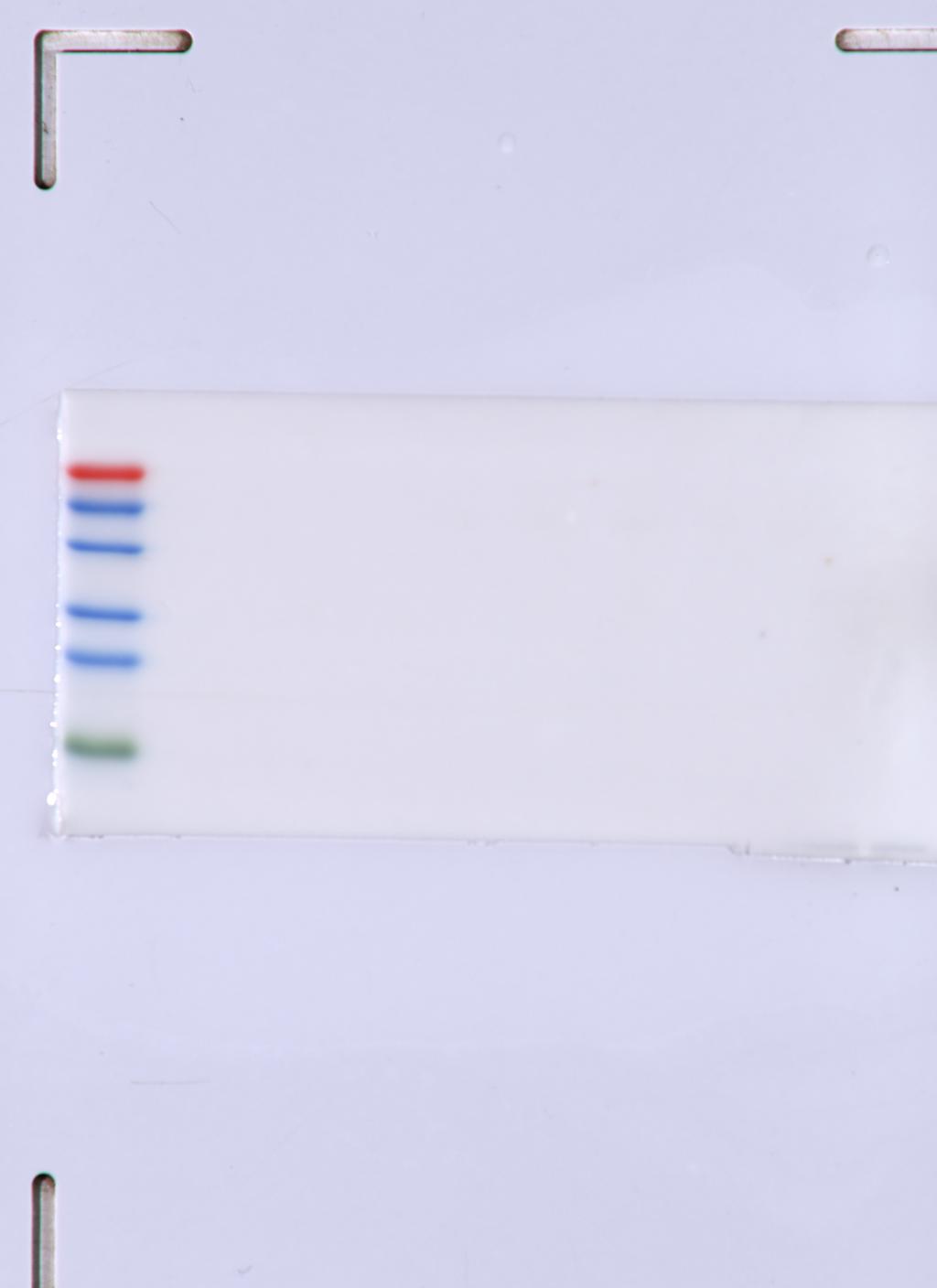

Supplement: Figure 5—figure supplement 2—source data 4. [file elife-108737-fig5-figsupp2-data4.zip › Figure 5—figure supplement 2—source data 4/α-flag-marker.jpg]

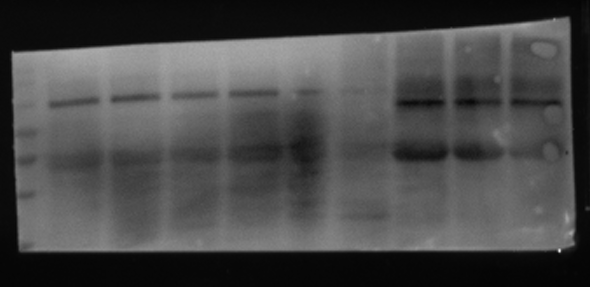

Supplement: Figure 5—figure supplement 2—source data 4. [file elife-108737-fig5-figsupp2-data4.zip › Figure 5—figure supplement 2—source data 4/α-myc.tif]
