# Supplementary material for: Herbivorous insects independently evolved salivary effectors to regulate plant immunity by destabilizing the malectin-LRR RLP NtRLP4
Source: eLife. 2026 May 5;14:RP108737. doi: 10.7554/eLife.108737 (PMC13143284; doi:10.7554/eLife.108737)
Supplement: Figure 5—source data 1. [file elife-108737-fig5-data1.zip › Figure 5—source data 1.pptx]

## Slide 1
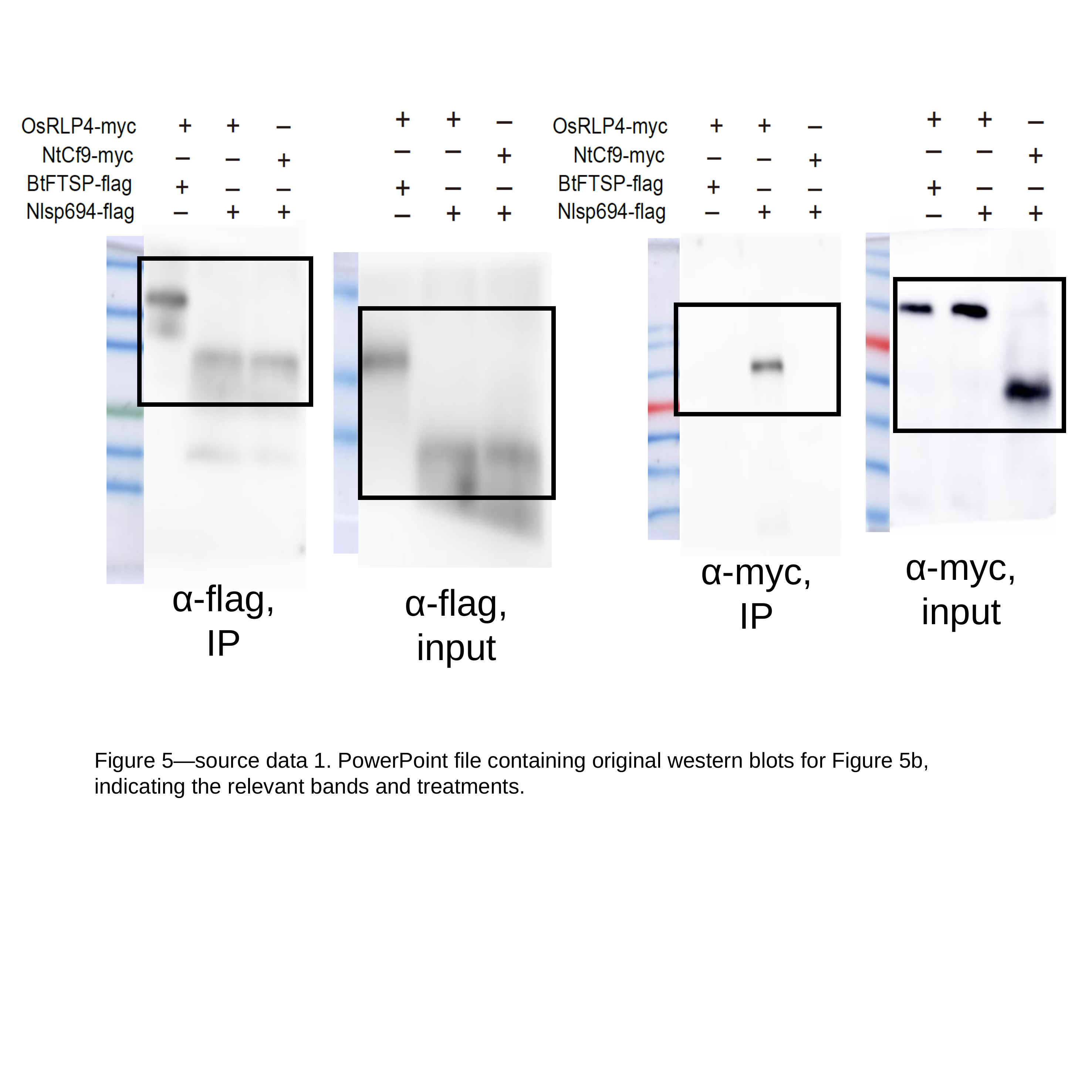

α-myc, input
α-myc, IP
α-flag, IP
α-flag, input
Figure 5—source data 1. PowerPoint file containing original western blots for Figure 5b, indicating the relevant bands and treatments.
