# Supplementary material for: Herbivorous insects independently evolved salivary effectors to regulate plant immunity by destabilizing the malectin-LRR RLP NtRLP4
Source: eLife. 2026 May 5;14:RP108737. doi: 10.7554/eLife.108737 (PMC13143284; doi:10.7554/eLife.108737)
Supplement: Supplementary file 1. — (A) Identification of RDP and SP101 homologs in insect species. (B) Proteins from a Nicotiana benthamiana cDNA library screened by yeast two-hybrid using BtRDP as a bait. (C) Differentially expressed genes between empty vector (EV) and oeRLP#1 transgenic plant. (D) Primers used in this study. [file elife-108737-supp1.docx]

Supplementary Materials for

**Herbivorous insects independently evolved salivary effectors to regulate plant immunity by destabilizing the malectin-LRR RLP4**

Xin Wang *et al.*

*Corresponding author. Jun-Min Li, lijunmin@nbu.edu.cn; Hai-Jian Huang, huanghaijian@nbu.edu.cn

**Table A.** **Identification of RDP and SP101 homologs in insect species**

| **Family** | **Species** | **Accession** | **Total Base** | **Number of RDP homologs** | **Number of SP101 homologs** | **Note** |
| --- | --- | --- | --- | --- | --- | --- |
| Aleyrodidae | *Bemisia tabaci* MED | GCA_918797505 | 609.6 MB | 1 | 0 | Assembled genome |
|  | *Bemisia tabaci* MEAM1 | GCF_001854935 | 600.6 MB | 1 | 0 | Assembled genome |
|  | *Bemisia tabaci* India | SRR1159209 | 9.7 GB* | 1 | 0 | Assembled genome |
|  | *Bemisia tabaci* SSA1 | GCA_902825415 | 657.8 MB | 1 | 0 | Assembled genome |
|  | *Bemisia tabaci* SSA2 | GCA_903994125 | 625.3 MB | 1 | 0 | Assembled genome |
|  | *Aleurocanthus spiniferus* | SRR17330024 | 9.2 GB* | 1 | 0 | RNA-seq, whole body |
|  | *Aleyrodes proletella* | SRX14998536 | 1.9 GB* | 1 | 0 | RNA-Seq, whole body |
|  | *Trialeurodes vaporariorum* | GCA_011764245 | 814.7 MB | 1 | 0 | Assembled genome |
|  | *Dialeurodes citri* | SRR2980521 | 5.0 GB* | 1 | 0 | RNA-Seq, whole body |
| Delphacidae | *Nilaparvata lugens* | GCF_014356525 | 1,088 MB | 0 | 1 | Assembled genome |
|  | *Laodelphax striatellus* | GCA_017141395 | 510.2 MB | 0 | 1 | Assembled genome |
|  | *Sogatella furcifera* | GCA_017141385 | 563.8 MB | 0 | 1 | Assembled genome |
| Aphididae | *Acyrthosiphon pisum* | GCA_005508785 | 533.6 MB | 0 | 0 | Assembled genome |
|  | *Myzus persicae* | GCA_001856785 | 347.3 MB | 0 | 0 | Assembled genome |
|  | *Sitobion miscanthi* | GCA_008086715 | 397.9 MB | 0 | 0 | Assembled genome |
| Aphalaridae | *Pachypsylla venusta* | GCA_012654025 | 482.0 MB | 0 | 0 | Assembled genome |
|  | *Diaci psyllid* | GCA_000475195 | 485.7 MB | 0 | 0 | Assembled genome |
| Alydidae | *Riptortus pedestris* | GCA_019009955 | 1.0 GB | 0 | 0 | Assembled genome |
| Lygaeidae | *Oncopeltus fasciatus* | GCA_000696205 | 1.1 GB | 0 | 0 | Assembled genome |
| Pentatomidae | *Halyomorpha halys* | GCA_000696795 | 998.2 MB | 0 | 0 | Assembled genome |
| Cimicidae | *Cimex lectularius* | GCA_000648675 | 510.8 MB | 0 | 0 | Assembled genome |
| Reduviidae | *Rhodnius prolixus* | GCA_000181055 | 706.8 MB | 0 | 0 | Assembled genome |
| Gerridae | *Gerris buenoi* | GCA_001010745 | 994.4 MB | 0 | 0 | Assembled genome |
| Drosophilidae | *Drosophila melanogaster* | GCA_002310755 | 120.4 MB | 0 | 0 | Assembled genome |
| Culicidae | *Aedes albopictus* | GCA_018104305 | 1.3 GB | 0 | 0 | Assembled genome |
| Tenebrionidae | *Tribolium castaneum* | GCA_000002335 | 165.9 MB | 0 | 0 | Assembled genome |
| Chrysomeloidea | *Anoplophora glabripennis* | GCA_000390285 | 679.8 MB | 0 | 0 | Assembled genome |
| Bombycidae | *Bombyx mori* | GCA_027366755 | 456.6 MB | 0 | 0 | Assembled genome |
| Noctuidae | *Spodoptera frugiperda* | GCA_023101765 | 383.9 MB | 0 | 0 | Assembled genome |
| Pediculidae | *Pediculus humanus* | GCA_000006295 | 110.8 MB | 0 | 0 | Assembled genome |

^*^ The total base of raw data retrieved from SRA database.

**Table B. Proteins from a *Nicotiana benthamiana* cDNA library screened by yeast two hybrid using Bt****RDP as a bait**

| ***N. benthamiana* v2.6.1 Accession** ^1^ | **Annotation** | **Number of colonies** | **Region** |
| --- | --- | --- | --- |
| Niben261Chr07g1310001.1 | Putative leucine-rich repeat receptor-like serine/threonine-protein kinase | 3 | 455-709 |
| Niben261Chr13g1396009.1 | Chloroplast stem-loop binding protein of 41 kDa b | 1 | 162-374 |
| Niben261Chr02g0086007.1 | Sorbitol dehydrogenase-like | 1 | 95-305 |
| Niben261Chr17g0179002.1 | Guanine nucleotide-binding protein subunit beta-like protein | 1 | 92-326 |
| Niben261Chr01g1673009.1 | Ribulose bisphosphate carboxylase/oxygenase activase 2 | 2 | 228-420 |
| Niben261Chr07g1128067.1 | Ribulose-1,5-bisphosphate carboxylase/oxygenase small subunit | 5 | 23-181 |
| Niben261Chr14g0161005.1 | Cytochrome b6-f complex iron-sulfur subunit 2 | 5 | 1-216 |

^1^ The sequence can be downloaded from Sol Genomics Network (https://solgenomics.net/ftp/genomes/Nicotiana_benthamianaV261)

**Table C.** **Differentially expressed genes between empty vector (EV) and oeRLP#1 transgenic plant.**

| **Gene ID** | **oeRLP#1** | | | | **EV** | | | | **Description** |
| --- | --- | --- | --- | --- | --- | --- | --- | --- | --- |
|  | **R1** | **R2** | **R3** | **R4** | **R1** | **R2** | **R3** | **R4** |  |
| Nitab4.5_0000757g0070 | 22 | 11 | 8 | 14 | 0 | 0 | 0 | 0 | SRC2-like protein |
| Nitab4.5_0000014g0130 | 0 | 0 | 4 | 7 | 0 | 0 | 0 | 0 | Unknown Protein |
| Nitab4.5_0001143g0060 | 3 | 6 | 1 | 0 | 0 | 0 | 0 | 0 | PBSP domain-containing protein |
| Nitab4.5_0002689g0030 | 3 | 1 | 1 | 9 | 0 | 0 | 0 | 0 | Non-structural maintenance of chromosome element 4 |
| Nitab4.5_0000171g0100 | 3 | 1 | 5 | 1 | 0 | 0 | 0 | 0 | Two-component response regulator ARR3 |
| Nitab4.5_0000344g0220 | 3 | 0 | 0 | 7 | 0 | 0 | 0 | 0 | LEA-like protein |
| Nitab4.5_0000108g0090 | 7 | 8 | 9 | 7 | 0 | 0 | 0 | 0 | Receptor serine_threonine kinase |
| Nitab4.5_0003282g0020 | 3 | 1 | 3 | 2 | 0 | 0 | 0 | 0 | RLK, Receptor like protein, putative resistance protein with an antifungal domain |
| Nitab4.5_0000287g0160 | 1 | 1 | 2 | 4 | 0 | 0 | 0 | 0 | BHLH transcription factor-like |
| Nitab4.5_0004038g0030 | 2 | 0 | 1 | 4 | 0 | 0 | 0 | 0 | -- |
| Nitab4.5_0001715g0030 | 0 | 0 | 2 | 4 | 0 | 0 | 0 | 0 | Os12g0114200 protein |
| Nitab4.5_0002380g0020 | 1 | 0 | 4 | 3 | 0 | 0 | 0 | 0 | -- |
| Nitab4.5_0000516g0160 | 5 | 1 | 0 | 5 | 0 | 0 | 1 | 0 | Dof zinc finger protein |
| Nitab4.5_0003395g0030 | 5 | 6 | 1 | 3 | 0 | 1 | 0 | 0 | SNARE associated Golgi protein |
| Nitab4.5_0002123g0050 | 2 | 1 | 1 | 2 | 0 | 0 | 0 | 0 | Beta Galactosidase-like protein |
| Nitab4.5_0001286g0080 | 2 | 2 | 2 | 0 | 0 | 0 | 0 | 0 | Alpha mannosidase-like protein |
| Nitab4.5_0001146g0240 | 2 | 1 | 1 | 2 | 0 | 0 | 0 | 0 | Unknown Protein |
| Nitab4.5_0000171g0070 | 2 | 10 | 3 | 2 | 1 | 0 | 0 | 0 | U-box domain-containing protein 13 |
| Nitab4.5_0003558g0030 | 10 | 3 | 1 | 3 | 1 | 0 | 0 | 0 | Zinc finger (Ran-binding) family protein |
| Nitab4.5_0000068g0120 | 0 | 2 | 3 | 0 | 0 | 0 | 0 | 0 | Dof zinc finger protein |
| Nitab4.5_0000725g0100 | 7 | 3 | 2 | 2 | 1 | 0 | 0 | 0 | UDP-glucosyltransferase family 1 protein |
| Nitab4.5_0000282g0040 | 4 | 2 | 2 | 4 | 0 | 1 | 0 | 0 | Cohesin subunit |
| Nitab4.5_0000458g0090 | 0 | 1 | 2 | 2 | 0 | 0 | 0 | 0 | Blue copper protein |
| Nitab4.5_0000856g0230 | 2 | 3 | 2 | 0 | 0 | 0 | 0 | 0 | Methyltransferase-like protein 6 |
| Nitab4.5_0002244g0110 | 5 | 5 | 4 | 0 | 0 | 0 | 0 | 1 | Isopentenyl-diphosphate delta-isomerase family protein |
| Nitab4.5_0000165g0050 | 4 | 1 | 3 | 0 | 0 | 0 | 0 | 0 | RNA-dependent RNA polymerase family protein |
| Nitab4.5_0000231g0180 | 2 | 2 | 0 | 0 | 0 | 0 | 0 | 0 | Hydrolase alpha_beta fold family protein |
| Nitab4.5_0004261g0030 | 12 | 11 | 12 | 1 | 2 | 0 | 1 | 0 | LRR receptor-like serine_threonine-protein kinase, RLP |
| Nitab4.5_0000705g0010 | 11 | 5 | 6 | 4 | 1 | 1 | 1 | 0 | Receptor like protein kinase |
| Nitab4.5_0000223g0340 | 9 | 9 | 3 | 1 | 1 | 1 | 1 | 0 | WRKY transcription factor-30 |
| Nitab4.5_0002100g0100 | 3 | 0 | 0 | 2 | 0 | 0 | 0 | 0 | N-hydroxycinnamoyl_benzoyltransferase 1 |
| Nitab4.5_0000213g0080 | 4 | 0 | 0 | 2 | 0 | 0 | 0 | 0 | Integrin-linked kinase-associated serine_threonine phosphatase 2C |
| Nitab4.5_0000274g0230 | 3 | 6 | 0 | 1 | 1 | 0 | 0 | 0 | RING finger protein 6 |
| Nitab4.5_0002177g0170 | 11 | 9 | 8 | 2 | 2 | 0 | 0 | 1 | Receptor like kinase, RLK |
| Nitab4.5_0003169g0020 | 9 | 2 | 0 | 4 | 0 | 0 | 1 | 1 | Disease resistance response |
| Nitab4.5_0000200g0030 | 1 | 7 | 4 | 2 | 0 | 0 | 0 | 1 | Aldose 1-epimerase family protein |
| Nitab4.5_0000529g0080 | 5 | 1 | 3 | 7 | 2 | 0 | 0 | 0 | Calmodulin |
| Nitab4.5_0001210g0030 | 2 | 5 | 0 | 1 | 1 | 0 | 0 | 0 | Unknown Protein |
| Nitab4.5_0003051g0020 | 25 | 22 | 23 | 5 | 5 | 0 | 1 | 2 | NBS-LRR class disease resistance protein |
| Nitab4.5_0000856g0110 | 1 | 3 | 2 | 3 | 0 | 0 | 0 | 0 | U-box domain-containing protein |
| Nitab4.5_0003558g0070 | 18 | 17 | 22 | 15 | 5 | 0 | 0 | 3 | Zinc finger (Ran-binding) family protein |
| Nitab4.5_0000495g0060 | 2 | 5 | 2 | 7 | 0 | 0 | 0 | 1 | Ring finger protein |
| Nitab4.5_0002444g0010 | 0 | 3 | 2 | 0 | 0 | 0 | 0 | 0 | -- |
| Nitab4.5_0001863g0220 | 8 | 7 | 9 | 6 | 1 | 2 | 0 | 0 | Genomic DNA chromosome 3 TAC clone K1G2 |
| Nitab4.5_0002221g0060 | 4 | 4 | 1 | 7 | 0 | 0 | 0 | 2 | Cytochrome P450 |
| Nitab4.5_0000246g0210 | 1 | 5 | 0 | 2 | 0 | 1 | 0 | 0 | Pollen allergen Phl p 11 |
| Nitab4.5_0000020g0210 | 2 | 0 | 1 | 2 | 0 | 0 | 0 | 0 | -- |
| Nitab4.5_0001794g0030 | 7 | 8 | 3 | 8 | 0 | 0 | 3 | 0 | Flavoprotein wrbA |
| Nitab4.5_0002156g0030 | 7 | 3 | 1 | 0 | 0 | 1 | 0 | 1 | Genomic DNA chromosome 5 P1 clone MQD19 |
| Nitab4.5_0000462g0110 | 17 | 6 | 11 | 16 | 1 | 1 | 2 | 3 | Receptor like kinase, RLK |
| Nitab4.5_0002859g0040 | 2 | 0 | 4 | 0 | 0 | 0 | 0 | 0 | Dynein light chain 1 cytoplasmic |
| Nitab4.5_0000131g0130 | 7 | 4 | 2 | 10 | 0 | 0 | 1 | 2 | LRR receptor-like serine_threonine-protein kinase, RLP |
| Nitab4.5_0000278g0050 | 2 | 2 | 1 | 6 | 0 | 0 | 1 | 1 | Os12g0581300 protein (Fragment) |
| Nitab4.5_0004821g0030 | 3 | 4 | 4 | 3 | 0 | 0 | 0 | 1 | Zinc finger family protein |
| Nitab4.5_0006338g0090 | 7 | 0 | 3 | 1 | 0 | 0 | 0 | 1 | Ring H2 finger protein |
| Nitab4.5_0002816g0080 | 6 | 1 | 2 | 3 | 1 | 0 | 0 | 1 | Os04g0461600 protein (Fragment) |
| Nitab4.5_0000028g0360 | 6 | 6 | 9 | 0 | 3 | 0 | 0 | 0 | Unknown Protein |
| Nitab4.5_0000125g0360 | 2 | 4 | 6 | 8 | 1 | 2 | 0 | 0 | Unknown Protein |
| Nitab4.5_0000200g0100 | 6 | 4 | 3 | 4 | 0 | 0 | 0 | 2 | WRKY transcription factor 23 |
| Nitab4.5_0000010g0270 | 1 | 2 | 3 | 2 | 0 | 1 | 0 | 0 | Receptor like kinase, RLK |
| Nitab4.5_0000187g0090 | 3 | 5 | 2 | 2 | 0 | 0 | 1 | 0 | Tumor susceptibility protein 101 (Fragment) |
| Nitab4.5_0000040g0670 | 2 | 2 | 0 | 1 | 0 | 0 | 0 | 0 | GHMP kinase family protein |
| Nitab4.5_0003542g0020 | 11 | 15 | 17 | 0 | 1 | 2 | 3 | 1 | Receptor like kinase, RLK |
| Nitab4.5_0000621g0030 | 15 | 21 | 6 | 27 | 0 | 3 | 6 | 2 | Glutaredoxin |
| Nitab4.5_0002114g0080 | 0 | 2 | 4 | 3 | 0 | 0 | 0 | 0 | Ras-related protein Rab-25 |
| Nitab4.5_0000098g0150 | 11 | 8 | 3 | 16 | 1 | 0 | 0 | 4 | Unknown Protein |
| Nitab4.5_0004136g0020 | 2 | 2 | 3 | 1 | 0 | 0 | 1 | 0 |  |
| Nitab4.5_0004197g0020 | 2 | 3 | 4 | 33 | 4 | 2 | 0 | 1 | Patatin-like phospholipase domain-containing protein c |
| Nitab4.5_0000617g0080 | 2 | 4 | 4 | 3 | 1 | 0 | 0 | 1 | Nuclear nucleic acid-binding protein C1D |
| Nitab4.5_0001204g0110 | 2 | 4 | 1 | 1 | 0 | 1 | 0 | 0 | NAC domain transcription factor protein |
| Nitab4.5_0000825g0090 | 15 | 8 | 10 | 5 | 4 | 1 | 0 | 1 | Receptor kinase |
| Nitab4.5_0002905g0040 | 22 | 24 | 14 | 7 | 6 | 5 | 0 | 0 | Unknown Protein |
| Nitab4.5_0001662g0030 | 20 | 21 | 14 | 31 | 4 | 1 | 1 | 8 | Gibberellin 2-oxidase 2 |
| Nitab4.5_0000352g0090 | 3 | 0 | 6 | 3 | 0 | 1 | 1 | 0 | Coatomer alpha subunit-like protein |
| Nitab4.5_0000080g0230 | 1 | 2 | 3 | 2 | 0 | 0 | 0 | 1 | 5_apos-3_apos exoribonuclease 2 |
| Nitab4.5_0005052g0030 | 26 | 21 | 21 | 4 | 7 | 2 | 1 | 2 | Receptor like kinase, RLK |
| Nitab4.5_0000305g0140 | 2 | 3 | 1 | 4 | 0 | 0 | 1 | 0 | Ethylene-responsive transcription factor 4 |
| Nitab4.5_0000440g0150 | 2 | 5 | 2 | 5 | 1 | 0 | 1 | 0 | 2,3-bisphosphoglycerate-dependent phosphoglycerate mutase |
| Nitab4.5_0000258g0120 | 17 | 6 | 7 | 4 | 1 | 1 | 3 | 0 | Cyclic nucleotide gated channel |
| Nitab4.5_0006460g0020 | 11 | 9 | 7 | 6 | 0 | 0 | 0 | 5 | Unknown Protein |
| Nitab4.5_0000753g0120 | 1 | 3 | 4 | 3 | 0 | 0 | 1 | 1 | Unknown Protein |
| Nitab4.5_0000976g0090 | 1 | 4 | 4 | 2 | 0 | 0 | 1 | 0 | FLORICAULA_LEAFY-like protein |
| Nitab4.5_0000008g0870 | 18 | 27 | 13 | 29 | 4 | 5 | 1 | 5 | Hydroxycinnamoyl transferase |
| Nitab4.5_0001232g0030 | 7 | 19 | 2 | 13 | 0 | 3 | 4 | 0 | Unknown Protein |
| Nitab4.5_0001213g0030 | 2 | 2 | 0 | 3 | 0 | 0 | 0 | 1 | LRR receptor-like serine_threonine-protein kinase, RLP |
| Nitab4.5_0000444g0090 | 18 | 13 | 17 | 6 | 4 | 3 | 1 | 2 | Receptor-like protein kinase At3g21340 |
| Nitab4.5_0005936g0030 | 4 | 10 | 6 | 12 | 1 | 1 | 0 | 4 | F-box family protein |
| Nitab4.5_0000519g0380 | 8 | 0 | 2 | 8 | 0 | 2 | 1 | 0 | Transportin |
| Nitab4.5_0000188g0120 | 8 | 2 | 3 | 3 | 1 | 1 | 0 | 0 | Armadillo_beta-catenin repeat family protein |
| Nitab4.5_0000038g0030 | 8 | 14 | 13 | 16 | 2 | 0 | 6 | 2 | Cysteine-rich receptor-like protein kinase |
| Nitab4.5_0003514g0020 | 8 | 12 | 14 | 2 | 3 | 2 | 1 | 0 | Receptor-like protein kinase At3g21340 |
| Nitab4.5_0000344g0120 | 12 | 14 | 7 | 6 | 3 | 1 | 1 | 2 | Cell division protease ftsH homolog 3 |
| Nitab4.5_0000041g0370 | 2 | 2 | 2 | 1 | 0 | 0 | 1 | 0 | Acetyl esterase |
| Nitab4.5_0003443g0050 | 3 | 4 | 2 | 1 | 0 | 0 | 0 | 1 | Hypoxanthine phosphoribosyltransferase |
| Nitab4.5_0000249g0390 | 1 | 3 | 1 | 3 | 0 | 0 | 0 | 1 | Homeodomain-like |
| Nitab4.5_0000517g0040 | 9 | 4 | 4 | 2 | 1 | 1 | 0 | 1 | ELF4-like protein |
| Nitab4.5_0001438g0020 | 5 | 4 | 3 | 1 | 0 | 1 | 1 | 0 | Receptor-like protein kinase |
| Nitab4.5_0002177g0100 | 1 | 2 | 3 | 1 | 0 | 0 | 1 | 0 | Receptor like kinase, RLK |
| Nitab4.5_0002783g0040 | 1 | 2 | 2 | 6 | 0 | 0 | 0 | 2 | Polygalacturonase |
| Nitab4.5_0004728g0070 | 3 | 1 | 1 | 3 | 0 | 0 | 0 | 1 | Mitochondrial phosphate carrier protein |
| Nitab4.5_0000286g0030 | 6 | 5 | 4 | 2 | 1 | 1 | 1 | 0 | Ankyrin repeat protein |
| Nitab4.5_0003558g0090 | 16 | 0 | 18 | 3 | 1 | 0 | 5 | 2 | Zinc finger (Ran-binding) family protein |
| Nitab4.5_0000716g0240 | 9 | 2 | 9 | 4 | 2 | 3 | 0 | 0 | Unknown Protein |
| Nitab4.5_0001896g0050 | 2 | 1 | 3 | 2 | 0 | 1 | 0 | 0 | Os06g0220000 protein (Fragment) |
| Nitab4.5_0000082g0400 | 2 | 2 | 6 | 20 | 2 | 1 | 1 | 2 | Patatin-like phospholipase domain-containing protein c |
| Nitab4.5_0006826g0060 | 3 | 12 | 6 | 5 | 2 | 3 | 1 | 0 |  |
| Nitab4.5_0003551g0110 | 5 | 3 | 4 | 19 | 4 | 1 | 0 | 1 | Unknown Protein |
| Nitab4.5_0001302g0090 | 3 | 1 | 1 | 2 | 1 | 1 | 0 | 0 | Glycosyltransferase-like protein |
| Nitab4.5_0000462g0120 | 13 | 14 | 10 | 18 | 5 | 1 | 3 | 2 | Receptor like kinase, RLK |
| Nitab4.5_0001608g0050 | 6 | 10 | 14 | 4 | 4 | 1 | 1 | 1 | Cathepsin B-like cysteine proteinase |
| Nitab4.5_0001048g0080 | 0 | 2 | 2 | 1 | 0 | 1 | 0 | 0 | ATP-dependent RNA helicase |
| Nitab4.5_0000404g0060 | 12 | 7 | 6 | 16 | 2 | 2 | 1 | 3 | Unknown Protein |
| Nitab4.5_0001461g0070 | 23 | 17 | 6 | 6 | 5 | 2 | 1 | 2 | cytochrome P450 |
| Nitab4.5_0004692g0020 | 2 | 2 | 5 | 4 | 0 | 0 | 2 | 1 | RING zinc finger-containing protein |
| Nitab4.5_0004732g0010 | 8 | 11 | 5 | 1 | 4 | 1 | 1 | 0 | Receptor-like kinase |
| Nitab4.5_0000262g0190 | 1 | 1 | 3 | 9 | 1 | 0 | 1 | 1 | Pectinesterase |
| Nitab4.5_0003913g0040 | 10 | 9 | 6 | 10 | 1 | 1 | 2 | 3 | Subtilisin-like protease |
| Nitab4.5_0000153g0200 | 3 | 5 | 2 | 5 | 0 | 1 | 3 | 0 |  |
| Nitab4.5_0000747g0020 | 2 | 3 | 6 | 1 | 1 | 1 | 0 | 1 | Serine_threonine-protein kinase receptor |
| Nitab4.5_0000232g0330 | 4 | 1 | 2 | 0 | 1 | 1 | 0 | 0 | Receptor like kinase, RLK |
| Nitab4.5_0000174g0170 | 24 | 30 | 12 | 14 | 12 | 2 | 4 | 1 | Unknown Protein |
| Nitab4.5_0000370g0110 | 1 | 0 | 3 | 2 | 0 | 0 | 0 | 0 | Aldose 1-epimerase family protein |
| Nitab4.5_0001439g0050 | 5 | 2 | 3 | 3 | 1 | 1 | 0 | 1 | Cytochrome P450 |
| Nitab4.5_0000262g0080 | 2 | 6 | 5 | 5 | 2 | 1 | 0 | 1 | 8-amino-7-oxononanoate synthase-like protein |
| Nitab4.5_0004800g0070 | 3 | 3 | 1 | 3 | 2 | 0 | 1 | 0 | F-box_kelch-repeat protein At1g22040 |
| Nitab4.5_0000980g0260 | 0 | 2 | 3 | 2 | 1 | 0 | 0 | 0 | Neutral invertase like protein |
| Nitab4.5_0002285g0010 | 8 | 5 | 5 | 3 | 1 | 1 | 2 | 1 | Nodulin-like protein (Fragment) |
| Nitab4.5_0004506g0120 | 10 | 7 | 11 | 3 | 3 | 1 | 1 | 2 | Cyclic nucleotide gated channel |
| Nitab4.5_0004657g0030 | 68 | 50 | 39 | 150 | 20 | 10 | 21 | 23 | Auxin response factor 14 |
| Nitab4.5_0002328g0020 | 15 | 16 | 17 | 13 | 6 | 4 | 1 | 3 | Heat stress transcription factor-type, DNA-binding |
| Nitab4.5_0000676g0220 | 3 | 0 | 3 | 3 | 0 | 1 | 1 | 1 | Myb family transcription factor |
| Nitab4.5_0002321g0060 | 5 | 7 | 3 | 4 | 1 | 0 | 3 | 0 | NAC domain protein IPR003441 protein |
| Nitab4.5_0000892g0040 | 3 | 2 | 3 | 1 | 0 | 0 | 0 | 1 | Receptor-like protein kinase |
| Nitab4.5_0000146g0180 | 5 | 5 | 1 | 2 | 2 | 1 | 0 | 0 | Oligopeptidase (Protease II) |
| Nitab4.5_0002916g0060 | 10 | 10 | 6 | 14 | 4 | 1 | 4 | 1 | 1-aminocyclopropane-1-carboxylate oxidase-like protein |
| Nitab4.5_0000441g0240 | 12 | 6 | 5 | 8 | 4 | 2 | 0 | 2 | Membrane protein |
| Nitab4.5_0004625g0010 | 5 | 3 | 6 | 1 | 2 | 1 | 0 | 0 | Serine_threonine-protein kinase receptor |
| Nitab4.5_0001143g0050 | 45 | 43 | 25 | 52 | 3 | 9 | 10 | 20 | PBSP domain-containing protein |
| Nitab4.5_0004490g0050 | 4 | 2 | 2 | 3 | 0 | 1 | 0 | 1 | 3-deoxy-D-manno-octulosonic acid transferase-like protein |
| Nitab4.5_0000028g0180 | 134 | 78 | 91 | 41 | 49 | 14 | 14 | 11 | Blue copper protein (Fragment) |
| Nitab4.5_0000989g0020 | 12 | 9 | 5 | 7 | 2 | 1 | 2 | 3 | Glutamate-gated kainate-type ion channel receptor subunit GluR5 |
| Nitab4.5_0002222g0070 | 8 | 6 | 1 | 13 | 1 | 4 | 2 | 0 | F-box family protein |
| Nitab4.5_0002796g0030 | 13 | 18 | 10 | 11 | 0 | 9 | 0 | 5 | Unknown Protein |
| Nitab4.5_0000462g0170 | 2 | 2 | 3 | 0 | 1 | 0 | 0 | 0 | Receptor like kinase, RLK |
| Nitab4.5_0004464g0010 | 4 | 1 | 2 | 4 | 1 | 0 | 1 | 1 | Cytochrome P450 |
| Nitab4.5_0002251g0110 | 83 | 40 | 58 | 39 | 25 | 8 | 11 | 14 | NBS-LRR class disease resistance protein |
| Nitab4.5_0000287g0290 | 10 | 4 | 4 | 3 | 1 | 2 | 2 | 1 | Cytochrome P450 |
| Nitab4.5_0001143g0020 | 57 | 71 | 31 | 152 | 23 | 11 | 15 | 32 | PBSP domain-containing protein |
| Nitab4.5_0001157g0010 | 12 | 4 | 6 | 2 | 2 | 2 | 0 | 3 | Ribosomal-protein-alanine N-acetyltransferase |
| Nitab4.5_0001160g0190 | 5 | 5 | 3 | 5 | 0 | 1 | 2 | 1 | Calcium-binding protein 39 |
| Nitab4.5_0001386g0050 | 6 | 11 | 9 | 29 | 2 | 6 | 0 | 6 | Wound induced protein |
| Nitab4.5_0001423g0140 | 4 | 1 | 2 | 2 | 0 | 2 | 1 | 1 | Phosphoglycerate mutase family protein |
| Nitab4.5_0003063g0040 | 1 | 3 | 5 | 7 | 1 | 1 | 2 | 0 | Dof zinc finger protein 6 |
| Nitab4.5_0000235g0130 | 59 | 40 | 19 | 13 | 17 | 5 | 3 | 10 | 3-hydroxy-3-methylglutaryl coenzyme A reductase |
| Nitab4.5_0004955g0020 | 3 | 1 | 2 | 9 | 1 | 0 | 2 | 2 | Homeobox leucine zipper protein |
| Nitab4.5_0000231g0240 | 5 | 2 | 2 | 18 | 3 | 3 | 1 | 0 | Ninja-family protein 1 |
| Nitab4.5_0001816g0090 | 4 | 4 | 2 | 13 | 1 | 1 | 2 | 3 | AT-hook DNA-binding protein (Fragment) |
| Nitab4.5_0002405g0110 | 1 | 3 | 2 | 1 | 0 | 1 | 1 | 1 | Tubulin beta chain |
| Nitab4.5_0000008g0370 | 5 | 2 | 2 | 15 | 2 | 0 | 1 | 3 | PAR-1c protein |
| Nitab4.5_0003912g0030 | 3 | 1 | 3 | 4 | 2 | 1 | 0 | 1 | Dihydroflavonol 4-reductase family-binding domain |
| Nitab4.5_0001087g0030 | 72 | 54 | 46 | 34 | 23 | 9 | 8 | 16 | Unknown Protein |
| Nitab4.5_0002817g0050 | 0 | 5 | 2 | 4 | 1 | 0 | 1 | 1 | Heat shock-like protein |
| Nitab4.5_0000057g0070 | 10 | 9 | 9 | 4 | 3 | 1 | 4 | 1 | Receptor-like kinase |
| Nitab4.5_0001952g0050 | 4 | 7 | 4 | 2 | 1 | 1 | 2 | 0 | NHL repeat-containing protein-like |
| Nitab4.5_0002331g0010 | 3 | 3 | 11 | 0 | 1 | 2 | 2 | 0 | Peroxidase 5 |
| Nitab4.5_0000130g0140 | 126 | 71 | 48 | 71 | 64 | 6 | 13 | 7 | 1-aminocyclopropane-1-carboxylate oxidase |
| Nitab4.5_0000970g0150 | 10 | 2 | 3 | 5 | 3 | 0 | 2 | 0 | Galactokinase like protein |
| Nitab4.5_0002356g0140 | 4 | 3 | 3 | 1 | 1 | 1 | 1 | 0 | Receptor like kinase, RLK |
| Nitab4.5_0006034g0010 | 6 | 7 | 10 | 3 | 4 | 2 | 0 | 1 | Cc-nbs-lrr, resistance protein |
| Nitab4.5_0002337g0050 | 4 | 6 | 5 | 3 | 3 | 1 | 0 | 1 | Oxidoreductase 2OG-Fe oxygenase family protein |
| Nitab4.5_0000188g0090 | 39 | 72 | 46 | 85 | 17 | 11 | 17 | 25 | Chaperone protein dnaJ 11 |
| Nitab4.5_0001863g0230 | 7 | 8 | 5 | 7 | 2 | 1 | 2 | 2 | Ring zinc finger protein (Fragment) |
| Nitab4.5_0000391g0120 | 8 | 6 | 3 | 4 | 2 | 2 | 2 | 1 | Unknown Protein |
| Nitab4.5_0001777g0020 | 72 | 118 | 132 | 21 | 58 | 19 | 12 | 11 | Os06g0524700 protein (Fragment) |
| Nitab4.5_0000864g0100 | 4 | 3 | 3 | 5 | 0 | 2 | 1 | 1 | Cold-shock DNA binding protein |
| Nitab4.5_0001461g0050 | 65 | 34 | 17 | 32 | 16 | 7 | 12 | 9 | Alpha-humulene_(-)-(E)-beta-caryophyllene synthase |
| Nitab4.5_0000118g0080 | 27 | 9 | 10 | 26 | 4 | 5 | 5 | 7 | Cytochrome P450 |
| Nitab4.5_0000795g0070 | 41 | 23 | 17 | 24 | 9 | 7 | 9 | 7 | Solute carrier family 2, facilitated glucose transporter member 3 |
| Nitab4.5_0001231g0050 | 35 | 42 | 43 | 88 | 17 | 12 | 11 | 20 | Ethylene responsive transcription factor 2b |
| Nitab4.5_0000017g0160 | 14 | 12 | 5 | 6 | 0 | 1 | 5 | 5 | WRKY transcription factor 2 |
| Nitab4.5_0000262g0170 | 2 | 1 | 3 | 4 | 0 | 1 | 1 | 1 | Glycerol-3-phosphate transporter |
| Nitab4.5_0001270g0170 | 52 | 44 | 38 | 97 | 18 | 17 | 16 | 18 | Atcambp25-binding protein OF |
| Nitab4.5_0000212g0200 | 2 | 2 | 2 | 2 | 1 | 1 | 0 | 0 | Unknown Protein |
| Nitab4.5_0000655g0030 | 15 | 11 | 13 | 6 | 3 | 3 | 5 | 2 | NaCl-inducible Ca2+-binding protein |
| Nitab4.5_0001223g0090 | 10 | 5 | 5 | 4 | 3 | 1 | 1 | 2 | Serine_threonine-protein kinase receptor |
| Nitab4.5_0000013g0280 | 72 | 63 | 43 | 54 | 13 | 13 | 18 | 27 | Kinase family protein |
| Nitab4.5_0000170g0410 | 10 | 4 | 4 | 3 | 3 | 1 | 0 | 2 | Low affinity zinc transporter |
| Nitab4.5_0003558g0080 | 3 | 6 | 13 | 9 | 2 | 2 | 1 | 4 | Zinc finger (Ran-binding) family protein |
| Nitab4.5_0000230g0020 | 5 | 7 | 6 | 6 | 3 | 2 | 1 | 1 | Receptor like protein kinase |
| Nitab4.5_0000188g0200 | 20 | 17 | 5 | 12 | 5 | 3 | 5 | 3 | Cytochrome P450 |
| Nitab4.5_0000635g0040 | 109 | 66 | 50 | 59 | 29 | 14 | 18 | 26 | Epoxide hydrolase 3 |
| Nitab4.5_0002131g0040 | 3 | 4 | 2 | 1 | 0 | 1 | 1 | 0 | Calcium dependent protein kinase 2 |
| Nitab4.5_0000944g0090 | 7 | 5 | 2 | 5 | 1 | 1 | 3 | 1 | Glutamate decarboxylase |
| Nitab4.5_0000403g0140 | 4 | 2 | 2 | 2 | 0 | 0 | 1 | 2 | Alcohol dehydrogenase zinc-containing |
| Nitab4.5_0001492g0080 | 16 | 10 | 10 | 4 | 4 | 3 | 4 | 1 | Receptor like kinase, RLK |
| Nitab4.5_0000563g0210 | 45 | 27 | 23 | 23 | 20 | 5 | 4 | 7 | Unknown Protein |
| Nitab4.5_0002776g0050 | 26 | 19 | 19 | 16 | 11 | 8 | 1 | 5 | Glutathione peroxidase |
| Nitab4.5_0000342g0270 | 9 | 10 | 6 | 15 | 4 | 3 | 2 | 4 | Ethylene receptor |
| Nitab4.5_0000008g0460 | 2 | 2 | 4 | 2 | 0 | 2 | 0 | 1 | Os03g0169000 protein (Fragment) |
| Nitab4.5_0001030g0120 | 4 | 4 | 9 | 2 | 3 | 1 | 2 | 0 | Microtubule-associated protein TORTIFOLIA1 |
| Nitab4.5_0000006g0300 | 40 | 35 | 23 | 17 | 10 | 6 | 11 | 9 | WRKY transcription factor |
| Nitab4.5_0000381g0130 | 44 | 47 | 116 | 12 | 27 | 20 | 20 | 3 | WRKY transcription factor 6 |
| Nitab4.5_0000742g0110 | 9 | 8 | 9 | 26 | 5 | 5 | 0 | 7 | Chaperone protein dnaJ |
| Nitab4.5_0000059g0360 | 5 | 1 | 4 | 5 | 0 | 2 | 0 | 2 | Glucose transporter 8 |
| Nitab4.5_0001014g0030 | 4 | 2 | 1 | 4 | 1 | 0 | 1 | 1 | Cytochrome P450 |
| Nitab4.5_0002265g0140 | 8 | 12 | 14 | 6 | 3 | 3 | 7 | 1 | cDNA clone J023121M11 full insert sequence |
| Nitab4.5_0002241g0040 | 8 | 7 | 4 | 6 | 2 | 2 | 4 | 0 | Universal stress protein |
| Nitab4.5_0000117g0060 | 2 | 12 | 4 | 9 | 3 | 2 | 2 | 2 | Unknown Protein |
| Nitab4.5_0000057g0260 | 19 | 21 | 20 | 23 | 8 | 7 | 3 | 8 | Transmembrane BAX inhibitor motif-containing protein 4 |
| Nitab4.5_0000443g0080 | 2 | 2 | 1 | 2 | 0 | 1 | 0 | 0 | LRR receptor-like serine_threonine-protein kinase, RLP |
| Nitab4.5_0002022g0010 | 2 | 2 | 1 | 1 | 1 | 0 | 1 | 0 | Ternary complex factor MIP1 |
| Nitab4.5_0001711g0050 | 7 | 7 | 9 | 80 | 8 | 9 | 9 | 8 | Non-specific lipid-transfer protein |
| Nitab4.5_0003766g0010 | 28 | 63 | 48 | 72 | 19 | 21 | 21 | 10 | Plant-specific domain TIGR01615 family protein |
| Nitab4.5_0001691g0140 | 9 | 9 | 7 | 9 | 6 | 3 | 1 | 1 | Cation diffusion facilitator family transporter |
| Nitab4.5_0001575g0050 | 17 | 8 | 6 | 9 | 7 | 1 | 4 | 1 | U-box domain-containing protein 5 |
| Nitab4.5_0003924g0080 | 7 | 7 | 6 | 10 | 3 | 3 | 3 | 2 | AT5g06970_MOJ9_14 |
| Nitab4.5_0001494g0140 | 7 | 7 | 5 | 7 | 1 | 0 | 4 | 5 | Chaperone protein dnaJ |
| Nitab4.5_0002765g0040 | 5 | 2 | 1 | 1 | 1 | 1 | 0 | 0 | Histone-lysine N-methyltransferase MEDEA |
| Nitab4.5_0002116g0040 | 4 | 3 | 1 | 2 | 0 | 1 | 0 | 2 | Chaperone protein dnaJ |
| Nitab4.5_0001536g0020 | 16 | 8 | 10 | 9 | 8 | 2 | 3 | 2 | Receptor-like protein kinase At3g21340 |
| Nitab4.5_0002682g0060 | 11 | 9 | 6 | 47 | 5 | 4 | 3 | 13 | Alpha-galactosidase |
| Nitab4.5_0002219g0040 | 91 | 23 | 36 | 29 | 15 | 15 | 14 | 16 | LRR receptor-like serine_threonine-protein kinase, RLP |
| Nitab4.5_0001654g0010 | 36 | 43 | 27 | 32 | 16 | 9 | 11 | 11 | Hydroxycinnamoyl transferase |
| Nitab4.5_0007184g0020 | 7 | 7 | 8 | 8 | 2 | 2 | 4 | 2 | Pathogen-induced calmodulin-binding protein (Fragment) |
| Nitab4.5_0004625g0020 | 7 | 4 | 2 | 8 | 1 | 3 | 3 | 1 | CONSTANS-like zinc finger protein |
| Nitab4.5_0005043g0060 | 12 | 6 | 16 | 3 | 3 | 4 | 3 | 2 | Glutaredoxin |
| Nitab4.5_0000430g0110 | 6 | 11 | 9 | 13 | 8 | 1 | 1 | 3 | CHP-rich zinc finger protein-like |
| Nitab4.5_0000209g0240 | 2 | 2 | 2 | 7 | 1 | 1 | 0 | 2 | Genomic DNA chromosome 3 P1 clone MUJ8 |
| Nitab4.5_0001495g0020 | 5 | 8 | 3 | 3 | 1 | 2 | 1 | 3 | Unknown Protein |
| Nitab4.5_0000357g0200 | 11 | 7 | 5 | 3 | 5 | 1 | 0 | 2 | RING finger protein 38 |
| Nitab4.5_0001599g0220 | 3 | 2 | 2 | 2 | 1 | 1 | 1 | 1 | Baculoviral IAP repeat-containing 4 (Predicted) |
| Nitab4.5_0000462g0080 | 6 | 9 | 8 | 6 | 3 | 3 | 3 | 1 | Receptor like kinase, RLK |
| Nitab4.5_0000615g0040 | 13 | 10 | 7 | 16 | 8 | 2 | 3 | 3 | Short-chain dehydrogenase_reductase family protein |
| Nitab4.5_0000705g0190 | 4 | 1 | 2 | 1 | 1 | 1 | 1 | 1 | GRAS family transcription factor |
| Nitab4.5_0004507g0010 | 8 | 3 | 3 | 1 | 1 | 1 | 2 | 1 | Receptor-like kinase |
| Nitab4.5_0001860g0020 | 24 | 18 | 22 | 17 | 11 | 5 | 8 | 5 | Glycosyl transferase family 17 protein |
| Nitab4.5_0002088g0040 | 22 | 20 | 16 | 11 | 6 | 7 | 8 | 3 | Genomic DNA chromosome 5 P1 clone MRH10 |
| Nitab4.5_0004048g0040 | 15 | 7 | 15 | 11 | 4 | 5 | 4 | 4 | Alcohol dehydrogenase (Fragment) |
| Nitab4.5_0001553g0040 | 9 | 10 | 10 | 1 | 4 | 3 | 2 | 2 | Nbs-lrr, resistance protein |
| Nitab4.5_0004813g0010 | 23 | 15 | 12 | 5 | 4 | 8 | 2 | 6 | VQ motif family protein |
| Nitab4.5_0001714g0100 | 45 | 31 | 26 | 28 | 19 | 11 | 9 | 7 | Phospholipase D |
| Nitab4.5_0002613g0020 | 4 | 2 | 2 | 3 | 1 | 0 | 2 | 0 | Tyrosyl-DNA phosphodiesterase 1 |
| Nitab4.5_0000622g0110 | 5 | 4 | 3 | 1 | 1 | 1 | 1 | 1 |  |
| Nitab4.5_0002345g0020 | 16 | 7 | 8 | 9 | 5 | 3 | 3 | 3 | RLK, Receptor like protein, putative resistance protein with an antifungal domain |
| Nitab4.5_0000250g0100 | 3 | 1 | 4 | 6 | 2 | 1 | 1 | 1 | Zinc finger FYVE domain containing 26 |
| Nitab4.5_0000652g0050 | 68 | 65 | 47 | 42 | 22 | 11 | 26 | 22 | Glutathione S-transferase-like protein |
| Nitab4.5_0001049g0140 | 16 | 24 | 52 | 14 | 6 | 13 | 9 | 10 | Pistil extensin like protein (Fragment) |
| Nitab4.5_0000705g0020 | 14 | 8 | 11 | 12 | 5 | 3 | 4 | 4 | Receptor like protein kinase |
| Nitab4.5_0002780g0130 | 46 | 36 | 40 | 26 | 22 | 9 | 12 | 11 | U-box domain-containing protein |
| Nitab4.5_0000962g0010 | 9 | 6 | 4 | 6 | 2 | 1 | 4 | 3 | UDP-glucuronosyltransferase 1-1 |
| Nitab4.5_0001874g0010 | 8 | 6 | 3 | 4 | 3 | 1 | 2 | 2 | NPR1-like protein (Fragment) |
| Nitab4.5_0005528g0080 | 13 | 11 | 9 | 6 | 3 | 5 | 2 | 5 | Receptor-like kinase |
| Nitab4.5_0002232g0030 | 106 | 84 | 51 | 203 | 44 | 26 | 47 | 50 | Unknown Protein |
| Nitab4.5_0003885g0100 | 3 | 2 | 2 | 2 | 0 | 0 | 2 | 1 | Kelch-like protein |
| Nitab4.5_0000175g0060 | 5 | 3 | 4 | 1 | 2 | 0 | 1 | 2 | NB-ARC domain containing protein expressed |
| Nitab4.5_0000250g0040 | 4 | 5 | 5 | 2 | 1 | 2 | 2 | 1 | Os10g0422600 protein (Fragment) |
| Nitab4.5_0000882g0090 | 14 | 26 | 15 | 23 | 14 | 3 | 6 | 7 | Protein transport protein Sec61 beta subunit |
| Nitab4.5_0001926g0080 | 15 | 10 | 12 | 34 | 5 | 4 | 8 | 9 | Exoribonuclease R |
| Nitab4.5_0001553g0030 | 4 | 7 | 3 | 1 | 2 | 3 | 1 | 1 | Cc-nbs-lrr, resistance protein |
| Nitab4.5_0000575g0130 | 85 | 64 | 85 | 356 | 54 | 44 | 48 | 80 | Aquaporin 2 |
| Nitab4.5_0000134g0180 | 6 | 26 | 8 | 16 | 5 | 4 | 8 | 5 | F-box protein PP2-B1 |
| Nitab4.5_0001326g0060 | 4 | 5 | 4 | 6 | 2 | 1 | 3 | 1 | Pyrimidine 5_apos-nucleotidase |
| Nitab4.5_0000465g0060 | 17 | 10 | 13 | 7 | 8 | 3 | 7 | 1 | Armadillo_beta-catenin repeat family protein |
| Nitab4.5_0000294g0190 | 8 | 10 | 6 | 6 | 4 | 3 | 2 | 3 | COP9 signalosome subunit 6 |
| Nitab4.5_0005325g0010 | 6 | 9 | 9 | 7 | 0 | 6 | 3 | 2 | Unknown Protein |
| Nitab4.5_0004529g0010 | 292 | 211 | 140 | 155 | 96 | 54 | 75 | 85 | 3-hydroxy-3-methylglutaryl coenzyme A reductase |
| Nitab4.5_0000082g0090 | 2 | 4 | 3 | 3 | 2 | 1 | 0 | 1 | Receptor like kinase, RLK |
| Nitab4.5_0000005g0240 | 4 | 3 | 5 | 5 | 2 | 1 | 1 | 2 | U3 small nucleolar RNA-associated protein 6 homolog |
| Nitab4.5_0001030g0010 | 1 | 2 | 3 | 2 | 0 | 1 | 1 | 1 | Tir-nbs-lrr, resistance protein |
| Nitab4.5_0000293g0110 | 3 | 5 | 2 | 2 | 2 | 1 | 1 | 0 | MORN repeat-containing protein |
| Nitab4.5_0000429g0030 | 60 | 62 | 36 | 222 | 43 | 30 | 32 | 45 | ABC transporter G family member 22 |
| Nitab4.5_0003290g0010 | 8 | 5 | 3 | 2 | 2 | 3 | 0 | 2 | Calcium-dependent protein kinase |
| Nitab4.5_0000737g0060 | 25 | 20 | 10 | 45 | 13 | 9 | 8 | 10 | Hydroxycinnamoyl transferase |
| Nitab4.5_0000987g0110 | 6 | 4 | 8 | 6 | 4 | 1 | 3 | 2 | Mitochondrial glycoprotein family protein |
| Nitab4.5_0000044g0240 | 48 | 49 | 57 | 33 | 32 | 18 | 13 | 12 | Inorganic phosphate transporter |
| Nitab4.5_0000336g0020 | 79 | 47 | 37 | 62 | 22 | 23 | 30 | 16 | Universal stress protein |
| Nitab4.5_0000040g0440 | 4 | 4 | 12 | 3 | 1 | 2 | 3 | 3 | Lipase |
| Nitab4.5_0000775g0070 | 39 | 30 | 38 | 46 | 11 | 20 | 13 | 18 | ATP dependent RNA helicase |
| Nitab4.5_0000198g0120 | 29 | 31 | 31 | 31 | 8 | 11 | 15 | 15 | Flavin-binding kelch domain F box protein |
| Nitab4.5_0001357g0030 | 19 | 31 | 32 | 37 | 9 | 9 | 11 | 19 | Aluminum-induced protein-like |
| Nitab4.5_0002548g0020 | 3 | 1 | 4 | 1 | 1 | 1 | 2 | 1 | Receptor like kinase, RLK |
| Nitab4.5_0000397g0280 | 18 | 10 | 11 | 15 | 9 | 7 | 3 | 3 | Ras-related protein Rab-21 |
| Nitab4.5_0001474g0070 | 16 | 49 | 101 | 63 | 37 | 9 | 22 | 25 |  |
| Nitab4.5_0001392g0090 | 7 | 6 | 5 | 12 | 1 | 3 | 2 | 6 | Unknown Protein |
| Nitab4.5_0000258g0290 | 15 | 16 | 9 | 14 | 4 | 6 | 7 | 5 | Glycogen synthase kinase |
| Nitab4.5_0000072g0120 | 2 | 7 | 5 | 4 | 3 | 3 | 1 | 1 | Alpha-L-fucosidase 1 |
| Nitab4.5_0000123g0220 | 44 | 33 | 42 | 122 | 26 | 21 | 25 | 27 | Atcambp25-binding protein OF |
| Nitab4.5_0000416g0140 | 49 | 56 | 44 | 91 | 44 | 21 | 18 | 17 | Chitinase A |
| Nitab4.5_0001014g0010 | 6 | 6 | 5 | 10 | 2 | 3 | 2 | 4 | Cytochrome P450 |
| Nitab4.5_0000200g0140 | 13 | 13 | 12 | 12 | 4 | 7 | 5 | 5 | 2-hydroxyacid dehydrongenase (Fragment) |
| Nitab4.5_0001764g0140 | 19 | 36 | 11 | 20 | 7 | 8 | 2 | 19 | Unknown Protein |
| Nitab4.5_0000825g0110 | 70 | 55 | 57 | 38 | 39 | 18 | 22 | 13 | Receptor serine_threonine kinase |
| Nitab4.5_0002219g0030 | 25 | 19 | 15 | 41 | 14 | 8 | 5 | 14 | Unknown Protein |
| Nitab4.5_0004300g0180 | 4 | 9 | 6 | 5 | 5 | 2 | 1 | 2 | NAC domain transcription factor protein |
| Nitab4.5_0002909g0010 | 66 | 48 | 50 | 27 | 34 | 20 | 16 | 10 | Receptor-like protein kinase |
| Nitab4.5_0000489g0040 | 5 | 3 | 3 | 7 | 1 | 2 | 1 | 3 | Mediator of RNA polymerase II transcription subunit 25 |
| Nitab4.5_0000130g0320 | 25 | 23 | 22 | 13 | 13 | 7 | 4 | 10 | WRKY transcription factor |
| Nitab4.5_0002649g0020 | 20 | 19 | 12 | 25 | 11 | 8 | 8 | 6 | Calcium-dependent protein kinase 4 |
| Nitab4.5_0001133g0040 | 40 | 47 | 33 | 33 | 26 | 7 | 8 | 24 | BURP domain-containing protein |
| Nitab4.5_0000486g0060 | 7 | 15 | 10 | 8 | 3 | 1 | 2 | 11 | Golgi SNAP receptor complex member 1 |
| Nitab4.5_0002192g0050 | 12 | 7 | 3 | 4 | 5 | 2 | 1 | 3 | Serine_threonine-protein kinase receptor |
| Nitab4.5_0001616g0120 | 6 | 5 | 3 | 1 | 2 | 2 | 1 | 2 | Os03g0731050 protein (Fragment) |
| Nitab4.5_0002463g0040 | 3 | 9 | 5 | 3 | 3 | 1 | 3 | 2 | Cathepsin B |
| Nitab4.5_0000280g0250 | 13 | 11 | 12 | 9 | 5 | 6 | 4 | 3 | Cc-nbs-lrr, resistance protein |
| Nitab4.5_0000794g0140 | 29 | 30 | 37 | 28 | 15 | 14 | 11 | 14 | Calmodulin binding protein |
| Nitab4.5_0000064g0010 | 15 | 9 | 8 | 9 | 6 | 5 | 4 | 1 | DMI1 protein (Fragment)-binding domain |
| Nitab4.5_0002238g0030 | 31 | 22 | 16 | 27 | 10 | 12 | 8 | 11 | class I heat shock protein 1 |
| Nitab4.5_0001135g0060 | 88 | 38 | 52 | 37 | 29 | 23 | 20 | 20 | CCR4-NOT transcription complex subunit 7 |
| Nitab4.5_0005003g0010 | 20 | 12 | 24 | 25 | 3 | 15 | 8 | 9 | Mitochondrial import inner membrane translocase subunit tim23 |
| Nitab4.5_0000588g0160 | 20 | 36 | 26 | 55 | 11 | 21 | 7 | 18 | Os03g0133300 protein (Fragment) |
| Nitab4.5_0003914g0100 | 9 | 6 | 7 | 12 | 6 | 4 | 2 | 3 | AT4G35080-like protein (Fragment) |
| Nitab4.5_0002907g0030 | 2 | 1 | 1 | 2 | 1 | 0 | 1 | 0 | Serine_threonine kinase receptor |
| Nitab4.5_0003965g0070 | 5 | 7 | 6 | 8 | 1 | 2 | 3 | 5 | Nitrilase 1 like protein |
| Nitab4.5_0000208g0400 | 15 | 8 | 10 | 8 | 7 | 4 | 3 | 4 | Receptor-like protein kinase At3g21340 |
| Nitab4.5_0003304g0010 | 8 | 9 | 7 | 10 | 6 | 2 | 3 | 4 | DEP domain-containing protein 1B |
| Nitab4.5_0004554g0020 | 18 | 12 | 10 | 35 | 10 | 5 | 9 | 9 | Exoribonuclease R |
| Nitab4.5_0003180g0060 | 23 | 18 | 16 | 17 | 6 | 3 | 10 | 13 | BHLH transcription factor |
| Nitab4.5_0002765g0010 | 23 | 10 | 13 | 24 | 8 | 4 | 10 | 8 | Caffeoyl-CoA O-methyltransferase |
| Nitab4.5_0000051g0310 | 3 | 6 | 9 | 9 | 3 | 4 | 2 | 3 | Thaumatin-like protein 12104-13574 |
| Nitab4.5_0004152g0010 | 53 | 35 | 13 | 61 | 32 | 11 | 13 | 15 | Unknown Protein |
| Nitab4.5_0003227g0030 | 4 | 6 | 4 | 4 | 1 | 4 | 2 | 2 | Beta-mannosidase |
| Nitab4.5_0002632g0030 | 6 | 19 | 20 | 7 | 9 | 3 | 6 | 4 | Unknown Protein |
| Nitab4.5_0000718g0300 | 4 | 9 | 3 | 9 | 3 | 3 | 2 | 2 | -- |
| Nitab4.5_0000582g0180 | 10 | 19 | 12 | 23 | 7 | 7 | 5 | 8 | Phenylalanine ammonia-lyase |
| Nitab4.5_0000514g0110 | 3 | 4 | 9 | 4 | 2 | 3 | 3 | 1 | Glucose-6P_phosphate translocator |
| Nitab4.5_0002649g0010 | 3 | 2 | 4 | 10 | 2 | 1 | 4 | 2 | Alkaline alpha galactosidase |
| Nitab4.5_0004195g0080 | 58 | 42 | 58 | 28 | 37 | 12 | 16 | 17 | Nodulin-like protein (Fragment) |
| Nitab4.5_0000231g0070 | 5 | 7 | 8 | 4 | 2 | 3 | 2 | 4 | Ribosome maturation factor rimP |
| Nitab4.5_0007385g0010 | 20 | 21 | 11 | 10 | 3 | 10 | 9 | 6 | RING finger protein 38 |
| Nitab4.5_0001315g0010 | 4 | 5 | 1 | 9 | 2 | 2 | 2 | 3 | Peptide transporter 1 |
| Nitab4.5_0000867g0110 | 5 | 4 | 5 | 3 | 2 | 4 | 1 | 1 | -- |
| Nitab4.5_0000368g0350 | 25 | 14 | 12 | 11 | 8 | 7 | 9 | 3 | GRAS family transcription factor |
| Nitab4.5_0007178g0030 | 3 | 6 | 8 | 3 | 3 | 3 | 1 | 1 | Microtubule-associated protein TORTIFOLIA1 |
| Nitab4.5_0000170g0440 | 170 | 133 | 79 | 275 | 65 | 56 | 76 | 95 | Unknown Protein |
| Nitab4.5_0000355g0180 | 3 | 2 | 6 | 8 | 2 | 1 | 3 | 2 | F-box_LRR-repeat protein 3 |
| Nitab4.5_0000551g0260 | 18 | 13 | 17 | 6 | 9 | 5 | 5 | 5 | -- |
| Nitab4.5_0004646g0060 | 3 | 3 | 5 | 2 | 2 | 1 | 0 | 2 | GRAS family transcription factor |
| Nitab4.5_0001200g0090 | 56 | 58 | 34 | 75 | 28 | 18 | 30 | 24 | Hydroxymethylglutaryl-CoA synthase |
| Nitab4.5_0002761g0020 | 7 | 12 | 8 | 5 | 4 | 4 | 2 | 4 | Solute carrier family 22 member 5 (Predicted) |
| Nitab4.5_0001046g0030 | 10 | 3 | 5 | 10 | 3 | 3 | 4 | 3 | Neurogenic locus notch protein-like |
| Nitab4.5_0000473g0120 | 5 | 8 | 7 | 4 | 4 | 1 | 3 | 3 | Mitochondrial carrier family |
| Nitab4.5_0005528g0030 | 7 | 4 | 3 | 3 | 3 | 2 | 0 | 3 | Beta-lactamase domain protein |
| Nitab4.5_0002229g0170 | 14 | 15 | 13 | 13 | 5 | 7 | 7 | 6 | DDRGK domain-containing protein 1 |
| Nitab4.5_0000075g0120 | 3 | 2 | 2 | 1 | 1 | 2 | 0 | 0 | Cc-nbs-lrr, resistance protein |
| Nitab4.5_0001496g0050 | 37 | 32 | 23 | 29 | 24 | 8 | 10 | 13 | Lysine ketoglutarate reductase trans-splicing related 1 |
| Nitab4.5_0001373g0080 | 11 | 11 | 4 | 8 | 2 | 4 | 5 | 5 | RER1 protein |
| Nitab4.5_0000028g0060 | 17 | 24 | 14 | 7 | 11 | 6 | 3 | 8 | Glycosyl transferase family 17 protein |
| Nitab4.5_0003527g0010 | 7 | 9 | 13 | 8 | 6 | 3 | 3 | 5 | Pterin-4-alpha-carbinolamine dehydratase |
| Nitab4.5_0000354g0130 | 19 | 22 | 14 | 47 | 8 | 6 | 15 | 18 | Hydroxycinnamoyl CoA quinate transferase 2 |
| Nitab4.5_0002003g0070 | 60 | 63 | 44 | 67 | 30 | 23 | 20 | 34 | LOB domain protein 38 |
| Nitab4.5_0003034g0050 | 5 | 3 | 4 | 6 | 2 | 2 | 2 | 2 | Os05g0264200 protein (Fragment) |
| Nitab4.5_0000363g0070 | 10 | 5 | 4 | 12 | 8 | 2 | 2 | 3 | Protein tolB |
| Nitab4.5_0001234g0050 | 21 | 16 | 12 | 16 | 11 | 6 | 6 | 6 | CBL-interacting protein kinase 11 |
| Nitab4.5_0000187g0040 | 17 | 17 | 13 | 47 | 11 | 9 | 12 | 12 | Plant-specific domain TIGR01615 family protein |
| Nitab4.5_0000006g0530 | 7 | 7 | 5 | 10 | 4 | 3 | 3 | 3 | CONSTANS-like zinc finger protein |
| Nitab4.5_0000048g0080 | 43 | 71 | 105 | 25 | 36 | 35 | 31 | 11 | WRKY transcription factor 6 |
| Nitab4.5_0000080g0290 | 10 | 9 | 10 | 11 | 1 | 5 | 4 | 9 | Cysteine-type peptidase |
| Nitab4.5_0001600g0120 | 95 | 112 | 79 | 83 | 59 | 34 | 32 | 47 | LRR receptor-like serine_threonine-protein kinase, RLP |
| Nitab4.5_0000696g0030 | 23 | 16 | 14 | 8 | 13 | 4 | 5 | 6 | WRKY transcription factor |
| Nitab4.5_0000080g0010 | 6 | 9 | 8 | 25 | 4 | 7 | 7 | 4 | Dehydration-responsive family protein |
| Nitab4.5_0000308g0310 | 13 | 8 | 6 | 7 | 7 | 4 | 3 | 2 | Genomic DNA chromosome 5 TAC clone K1F13 |
| Nitab4.5_0000651g0250 | 8 | 11 | 8 | 16 | 6 | 3 | 4 | 6 | Os07g0587200 protein (Fragment) |
| Nitab4.5_0000171g0310 | 27 | 21 | 21 | 37 | 15 | 11 | 14 | 10 | Unknown Protein |
| Nitab4.5_0000207g0430 | 6 | 6 | 7 | 5 | 4 | 4 | 2 | 2 | Serine_threonine kinase receptor |
| Nitab4.5_0000021g0020 | 76 | 49 | 43 | 134 | 37 | 28 | 35 | 42 | O-methyltransferase |
| Nitab4.5_0003858g0010 | 320 | 303 | 193 | 253 | 170 | 87 | 102 | 147 | Peroxidase |
| Nitab4.5_0000742g0100 | 47 | 46 | 36 | 65 | 24 | 28 | 20 | 19 | Unknown Protein |
| Nitab4.5_0001320g0070 | 3 | 2 | 3 | 2 | 0 | 1 | 2 | 2 | Myosin |
| Nitab4.5_0000377g0160 | 11 | 5 | 8 | 8 | 4 | 4 | 4 | 3 | Unknown Protein |
| Nitab4.5_0000618g0030 | 15 | 7 | 7 | 7 | 9 | 4 | 5 | 1 | Glycosyl transferase family 8 glycogenin |
| Nitab4.5_0001450g0190 | 29 | 21 | 24 | 31 | 12 | 16 | 11 | 11 | U-box domain-containing protein 14 |
| Nitab4.5_0001373g0040 | 7 | 5 | 8 | 6 | 3 | 4 | 1 | 6 | Receptor like kinase, RLK |
| Nitab4.5_0003247g0060 | 12 | 6 | 9 | 8 | 3 | 5 | 4 | 5 | Zinc finger-homeodomain protein 1 (Fragment) |
| Nitab4.5_0005478g0020 | 8 | 8 | 6 | 7 | 6 | 4 | 1 | 2 | Farnesyl pyrophosphate synthase |
| Nitab4.5_0001244g0100 | 4 | 4 | 3 | 1 | 2 | 1 | 2 | 1 | -- |
| Nitab4.5_0002245g0130 | 26 | 24 | 14 | 18 | 9 | 10 | 8 | 12 | Transmembrane protein 222 (Fragment) |
| Nitab4.5_0001558g0060 | 10 | 6 | 10 | 4 | 1 | 2 | 6 | 5 | Oligopeptide transporter 4 |
| Nitab4.5_0000438g0070 | 76 | 52 | 41 | 45 | 30 | 24 | 27 | 23 | Calcium-dependent protein kinase |
| Nitab4.5_0002080g0050 | 28 | 43 | 21 | 50 | 14 | 15 | 13 | 26 | F-box protein PP2-B1 |
| Nitab4.5_0000306g0170 | 231 | 169 | 124 | 156 | 114 | 83 | 72 | 59 | ATP-binding cassette transporter |
| Nitab4.5_0004506g0060 | 7 | 6 | 7 | 5 | 2 | 4 | 3 | 3 | WRKY transcription factor 6 |
| Nitab4.5_0000092g0120 | 12 | 9 | 7 | 12 | 4 | 6 | 4 | 5 | GRAS family transcription factor |
| Nitab4.5_0001175g0140 | 87 | 85 | 65 | 93 | 58 | 32 | 30 | 40 | Binding protein |
| Nitab4.5_0000041g0380 | 13 | 11 | 10 | 8 | 3 | 7 | 3 | 8 | Serine_threonine-protein phosphatase 2A regulatory subunit delta 1 isoform |
| Nitab4.5_0000622g0170 | 127 | 83 | 92 | 139 | 105 | 42 | 40 | 28 | 1-aminocyclopropane-1-carboxylate oxidase |
| Nitab4.5_0000883g0070 | 3 | 3 | 4 | 2 | 3 | 1 | 1 | 1 | Receptor-like kinase |
| Nitab4.5_0001231g0120 | 5 | 4 | 4 | 8 | 4 | 2 | 2 | 2 | RING finger protein |
| Nitab4.5_0001822g0080 | 9 | 6 | 4 | 5 | 2 | 4 | 2 | 3 | Pentatricopeptide repeat-containing protein-like protein |
| Nitab4.5_0000101g0080 | 24 | 22 | 16 | 13 | 10 | 10 | 8 | 8 | Receptor-like kinase |
| Nitab4.5_0001301g0070 | 126 | 175 | 97 | 233 | 59 | 66 | 81 | 102 | E3 ubiquitin-protein ligase MARCH6 |
| Nitab4.5_0002399g0010 | 19 | 26 | 16 | 26 | 13 | 8 | 12 | 9 | Transmembrane protein 56 |
| Nitab4.5_0004087g0030 | 6 | 7 | 5 | 8 | 4 | 4 | 2 | 3 | Receptor-like kinase |
| Nitab4.5_0000519g0310 | 17 | 11 | 9 | 13 | 8 | 8 | 5 | 3 | Genomic DNA chromosome 3 P1 clone MLD14 |
| Nitab4.5_0000188g0340 | 58 | 57 | 108 | 65 | 44 | 22 | 25 | 51 | Endochitinase (Chitinase) |
| Nitab4.5_0000326g0340 | 5 | 4 | 3 | 8 | 3 | 3 | 2 | 2 | Myosin XI-2 |
| Nitab4.5_0000005g0190 | 6 | 5 | 3 | 3 | 1 | 4 | 2 | 2 | Transcription factor WRKY |
| Nitab4.5_0000052g0220 | 67 | 67 | 56 | 172 | 56 | 37 | 44 | 42 | Aspartate aminotransferase |
| Nitab4.5_0000033g0230 | 19 | 20 | 21 | 30 | 19 | 8 | 9 | 8 | CHP-rich zinc finger protein-like |
| Nitab4.5_0007482g0040 | 23 | 13 | 19 | 17 | 3 | 9 | 12 | 12 | Mediator of RNA polymerase II transcription subunit 21 |
| Nitab4.5_0001288g0100 | 3 | 4 | 7 | 4 | 5 | 1 | 2 | 1 | Receptor like kinase, RLK |
| Nitab4.5_0000676g0060 | 1 | 6 | 11 | 6 | 3 | 2 | 3 | 5 | Serine_threonine-protein phosphatase 6 regulatory ankyrin repeat subunit A |
| Nitab4.5_0000721g0200 | 17 | 21 | 18 | 13 | 14 | 6 | 7 | 7 | Calcium dependent protein kinase 1 |
| Nitab4.5_0000297g0160 | 9 | 7 | 11 | 27 | 5 | 7 | 5 | 9 | Uncharacterized secreted protein |
| Nitab4.5_0003316g0030 | 12 | 23 | 12 | 23 | 11 | 6 | 10 | 8 | WRKY transcription factor 2 |
| Nitab4.5_0002320g0020 | 34 | 46 | 44 | 45 | 32 | 21 | 15 | 16 | Hydroxycinnamoyl CoA shikimate_quinate hydroxycinnamoyltransferase |
| Nitab4.5_0000055g0100 | 11 | 13 | 8 | 35 | 11 | 6 | 8 | 9 | Saccharopine dehydrogenase (NAD(+) L-glutamate-forming) |
| Nitab4.5_0001317g0070 | 186 | 130 | 112 | 202 | 88 | 75 | 73 | 79 | Arginine decarboxylase |
| Nitab4.5_0001713g0020 | 18 | 12 | 22 | 12 | 40 | 39 | 24 | 28 | Os12g0604200 protein (Fragment) |
| Nitab4.5_0001913g0140 | 2 | 4 | 4 | 3 | 9 | 6 | 6 | 3 | Genomic DNA chromosome 3 TAC clone K10D20 |
| Nitab4.5_0003477g0040 | 11 | 13 | 13 | 10 | 34 | 27 | 21 | 12 | Transcription factor |
| Nitab4.5_0001735g0040 | 2 | 3 | 3 | 2 | 2 | 4 | 10 | 5 | Glutamate decarboxylase |
| Nitab4.5_0003746g0030 | 20 | 21 | 24 | 24 | 42 | 62 | 43 | 31 | RAG1-activating protein 1 homolog |
| Nitab4.5_0003855g0010 | 2 | 2 | 2 | 2 | 3 | 4 | 2 | 5 | Serine_threonine protein kinase-like |
| Nitab4.5_0002403g0050 | 3 | 6 | 4 | 14 | 12 | 10 | 18 | 14 | Fasciclin-like arabinogalactan protein 9 |
| Nitab4.5_0000603g0070 | 5 | 3 | 6 | 7 | 15 | 8 | 12 | 7 | Copper transport protein 86 |
| Nitab4.5_0000104g0010 | 10 | 11 | 9 | 5 | 19 | 20 | 20 | 14 | Phototropic-responsive NPH3 family protein |
| Nitab4.5_0004599g0090 | 2 | 11 | 10 | 5 | 9 | 18 | 17 | 12 | BZIP transcription factor family protein |
| Nitab4.5_0001586g0100 | 24 | 21 | 25 | 12 | 46 | 42 | 39 | 36 | Chitinase |
| Nitab4.5_0000037g0170 | 3 | 2 | 5 | 3 | 4 | 4 | 6 | 11 | Unknown Protein |
| Nitab4.5_0001044g0050 | 1 | 3 | 3 | 1 | 4 | 6 | 3 | 5 | Leucine Rich Repeat family protein expressed |
| Nitab4.5_0000895g0210 | 8 | 6 | 8 | 9 | 10 | 16 | 25 | 11 | Pentatricopeptide repeat-containing protein |
| Nitab4.5_0001823g0020 | 13 | 14 | 20 | 11 | 28 | 24 | 31 | 33 | Malate dehydrogenase, cytoplasmic |
| Nitab4.5_0000107g0120 | 25 | 16 | 30 | 17 | 31 | 73 | 55 | 19 | O-methyltransferase |
| Nitab4.5_0001560g0050 | 41 | 30 | 32 | 34 | 91 | 83 | 67 | 36 | Serine_threonine protein kinase |
| Nitab4.5_0000132g0510 | 2 | 2 | 3 | 5 | 4 | 7 | 7 | 8 | Ankyrin repeat domain 1 |
| Nitab4.5_0000203g0030 | 26 | 8 | 10 | 13 | 25 | 36 | 37 | 19 | BURP domain-containing protein (Fragment) |
| Nitab4.5_0000046g0200 | 502 | 564 | 874 | 481 | 1278 | 1545 | 1249 | 865 | Non-specific lipid-transfer protein |
| Nitab4.5_0001120g0010 | 2 | 1 | 3 | 1 | 4 | 5 | 4 | 1 | Multidrug resistance protein ABC transporter family |
| Nitab4.5_0002632g0060 | 2 | 3 | 1 | 1 | 3 | 3 | 4 | 4 | Phosphoglycerate kinase |
| Nitab4.5_0000173g0030 | 6 | 7 | 8 | 16 | 10 | 29 | 20 | 18 | Aquaporin-like protein |
| Nitab4.5_0000363g0310 | 7 | 8 | 11 | 4 | 23 | 15 | 15 | 8 | DNAJ heat shock N-terminal domain-containing protein |
| Nitab4.5_0000004g0060 | 3 | 4 | 3 | 2 | 4 | 4 | 9 | 8 | mRNA binding protein Pumilio 2 |
| Nitab4.5_0000080g0080 | 11 | 7 | 8 | 9 | 21 | 28 | 18 | 5 | Long-chain-fatty-acid--CoA ligase |
| Nitab4.5_0002552g0010 | 19 | 10 | 15 | 14 | 33 | 39 | 21 | 24 | Lipase |
| Nitab4.5_0000831g0030 | 7 | 23 | 16 | 9 | 23 | 35 | 30 | 26 | BZIP transcription factor family protein |
| Nitab4.5_0002085g0130 | 10 | 16 | 19 | 9 | 30 | 24 | 28 | 29 | GDSL esterase_lipase At5g33370 |
| Nitab4.5_0001797g0030 | 3 | 3 | 3 | 2 | 4 | 8 | 5 | 6 | Glycosyltransferase |
| Nitab4.5_0000048g0070 | 5 | 4 | 8 | 7 | 8 | 8 | 17 | 17 | GDSL esterase_lipase At1g28590 |
| Nitab4.5_0000633g0070 | 2 | 3 | 4 | 8 | 8 | 12 | 7 | 9 | AT-hook motif nuclear localized protein 1 |
| Nitab4.5_0002482g0070 | 53 | 53 | 58 | 23 | 78 | 137 | 107 | 66 | Polyphenol oxidase |
| Nitab4.5_0000202g0470 | 30 | 29 | 24 | 15 | 43 | 60 | 50 | 55 | Glutathione S-transferase |
| Nitab4.5_0002393g0070 | 9 | 3 | 8 | 8 | 11 | 11 | 24 | 12 | 50S ribosomal protein L28 |
| Nitab4.5_0004979g0130 | 9 | 6 | 7 | 4 | 15 | 11 | 13 | 14 | FIP1 |
| Nitab4.5_0001082g0080 | 10 | 4 | 6 | 7 | 8 | 20 | 18 | 11 | Neutral invertase like protein |
| Nitab4.5_0005622g0060 | 3 | 1 | 2 | 2 | 4 | 1 | 5 | 4 | Proline-, glutamic acid- and leucine-rich protein 1 |
| Nitab4.5_0000391g0390 | 2 | 1 | 1 | 2 | 4 | 5 | 3 | 2 | Xenotropic and polytropic retrovirus receptor |
| Nitab4.5_0005169g0010 | 4 | 1 | 3 | 2 | 6 | 6 | 8 | 2 | Octicosapeptide_Phox_Bem1p domain-containing protein |
| Nitab4.5_0002165g0040 | 4 | 2 | 6 | 4 | 5 | 11 | 8 | 10 | Replication protein A subunit |
| Nitab4.5_0000083g0140 | 3 | 4 | 4 | 2 | 10 | 4 | 7 | 7 | Pentatricopeptide repeat-containing protein At4g21190 |
| Nitab4.5_0006360g0010 | 7 | 14 | 13 | 18 | 33 | 25 | 32 | 19 | Heat stress transcription factor A3-type, DNA-binding |
| Nitab4.5_0001231g0030 | 2 | 2 | 3 | 1 | 4 | 2 | 4 | 8 | DNA primase |
| Nitab4.5_0000243g0070 | 2 | 4 | 4 | 4 | 7 | 9 | 7 | 7 | Glucosyltransferase-2 |
| Nitab4.5_0003649g0020 | 7 | 14 | 13 | 11 | 38 | 23 | 15 | 19 | Outer membrane lipoprotein blc |
| Nitab4.5_0000976g0170 | 4 | 5 | 14 | 5 | 17 | 16 | 14 | 13 | Sec14-like (Fragment) |
| Nitab4.5_0001034g0170 | 8 | 6 | 3 | 2 | 11 | 13 | 8 | 11 | ATP-dependent RNA helicase fal1 |
| Nitab4.5_0002927g0060 | 2 | 3 | 3 | 2 | 5 | 5 | 4 | 6 | Exosome complex exonuclease RRP4 |
| Nitab4.5_0000178g0330 | 4 | 1 | 3 | 6 | 6 | 8 | 10 | 7 | Mannan endo-1 4-beta-mannosidase |
| Nitab4.5_0001970g0020 | 12 | 12 | 28 | 17 | 20 | 19 | 38 | 68 | Calcium_proton exchanger |
| Nitab4.5_0003559g0050 | 5 | 9 | 12 | 7 | 16 | 21 | 19 | 14 | Unknown Protein |
| Nitab4.5_0002265g0180 | 2 | 5 | 4 | 3 | 8 | 7 | 11 | 5 | Beta-1,3-galactosyl-O-glycosyl-glycoprotein beta-1,6-N-acetylglucosaminyltransferase 4 |
| Nitab4.5_0003077g0030 | 4 | 5 | 2 | 3 | 5 | 5 | 13 | 10 | Unknown Protein |
| Nitab4.5_0000516g0020 | 1 | 2 | 2 | 1 | 4 | 2 | 4 | 3 | Unknown Protein |
| Nitab4.5_0000614g0050 | 21 | 8 | 5 | 13 | 24 | 41 | 25 | 13 | Unknown Protein |
| Nitab4.5_0003978g0050 | 1 | 6 | 6 | 4 | 7 | 7 | 15 | 7 | Ganglioside-induced differentiation-associated protein 1 |
| Nitab4.5_0001728g0060 | 4 | 1 | 2 | 1 | 5 | 2 | 5 | 4 | Ribonucleoside-diphosphate reductase |
| Nitab4.5_0001989g0070 | 4 | 13 | 7 | 5 | 9 | 15 | 12 | 28 | 3-oxo-5-alpha-steroid 4-dehydrogenase family protein |
| Nitab4.5_0000094g0020 | 42 | 26 | 31 | 20 | 75 | 71 | 66 | 50 | Hydrolase alpha_beta fold family protein expressed |
| Nitab4.5_0000855g0100 | 2 | 1 | 2 | 2 | 7 | 2 | 3 | 4 | MORC family CW-type zinc finger 3 |
| Nitab4.5_0003062g0020 | 2 | 1 | 1 | 3 | 6 | 3 | 4 | 3 | Cc-nbs-lrr, resistance protein with an R1 specific domain |
| Nitab4.5_0002616g0040 | 3 | 4 | 3 | 2 | 5 | 7 | 8 | 7 | Unknown Protein |
| Nitab4.5_0001268g0020 | 9 | 13 | 10 | 20 | 6 | 22 | 27 | 59 | Calcium-dependent protein kinase 2 |
| Nitab4.5_0001256g0060 | 5 | 1 | 4 | 2 | 9 | 8 | 6 | 3 | Malonyl CoA anthocyanin 3-O-glucoside-6_apos_apos-O-malonyltransferase |
| Nitab4.5_0004813g0050 | 9 | 9 | 7 | 12 | 19 | 30 | 22 | 11 | WD-40 repeat family protein |
| Nitab4.5_0001494g0050 | 13 | 13 | 15 | 6 | 22 | 38 | 24 | 18 | Transcription factor |
| Nitab4.5_0002252g0080 | 2 | 1 | 1 | 2 | 3 | 3 | 3 | 5 | DnaJ homolog subfamily C member 7 |
| Nitab4.5_0000129g0190 | 1 | 2 | 2 | 0 | 2 | 2 | 3 | 3 | WD-repeat protein |
| Nitab4.5_0001163g0010 | 8 | 7 | 12 | 9 | 12 | 19 | 17 | 32 | Self-pruning interacting protein 1 |
| Nitab4.5_0000154g0280 | 3 | 5 | 5 | 4 | 4 | 13 | 14 | 7 | Hydrolase alpha_beta fold family protein |
| Nitab4.5_0002762g0010 | 11 | 18 | 7 | 15 | 23 | 24 | 30 | 36 | Beta-xylosidase 1 |
| Nitab4.5_0000187g0160 | 30 | 30 | 13 | 15 | 68 | 49 | 37 | 39 | Auxin responsive protein |
| Nitab4.5_0001892g0050 | 7 | 5 | 4 | 4 | 13 | 17 | 9 | 4 | Multi-sensor hybrid histidine kinase |
| Nitab4.5_0000287g0320 | 7 | 9 | 4 | 7 | 15 | 12 | 18 | 15 | Peroxisomal multifunctional enzyme type 2 |
| Nitab4.5_0000928g0090 | 20 | 20 | 8 | 20 | 33 | 39 | 44 | 35 | Amino acid transporter |
| Nitab4.5_0001030g0140 | 6 | 11 | 8 | 6 | 17 | 22 | 15 | 14 | Glycosyltransferase family GT8 protein |
| Nitab4.5_0004651g0010 | 33 | 25 | 23 | 28 | 74 | 56 | 72 | 42 | Unknown Protein |
| Nitab4.5_0000307g0240 | 17 | 31 | 47 | 13 | 65 | 78 | 69 | 34 | BHLH transcription factor |
| Nitab4.5_0003381g0060 | 2 | 4 | 5 | 2 | 7 | 10 | 4 | 8 | Iaa-amino acid hydrolase 9 |
| Nitab4.5_0000567g0070 | 2 | 4 | 2 | 2 | 4 | 2 | 8 | 7 | Serine carboxypeptidase |
| Nitab4.5_0000396g0260 | 2 | 2 | 1 | 1 | 3 | 3 | 3 | 4 | Glycogen synthase |
| Nitab4.5_0002421g0040 | 7 | 3 | 7 | 7 | 13 | 8 | 16 | 14 | Pentatricopeptide repeat-containing protein |
| Nitab4.5_0005376g0030 | 1 | 2 | 3 | 3 | 4 | 6 | 6 | 3 | Pentatricopeptide repeat-containing protein |
| Nitab4.5_0001934g0020 | 2 | 5 | 4 | 2 | 14 | 3 | 3 | 7 | Cytochrome P450 |
| Nitab4.5_0000218g0060 | 3 | 4 | 3 | 1 | 5 | 12 | 6 | 1 | Acyltransferase-like protein |
| Nitab4.5_0007418g0010 | 3 | 4 | 12 | 7 | 15 | 16 | 8 | 22 | -- |
| Nitab4.5_0004787g0010 | 65 | 52 | 45 | 34 | 91 | 155 | 136 | 65 | Unknown Protein |
| Nitab4.5_0000635g0100 | 156 | 153 | 215 | 66 | 307 | 486 | 352 | 202 | Auxin-responsive GH3-like |
| Nitab4.5_0000603g0060 | 9 | 4 | 4 | 14 | 13 | 24 | 15 | 19 | Os12g0236050 protein (Fragment) |
| Nitab4.5_0001356g0110 | 6 | 14 | 5 | 9 | 17 | 31 | 13 | 17 | GTP binding protein |
| Nitab4.5_0002577g0050 | 3 | 3 | 4 | 2 | 6 | 4 | 9 | 6 | Ribonucleoside-diphosphate reductase |
| Nitab4.5_0000085g0320 | 4 | 3 | 3 | 2 | 6 | 6 | 6 | 10 | Homocysteine s-methyltransferase |
| Nitab4.5_0000353g0010 | 1 | 1 | 2 | 5 | 5 | 3 | 12 | 4 | Heat stress transcription factor A3-type, DNA-binding |
| Nitab4.5_0000194g0290 | 3 | 7 | 5 | 5 | 18 | 15 | 11 | 4 | Unknown Protein |
| Nitab4.5_0002070g0010 | 150 | 145 | 105 | 68 | 345 | 327 | 284 | 125 | -- |
| Nitab4.5_0000785g0140 | 2 | 5 | 1 | 1 | 3 | 6 | 7 | 6 | Genomic DNA chromosome 5 TAC clone K19B1 |
| Nitab4.5_0000410g0370 | 15 | 5 | 7 | 7 | 17 | 28 | 23 | 12 | Hydrolase alpha_beta fold family protein |
| Nitab4.5_0001701g0170 | 36 | 39 | 37 | 40 | 61 | 86 | 99 | 108 | Fasciclin-like arabinogalactan protein 19 |
| Nitab4.5_0002352g0050 | 2 | 5 | 4 | 2 | 6 | 6 | 11 | 5 | Phytochrome kinase substrate 1 |
| Nitab4.5_0000315g0010 | 2 | 8 | 6 | 4 | 10 | 9 | 15 | 13 | Unknown Protein |
| Nitab4.5_0000081g0120 | 5 | 3 | 3 | 6 | 8 | 6 | 10 | 16 | Germin-like protein |
| Nitab4.5_0000073g0350 | 12 | 4 | 5 | 7 | 22 | 19 | 17 | 7 | class I heat shock protein |
| Nitab4.5_0001556g0100 | 24 | 21 | 12 | 17 | 57 | 43 | 39 | 36 | Auxin responsive protein |
| Nitab4.5_0003138g0030 | 0 | 1 | 1 | 1 | 2 | 1 | 3 | 1 | Receptor like kinase, RLK |
| Nitab4.5_0002649g0040 | 2 | 5 | 4 | 3 | 8 | 7 | 8 | 9 | Histone-lysine N-methyltransferase NSD3 |
| Nitab4.5_0000008g0230 | 21 | 10 | 19 | 29 | 46 | 78 | 42 | 20 | Unknown Protein |
| Nitab4.5_0000246g0010 | 2 | 3 | 1 | 3 | 7 | 6 | 5 | 5 | E3 SUMO-protein ligase NSE2 |
| Nitab4.5_0000956g0070 | 6 | 9 | 10 | 9 | 10 | 17 | 26 | 28 | Phosphatidylinositol-specific phospholipase c |
| Nitab4.5_0007026g0060 | 14 | 4 | 9 | 14 | 40 | 28 | 19 | 9 | Inositol 1 4 5-trisphosphate 5-phosphatase |
| Nitab4.5_0000104g0330 | 2 | 1 | 7 | 4 | 8 | 9 | 7 | 8 | Genomic DNA chromosome 5 P1 clone MQN23 |
| Nitab4.5_0000679g0110 | 1 | 1 | 3 | 1 | 2 | 5 | 4 | 2 | 5_apos-AMP-activated protein kinase subunit beta-2 |
| Nitab4.5_0000102g0020 | 14 | 8 | 1 | 3 | 20 | 20 | 18 | 5 | Os06g0207500 protein (Fragment) |
| Nitab4.5_0003259g0080 | 144 | 170 | 125 | 203 | 283 | 331 | 425 | 510 | ChaC cation transport regulator-like 1 |
| Nitab4.5_0000257g0230 | 1 | 3 | 2 | 1 | 6 | 6 | 2 | 4 | Polymerase (DNA directed) mu |
| Nitab4.5_0004230g0010 | 35 | 25 | 37 | 31 | 121 | 111 | 61 | 20 | Unknown Protein |
| Nitab4.5_0000770g0100 | 1 | 2 | 1 | 2 | 1 | 3 | 5 | 6 | Laccase |
| Nitab4.5_0000006g0100 | 4 | 1 | 5 | 9 | 11 | 15 | 14 | 7 | Glucose-6-phosphate_phosphate translocator 2 |
| Nitab4.5_0003039g0040 | 11 | 1 | 3 | 9 | 17 | 22 | 14 | 5 | Ethylene-responsive transcription factor 13 |
| Nitab4.5_0000709g0070 | 24 | 29 | 26 | 33 | 76 | 94 | 61 | 40 | ACT domain-containing protein |
| Nitab4.5_0000283g0170 | 93 | 62 | 45 | 62 | 280 | 114 | 126 | 125 | Gibberellin-regulated protein 2 |
| Nitab4.5_0000246g0100 | 2 | 7 | 8 | 4 | 8 | 19 | 15 | 9 | BZIP transcription factor family protein |
| Nitab4.5_0000209g0090 | 3 | 3 | 13 | 8 | 10 | 22 | 19 | 18 | Ethylene-responsive transcription factor 11 |
| Nitab4.5_0000401g0150 | 1 | 2 | 3 | 1 | 2 | 3 | 4 | 8 | Methyltransferase |
| Nitab4.5_0004924g0030 | 36 | 12 | 19 | 47 | 76 | 89 | 66 | 50 | Calmodulin-binding protein |
| Nitab4.5_0000123g0610 | 1 | 1 | 1 | 1 | 1 | 2 | 6 | 2 | -- |
| Nitab4.5_0004160g0020 | 1 | 1 | 1 | 2 | 4 | 2 | 3 | 3 | BEL1-like homeodomain protein 3 |
| Nitab4.5_0000682g0080 | 3 | 2 | 3 | 2 | 5 | 11 | 3 | 5 | Peroxidase |
| Nitab4.5_0000091g0510 | 12 | 19 | 13 | 24 | 36 | 62 | 44 | 31 | Xyloglucan endotransglucosylase_hydrolase 1 |
| Nitab4.5_0004425g0020 | 2 | 1 | 2 | 3 | 4 | 3 | 7 | 5 | Cytochrome P450 |
| Nitab4.5_0005206g0010 | 12 | 7 | 7 | 10 | 28 | 37 | 22 | 5 | Cellulose synthase-like C1-2 glycosyltransferase family 2 protein |
| Nitab4.5_0001952g0250 | 11 | 7 | 4 | 7 | 12 | 37 | 17 | 7 | Peroxidase 65 |
| Nitab4.5_0000630g0080 | 17 | 8 | 11 | 6 | 18 | 23 | 40 | 26 | Unknown Protein |
| Nitab4.5_0001492g0030 | 4 | 1 | 2 | 3 | 5 | 5 | 7 | 7 | Caffeoyl-CoA O-methyltransferase |
| Nitab4.5_0001657g0020 | 2 | 1 | 2 | 2 | 4 | 4 | 3 | 6 | Receptor like kinase, RLK |
| Nitab4.5_0000647g0120 | 3 | 5 | 2 | 2 | 7 | 11 | 6 | 7 | Receptor like protein kinase |
| Nitab4.5_0000978g0100 | 4 | 3 | 4 | 2 | 4 | 12 | 8 | 7 | Chitinase-like protein |
| Nitab4.5_0002700g0020 | 2 | 3 | 2 | 10 | 18 | 7 | 11 | 8 | Unknown Protein |
| Nitab4.5_0000038g0160 | 17 | 10 | 8 | 7 | 32 | 38 | 27 | 12 | Bile acid sodium symporter family protein |
| Nitab4.5_0000565g0270 | 2 | 1 | 1 | 0 | 2 | 3 | 3 | 2 | UDP-glucuronosyltransferase |
| Nitab4.5_0002415g0020 | 17 | 13 | 22 | 55 | 92 | 88 | 67 | 27 | Unknown Protein |
| Nitab4.5_0001612g0090 | 1 | 1 | 1 | 1 | 1 | 5 | 2 | 4 | Zinc ion binding protein |
| Nitab4.5_0000764g0020 | 4 | 1 | 1 | 3 | 6 | 9 | 6 | 4 | Inositol 1 4 5-trisphosphate 5-phosphatase |
| Nitab4.5_0003328g0020 | 47 | 38 | 32 | 59 | 103 | 135 | 129 | 91 | Fasciclin-like arabinogalactan protein 10 |
| Nitab4.5_0004557g0020 | 8 | 10 | 7 | 18 | 31 | 38 | 32 | 13 | Tyrosine-protein kinase transforming protein Src |
| Nitab4.5_0000134g0030 | 19 | 21 | 29 | 16 | 93 | 50 | 30 | 50 | photosystem II polypeptide |
| Nitab4.5_0000256g0410 | 2 | 2 | 1 | 2 | 6 | 4 | 5 | 3 | AT-hook motif nuclear localized protein 13 |
| Nitab4.5_0000040g0410 | 2 | 4 | 2 | 10 | 17 | 11 | 6 | 13 | Unknown Protein |
| Nitab4.5_0000047g0180 | 2 | 1 | 1 | 1 | 3 | 2 | 5 | 3 | Phosphatidylinositol transfer protein SFH5 |
| Nitab4.5_0002315g0170 | 2 | 9 | 8 | 5 | 12 | 23 | 11 | 17 | DNA-3-methyladenine glycosylase I |
| Nitab4.5_0000568g0030 | 7 | 2 | 13 | 8 | 8 | 32 | 21 | 17 | Expansin |
| Nitab4.5_0002654g0010 | 3 | 4 | 3 | 2 | 8 | 7 | 12 | 6 | SNARE associated Golgi protein |
| Nitab4.5_0000124g0040 | 1 | 2 | 4 | 3 | 8 | 7 | 7 | 5 | Gibberellin 20-oxidase-1 |
| Nitab4.5_0000170g0250 | 5 | 3 | 3 | 6 | 10 | 22 | 12 | 4 | LRR receptor-like serine_threonine-protein kinase, RLP |
| Nitab4.5_0000578g0100 | 2 | 3 | 3 | 2 | 7 | 4 | 10 | 4 | Pentatricopeptide repeat-containing protein |
| Nitab4.5_0000036g0030 | 2 | 2 | 2 | 0 | 4 | 8 | 3 | 3 | Speckle-type POZ protein |
| Nitab4.5_0000630g0170 | 6 | 3 | 2 | 10 | 12 | 18 | 16 | 11 | Unknown Protein |
| Nitab4.5_0001196g0050 | 3 | 7 | 7 | 9 | 15 | 9 | 19 | 26 | -- |
| Nitab4.5_0007026g0020 | 5 | 2 | 4 | 1 | 13 | 10 | 6 | 2 | Peptide transporter-like protein |
| Nitab4.5_0002340g0070 | 9 | 3 | 4 | 10 | 16 | 27 | 16 | 10 | C4-dicarboxylate transporter_malic acid transport family protein |
| Nitab4.5_0000302g0040 | 0 | 2 | 4 | 1 | 5 | 3 | 4 | 7 | Syntaxin |
| Nitab4.5_0003264g0030 | 15 | 7 | 16 | 21 | 46 | 65 | 41 | 7 | Unknown Protein |
| Nitab4.5_0000969g0040 | 2 | 1 | 3 | 0 | 5 | 8 | 2 | 2 | U-box domain-containing protein 13 |
| Nitab4.5_0001591g0040 | 7 | 3 | 4 | 20 | 16 | 25 | 23 | 27 | Blue copper protein |
| Nitab4.5_0002816g0090 | 2 | 7 | 1 | 3 | 9 | 11 | 8 | 6 | Unknown Protein |
| Nitab4.5_0001968g0130 | 2 | 0 | 1 | 2 | 3 | 7 | 2 | 1 | Solute carrier family 15 member 4 |
| Nitab4.5_0000866g0020 | 2 | 14 | 1 | 8 | 16 | 15 | 16 | 21 | Male sterility 5 family protein (Fragment) |
| Nitab4.5_0000568g0130 | 6 | 7 | 5 | 6 | 22 | 32 | 9 | 5 | Xenotropic and polytropic retrovirus receptor |
| Nitab4.5_0000375g0160 | 1 | 1 | 1 | 2 | 2 | 3 | 4 | 6 | CBL-interacting protein kinase 13 |
| Nitab4.5_0001952g0140 | 7 | 5 | 7 | 10 | 19 | 35 | 20 | 6 | Cellulose synthase-like C1-2 glycosyltransferase family 2 protein |
| Nitab4.5_0002314g0090 | 3 | 4 | 5 | 1 | 11 | 11 | 8 | 6 | U-box domain-containing protein 4 |
| Nitab4.5_0000208g0100 | 4 | 3 | 6 | 6 | 12 | 17 | 17 | 6 | Serine_threonine-protein kinase bud32 (EC 2.7.11.1) |
| Nitab4.5_0004588g0010 | 3 | 2 | 2 | 3 | 2 | 4 | 13 | 6 | Os03g0366700 protein (Fragment) |
| Nitab4.5_0004399g0080 | 4 | 2 | 4 | 5 | 7 | 7 | 10 | 18 | Unknown Protein |
| Nitab4.5_0001748g0050 | 10 | 7 | 5 | 9 | 11 | 31 | 27 | 15 | C4-dicarboxylate transporter_malic acid transport family protein |
| Nitab4.5_0001438g0070 | 3 | 3 | 5 | 3 | 16 | 7 | 8 | 5 | Auxin-responsive protein |
| Nitab4.5_0007027g0010 | 2 | 2 | 1 | 1 | 6 | 5 | 5 | 2 | Protein kinase 5 |
| Nitab4.5_0001820g0070 | 1 | 1 | 3 | 2 | 7 | 4 | 4 | 3 | Serine carboxypeptidase K10B2.2 |
| Nitab4.5_0000080g0250 | 40 | 35 | 23 | 38 | 114 | 104 | 82 | 79 | ATP binding _ serine-threonine kinase |
| Nitab4.5_0000804g0070 | 5 | 1 | 4 | 4 | 8 | 13 | 6 | 11 | Aluminum-activated malate transporter (Fragment) |
| Nitab4.5_0001693g0130 | 2 | 1 | 1 | 2 | 3 | 5 | 6 | 4 | Aldo_keto reductase family protein |
| Nitab4.5_0001315g0320 | 2 | 2 | 1 | 0 | 4 | 4 | 6 | 1 | Os01g0786800 protein (Fragment) |
| Nitab4.5_0000116g0440 | 0 | 3 | 2 | 2 | 2 | 2 | 8 | 7 | Phosphoserine phosphatase |
| Nitab4.5_0002031g0120 | 1 | 2 | 2 | 2 | 6 | 10 | 3 | 1 | Receptor protein kinase-like protein |
| Nitab4.5_0001439g0100 | 78 | 48 | 45 | 157 | 164 | 348 | 270 | 143 | Arabinogalactan |
| Nitab4.5_0004554g0010 | 2 | 1 | 0 | 2 | 9 | 4 | 2 | 1 | Xenotropic and polytropic retrovirus receptor |
| Nitab4.5_0000136g0450 | 1 | 2 | 1 | 1 | 3 | 3 | 4 | 4 | Chromodomain-helicase-DNA-binding protein 6 |
| Nitab4.5_0003603g0040 | 175 | 52 | 20 | 146 | 352 | 419 | 275 | 80 | Expansin-like protein |
| Nitab4.5_0001423g0100 | 8 | 17 | 18 | 9 | 27 | 31 | 46 | 46 | Glucose transporter 8 |
| Nitab4.5_0001049g0080 | 1 | 2 | 1 | 1 | 5 | 1 | 4 | 3 | Survival motor neuron containing protein |
| Nitab4.5_0000509g0080 | 3 | 1 | 1 | 2 | 5 | 6 | 9 | 3 | Unknown protein DS12 from 2D-PAGE of leaf, chloroplastic |
| Nitab4.5_0002629g0030 | 112 | 81 | 42 | 211 | 280 | 501 | 309 | 221 | Fasciclin-like arabinogalactan protein 4 |
| Nitab4.5_0000535g0030 | 3 | 2 | 7 | 5 | 24 | 9 | 9 | 12 | Aluminum-activated malate transporter (Fragment) |
| Nitab4.5_0000257g0150 | 1 | 1 | 0 | 1 | 2 | 6 | 2 | 2 | IAA-amino acid hydrolase |
| Nitab4.5_0002859g0150 | 2 | 5 | 8 | 3 | 18 | 11 | 7 | 14 | D-isomer specific 2-hydroxyacid dehydrogenase |
| Nitab4.5_0001361g0130 | 1 | 1 | 2 | 1 | 1 | 6 | 4 | 2 | Ternary complex factor MIP1 |
| Nitab4.5_0000307g0100 | 2 | 0 | 1 | 1 | 6 | 4 | 1 | 3 | Pentatricopeptide repeat-containing protein |
| Nitab4.5_0000357g0250 | 2 | 2 | 1 | 0 | 2 | 3 | 4 | 4 | Exocyst complex component EXO70 |
| Nitab4.5_0000386g0240 | 5 | 1 | 7 | 8 | 15 | 27 | 15 | 7 | Digalactosyldiacylglycerol synthase 2, chloroplastic |
| Nitab4.5_0000157g0160 | 1 | 1 | 1 | 0 | 1 | 2 | 4 | 3 | 60S ribosomal protein L5, mitochondrial |
| Nitab4.5_0001789g0130 | 3 | 3 | 4 | 4 | 14 | 17 | 7 | 6 | Protein kinase domain containing protein |
| Nitab4.5_0002613g0030 | 27 | 25 | 16 | 33 | 77 | 118 | 76 | 33 | Xyloglucan endotransglucosylase_hydrolase 1 |
| Nitab4.5_0000167g0070 | 2 | 2 | 2 | 3 | 7 | 0 | 10 | 8 | RING finger protein 13 |
| Nitab4.5_0000306g0030 | 5 | 1 | 3 | 4 | 15 | 11 | 6 | 5 | Male sterility MS5 family protein |
| Nitab4.5_0001700g0060 | 22 | 11 | 10 | 29 | 60 | 87 | 55 | 14 | Expressed protein (Fragment) |
| Nitab4.5_0000678g0050 | 1 | 0 | 1 | 1 | 4 | 2 | 1 | 2 | Sulfate transporter |
| Nitab4.5_0003625g0030 | 0 | 7 | 7 | 1 | 15 | 4 | 11 | 17 | -- |
| Nitab4.5_0002356g0060 | 7 | 2 | 7 | 7 | 20 | 30 | 15 | 8 | Hydroxycinnamoyl CoA quinate transferase |
| Nitab4.5_0000137g0100 | 1 | 2 | 2 | 3 | 8 | 4 | 5 | 4 | Lipoxygenase |
| Nitab4.5_0000082g0130 | 1 | 2 | 2 | 3 | 6 | 4 | 3 | 10 | tRNA-splicing endonuclease subunit sen54 |
| Nitab4.5_0002234g0100 | 19 | 24 | 5 | 24 | 72 | 68 | 55 | 22 | BCL-2 binding anthanogene-1 |
| Nitab4.5_0000302g0200 | 2 | 0 | 1 | 4 | 6 | 6 | 4 | 4 | Myb-related transcription factor |
| Nitab4.5_0000610g0050 | 3 | 0 | 2 | 2 | 7 | 1 | 12 | 3 | Genomic DNA chromosome 5 P1 clone MMN10 |
| Nitab4.5_0001685g0060 | 1 | 4 | 1 | 0 | 5 | 3 | 7 | 5 | F-box family protein |
| Nitab4.5_0002579g0060 | 25 | 4 | 8 | 28 | 64 | 68 | 57 | 14 | Xyloglucan endotransglucosylase_hydrolase 2 |
| Nitab4.5_0000243g0040 | 1 | 0 | 3 | 3 | 8 | 6 | 6 | 1 | -- |
| Nitab4.5_0000343g0160 | 27 | 31 | 17 | 37 | 76 | 132 | 91 | 52 | Mps one binder kinase activator-like 1A |
| Nitab4.5_0000111g0120 | 3 | 1 | 4 | 0 | 6 | 4 | 7 | 7 | Pentatricopeptide repeat-containing protein |
| Nitab4.5_0000022g0430 | 2 | 0 | 2 | 1 | 2 | 4 | 4 | 7 | Sodium_calcium exchanger protein (Fragment) |
| Nitab4.5_0000678g0040 | 1 | 0 | 0 | 1 | 1 | 2 | 2 | 1 | Serine_threonine kinase |
| Nitab4.5_0002240g0060 | 2 | 0 | 3 | 1 | 4 | 6 | 5 | 4 | Lysine ketoglutarate reductase trans-splicing related 1-like |
| Nitab4.5_0003338g0080 | 4 | 2 | 3 | 2 | 15 | 6 | 6 | 10 | Rhomboid family protein |
| Nitab4.5_0000227g0020 | 84 | 48 | 49 | 97 | 256 | 327 | 226 | 90 | Receptor like kinase, RLK |
| Nitab4.5_0002022g0020 | 2 | 0 | 2 | 3 | 3 | 8 | 4 | 11 | Pathogenesis-related protein |
| Nitab4.5_0003553g0130 | 2 | 0 | 2 | 1 | 2 | 4 | 6 | 5 | Homeobox-leucine zipper protein |
| Nitab4.5_0001885g0030 | 1 | 1 | 2 | 1 | 4 | 4 | 5 | 5 | DNA-directed RNA polymerase |
| Nitab4.5_0001580g0070 | 5 | 4 | 2 | 9 | 17 | 16 | 16 | 18 | Serine_threonine-protein kinase 38 |
| Nitab4.5_0000929g0060 | 3 | 1 | 1 | 2 | 4 | 10 | 5 | 3 | Receptor like kinase, RLK |
| Nitab4.5_0002823g0080 | 5 | 0 | 2 | 1 | 7 | 7 | 9 | 3 | Receptor-like kinase |
| Nitab4.5_0001003g0160 | 2 | 2 | 1 | 0 | 3 | 3 | 7 | 4 | At1g65470_F5I14_33 (Fragment) |
| Nitab4.5_0000166g0100 | 4 | 1 | 0 | 1 | 13 | 4 | 4 | 2 | Peptide transporter-like protein |
| Nitab4.5_0000358g0030 | 5 | 2 | 5 | 3 | 14 | 5 | 13 | 18 | 60S ribosomal protein L21-like protein |
| Nitab4.5_0003384g0010 | 3 | 6 | 3 | 0 | 8 | 8 | 14 | 9 | Unknown Protein |
| Nitab4.5_0000021g1000 | 1 | 1 | 1 | 0 | 2 | 3 | 1 | 6 | Inositol-tetrakisphosphate 1-kinase 1 |
| Nitab4.5_0000232g0060 | 2 | 0 | 3 | 1 | 5 | 5 | 4 | 7 | A_IG002N01.30 protein (Fragment) |
| Nitab4.5_0001911g0060 | 1 | 1 | 1 | 1 | 2 | 4 | 2 | 2 | Pentatricopeptide repeat-containing protein |
| Nitab4.5_0001039g0020 | 1 | 0 | 1 | 4 | 7 | 6 | 7 | 4 | Os06g0524700 protein (Fragment) |
| Nitab4.5_0000002g0330 | 1 | 1 | 1 | 1 | 6 | 3 | 0 | 3 | CAS1 domain containing 1 |
| Nitab4.5_0002016g0020 | 1 | 1 | 2 | 4 | 7 | 13 | 5 | 1 | Multidrug resistance protein mdtK |
| Nitab4.5_0000145g0040 | 0 | 1 | 1 | 1 | 4 | 3 | 1 | 1 | Cc-nbs-lrr, resistance protein with an R1 specific domain |
| Nitab4.5_0000232g0150 | 3 | 2 | 1 | 7 | 13 | 5 | 12 | 12 | -- |
| Nitab4.5_0003328g0010 | 6 | 3 | 2 | 1 | 11 | 19 | 8 | 5 | Unknown Protein |
| Nitab4.5_0001816g0070 | 1 | 1 | 2 | 2 | 7 | 7 | 1 | 5 | Uncharacterized conserved membrane protein |
| Nitab4.5_0000037g0180 | 212 | 123 | 80 | 236 | 594 | 794 | 549 | 326 | Xyloglucan endotransglucosylase_hydrolase 8 |
| Nitab4.5_0000563g0180 | 6 | 19 | 11 | 8 | 35 | 52 | 46 | 19 | Cyclin-dependent protein kinase regulator Pho80 |
| Nitab4.5_0000284g0090 | 1 | 2 | 3 | 1 | 10 | 4 | 3 | 2 | Speckle-type POZ protein |
| Nitab4.5_0000103g0070 | 0 | 0 | 1 | 0 | 1 | 2 | 2 | 1 | SET and MYND domain containing 3 |
| Nitab4.5_0000986g0060 | 2 | 2 | 0 | 1 | 9 | 1 | 2 | 3 | Uncharacterized membrane protein |
| Nitab4.5_0000262g0180 | 0 | 1 | 1 | 1 | 2 | 1 | 2 | 4 | Pectinesterase |
| Nitab4.5_0000092g0030 | 2 | 1 | 1 | 1 | 5 | 7 | 10 | 1 | 4-alpha-glucanotransferase |
| Nitab4.5_0000008g0360 | 0 | 1 | 1 | 2 | 0 | 5 | 5 | 8 | Zinc finger family protein (Fragment) |
| Nitab4.5_0000351g0070 | 0 | 1 | 1 | 1 | 2 | 4 | 3 | 1 | ATP-dependent DNA helicase Ta0057, Rad3 type |
| Nitab4.5_0000209g0140 | 1 | 2 | 2 | 2 | 8 | 4 | 12 | 3 | EPIDERMAL PATTERNING FACTOR-like protein 6 |
| Nitab4.5_0005893g0010 | 29 | 17 | 24 | 23 | 64 | 125 | 95 | 55 | 1-aminocyclopropane-1-carboxylate oxidase 1 |
| Nitab4.5_0000052g0280 | 2 | 0 | 2 | 6 | 10 | 12 | 10 | 8 | Uncharacterized basic helix-loop-helix protein At1g06150 |
| Nitab4.5_0001293g0050 | 2 | 1 | 0 | 3 | 4 | 5 | 15 | 1 | Cyclin-dependent protein kinase regulator Pho80 |
| Nitab4.5_0000845g0120 | 1 | 0 | 0 | 2 | 6 | 5 | 1 | 2 | Cysteine-rich repeat secretory protein 3 |
| Nitab4.5_0003098g0060 | 1 | 2 | 0 | 1 | 1 | 4 | 3 | 5 | Mitochondrial glycoprotein |
| Nitab4.5_0000009g0500 | 1 | 0 | 1 | 3 | 5 | 6 | 3 | 3 | Expansin-like protein |
| Nitab4.5_0002287g0070 | 1 | 1 | 2 | 1 | 6 | 2 | 5 | 3 | Tetraacyldisaccharide 4_apos-kinase family protein |
| Nitab4.5_0001840g0060 | 2 | 2 | 1 | 3 | 8 | 15 | 5 | 2 | Purple acid phosphatase |
| Nitab4.5_0003976g0070 | 0 | 1 | 1 | 1 | 2 | 3 | 4 | 6 | Cyclin-dependent protein kinase regulator-like protein |
| Nitab4.5_0002252g0060 | 2 | 1 | 1 | 1 | 8 | 9 | 3 | 1 | Pollen allergen Phl p 11 |
| Nitab4.5_0001935g0050 | 1 | 0 | 1 | 1 | 5 | 3 | 2 | 1 | Prolyl 3-hydroxylase 1 |
| Nitab4.5_0000263g0090 | 2 | 1 | 11 | 2 | 15 | 27 | 12 | 11 | DNA binding protein |
| Nitab4.5_0002904g0030 | 1 | 1 | 0 | 2 | 3 | 0 | 5 | 3 | -- |
| Nitab4.5_0000101g0200 | 7 | 13 | 8 | 4 | 46 | 44 | 29 | 9 | Cytokinin riboside 5_apos-monophosphate phosphoribohydrolase LOG |
| Nitab4.5_0000244g0350 | 7 | 7 | 7 | 14 | 34 | 59 | 40 | 5 | Xyloglucan endotransglucosylase_hydrolase 9 |
| Nitab4.5_0002954g0050 | 1 | 1 | 1 | 0 | 3 | 3 | 2 | 1 | RING finger protein 24 |
| Nitab4.5_0000768g0030 | 4 | 1 | 5 | 1 | 15 | 11 | 14 | 4 | BHLH transcription factor |
| Nitab4.5_0001034g0150 | 3 | 2 | 2 | 3 | 4 | 18 | 13 | 4 | Multidrug resistance protein mdtK |
| Nitab4.5_0000479g0030 | 1 | 1 | 0 | 2 | 4 | 3 | 5 | 5 | Snurportin-like protein |
| Nitab4.5_0000980g0290 | 78 | 24 | 20 | 35 | 396 | 74 | 107 | 74 | -- |
| Nitab4.5_0002195g0010 | 8 | 0 | 9 | 1 | 13 | 34 | 20 | 9 | DNA binding protein |
| Nitab4.5_0000307g0280 | 1 | 0 | 1 | 1 | 5 | 3 | 2 | 1 | Phosphatidylinositol-4-phosphate 5-kinase family protein |
| Nitab4.5_0000790g0120 | 0 | 0 | 1 | 0 | 1 | 1 | 2 | 2 | Glycosyltransferase family 77 protein |
| Nitab4.5_0000252g0180 | 0 | 0 | 1 | 0 | 2 | 1 | 1 | 2 | Unknown Protein |
| Nitab4.5_0001039g0080 | 0 | 0 | 1 | 0 | 1 | 3 | 3 | 1 | Microtubule plus-end binding protein |
| Nitab4.5_0002152g0060 | 1 | 2 | 2 | 3 | 9 | 12 | 11 | 4 | Zinc finger family protein (Fragment) |
| Nitab4.5_0000358g0040 | 1 | 0 | 1 | 1 | 1 | 6 | 5 | 4 | Unknown Protein |
| Nitab4.5_0000934g0180 | 0 | 0 | 1 | 0 | 3 | 2 | 2 | 1 | Cyclin A-like protein |
| Nitab4.5_0002972g0030 | 0 | 1 | 2 | 0 | 3 | 2 | 4 | 4 | Protein serine_threonine kinase |
| Nitab4.5_0000071g0140 | 0 | 1 | 1 | 1 | 2 | 2 | 6 | 5 | 1-(5-phosphoribosyl)-5-((5-phosphoribosylamino)methylideneamino)imidazole-4-carboxamide isomerase |
| Nitab4.5_0001797g0070 | 1 | 0 | 1 | 3 | 9 | 7 | 4 | 3 | Squamosa promoter binding protein 3 |
| Nitab4.5_0001553g0010 | 1 | 0 | 0 | 1 | 2 | 2 | 3 | 0 | Nbs-lrr, resistance protein |
| Nitab4.5_0000184g0070 | 0 | 1 | 0 | 0 | 2 | 1 | 1 | 2 | Essential meiotic endonuclease 1B |
| Nitab4.5_0001323g0050 | 0 | 0 | 2 | 1 | 8 | 4 | 3 | 0 | -- |
| Nitab4.5_0000127g0020 | 2 | 3 | 1 | 0 | 6 | 4 | 13 | 3 | Unknown Protein |
| Nitab4.5_0000481g0010 | 5 | 2 | 0 | 1 | 10 | 20 | 8 | 1 | Ethylene-responsive transcription factor 7 |
| Nitab4.5_0000776g0070 | 0 | 1 | 1 | 1 | 2 | 2 | 2 | 4 | LRR receptor-like serine_threonine-protein kinase, RLP |
| Nitab4.5_0002321g0020 | 1 | 1 | 0 | 1 | 2 | 1 | 3 | 6 | Unknown Protein |
| Nitab4.5_0001151g0060 | 1 | 2 | 1 | 0 | 3 | 6 | 7 | 2 | Receptor-like protein kinase At3g21340 |
| Nitab4.5_0002918g0050 | 0 | 1 | 0 | 0 | 3 | 1 | 2 | 1 | Protein phosphatase 2C |
| Nitab4.5_0000396g0030 | 1 | 2 | 4 | 0 | 11 | 18 | 6 | 4 | EPIDERMAL PATTERNING FACTOR-like protein 2 |
| Nitab4.5_0004479g0040 | 0 | 0 | 1 | 1 | 5 | 4 | 3 | 0 | Telomere repeat-binding protein 4 |
| Nitab4.5_0000034g0260 | 0 | 0 | 2 | 2 | 4 | 0 | 12 | 5 | Sentrin-specific protease 2 |
| Nitab4.5_0001402g0150 | 2 | 0 | 0 | 1 | 3 | 2 | 8 | 3 | Gpi-anchor transamidase |
| Nitab4.5_0005625g0020 | 6 | 5 | 5 | 3 | 31 | 49 | 22 | 6 | Purple acid phosphatase |
| Nitab4.5_0001639g0040 | 0 | 1 | 0 | 1 | 6 | 5 | 4 | 2 | F-box family protein |
| Nitab4.5_0000849g0010 | 0 | 1 | 0 | 0 | 1 | 2 | 2 | 5 | RAG1-activating protein 1 homolog |
| Nitab4.5_0000332g0130 | 107 | 65 | 60 | 46 | 433 | 678 | 400 | 87 | -- |
| Nitab4.5_0001921g0030 | 4 | 0 | 4 | 3 | 30 | 21 | 8 | 2 | Phi-1 protein (Fragment) |
| Nitab4.5_0002716g0020 | 3 | 0 | 1 | 0 | 15 | 6 | 3 | 4 | Unknown Protein |
| Nitab4.5_0000563g0300 | 0 | 0 | 0 | 2 | 2 | 2 | 2 | 8 | Blue copper protein |
| Nitab4.5_0000725g0060 | 0 | 0 | 0 | 1 | 5 | 1 | 0 | 2 | O-methyltransferase 1 |
| Nitab4.5_0000848g0010 | 0 | 0 | 2 | 0 | 8 | 5 | 5 | 0 | Glutathione S-transferase |
| Nitab4.5_0001531g0030 | 1 | 0 | 0 | 0 | 2 | 1 | 3 | 4 | L-lactate dehydrogenase |
| Nitab4.5_0000980g0200 | 0 | 0 | 0 | 0 | 2 | 0 | 2 | 1 | Tripeptidyl peptidase II |
| Nitab4.5_0000065g0070 | 0 | 1 | 1 | 2 | 9 | 8 | 0 | 11 | -- |
| Nitab4.5_0001529g0020 | 0 | 0 | 0 | 2 | 2 | 8 | 5 | 1 | Protease inhibitor_seed storage_lipid transfer protein family protein |
| Nitab4.5_0004977g0030 | 0 | 0 | 0 | 2 | 0 | 4 | 4 | 11 | Unknown Protein |
| Nitab4.5_0001329g0130 | 0 | 0 | 0 | 0 | 2 | 1 | 5 | 1 | DNA repair and recombination protein radA |
| Nitab4.5_0001365g0050 | 0 | 0 | 0 | 1 | 4 | 1 | 0 | 4 | Epidermal growth factor receptor substrate 15 |
| Nitab4.5_0003061g0020 | 0 | 0 | 0 | 0 | 3 | 2 | 0 | 2 | Myb-related transcription factor |
| Nitab4.5_0000401g0220 | 0 | 0 | 0 | 0 | 0 | 2 | 3 | 1 | HAPp48 5 protein (Fragment) |
| Nitab4.5_0001642g0050 | 0 | 0 | 1 | 2 | 6 | 9 | 7 | 2 | Gibberellin-regulated protein 2 |
| Nitab4.5_0001756g0030 | 0 | 0 | 0 | 0 | 4 | 2 | 0 | 3 | Protection of telomeres 1 protein |
| Nitab4.5_0000343g0220 | 0 | 0 | 0 | 0 | 4 | 3 | 0 | 1 | Unknown Protein |
| Nitab4.5_0003374g0070 | 0 | 0 | 0 | 0 | 0 | 0 | 3 | 2 | BHLH transcription factor |
| Nitab4.5_0000850g0170 | 0 | 0 | 1 | 0 | 4 | 0 | 6 | 3 | Actin-related protein 2_3 complex subunit 4 |
| Nitab4.5_0001239g0070 | 0 | 0 | 0 | 0 | 3 | 2 | 0 | 0 | Unknown Protein |
| Nitab4.5_0002438g0030 | 0 | 0 | 0 | 0 | 2 | 8 | 4 | 0 | High affinity sulfate transporter 2 |

**Table** **D. Primers used in this study**

| **Genes** | **Forward primer (5’-3’)** | | Reverse primer (5’-3’) |  |  |
| --- | --- | --- | --- | --- | --- |
| **Primers used in qPCR** | |  |  |  |  |
| *BtActin* | TCTTCCAGCCATCCTTCTTG | | CGGTGATTTCCTTCTGCATT |  |  |
| *BtRDP* | TTGGCTTTCCTTGTCCTCGC | | CGTCGCAGAGTTCGTAGTCA |  |  |
| *Nttubulin* | AAGTACATGGCTTGCTGCCT | | ATCAATGCGCGAGAAGACCT |  |  |
| *NtGAPDH* | GCAGTGAACGACCCATTTATCTC | | AACCTTCTTGGCACCACCCT |  |  |
| *NtPAL* | AAGAAGCGTTCCGTGTTGCTG | | TCGGGCTTTCCATTCATCACC |  |  |
| *NtNPR1* | GCTGTAGCGTTCCTTGTTGA | | AGGCCTTATCAAGGGTTATG |  |  |
| *NtFAD7* | CATGTGGCTTGACTTAGTTACCTACT | | CCCTGACTTCTTTGGCTCCTT |  |  |
| *NtPDF1.2* | GGAAATGGCAAACTCCATGCG | | ATCCTTCGGTCAGACAAACG |  |  |
| *NtRLP4* | ATTCCTGAAAGCCTTGGGCA | | AGCGTAAGATGGGTTCCACA |  |  |
| *NtSOBIR1* | GCGAAAGGCAAACAGAACACA | | GGACCTCCAGTTTACGGCAT |  |  |
| *SlRLP4* | TGACCTCATTACGGACGCTG | | CATGCCCCTAATCCGATCCC |  |  |
| *OsRLP4* | TCTTGACAGCGAGCCGATTT | | TGGATCACCATGACCAGTGC |  |  |
| *NlSP104* | GTCTTAGCCGTGTGCATAGC | | TGAAGAGGCGGATTGTTGGA |  |  |
| **Primers used in double stranded RNA synthesis** | | | |  |  |
| *GFP* | TAATACGACTCACTATAGGGAGAATGAGTAAAGGAGAAGAACTTTTC | | TAATACGACTCACTATAGGGAGATTTGTATAGTTCATCCATGCCATGT |  |  |
| *BtRDP* | TAATACGACTCACTATAGGGTTGGCCGTCCCAACCGCCACCC | | TAATACGACTCACTATAGGGTCACAAGTAGATGCTCGGGTTG |  |  |
| *NlSP104* | TAATACGACTCACTATAGGGAACCCCAACCCGAAGCCGAT | | TAATACGACTCACTATAGGGCTACAAGAAAGGCATGTATTGTC |  |  |
| **Primers used in binary vector construction** | | | | |  |
| *BtRDP-flag/mCherry* | ATGCACAAATTATTGGCTTTCCATGCACAAATTATTGGCTTTCC | | GAGGAGAAGAGCCGTCGCAAGTAGATGCTCGGGTTG |  |  |
| *BtRDP^-sp^- flag* | CGACGACAAGACCGTCACCATGTTGGCCGTCCCAACCGCCACCC | | GAGGAGAAGAGCCGTCGCAAGTAGATGCTCGGGTTG |  |  |
| *NtRLP4-myc/gfp* | CGACGACAAGACCGTCACCATGATGAGATTCCACTATGGTTTCT | | GAGGAGAAGAGCCGTCGGGTAAGCAAGGGAGGTC |  |  |
| *NtRLP4-mCherry* | CGACGACAAGACCGTCACCATGGATCCATATGTAATGCGAATAAGC | | GAGGAGAAGAGCCGTCGGGTAAGCAAGGGAGGTC |  |  |
| *NtSOBIR1-gfp* | GACGAGCTGTACAAGGGTACCATGGCCTTCACTGCTTCACAAATCC | | GCGGACTCTAGTTCATCTAGATTAATGCTTGATCTGAGTTAAC |  |  |
| *NtSOBIR1-flag* | CGACGACAAGACCGTCACCATGAAACTAAATCTCTATCCACC | | GAGGAGAAGAGCCGTCGATGCTTGATCTGAGTTAAC |  |  |
| *RFP-mCheery* | CTTCGACGACAAGACCGGGCCCATGGCCTCCTCCGAGAACGTCA | | AGTGAGGAGAAGAGCCGGGCCCACAGGAACAGGTGGTGGCGG |  |  |
| *NLSP104-flag* | CGACGACAAGACCGTCACCATGCATCCAAGAACGATCATCCG | | GAGGAGAAGAGCCGTCGTGCCGGACTACCCCCACCT |  |  |
| *BtFTSP-flag* | CGACGACAAGACCGTCACCATGTCAGCCCTAAGCTTTACTG | | GAGGAGAAGAGCCGTCGCAAGAAAGGCATGTATTGTC |  |  |
| *SLRLP4-myc* | CGACGACAAGACCGTCACCATGCGCCATGAGCCATATGTAATG | | GAGGAGAAGAGCCGTCGGGTAAGCAAGGGAGGTCC |  |  |
| *OsRLP4-myc* | CGACGACAAGACCGTCACCATGGCGGATCCTAGCAAAGAGCC | | GAGGAGAAGAGCCGTCGGGAAGGAAGCAAATGTGG |  |  |
|  |  | |  |  |  |
| **Primers used in Y2H vector construction** | | | |  |  |
| *AD-NtRLP4_(23-541）_* | GTACCAGATTACGCTCATATGGATCCATATGTAATGCGAATAAGC | | CAGCTCGAGCTCGATGGATCCTTAACACGTTGGTAATCCCG |  |  |
| *AD-NtRLP4_(31-336）_* | GTACCAGATTACGCTCATATGATAAGCTGTGGAGCTCGAC | | CAGCTCGAGCTCGATGGATCCTTAAATCTCAAAAATTTC |  |  |
| *AD-NtRLP4_(375-541）_* | GTACCAGATTACGCTCATATGCCTGACGAAGTCAAGGG | | CAGCTCGAGCTCGATGGATCCTTAACACGTTGGTAATCCCG |  |  |
| *AD-NtRLP4_(573-625）_* | GTACCAGATTACGCTCATATGGGAACCCATCTTACGCTTG | | CAGCTCGAGCTCGATGGATCCTCAGGTAAGCAAGGGAGG |  |  |
| *AD-SLRLP4_(23-546）_* | GTACCAGATTACGCTCATATGATAAGCTGTGGAGCTCGAC | | CAGCTCGAGCTCGATGGATCCTTAATCATGTAAGATGTGTTCCGCAAG |  |  |
| *AD-OsRLP4_(29-551）_* | GTACCAGATTACGCTCATATGGCGGATCCTAGCAAAGAGCC | | CAGCTCGAGCTCGATGGATCCTGCCGCATTCATGTAAACCGG |  |  |
| *AD-OsRLP4_(40-374）_* | GTACCAGATTACGCTCATATGATAAGCTGTGGGAGTTTTG | | CAGCTCGAGCTCGATGGATCCTCTCAAAGACCTCAATAGC |  |  |
| *AD-OsRLP4_(377-551）_* | GTACCAGATTACGCTCATATGGGCCGAAAAGAAAACTTTAAC | | CAGCTCGAGCTCGATGGATCCTGCCGCATTCATGTAAACCGG |  |  |
| *AD-OsRLP4_(552-582）_* | GTACCAGATTACGCTCATATGCCGCATTTATCTGTGGCTGC | | CAGCTCGAGCTCGATGGATCCTCTAGGAAGGAAGCAAATGTGG |  |  |
| *BK-BtRDP^-sp^* | TCAGAGGAGGACCTGCATATGTTGGCCGTCCCAACCGCCACCC | | CCGCTGCAGGTCGACGGATCCTCACAAGTAGATGCTCGGGTTG |  |  |
| *BK-BtSP37.4^-sp^* | TCAGAGGAGGACCTGCATATGGACAGTGGAGCTACAGCGCC | | CCGCTGCAGGTCGACGGATCCTCTAAAAGTGGTGGGTGGTTTGT |  |  |
| *BK-BtSP16.3^-sp^* | TCAGAGGAGGACCTGCATATGTTGCCGTCGAAGGAACCTGGA | | CCGCTGCAGGTCGACGGATCCAGAGAGCACCATTTAAGATCCTC |  |  |
| *BK-BtFTSP^-sp^* | TCAGAGGAGGACCTGCATATGGGCAAAGATGAAGGCAAAGG | | CCGCTGCAGGTCGACGGATCCTTATGCCGGACTACCCCCAC |  |  |
| *BK-NLSP104^-sp^* | TCAGAGGAGGACCTGCATATGAACCCCAACCCGAAGCCGAT | | CCGCTGCAGGTCGACGGATCCCTACAAGAAAGGCATGTATTGTC |  |  |
| *BK-NlSP32030^-sp^*  (XP_039291719.1) | TCAGAGGAGGACCTGCATATGAAATATGTAACCTCGGCAGTGC | | CCGCTGCAGGTCGACGGATCCTTATTGCTTGGTGGCCCCTGCAG |  |  |
| *BK-NlSP706^-sp^*  (MF278706.1) | TCAGAGGAGGACCTGCATATGACTAGCGATGACGATTGCAGAG | | CCGCTGCAGGTCGACGGATCCTTACAAAAGTCCCATATCTGCTAA |  |  |
| *BK-NlSP8 ^-sp^*  (KU365967.1) | TCAGAGGAGGACCTGCATATGAGGTATCCTGGCTTTGGCGGG | | CCGCTGCAGGTCGACGGATCCCTATGGGAATCCTGGGTATGG |  |  |
| *BK-NlSP711 ^-sp^*  (MF278711.1) | TCAGAGGAGGACCTGCATATGGCAAGTACGGACATGGAATTC | | CCGCTGCAGGTCGACGGATCCTCAGAAAGTAACATCCATTCCC |  |  |
| *BK-NlSP714 ^-sp^*  (MF278714.1) | TCAGAGGAGGACCTGCATATGGAAACGTCTGAAGTCTACCTC | | CCGCTGCAGGTCGACGGATCCTTATTCGTTTAGAATTCTGGCAAG |  |  |
| *BK-NlSP715 ^-sp^*  (MF278715.1) | TCAGAGGAGGACCTGCATATGACGGGAAAATTTGATTTTGGAG | | CCGCTGCAGGTCGACGGATCCTCAAGCACTGATTTTCGCTTC |  |  |
| *BK- NlSP720^-sp^*  (MF278720.1) | TCAGAGGAGGACCTGCATATGGTACAGGAATCTAAATCATGGGC | | CCGCTGCAGGTCGACGGATCCTCACTTAAAAAGCGGACTGTAG |  |  |
| *BK-NlSP28 ^-sp^*  (XP_022195702.1) | TCAGAGGAGGACCTGCATATGACGAAGGGCGTAGAAGATGTG | | CCGCTGCAGGTCGACGGATCCTAGTACTTGGGGATTGGAATC |  |  |
| *BK-NlSP474 ^-sp^*  (XP_022196818.2) | TCAGAGGAGGACCTGCATATGTGTGTGGAGGGTACAGCAGC | | CCGCTGCAGGTCGACGGATCCTCACGAGAATTTCGCATTCCC |  |  |
| *BK-NlSP170 ^-sp^*  (XP_039284081.1) | TCAGAGGAGGACCTGCATATGTCTGGAACAGCAATGACTTCTG | | CCGCTGCAGGTCGACGGATCCCCACCACCAATAATAAGGTACT |  |  |
| *BK-NlSP2 ^-sp^*  (XP_022192221.2) | TCAGAGGAGGACCTGCATATGGCAGCTGTGGACCTTAGCTTC | | CCGCTGCAGGTCGACGGATCCTCAACTGAAATCGACATCTCC |  |  |
| *BK-NlSP3244 ^-sp^*  (XP_022207944.2) | TCAGAGGAGGACCTGCATATGGCGAATATTGCTGACCATGGA | | CCGCTGCAGGTCGACGGATCCCAATTGATAGTCAGTTTGACG |  |  |
| *BK-NlSHP ^-sp^*  (XP_022207944.2) | TCAGAGGAGGACCTGCATATGTTCCCATTTCCACTTCTCAGCC | | CCGCTGCAGGTCGACGGATCCTTAGAAGGTCAATGACTGGAAGG |  |  |
| *BK-LsSP397 ^-sp^*  (RZF44823.1) | TCAGAGGAGGACCTGCATATGGAATCAGATGATGTGCCGGTC | | CCGCTGCAGGTCGACGGATCCCTATGCAGCTGGTGGTGTTGTG |  |  |
| *BK-LsSP19899 ^-sp^*  (RZF42644.1) | TCAGAGGAGGACCTGCATATGGATTGCGAGACGAAACCGCATC | | CCGCTGCAGGTCGACGGATCCTTATCCAGCATTTCCAACCAAG |  |  |
| *BK-LsSP3 ^-sp^*  (RZF33006.1) | TCAGAGGAGGACCTGCATATGCGTTACGCCTCATATGAAAAGC | | CCGCTGCAGGTCGACGGATCCCTAACATTTTCCACATTTTCCC |  |  |
| *BK-LsSP4 ^-sp^*  (RZF48570.1) | TCAGAGGAGGACCTGCATATGGTGACATGTTTCCCGTTCCCC | | CCGCTGCAGGTCGACGGATCCTTAGAAGGTCAATGACATGAC |  |  |
| *BK-LsSP5 ^-sp^*  (RZF42817.1) | TCAGAGGAGGACCTGCATATGGCGGCTGTGCTACCAGCTAAC | | CCGCTGCAGGTCGACGGATCCTCAAGGTATGGGTGGAGTTAGG |  |  |
| *BK-LsSP6 ^-sp^*  (RZF33751.1) | TCAGAGGAGGACCTGCATATGGGGCCCAAATCCGACCGGAAAC | | CCGCTGCAGGTCGACGGATCCTTAGTAGACAAGTTGTGGTTGC |  |  |
| **Primers used in prokaryotic expression vector construction** | | | |  | |
| *BtRDP^-sp^-his* | CTGGTGCCGCGCGGCAGCCATATGTTGGCCGTCCCAACCGCCACCC | | ACGGAGCTCGAATTCGAATCCTCACAAGTAGATGCTCGGGTTG |  | |
| *GFP-his* | cagcaaATGGGTCGCGGATCCATGGTGAGCAAGGGCGAGGAG | | gtggtggtggtggtgctcgagTGTACAGCTCGTCCATGCCGA |  | |
| **Primers used in BIFC vector construction** | | | |  |  |
| *nYFP-NtRLP4* | ATCGAGGACTCCGGAGTCGACATGGATCCATATGTAATGCGAATAAGC | | GATCGGGGAAATTCGAGCTCTCAGGTAAGCAAGGGAGGTC |  |  |
| *nYFP-NtCf-9* | CTGTACAAGTCCGGAGTCGACATGGATTATGAAAATCTTGCA | | GATCGGGGAAATTCGAGCTCTCTAATATCTTTTCTTGTGTTTTTTC |  |  |
| *cYFP-BtRDP^-sp^* | CTGTACAAGTCCGGAGTCGACATGTTGGCCGTCCCAACCGCCACCC | | GATCGGGGAAATTCGAGCTCTCACAAGTAGATGCTCGGGTTG |  |  |
| *cYFP-BtFTSP^-sp^* | CTGTACAAGTCCGGAGTCGACATGGGCAAAGATGAAGGCAAAG | | GATCGGGGAAATTCGAGCTCTTATGCCGGACTACCCCCAC |  |  |
